# Evolutionary Genomics Suggests That CheV Is an Additional Adaptor for Accommodating Specific Chemoreceptors within the Chemotaxis Signaling Complex

**DOI:** 10.1371/journal.pcbi.1004723

**Published:** 2016-02-04

**Authors:** Davi R. Ortega, Igor B. Zhulin

**Affiliations:** 1 Computer Science and Mathematics Division, Oak Ridge National Laboratory, Oak Ridge, Tennessee, United States of America; 2 Department of Microbiology, University of Tennessee, Knoxville, Tennessee, United States of America; Pierre and Marie Curie University (UPMC), FRANCE

## Abstract

*Escherichia coli* and *Salmonella enterica* are models for many experiments in molecular biology including chemotaxis, and most of the results obtained with one organism have been generalized to another. While most components of the chemotaxis pathway are strongly conserved between the two species, *Salmonella* genomes contain some chemoreceptors and an additional protein, CheV, that are not found in *E*. *coli*. The role of CheV was examined in distantly related species *Bacillus subtilis* and *Helicobacter pylori*, but its role in bacterial chemotaxis is still not well understood. We tested a hypothesis that in enterobacteria CheV functions as an additional adaptor linking the CheA kinase to certain types of chemoreceptors that cannot be effectively accommodated by the universal adaptor CheW. Phylogenetic profiling, genomic context and comparative protein sequence analyses suggested that CheV interacts with specific domains of CheA and chemoreceptors from an orthologous group exemplified by the *Salmonella* McpC protein. Structural consideration of the conservation patterns suggests that CheV and CheW share the same binding spot on the chemoreceptor structure, but have some affinity bias towards chemoreceptors from different orthologous groups. Finally, published experimental results and data newly obtained via comparative genomics support the idea that CheV functions as a “phosphate sink” possibly to off-set the over-stimulation of the kinase by certain types of chemoreceptors. Overall, our results strongly suggest that CheV is an additional adaptor for accommodating specific chemoreceptors within the chemotaxis signaling complex.

## Author Summary

Due to the overwhelming complexity and diversity of biological systems, the functional roles of the majority of proteins encoded in sequenced genomes remain unknown or poorly understood. The multi-protein pathway controlling chemotaxis in bacteria and archaea is an example of such complexity and diversity. Chemotaxis pathway in *E*. *coli* is one of the best understood signal transduction networks in nature; however, this model organism lacks some of the system components, such as CheV, that are found in many other species. The biological role of CheV is still under avid debate. CheV is an auxiliary component of many chemotaxis systems and is present in important human pathogens, such as *Salmonella* and *Helicobacter*, where chemotaxis is being studied as an important virulence trait. Here we established the evolutionary history of the chemotaxis pathway in enterobacteria and combined a computational genomics approach with available structural information to propose a role for CheV. Our results show that CheV in enterics evolved as an adaptor for a specific type of chemoreceptors. Furthermore, we propose that some CheV-associated chemoreceptors might increase the kinase activity above the base level, and in these cases CheV acts as an attenuator.

## Introduction

Bacteria navigate in chemical gradients by regulating their flagellar motility. This behavior, known as chemotaxis, is characterized by high sensitivity and precise adaptation that are attributed to the underlying molecular machinery, which is best understood in the model organism *Escherichia coli* [1, 2]. Dedicated chemoreceptors (methyl-accepting chemotaxis proteins or MCPs), the CheW adaptor protein and the CheA histidine kinase form a self-organized protein complex [3–5]. Upon changes in concentrations of specific chemical cues, chemoreceptors modulate the kinase activity which in turn controls the flagella rotation via phosphorylation of the response regulator CheY [6]. Thus, MCPs, CheW, CheA, and CheY comprise an excitation pathway in chemotaxis which delivers the signal from a stimulus to the flagellar motor. The CheR methyltransferase and the CheB methylesterase that covalently modify MCPs encompass an adaptation pathway. Methylation enhances CheA activity, whereas demethylation reduces it [6]. The system also has the CheZ phosphatase, which dephosphorylates CheY leading to signal termination. *E*. *coli* has five chemoreceptors. Tar mediates attractant responses to aspartate and maltose [7, 8] and negative chemotaxis to metals [9]. Tsr governs attractant responses to serine [7] and quorum sensing autoinducer AI-2 [10], as well as chemotaxis to oxygen, redox, and oxidizable substrates [11, 12]. Trg mediates attractant responses to ribose and galactose [13]. Tap initiates attractant responses to dipeptides [14] and pyrimidines [15]. Aer mediates responses to oxygen and energy taxis [11, 12, 16]

Because of its close relatedness to *E*. *coli*, *Salmonella enterica* serovar Typhimurium has been a model for many experiments in chemotaxis and most of the results obtained with one organism have been generalized to another (reviewed in [1, 2, 17, 18]. The functional similarity among components of the chemotaxis system in the two species is remarkable [19]. However, there are also some noticeable differences. *S*. *enterica* has the CheV protein, which is not found in *E*. *coli*, and it also has a larger number of chemoreceptor genes than *E*. *coli* does. CheV is a fusion of the CheW domain with a response regulator domain similar to CheY. It is postulated to interact with chemoreceptors and CheA as a docking protein similarly to CheW and might play a role in signaling adaptation, as shown in another model organism, *Bacillus subtilis* [20, 21]; however, the precise role of CheV is not understood [22] despite of being present in approximately 60% of all sequenced genomes with chemotaxis systems. In fact, all chemotaxis systems identified in prokaryotes contain either CheW or CheV or both [23] and experimental evidence established their role as coupling proteins (also referred to as adaptors or scaffold proteins) in several model organisms including *E*. *coli* [24], *S*. *enterica* [25], *B*. *subtilis* [20], and *Helicobacter pylori* [26]. The CheW domain is topologically similar to SH3 domains [27] from eukaryotic scaffold proteins that also play a key role in signal transduction [28].

*S*. *enterica* lacks Tap, but has five chemoreceptors that are not present in *E*. *coli*. Tcp mediates attractant responses to citrate and repellent responses to phenol [29]. McpB and McpC mediate repellent responses to cysteine [30]. Function of two other chemoreceptors, Tip [31] and McpA [32] remains unknown. Why does *E*. *coli* have one adaptor and *S*. *enterica* has two? Is there a connection between having an extra adaptor (CheV) and extra MCPs that are present in *Salmonella*? We hypothesized that the function of CheV might be in accommodating certain types of MCPs that cannot be effectively accommodated by CheW. Here, we set up a series of comparative genomics studies to explore this hypothesis and to gain new insights about evolution and the biological function of the CheV protein in the chemotaxis protein complex.

## Results and Discussion

### CheV and the number of MCPs are the two major variances in *Enterobacteriales* chemotaxis machinery

**Fig 1:**
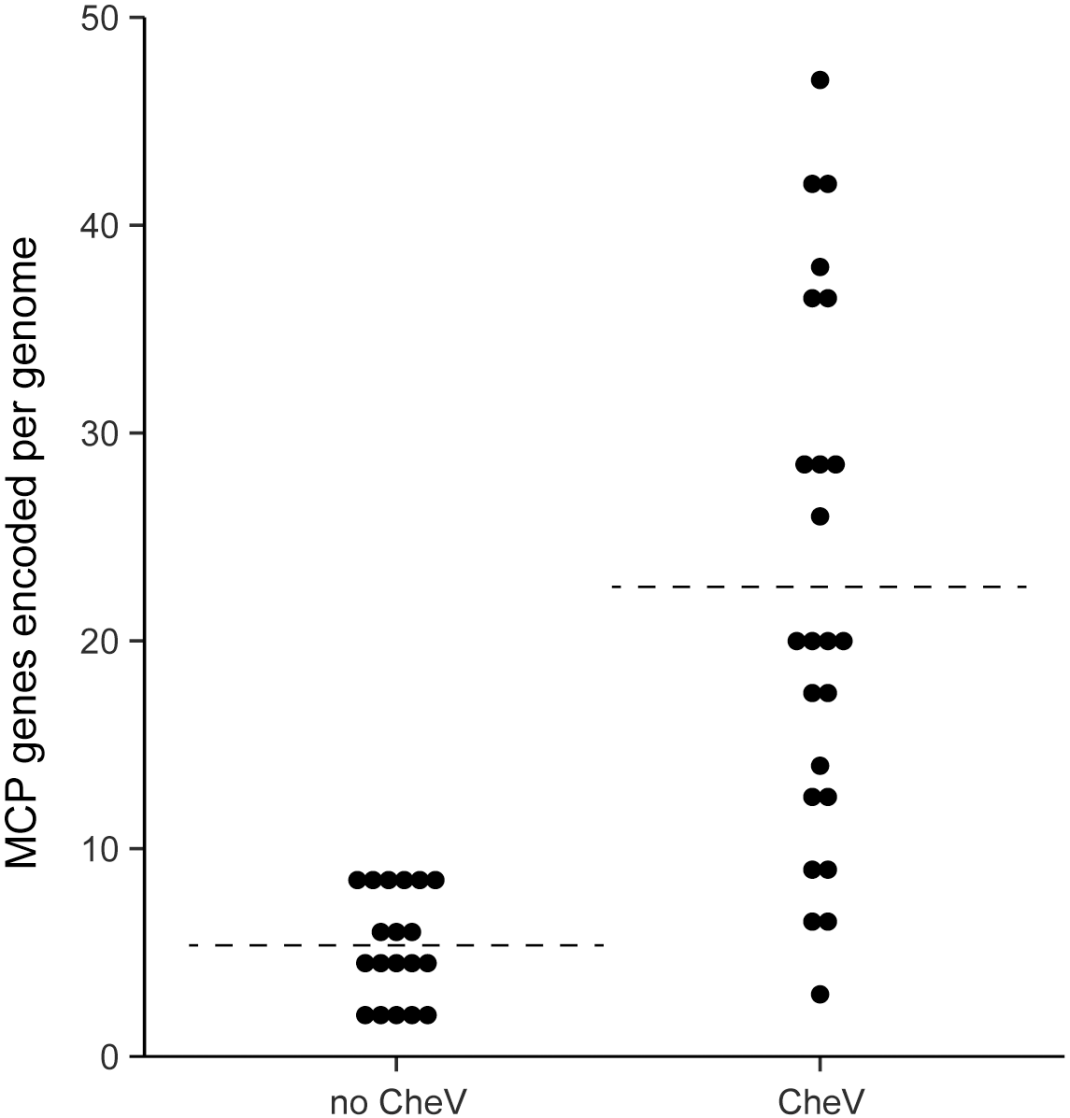
Number of MCP genes in 43 enterobacterial genomes with and without CheV. Each dot represents a genome. The dashed lines indicate the average number of MCPs for each distribution.

In order to understand the differences that are observed in *E*. *coli* and *S*. *enterica*, we have analyzed the set of chemotaxis machinery components in all of their close relatives for which genome information was available. The 213 complete genomes of *Enterobacteriales* available in the MiST2.2 database [33] were collected and analyzed for the presence of chemotaxis genes (S1 Table). Essentially all the genomes contain one copy of each of the key chemotaxis proteins: CheA, CheW, CheB, CheR and CheZ. The only exception was a subset of eight closely related *Erwinia* and *Enterobacter* species, where an apparent duplication of the nearly entire chemotaxis operon took place (S1 Table). Consequently, these genomes were excluded from analysis. A non-redundant set of 43 genomes (one representative of each species, randomly chosen, except for *E*. *coli* and *S*. *enterica* strains used as models in chemotaxis studies) was analyzed further (S1 Table). The only two variances among the chemotaxis systems of enterobacteria mirror those seen in *E*. *coli* and *S*. *enterica*: (i) the presence of CheV in some genomes and (ii) the number of MCP genes per genome (S1 Table). On average, the analyzed genomes of *Enterobacteriales* contain 15 chemoreceptor genes per genome (ranging from 2 in *Enterobacter aerogenes* and few other species to 42 in *Pantoea ananatis*). However, there was a major difference between genomes encoding CheV and genomes without CheV. Genomes without CheV contain on average only 5 chemoreceptor genes (ranging from 2 to 9); whereas genomes with CheV contain on average 23 chemoreceptor genes (ranging from 3 to 42) (Fig 1). The direct relationship between the large number of chemoreceptors and the presence of CheV suggests the hypothesis that the CheV adaptor might be necessary to accommodate certain chemoreceptors. This hypothesis is in line with the previous report that CheV might be a preferential adaptor for the aspartate chemoreceptor in *Campylobacter jejuni* [34].

To further investigate this hypothesis, we employed a series of comparative genomic approaches. Interpretation of results obtained by these methodologies strongly depends on the evolutionary history of the analyzed genes and the suitability of the dataset. For example, phylogenetic profiling would strongly benefit from independent events of gene loss in an analyzed dataset, because if the products of two genes interact, then the loss of one gene should coincide with the loss of another. Consequently, we analyzed the evolutionary history of the chemotaxis pathway in *Enterobacteriales* to ensure the dataset is suitable for this type of analysis.

We have compared topologies of the maximum-likelihood phylogenetic trees built from ribosomal 16S gene and CheA protein sequences. The nearly identical overall tree topologies and consistency within corresponding clades on both trees strongly suggest that the chemotaxis system in *Enterobacteriales* evolved vertically without any instances of a horizontal transfer of the *cheA* gene (S1 Fig). To understand the CheV evolution within *Enterobacteriales*, we have constructed a maximum-likelihood tree from aligned CheV protein sequences and compared its topology with that generated from CheA sequences (S2 Fig). The nearly identical topology and consistency within clades indicate the ancestral origins and vertical evolution of CheV in *Enterobacteriales* suggesting that CheV was present in their last common ancestor. This means that enterobacterial genomes without the *cheV* gene lost it during the course of evolution. We took advantage of this relatively balanced sample of closely related genomes to perform comparative analysis of sequence profiles in order to gain insights into CheV biological function and to identify its potential interacting partners within the chemotaxis pathway.

### Interaction between CheV and CheA

CheV has a response regulator domain (CheV_RR_), which is homologous to CheY protein [20, 22]. CheY can bind to P1 and P2 domains of CheA (here called CheA_P1_ and CheA_P2_ respectively). The P1 domain (also known as the histidine phosphotransfer or Hpt domain) contains a conserved histidine, from which a phosphate group is transferred to CheY; the P2 domain was proposed to be a docking module for CheY [35]. Consequently, we considered the hypothesis that CheV_RR_ can potentially bind to the same domains. The absence of CheV in the genome should change the conservation pattern in its interaction partners, CheA and MCPs, due to relaxing evolutionary pressure on residues that are involved in interaction with CheV. Analysis of multiple sequence alignment of CheA_P2_ domains of CheA (S3 Fig) shows that there is no significant difference in conservation pattern between sequences from genomes with and without CheV (S4 Fig). This suggests that CheV does not interact with CheA_P2_. Furthermore, CheA_P2_ is absent from many CheA proteins. We have analyzed more than 3000 bacterial and archaeal genomes for the presence and absence of CheV and the CheA_P2_ domain. We found no correlation between the presence of CheV and CheA_P2_. There are 2252 genomes with at least one CheA_P2_ domain in the CheA sequences and 1772 genomes with at least one CheV. Only 729 of these genomes contain both CheA_P2_ and CheV, which provides evidence that CheV and CheA_P2_ do not co-evolve. Because interacting proteins and domains are likely to co-evolve (36), observed distribution suggests that CheV does not interact with the CheA_P2_ domain.

**Fig 2:**
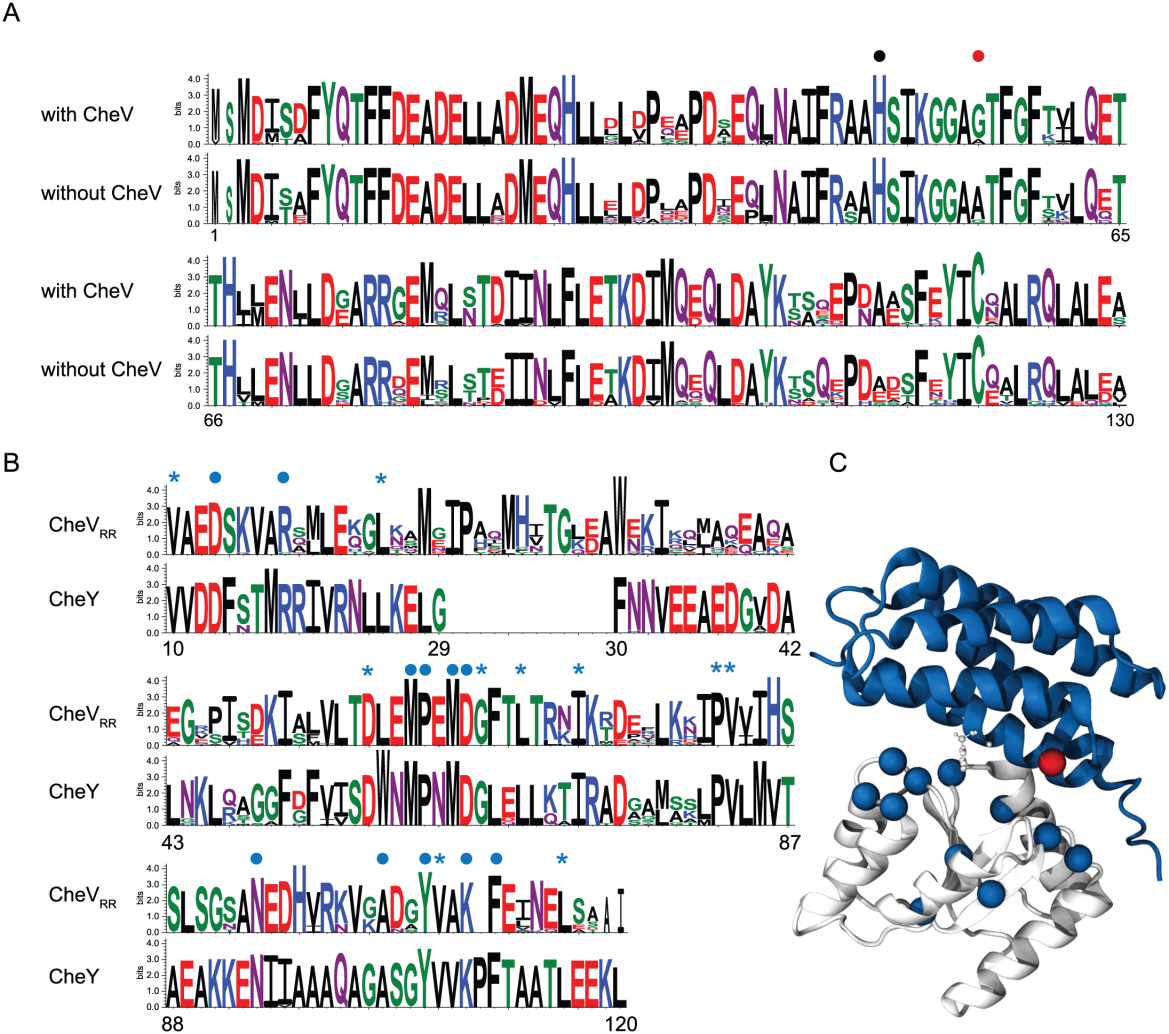
Analysis of patterns in sequence conservation suggests interaction between CheV_RR_ domain and CheA-P1. A) Comparison between sequence logos of CheA_P1_ from genomes with and without CheV. The CheA_P1_ active site His48 (black dot) and the only different position between the two sets Gly55 (red dot) are marked. B) Comparison between sequence logos of CheY and the CheV_RR_ domain. Positions that are conserved in both sets are marked (blue dots for solvent exposed positions (10 25 57 65 68 72 82 83 107 116) and blue stars for buried positions (13 18 60 61 63 64 94 103 106 109 111)). C) Cartoon representation of the CheY (white) and CheA_P1_ (blue) [35]. Solvent exposed positions conserved in CheY and CheV_RR_ datasets localize to the protein interface region (blue spheres). The single position that is different between the sets of CheA_P1_ with and without CheV, Gly55 (red sphere), lays in the C-terminal part of the second α-helix involved in the interaction protein region that also contains the active site His48 (white CPK representation).

On the other hand, the analysis of conservation patterns in multiple sequence alignment of the CheA_P1_ domain (S5 Fig) in genomes with and without CheV shows a nearly absolute conservation between the two groups with only one position significantly different (Fig 2A). The position 55 (numbers for CheA protein in *E*. *coli*) is occupied by a glycine in organisms with CheV, which is changed to an alanine in organisms without CheV. This observation indicated that the CheV_RR_ domain might interact with CheA_P1_. To explore this possibility further, we aligned the CheY proteins (known to interact with CheA_P1_) from the genomes with CheV protein and compared with the alignment of the CheV_RR_ domain from the same organisms (S6 Fig). The conservation within each group (CheY and CheV_RR_) is very high, however, only 21 out of 127 positions (less than 20% identity) are shared by both groups and only 11 of these positions are accessible to solvent and thus may participate in the interaction (Fig 2B). We mapped the relevant residues into the proposed interaction model between CheY and CheA_P1_ for *E*. *coli* [35](PDB code: 2LP4) as a model interaction between CheV_RR_ and CheA_P1_ (Fig 2C). The only significantly different position in CheA_P1_ domains from genomes with and without CheV, the Gly55, lays on the C-terminal part of the second α-helix of the structure of CheA_P1_ close to the active site for CheY, His48, within the known binding region of CheY in *E*. *coli*. Moreover, mapping the solvent exposed residues that are common to both CheY and CheV_RR_ onto the CheY structure shows that they are localized primarily around the CheA_P1_ binding region (Fig 2). Taken together, these results support the hypothesis that CheV_RR_ interacts with CheA via its P1, but not P2, domain.

In addition to the response regulator domain, CheV also contains an adaptor domain CheW (CheV_W_). Interestingly, the P5 domain of the histidine kinase (CheA_P5_), also known as the regulatory domain, is a CheW domain as well [17, 27]. The current model for the arrangement of the chemotaxis protein complex encompassing CheA-CheW-MCP proposes two distinct interaction surfaces between CheA_P5_ domain and the CheW protein forming a CheW domain hexagonal ring with three CheA proteins and three CheW proteins [36, 37]. As postulated above, we assume that CheV is an adaptor protein similarly to CheW. Then, it is reasonable to assume that CheV_W_ would be a part of the same CheW domain network in the chemotaxis complex patch. Surprisingly, using the computational approach described above, we did not identify any significant difference in conservation pattern between the sequences of CheW proteins from genomes with and without CheV (S7 Fig). The same result was obtained for the CheA_P5_ protein domain (S7 Fig). Thus, these results do not support the idea that CheV participates in the complex array as a part of the CheW–CheA_P5_ hexagonal ring. On the other hand, it has been shown previously that CheW from evolutionarily distant species can rescue a system with a *cheW* knockout, despite the low level of identity between the homologs [38] Thus, an alternative explanation, which opens the possibility for CheV_W_ to be a part of the array, is that the CheW fold evolved to maintain interactions between the adaptor domains CheV_W_, CheW and CheA_P5_ despite the low level of conservation at the residue level. This scenario is further supported by the facts that CheW is evolutionarily the most recent fold in the chemotaxis pathway [23] and that the CheW protein is highly dynamic [39]: both properties correlate with high evolvability and robustness–the molecule’s ability to evolve neutrally [40, 41].

### Interaction of CheV with chemoreceptors

Similarity of the CheV_W_ domain with CheW and CheA_P5_ suggests that CheV also interacts with chemoreceptors. In *Enterobacteriales*, chemoreceptors are the only genes of the chemotaxis pathway that are present as multiple homologs in a single genome. This may be a result of both ancestral and recent gene duplications as well as horizontal gene transfer. Therefore, in order to perform a meaningful phylogenetic profile analysis, it is necessary to classify all 644 chemoreceptor sequences in the analyzed enterobacterial pan-genome into orthologous groups.

### Chemoreceptors in the enterobacteria pan-genome belong to the same major length-class, but many different orthologous groups

**Fig 3:**
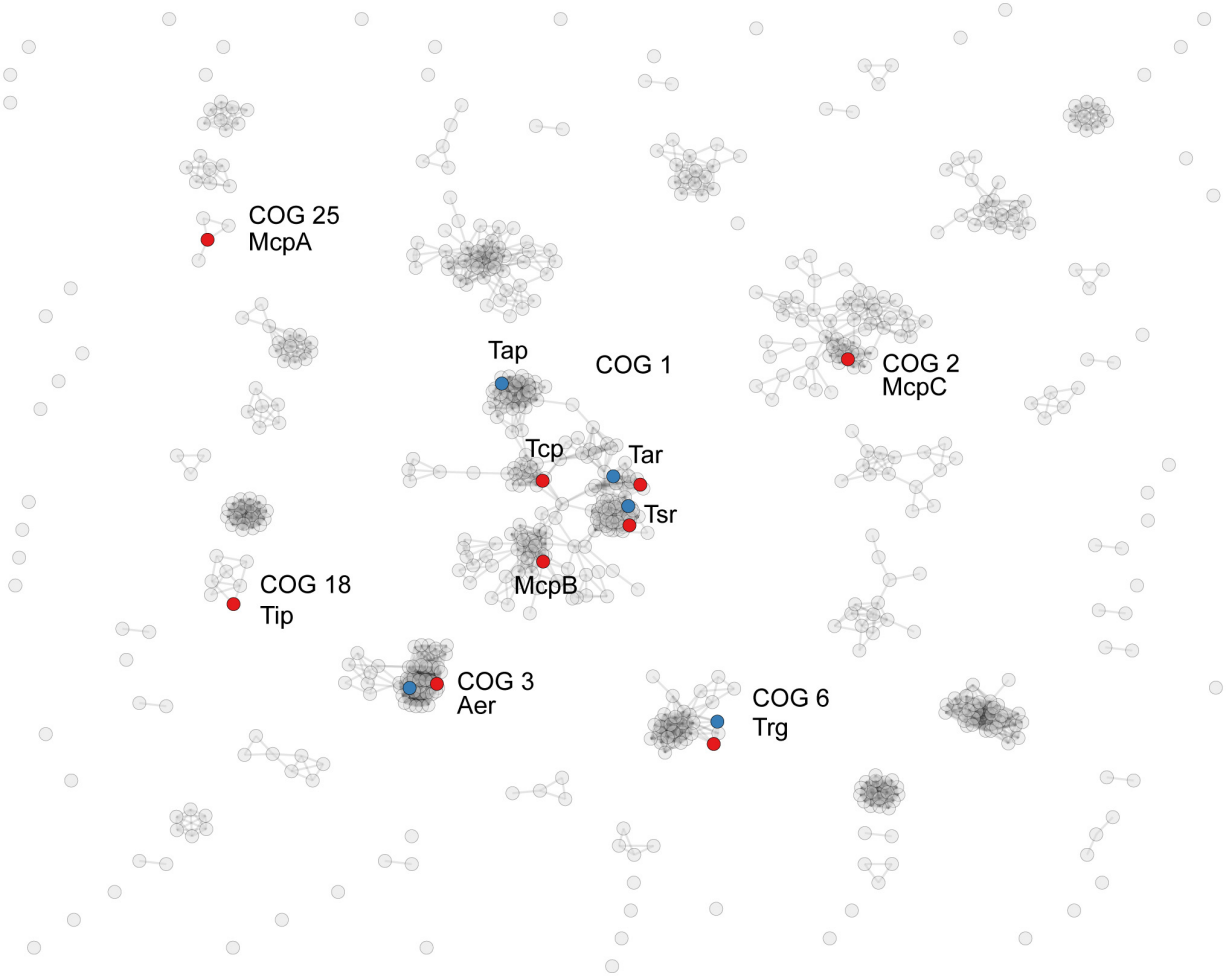
Clusters of orthologous groups of chemoreceptors from 43 enterobacterial genomes. Each node represents a chemoreceptor sequence. MCPs from *E*. *coli* (blue) and *S*. *enterica* (red) are labeled by name and a corresponding COG number. See S1 Dataset and Materials and Methods for details.

By matching all 644 chemoreceptor sequences in the non-redundant genome set to hidden Markov models designed for various length-classes of the chemoreceptor signaling domain [42] we determined that 599 chemoreceptor sequences belong to the 36H class (the signaling domain consists of 36 helical heptads) while 19 sequences belong to the 24H class (the signaling domain consists of 24 helical heptads) and 26 sequences remained unclassified. There was no correlation between the presence of CheV and chemoreceptors of a specific length-class. We then employed a principle of clusters of orthologous groups of proteins (COGs) [43] to obtain a higher resolution classification of chemoreceptors in enterobacteria (see Materials and Methods for details). Resulting chemoreceptor COGs in enterobacteria are visualized in Fig 3 and COG assignments of *E*. *coli* K12 and *S*. *enterica* LT2 chemoreceptors are specified in Table 1. The largest cluster of chemoreceptors (COG1) contains Tsr, Tar and Tap, whereas the other two *E*. *coli* chemoreceptors belong to separate groups: Trg in COG6 and Aer in COG3, which is consistent with recent phylogenetic studies [44]. The citrate sensor Tcp in *S*. *enterica* was found in COG1 (Fig 3, Table 1), which is also consistent with previous findings showing its relatedness to Tsr and Tar [45]. As a final result, all 644 chemoreceptor sequences in the pan-genome of analyzed enterobacteria were assigned to 99 GOGs that contained from 161 member sequences (COG1) to a single member sequence (COG44 to COG99) (S1 Dataset).

**Table 1:** COG assignment of chemoreceptors in *E*. *coli* and S. *enterica*.

Protein	Locus		COG number
	*E*. *coli*	*S*. *enterica*	
Tar	Y75_p1862	STM1919	1
Tsr	Y75_p4240	STM4533	1
Trg	Y75_p1397	STM1626	6
Tap	Y75_p1861		1
Aer	Y75_p2997	STM3217	3
Tcp		STM3577	1
Tip		STM1657	18
McpA		STM3138	25
McpB		STM3152	1
McpC		STM3216	2

### Phylogenetic profiling reveals co-evolution of CheV and a specific chemoreceptor COG

**Fig 4:**
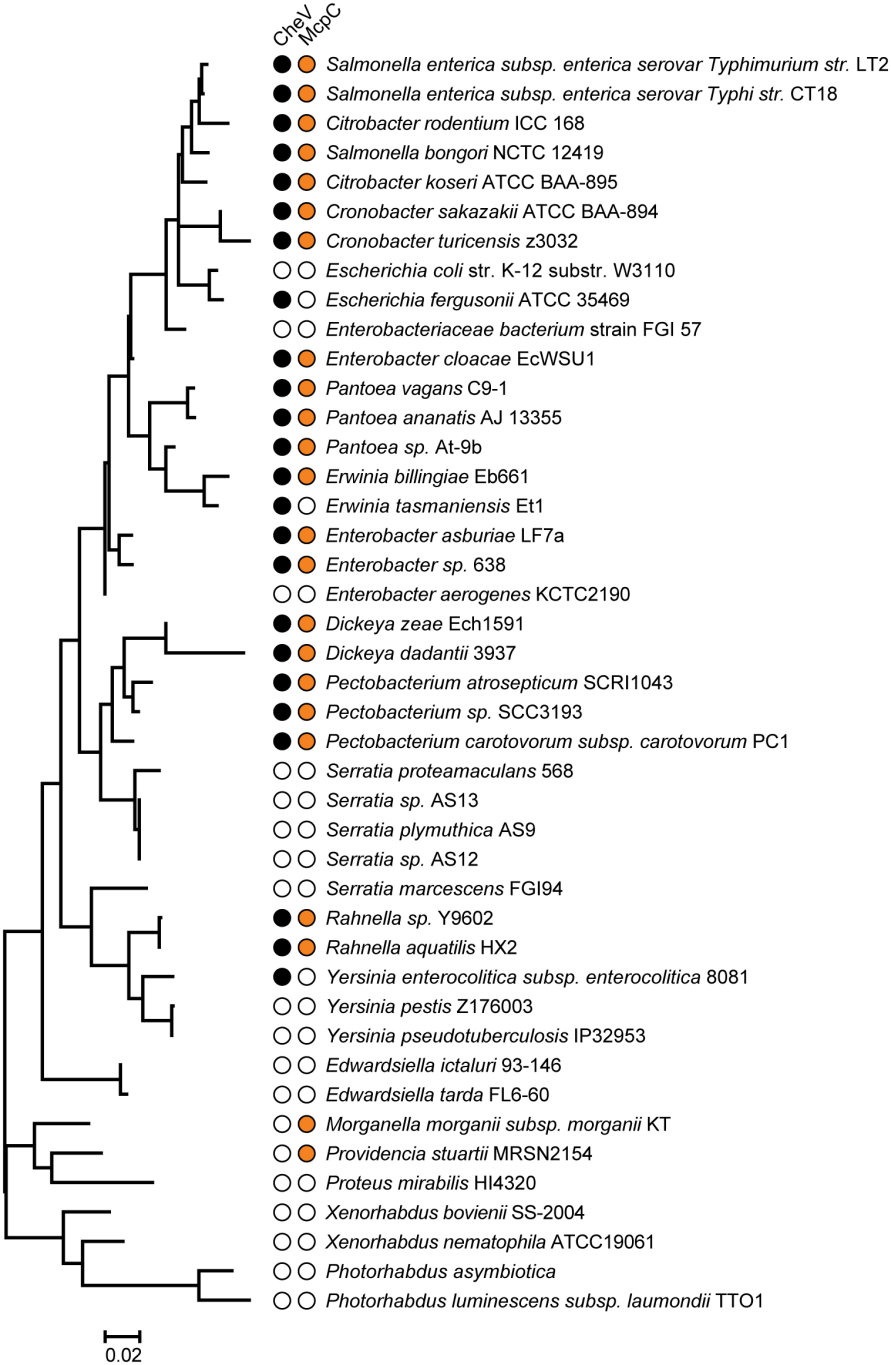
Co-Evolution of CheV and McpC orthologs. Phylogenetic profile shows the correlation of presence and absence of CheV (orange) and McpC orthologs (black). Left, 16S phylogenetic tree of the organisms used in this study.

We employed a principle of phylogenetic profiling to test a hypothesis that specific chemoreceptor COGs are linked to CheV. This method is based on the assumption that proteins that function together in a pathway or structural complex are likely to co-evolve [46]. We mapped instances of the presence and absence of CheV and all 99 chemoreceptor COGs onto the CheA phylogenetic tree in order to determine whether the presence of genes from any of the COGs correlate with presence of CheV in the genomes of *Enterobacteriales* (S8 Fig). As a result, we have found the strongest correlation (r = 0.77) between CheV and the second largest orthologous group–COG2, exemplified by the *S*. *enterica* McpC chemoreceptor (Fig 4), which suggests that chemoreceptors of COG2 need CheV to function optimally. We have further tested this hypothesis by using genomic context methods postulating that if two proteins interact, then in some genomes their genes can be fused or located adjacent to each other on the chromosome [47]. While we detected no fusion events between *cheV* and *mcp* genes in *Enterobacteriales*, the gene neighborhood analysis revealed that in two *Pantoea* genomes the *cheV* gene was adjacent to the *mcp* gene (locus tags Pat9b_0852/Pat_9b_0851 and Pvag_0292/Pvag_0291). Both *mcp* gene products belong to COG2 (S1 Dataset, S9 Fig), which further strengthens our hypothesis. No other cases of *cheV* and *mcp* gene neighborhood were found in the analyzed dataset.

We also mined a rich transcriptomic compendium for *S*. *enterica* serovar Typhimurium [48] in search for co-expression patterns between *cheV* and any of the *mcp* genes. We found no correlation between expression levels of a specific adaptor (CheW or CheV) and any MCP; however, interestingly, McpC appears to be a high-abundance chemoreceptor in *Salmonella*, similarly to Tar and Tsr (S2 Dataset).

If our hypothesis is correct, we expect that the COG2 group of receptors has unique features detectable as specific conservation patterns in chemoreceptor sequences from this group relative to other groups. Comparing chemoreceptors from COG2 and those from other COGs known to work with CheW might suggest which of these unique features are related to the interaction with CheV. We can assume with confidence that receptors from COG1 utilize CheW as an adaptor—*E*.*coli* has three out of five receptors from COG1 and does not have CheV. Thus, if COG1 chemoreceptors utilize CheW and not CheV, but COG2 chemoreceptors utilize CheV instead of or in addition to CheW, then COG1 and COG2 chemoreceptors should have group-specific conserved positions in their signaling domains responsible for the interaction with different adaptors.

### Differences in the signaling domains of chemoreceptors from COG1 and COG2

We constructed multiple sequence alignment of the signaling domains from COG1 and COG2 sequences, as well as from COG6 sequences (S9 Fig). We used COG6, the group containing the product of the *trg* gene from *E*. *coli* and *S*. *enterica*, as a control, because Trg is known to only utilize CheW and it has the same membrane topology as COG1 and COG2, in contrast to COG3 (exemplified by the *E*. *coli* Aer chemoreceptor), which is also known to interact with CheW but has a different membrane topology. In order to avoid evolutionary bias, we selected sequences only from organisms that have chemoreceptors from COG1, COG2 and COG6 as well as CheV proteins, (see Materials and Methods). Positions that are highly conserved (>90% identity) in COG1 and COG6, but differently highly conserved (>90% identity) in COG2 are likely to be important for the interaction between COG2 receptors and CheV.

**Fig 5:**
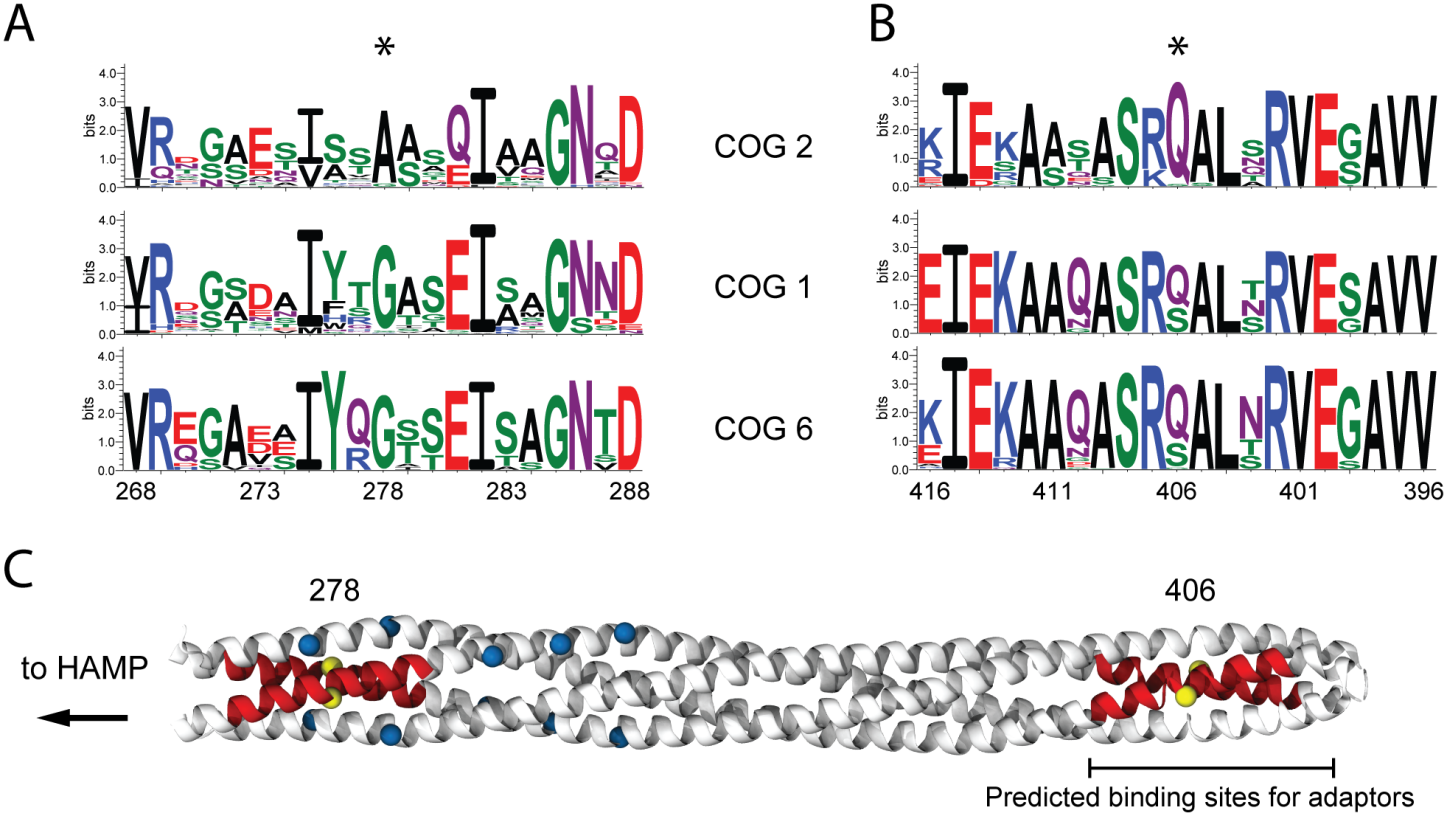
Changes in conservation patterns in chemoreceptors. Comparison of the sequence logo from sequences in COG1, COG2 and COG6 of the 20 amino-acid region around the Gly278 (A) and Ser406 (B), both marked with a star. The sequence is inverted in the B panel (right to left) to depict the difference in helix where the two positions are found. Gly278 is found in the descending helix and S406 is found in the ascending helix of the receptor. C) Cartoon representation based on the crystal structure (PDB code: 1QU7) [56] of the chemoreceptor signaling domain (white ribbons) and the methylation sites (blue spheres) with mapping of the 10 amino-acid region (red ribbons) around the two positions (yellow spheres) with significantly different pattern in sequences from COG2 compared to sequences from COG1 and COG6.

Surprisingly, there is only one position in the alignment that has the aforementioned characteristics: position 278 (numbers are given for the *E*. *coli* Tar chemoreceptor) is conserved in COG1 and COG6 as a glycine, and is also conserved in COG2 but as an alanine (Fig 5A, S2 Table). The position Gly278 lays away from the postulated adaptor binding site in the chemoreceptor structure: approximately from Asp365 to Leu415 [49, 50, 51] and is unlikely to be the CheV binding site on the chemoreceptor. Interestingly, this position has been a target of intense mutagenesis and is known to dramatically increase the kinase activity upon mutation to any other amino acid. In fact, mutations at the Gly278 site, including the alanine substitution, show the highest activation of the kinase in *E*.*coli*/*Samonella* chemotaxis system to date [52]. In addition, our recent molecular dynamic simulation study showed Gly278 as the site of the chemoreceptor with highest propensity for bending [53]. The bending mechanism of the chemoreceptor has been proposed to influence and even control the kinase activity in several studies [54, 55]. Thus, we predict that McpC and other chemoreceptors from COG2 that have Ala instead of Gly in position 278 tend to naturally increase the level of kinase activity in comparison to other chemoreceptors.

Within the proposed adaptor binding region, which shows overall extreme conservation not only among enterobacteria, but across prokaryotes [42], only one position, 406, has a unique type of distribution–conserved glutamine in COG2 and a glutamine/serine mix in COG1 and COG6 (Fig 5B)–which contrast to the norm that overall, COG6 is more conserved than COG1, which is more conserved than COG2. It is striking that among 50 amino acid positions in this highly evolutionarily constrained region, 49 positions had higher information content in COG1 and only 1 position had higher information content in COG2 (S10 Fig). We hypothesize that having a serine in the position 406 might increase the binding affinity between CheV_W_ and the chemoreceptor. This single difference among the highly conserved region of protein interaction suggests that CheV_W_ must have a mix of highly conserved residues in common with CheW protein and some that must be different and yet conserved among CheV proteins in the vicinity of the adaptor binding region for chemoreceptors due to some specificity towards receptors from COG2.

### Interactions between the adaptor domain of CheV and chemoreceptors

**Fig 6:**
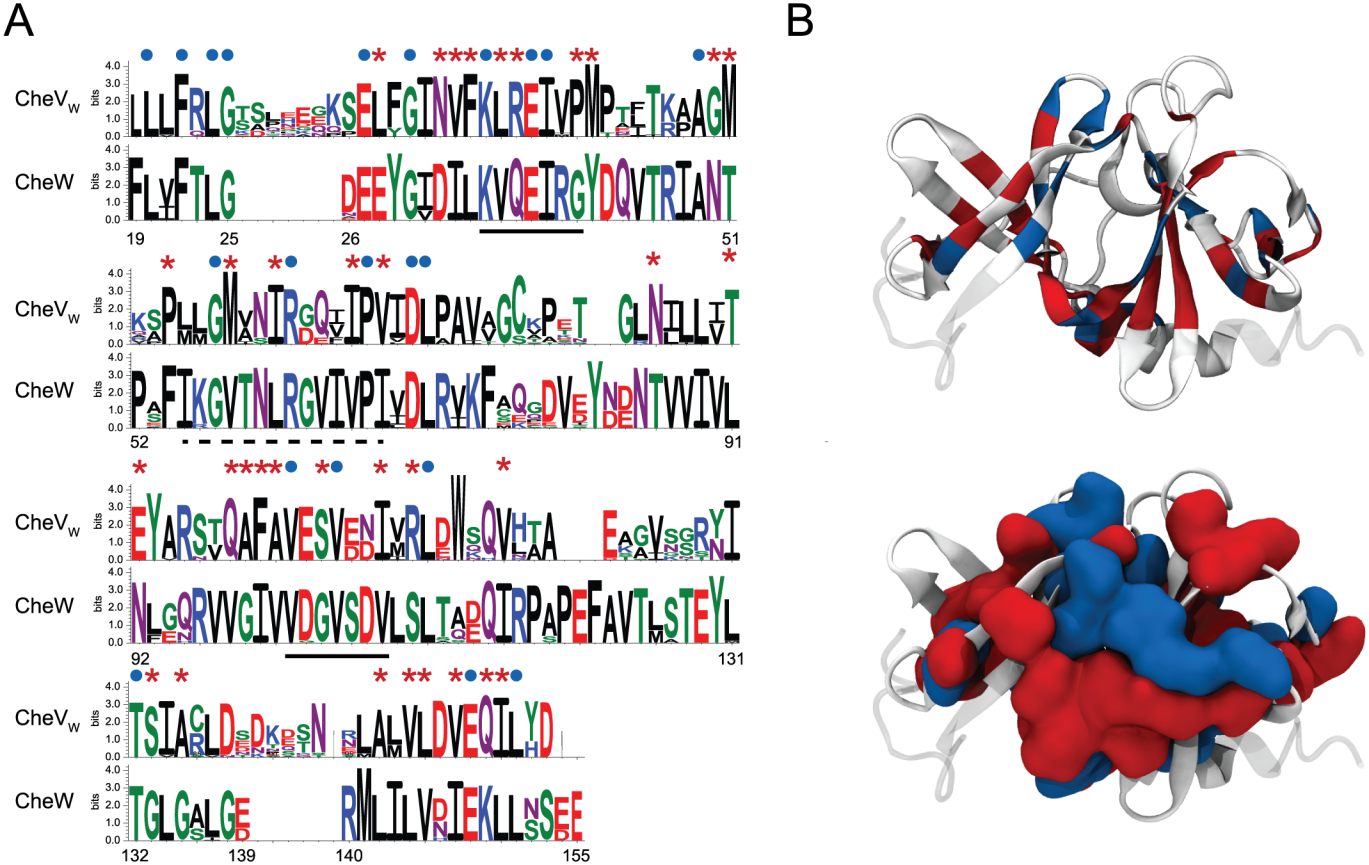
Analysis of the sequence conservation between CheW and CheV_W_. A) Sequence logo of CheV_W_ (top logo) and CheW (bottom logo). Positions conserved in both groups (20, 22, 24, 25, 27, 30, 35, 38, 39, 49, 57, 62, 67, 70, 71, 102, 105, 111, 132, 148, 151) (blue circles) and position conserved within each groups (28, 32, 33, 34, 36, 37, 41, 42, 50, 51, 54, 58, 61, 66, 68, 86, 89, 91, 92,98, 99, 100, 101, 104, 108, 110, 116, 133, 135, 142, 144, 145, 147, 149, 150) (red stars) are highlighted. Numbers for *E*. *coli* CheW. Proposed CheW regions for binding CheA [24, 58] and chemoreceptors [24, 50] are underlined in dashed and solid lines, correspondingly. B) Mapping of marked positions onto *E*. *coli* CheW NMR model [57] in ribbons (top) and accessible surface area (bottom).

We aligned sequences of CheW proteins and CheV_W_ domains from the non-redundant set of *Enterobacteriales* genomes (S11 Fig). Only sequences from organisms with CheV and CheW genes were selected to build sequence logos used to identify conservation patterns between these two groups (Fig 6A). We then mapped positions that are 100% conserved between and within CheW and CheV_W_ sequences onto the CheW NMR model (PDB code: 2HO9) [57](Fig 6B). Both types of residues are located in the solvent exposed central groove between the two β-barrel subdomains, which has been implicated in the interaction of CheW with chemoreceptors [24, 50, 58]. Residues forming the Arg62-Glu38 salt bridge, which was suggested to maintain a specific geometry between chemoreceptor and kinase binding sites on CheW [39], were universally conserved in CheW and CheV_W_ (Fig 6). These results suggest that the predicted chemoreceptor interaction region of the adaptor structure is conserved in both CheW and CheV_W_ domains and contains a set of residues conserved in both adaptors and a set of residues uniquely conserved in each adaptor family. This is line with the previous findings [22, 26] and supports the hypothesis that CheW and CheV_W_ share the same binding spot on chemoreceptors, but have some affinity bias towards chemoreceptors from different orthologous groups.

### CheV as an alternative signal termination mechanism

It is known that mixed teams of chemoreceptors come together to form a single cluster in organisms with a single chemotaxis array [59]. Based on our findings we suggest that CheV is necessary to accommodate chemoreceptors from COG2 in the chemotaxis array. Because of the uniquely conserved alanine in the position 278 in COG2 chemoreceptors, we propose that as these receptors are incorporated into the chemotaxis protein cluster, the base level of kinase activity increases, because position 278 in these receptors is occupied exclusively by alanine (a change from a uniformly conserved glycine to alanine in this position in COG1 chemoreceptors elevates the kinase activity). As previously shown, the presence of CheV in other chemotaxis systems influences the levels of phosphorylated CheY (CheY-P) [22] and our results suggest that in enterobacteria, CheV_RR_ specifically interacts with CheA_P1,_ a known CheY-interacting domain. Thus, we propose that CheV might work as a phosphate sink [60] “stealing” the extra phosphor groups from CheA_P1_ (resulting from over-stimulation of the kinase by COG2 chemoreceptors) before they can reach CheY and consequently normalizing the overall CheY-P concentration downstream of the system. Interestingly, based on experimental evidence the role of a phosphate sink for CheV was previously suggested in *H*. *pylori* [61] and mentioned as a possibility in *B*. *subtilis* [20].

**Fig 7:**
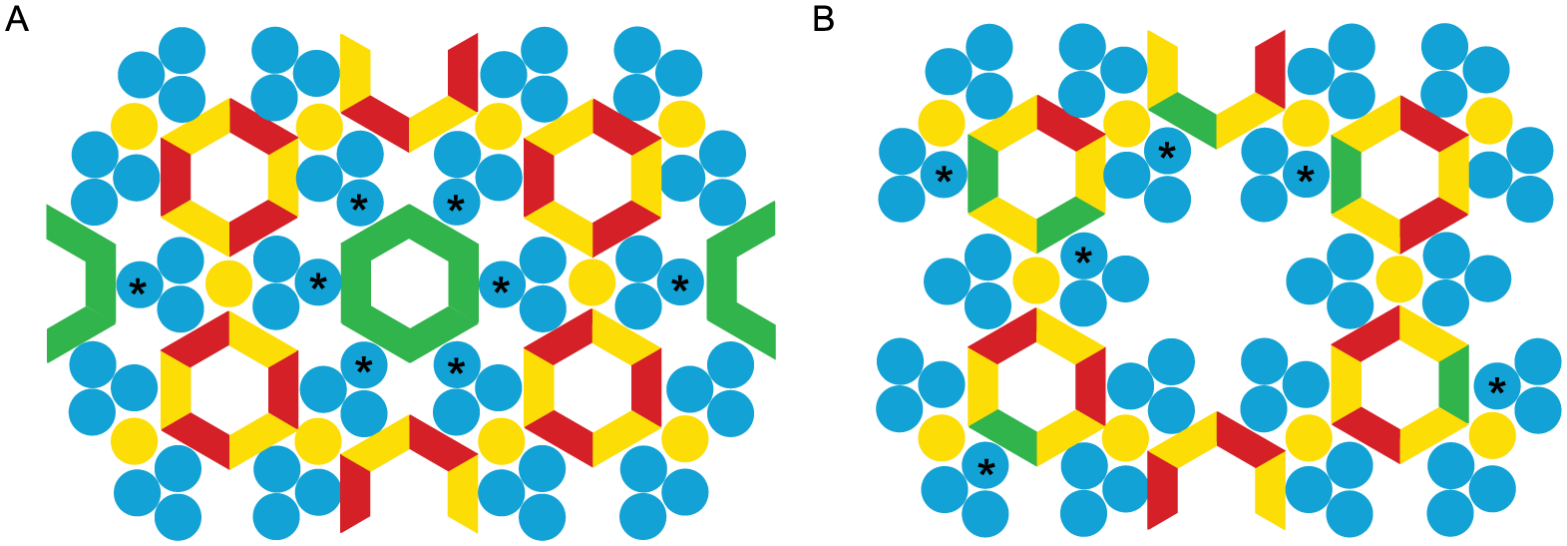
Schematic models of possible integration of CheV into the chemoreceptor array. Top-view of the arrangement of the array components showing the known and proposed interaction sites between chemoreceptor trimers (blue), CheA (yellow) and CheW (red) [36, 37], as well as potential locations of CheV (green). Chemoreceptors that interact with CheV are marked with asterisks. A) CheV occupies the proposed empty ring and does not interact with CheA_P5_ or CheW. B) CheV might be incorporated with CheW and CheA_P5_ into the hexagonal ring.

In order for this mechanism to work, we anticipate that precise positioning of CheV relative to CheA and CheW might not be essential given the stochastic nature of the chemotaxis system and that only the overall concentration of CheY-P needs to be controlled. Our lack of support for a hypothetical CheV_W_−CheW/CheA_P5_ interaction appears to be in contrast with our findings strongly suggesting that CheV interacts with chemoreceptors in the same binding region as CheW and CheA_P5_. However, the latest model for chemotaxis array assembly predicts an “empty” chemoreceptor hexagonal ring neighboring a CheA-CheW filled hexagonal ring with three kinases and three CheWs [36, 37]. In line with this model and our findings, we propose two competing models that differ solely on whether the CheV_W_−CheW/CheA_P5_ interaction takes place or not. We propose that CheV is incorporated in the chemotaxis array, by either (i) fully occupying one of the “empty” rings (Fig 7A) or (ii) mixing with the hexagonal ring made of CheW and CheA_P5_ (Fig 7B). In fact, the conservation of position 406 in COG2 chemoreceptors suggests that this position might determine whether the chemoreceptor will be facing the kinase/CheW or CheV. Clearly, only experimental verification can provide support for or against this hypothesis and help distinguishing between the two competing models for CheV positioning with the signaling array.

In summary, we tested a hypothesis that in enterobacteria CheV functions as an additional adaptor linking the CheA kinase to certain types of chemoreceptors that cannot be effectively accommodated by the universal adaptor CheW. Phylogenetic profiling, genomic context and comparative protein sequence analyses suggested that CheV interacts with chemoreceptors from an orthologous group COG2 exemplified by the *Salmonella* McpC protein. The biological function for CheV proposed here should be taken with caution when extrapolated to organisms outside enterobacteria. The chemotaxis system of F7 class (classification according to [23]) in enterobacteria differs dramatically from the F1 system in *B*. *subtilis* or the F3 system in *H*. *pylori*, both are model organisms to study CheV [20–22, 26, 61]. While we observed the direct relationship between the large number of chemoreceptors and the presence of CheV in enterobacteria, outliers are present both in and outside this group of organisms, For example, the model organism *H*. *pylori* has only four chemoreceptors and three CheV proteins [26]. Nevertheless, while the model for CheV interaction with the signaling array proposed here might not be generally applicable to other systems, the postulate that an additional adaptor, such as CheV, is necessary to incorporate certain types of chemoreceptors into the signaling array is likely to be broadly relevant.

## Materials and Methods

### Data sources and bioinformatics software

The primary source of data in this study is the MiST2.2 database [33] including pre-computed domain counts, classification of chemotaxis genes, protein and ribosomal 16S sequences. CheA and CheV proteins were assigned to chemotaxis classes [23] using previously described hidden Markov models [62] and the HMMER v3.0 software package [63]. Chemoreceptors were assigned to heptad classes using previously described hidden Markov models [42] using HMMER v2.0 [64]. Sequence alignments were built using L-INSI-I algorithm from MAFFT v6.864b package [65]. Phylogenetic trees were constructed using PhyML v3.0 [66]. Figures and calculations were produced by custom made scripts using ggplot2 [67] package for R language and NetworkX v1.8.1[68] and Numpy [69] modules for Python. Information content logos were built using Weblogo 3.0 [70].

### Phylogenetics

Maximum likelihood phylogenetic trees of protein sequences were built using PhyML with the following options: JTT model, empirical amino acid frequencies, 4 substitution categories, estimated gamma distribution parameter and subtree pruning and regrafting (SPR) topology search. Maximum likelihood phylogenetic tree of the ribosomal 16S DNA sequences was built using PhyML with the following options: GTR model, 20 substitution categories, estimated gamma distribution parameter and subtree pruning and regrafting (SPR) topology search.

### Genomic context analyses

Potential gene fusion events and gene neighborhoods of *cheV* genes were visualized and analyzed using the MiST database [33]. Expression data for chemotaxis genes was compiled from the *Salmonella* gene expression compendium [48].

### MCP COG construction and visualization

To obtain clusters of orthologous groups of MCPs, all chemoreceptor sequences were compared to each other using all-versus-all BLAST [71]. Two sequences were merged into a cluster if the E-value of the reciprocal best BLAST hit was below selected threshold of 10E-30 with 95% length coverage. Any given sequence with a reciprocal best BLAST hit to a sequence from a cluster became a member of this cluster. If a sequence had BLAST hits to sequences from two clusters, the clusters were merged. In a graphical representation of clustering, each cluster (COG) is represented independently of each other using the algorithm Neato from the NetworkX module for Python, where distances between nodes (sequences) are calculated based on connectivity within the cluster (number of reciprocal best BLAST hits with the other members of the cluster). The edges connecting the nodes are all equivalent, reflecting the binary (reciprocal best BLAST hit or not) nature of the graph. Thus, nodes with high connectivity are central while nodes with less connectivity tend to be placed in peripheral regions of the graph.

#### Position specific amino-acid content analysis per COG

In order to compare the sequences from COG1, COG2 and COG6 in organisms with CheV, we selected 178 sequences from COG1(101 sequences), COG2 (51 sequence) and COG6 (26 sequences) from the total of 246 sequences present in COG1 (161 sequence), COG2 (55 sequences) and COG6 (30 sequences). In addition, to avoid redundancy, we applied a 90% identity filter and the final dataset contained 126 sequences from COG1 (73 sequences), COG2 (37 sequences) and COG6 (16 sequences).

## Supporting Information

S1 Table Number of chemotaxis genes in *Enterobacteriales* genomes. Genomes comprising a non-redundant set used for comparative analysis (43 in total) are shown in bold.
(PDF)

S2 Table Highly conserved positions that distinguish COG1 chemoreceptors from COG2 chemoreceptors. Positions that are 100% conserved within each group and highly conserved group-specific positions are shown. Residue numbers indicated positions in the multiple sequence alignment. Position 280 corresponds to Gly278 in the *E*. *coli* Tar chemoreceptor.
(PDF)

S1 Fig Vertical evolution of CheA in *Enterobacteriales*. Maximum likelihood phylogenetic trees of 16S ribosomal RNA and CheA proteins have nearly identical topology. Each sequence tag contains the first two letters of the genus, the first three letters of the species and the organism id in the MIST database, followed by the locus (for CheA) and accession number. The tag also includes the chemotaxis class for CheA (e.g. F7).
(PDF)

S2 Fig Vertical evolution of CheV in *Enterobacteriales*. Comparison of the CheA and CheV phylogenetic trees suggests vertical evolution of CheV and supports the hypothesis that CheV was present in the common ancestor of *Enterobacteriales*. Each sequence tag contains the first two letters of the genus, the first three letters of the species and the organism id in the MIST database, followed by the locus and accession number. The tag also includes the chemotaxis class (e.g. F7).
(PDF)

S3 Fig Multiple sequence alignment of CheA_P2_ sequences from the non-redundant *Enterobacteriales* genome set. Each sequence tag contains the first two letters of the genus, the first three letters of the species and the organism id in the MiST database, followed by the locus and accession number. The tag also includes the chemotaxis class for CheA (e.g. F7) and shows the presence (1CheV) or absence (0CheV) of *cheV* genes in a corresponding genome.
(PDF)

S4 Fig Conservation patterns in the CheA_P2_ domain in organisms with CheV and without CheV. Sequence logos were generated from the multiple sequence alignment shown in S3 Fig
(PDF)

S5 Fig Multiple sequence alignment of CheA_P1_ sequences from the non-redundant *Enterobacteriales* genome set. Each sequence tag contains the first two letters of the genus, the first three letters of the species and the organism id in the MIST database, followed by the locus and accession number. The tag also includes the chemotaxis class for CheA (e.g. F7) and shows the presence (1CheV) or absence (0CheV) of *cheV* genes in a corresponding genome.
(PDF)

S6 Fig Multiple sequence alignment of CheY and CheV_RR_ sequences from the non-redundant *Enterobacteriales* genome set. Each sequence tag contains the first two letters of the genus, the first three letters of the species and the organism id in the MIST database, followed by the locus and accession number. The tag also includes the chemotaxis class for CheV (e.g. F7) and shows the presence (1CheV) or absence (0CheV) of *cheV* genes in a corresponding genome.
(PDF)

S7 Fig The P5 domain sequence conservation in CheA (A) and CheW (B) from genomes with CheV and without CheV. (PDF)

S8 Fig Complete phylogenetic profile of COGs of MCPs in comparison with CheW in genomes from Enterobacteriaceae. The organism list is ordered by the ribosomal 16S tree (left). The COGs of MCPs are ordered by the largest COG, containing the highest number of genes, to the smallest COG from left to right (Right panel). The color code represents the number of genes from a specific COG present in a given genome.
(PDF)

S9 Fig Multiple sequence alignment of chemoreceptors from COG1, COG2 and COG6. Each sequence tag contains the first two letters of the genus, the first three letters of the species and the organism id in the MiST database, followed by the locus and accession number. The tag also includes the heptad class (e.g. 36H) and the COG to which a given sequence belongs.
(PDF)

S10 Fig Information content in the putative adaptor binding region of chemoreceptor sequences from COG1 (blue) and COG2 (red). Positions are numbered as in *E*. *coli* Tar protein. Ser406 (marked with the star) is the only position in this region, which is more conserved in COG2 than COG1.
(PDF)

S11 Fig Multiple sequence alignment of CheW proteins and CheV-W domains from the non-redundant *Enterobacteriales* genome set. Each sequence tag contains the first two letters of the genus, the first three letters of the species and the organism id in the MiST database, followed by the locus and accession number. The tag also includes the chemotaxis class for CheV (e.g. F7)
(PDF)

S1 Dataset Chemoreceptor COG assignment for 43 Enterobacteriales genomes. (PDF)

S2 Dataset Heatmaps for chemotaxis gene expression in *Salmonella enterica* serovar Typhimurium under different growth conditions (data from ref. [48]). (XLSX)
