# Supplementary material for: Evolutionary Genomics Suggests That CheV Is an Additional Adaptor for Accommodating Specific Chemoreceptors within the Chemotaxis Signaling Complex
Source: PLoS Comput Biol. 2016 Feb 4;12(2):e1004723. doi: 10.1371/journal.pcbi.1004723 (PMC4742279; doi:10.1371/journal.pcbi.1004723)
Supplement: S1 Table — Genomes comprising a non-redundant set used for comparative analysis (43 in total) are shown in bold. (PDF) [file pcbi.1004723.s001.pdf]

S1 Table. Number of chemotaxis genes in *Enterobacteriales* genomes. Genomes comprising a non-redundant set used for comparative analysis (43 in total) are shown in bold.

Genus	Species	Strain	Chemotaxis proteins (number of genes)								
			MCP	CheA	CheB	CheCX	CheD	CheR	CheV	CheZ	CheW
aphid	secondary	symbionts secondary endosymbiont of Ctenarytaina eucalypti	0	0	0	0	0	0	0	0	0
aphid	secondary	symbionts secondary endosymbiont of Heteropsylla cubana	0	0	0	0	0	0	0	0	0
Buchnera	aphidicola	(Cinara tujafilina)	0	0	0	0	0	0	0	0	0
Buchnera	aphidicola	BCc	0	0	0	0	0	0	0	0	0
Buchnera	aphidicola	str. 5A (Acyrthosiphon pisum)	0	0	0	0	0	0	0	0	0
Buchnera	aphidicola	str. Ak (Acyrthosiphon kondoi)	0	0	0	0	0	0	0	0	0
Buchnera	aphidicola	str. APS (Acyrthosiphon pisum)	0	0	0	0	0	0	0	0	0
Buchnera	aphidicola	str. Bp (Baizongia pistaciae)	0	0	0	0	0	0	0	0	0
Buchnera	aphidicola	str. JF98 (Acyrthosiphon pisum)	0	0	0	0	0	0	0	0	0
Buchnera	aphidicola	str. JF99 (Acyrthosiphon pisum)	0	0	0	0	0	0	0	0	0
Buchnera	aphidicola	str. LL01 (Acyrthosiphon pisum)	0	0	0	0	0	0	0	0	0
Buchnera	aphidicola	str. Sg (Schizaphis graminum)	0	0	0	0	0	0	0	0	0
Buchnera	aphidicola	str. TLW03 (Acyrthosiphon pisum)	0	0	0	0	0	0	0	0	0
Buchnera	aphidicola	str. Tuc7 (Acyrthosiphon pisum)	0	0	0	0	0	0	0	0	0
Buchnera	aphidicola	str. Ua (Uroleucon ambrosiae)	0	0	0	0	0	0	0	0	0
Candidatus	Blochmannia	chromaiodes str. 640	0	0	0	0	0	0	0	0	0
Candidatus	Blochmannia	floridanus	0	0	0	0	0	0	0	0	0
Candidatus	Blochmannia	pennsylvanicus str. BPEN	0	0	0	0	0	0	0	0	0
Candidatus	Blochmannia	vafer str. BVAF	0	0	0	0	0	0	0	0	0
Candidatus	Hamiltonella	defensa 5AT (Acyrthosiphon pisum)	0	0	0	0	0	0	0	0	0
Candidatus	Moranella	endobia PCIT	0	0	0	0	0	0	0	0	0
Candidatus	Moranella	endobia PCVAL	0	0	0	0	0	0	0	0	0
Candidatus	Riesia	pediculicola USDA	0	0	0	0	0	0	0	0	0
**Citrobacter**	**koseri**	**ATCC BAA-895**	**14**	**1**	**1**	**0**	**0**	**1**	**1**	**1**	**1**
**Citrobacter**	**rodentium**	**ICC168**	**7**	**1**	**1**	**0**	**0**	**1**	**1**	**1**	**1**
**Cronobacter**	**sakazakii**	**ATCC BAA-894**	**26**	**1**	**1**	**0**	**0**	**1**	**1**	**1**	**1**
Cronobacter	sakazakii	ES15	23	1	1	0	0	1	1	1	1
Cronobacter	sakazakii	SP291	26	1	1	0	0	1	1	1	1
**Cronobacter**	**turicensis**	**z3032**	**28**	**1**	**1**	**0**	**0**	**1**	**1**	**1**	**1**
**Dickeya**	**dadantii**	**3937**	**47**	**1**	**1**	**0**	**0**	**1**	**1**	**1**	**1**
Dickeya	dadantii	Ech586	48	1	1	0	0	1	1	1	1
Dickeya	dadantii	Ech703	42	1	1	0	0	1	1	1	1
**Dickeya**	**zeae**	**Ech1591**	**42**	**1**	**1**	**0**	**0**	**1**	**1**	**1**	**1**
**Edwardsiella**	**ictaluri**	**93-146**	**9**	**1**	**1**	**0**	**0**	**1**	**0**	**1**	**1**
Edwardsiella	tarda	C07-087	8	1	1	0	0	1	0	1	1
Edwardsiella	tarda	EIB202	8	1	1	0	0	1	0	1	1
**Edwardsiella**	**tarda**	**FL6-60**	**8**	**1**	**1**	**0**	**0**	**1**	**0**	**1**	**1**
Enterobacter	aerogenes	EA1509E	2	1	1	0	0	1	0	1	1
**Enterobacter**	**aerogenes**	**KCTC 2190**	**2**	**1**	**1**	**0**	**0**	**1**	**0**	**1**	**1**
**Enterobacter**	**asburiae**	**LF7a**	**20**	**1**	**1**	**0**	**0**	**1**	**1**	**1**	**1**
**Enterobacter**	**cloacae**	**EcWSU1**	**17**	**1**	**1**	**0**	**0**	**1**	**1**	**1**	**1**
Enterobacter	cloacae	SCF1	14	1	1	0	0	1	1	1	1
Enterobacter	cloacae	subsp. cloacae ATCC 13047	16	2	2	0	0	2	1	2	2
Enterobacter	cloacae	subsp. cloacae ENHKU01	16	1	1	0	0	1	1	1	1
Enterobacter	cloacae	subsp. cloacae NCTC 9394	9	2	1	0	0	2	1	2	2
Enterobacter	cloacae	subsp. dissolvens SDM	16	2	2	0	0	2	1	2	2
**Enterobacter**	**sp.**	**638**	**18**	**1**	**1**	**0**	**0**	**1**	**1**	**1**	**1**
**Enterobacteriaceae**	**bacterium**	**strain FGI 57**	**6**	**1**	**1**	**0**	**0**	**1**	**0**	**1**	**1**
Erwinia	amylovora	ATCC 49946	11	2	2	0	0	2	1	2	2
Erwinia	amylovora	CFBP1430	11	2	2	0	0	2	1	2	2
**Erwinia**	**billingiae**	**Eb661**	**29**	**1**	**1**	**0**	**0**	**1**	**1**	**1**	**1**
Erwinia	pyrifoliae	DSM 12163	13	2	2	0	0	2	1	2	2
Erwinia	pyrifoliae	Ep1/96	13	2	2	0	0	2	1	2	2
Erwinia	sp.	Ejp617	13	2	2	0	0	2	1	2	2
**Erwinia**	**tasmaniensis**	**Et1/99**	**13**	**1**	**1**	**0**	**0**	**1**	**1**	**1**	**1**
Escherichia	blattae	DSM 4481	0	0	0	0	0	0	0	0	0
Escherichia	coli	42	5	1	1	0	0	1	0	1	1
Escherichia	coli	536	3	1	1	0	0	1	0	1	1
Escherichia	coli	55989	5	1	1	0	0	1	0	1	1
Escherichia	coli	ABU 83972	4	1	1	0	0	1	0	1	1
Escherichia	coli	APEC O1	4	1	1	0	0	1	0	1	1
Escherichia	coli	APEC O78	5	1	1	0	0	1	0	1	1
Escherichia	coli	ATCC 8739	5	1	1	0	0	1	0	1	1
Escherichia	coli	B str. REL606	5	1	1	0	0	1	0	1	1
Escherichia	coli	BL21(DE3)	5	1	1	0	0	1	0	1	1
Escherichia	coli	BL21(DE3)	5	1	1	0	0	1	0	1	1
Escherichia	coli	'BL21-Gold(DE3)pLysS AG'	5	1	1	0	0	1	0	1	1
Escherichia	coli	BW2952	5	1	1	0	0	1	0	1	1
Escherichia	coli	CFT073	4	1	1	0	0	1	0	1	1
Escherichia	coli	DH1	5	1	1	0	0	1	0	1	1

Escherichia	coli	DH1	5	1	1	0	0	1	0	1	1
Escherichia	coli	E24377A	5	1	1	0	0	1	0	1	1
Escherichia	coli	ED1a	3	1	1	0	0	1	0	1	1
Escherichia	coli	ETEC H10407	5	1	1	0	0	1	0	1	1
Escherichia	coli	HS	5	1	1	0	0	1	0	1	1
Escherichia	coli	IAI1	5	1	1	0	0	1	0	1	1
Escherichia	coli	IAI39	3	1	1	0	0	1	0	1	1
Escherichia	coli	IHE3034	4	1	1	0	0	1	0	1	1
Escherichia	coli	KO11FL	5	1	1	0	0	1	0	1	1
Escherichia	coli	KO11FL	5	1	1	0	0	1	0	1	1
Escherichia	coli	LF82	3	1	1	0	0	1	0	1	1
Escherichia	coli	NA114	4	1	1	0	0	1	0	1	1
Escherichia	coli	O103:H2 str. 12009	4	1	1	0	0	1	0	1	1
Escherichia	coli	O104:H4 str. 2009EL-2050	5	1	1	0	0	1	0	1	1
Escherichia	coli	O104:H4 str. 2009EL-2071	5	1	1	0	0	1	0	1	1
Escherichia	coli	O104:H4 str. 2011C-3493	5	1	1	0	0	1	0	1	1
Escherichia	coli	O111:H- str. 11128	5	1	1	0	0	1	0	1	1
Escherichia	coli	O127:H6 str. E2348/69	3	1	1	0	0	1	0	1	1
Escherichia	coli	O157:H7 str. EC4115	5	1	1	0	0	1	0	1	1
Escherichia	coli	O157:H7 str. EDL933	5	1	1	0	0	1	0	1	1
Escherichia	coli	O157:H7 str. Sakai	5	1	1	0	0	1	0	1	1
Escherichia	coli	O157:H7 str. TW14359	5	1	1	0	0	1	0	1	1
Escherichia	coli	O26:H11 str. 11368	5	1	1	0	0	1	0	1	1
Escherichia	coli	O55:H7 str. CB9615	5	1	1	0	0	1	0	1	1
Escherichia	coli	O55:H7 str. RM12579	4	1	1	0	0	1	0	1	1
Escherichia	coli	O7:K1 str. CE10	4	1	1	0	0	1	0	1	1
Escherichia	coli	O83:H1 str. NRG 857C	4	1	1	0	0	1	0	1	1
Escherichia	coli	P12b	4	1	1	0	0	1	0	1	1
Escherichia	coli	S88	3	1	1	0	0	1	0	1	1
Escherichia	coli	SE11	5	1	1	0	0	1	0	1	1
Escherichia	coli	SE15	4	1	1	0	0	1	0	1	1
Escherichia	coli	SMS-3-5	5	1	1	0	0	1	0	1	1
Escherichia	coli	str. 'clone D i14'	4	1	1	0	0	1	0	1	1
Escherichia	coli	str. 'clone D i2'	4	1	1	0	0	1	0	1	1
Escherichia	coli	str. K-12 substr. DH10B	4	0	1	0	0	1	0	1	1
Escherichia	coli	str. K-12 substr. MDS42	2	0	0	0	0	0	0	0	0
Escherichia	coli	str. K-12 substr. MG1655	5	1	1	0	0	1	0	1	1
**Escherichia**	**coli**	**str. K-12 substr. W3110**	**5**	**1**	**1**	**0**	**0**	**1**	**0**	**1**	**1**
Escherichia	coli	UM146	4	1	1	0	0	1	0	1	1
Escherichia	coli	UMN026	5	1	1	0	0	1	0	1	1
Escherichia	coli	UMNK88	5	1	1	0	0	1	0	1	1
Escherichia	coli	UTI89	4	1	1	0	0	1	0	1	1
Escherichia	coli	W	5	1	1	0	0	1	0	1	1
Escherichia	coli	W	5	1	1	0	0	1	0	1	1
Escherichia	coli	Xuzhou21	5	1	1	0	0	1	0	1	1
**Escherichia**	**fergusonii**	**ATCC 35469**	**3**	**1**	**1**	**0**	**0**	**1**	**1**	**1**	**1**
Klebsiella	oxytoca	E718	0	0	0	0	0	0	0	0	0
Klebsiella	oxytoca	KCTC 1686	0	0	0	0	0	0	0	0	0
Klebsiella	pneumoniae	342	0	0	0	0	0	0	0	0	0
Klebsiella	pneumoniae	KCTC 2242	0	0	0	0	0	0	0	0	0
Klebsiella	pneumoniae	subsp. pneumoniae 1084	0	0	0	0	0	0	0	0	0
Klebsiella	pneumoniae	subsp. pneumoniae HS11286	2	0	0	0	0	0	0	0	0
Klebsiella	pneumoniae	subsp. pneumoniae MGH 78578	0	0	0	0	0	0	0	0	0
Klebsiella	pneumoniae	subsp. pneumoniae NTUH-K2044	0	0	0	0	0	0	0	0	0
Klebsiella	variicola	At-22	0	0	0	0	0	0	0	0	0
**Morganella**	**morganii**	**subsp. morganii KT**	**9**	**1**	**1**	**0**	**0**	**1**	**0**	**1**	**1**
**Pantoea**	**ananatis**	**AJ13355**	**42**	**1**	**1**	**0**	**0**	**1**	**1**	**1**	**1**
Pantoea	ananatis	LMG 20103	42	1	1	0	0	1	1	1	1
Pantoea	ananatis	LMG 5342	45	1	1	0	0	1	1	1	1
Pantoea	ananatis	PA13	42	1	1	0	0	1	1	1	1
**Pantoea**	**sp.**	**At-9b**	**20**	**1**	**1**	**0**	**0**	**1**	**1**	**1**	**1**
**Pantoea**	**vagans**	**C9-1**	**29**	**1**	**1**	**0**	**0**	**1**	**1**	**2**	**1**
**Pectobacterium**	**atrosepticum**	**SCRI1043**	**36**	**1**	**1**	**0**	**0**	**1**	**1**	**1**	**1**
**Pectobacterium**	**carotovorum**	**subsp. carotovorum PC1**	**37**	**1**	**1**	**0**	**0**	**1**	**1**	**1**	**1**
Pectobacterium	carotovorum	subsp. carotovorum PCC21	37	1	1	0	0	1	1	1	1
**Pectobacterium**	**sp.**	**SCC3193**	**38**	**1**	**1**	**0**	**0**	**1**	**1**	**1**	**1**
Pectobacterium	wasabiae	WPP163	38	1	1	0	0	1	1	1	1
**Photorhabdus**	**asymbiotica**		**2**	**1**	**1**	**0**	**0**	**1**	**0**	**1**	**1**
**Photorhabdus**	**luminescens**	**subsp. laumondii TTO1**	**2**	**1**	**1**	**0**	**0**	**1**	**0**	**1**	**1**
**Proteus**	**mirabilis**	**HI4320**	**9**	**1**	**1**	**0**	**0**	**1**	**0**	**1**	**1**
**Providencia**	**stuartii**	**MRSN 2154**	**5**	**1**	**1**	**0**	**0**	**1**	**0**	**2**	**1**
Rahnella	aquatilis	CIP 78.65 = ATCC 33071	17	1	1	0	0	1	1	1	1
**Rahnella**	**aquatilis**	**HX2**	**20**	**1**	**1**	**0**	**0**	**1**	**1**	**1**	**1**
**Rahnella**	**sp.**	**Y9602**	**20**	**1**	**1**	**0**	**0**	**1**	**1**	**1**	**1**
Raoultella	ornithinolytica	B6	0	0	0	0	0	0	0	0	0
**Salmonella**	**bongori**	**NCTC 12419**	**9**	**1**	**1**	**0**	**0**	**1**	**1**	**1**	**1**
Salmonella	enterica	subsp. arizonae serovar 62:z4,z23:- str. RSK2980	8	1	1	0	0	1	1	1	1
Salmonella	enterica	subsp. enterica serovar Agona str. SL483	8	1	1	0	0	1	1	1	1

Salmonella	enterica	subsp. enterica serovar Choleraesuis str. SC-B67	9	1	1	0	0	1	1	1	1
Salmonella	enterica	subsp. enterica serovar Dublin str. CT_02021853	9	1	1	0	0	1	1	1	1
Salmonella	enterica	subsp. enterica serovar Enteritidis str. P125109	9	1	1	0	0	1	1	1	1
Salmonella	enterica	subsp. enterica serovar Gallinarum str. 287/91	8	1	1	0	0	1	1	1	1
Salmonella	enterica	subsp. enterica serovar Gallinarum/pullorum	9	1	1	0	0	1	1	1	1
Salmonella	enterica	subsp. enterica serovar Heidelberg str. B182	9	1	1	0	0	1	1	1	1
Salmonella	enterica	subsp. enterica serovar Heidelberg str. SL476	9	1	1	0	0	1	1	1	1
Salmonella	enterica	subsp. enterica serovar Javiana str. CFSAN001992	9	1	1	0	0	1	1	1	1
Salmonella	enterica	subsp. enterica serovar Newport str. SL254	9	1	1	0	0	1	1	1	1
Salmonella	enterica	subsp. enterica serovar Paratyphi A str. AKU_12601	6	1	1	0	0	1	1	1	1
Salmonella	enterica	subsp. enterica serovar Paratyphi A str. ATCC 9150	6	1	1	0	0	1	1	1	1
Salmonella	enterica	subsp. enterica serovar Paratyphi B str. SPB7	9	1	1	0	0	1	1	1	1
Salmonella	enterica	subsp. enterica serovar Paratyphi C strain RKS4594	8	1	1	0	0	1	1	1	1
Salmonella	enterica	subsp. enterica serovar Schwarzengrund str. CVM19633	9	1	1	0	0	1	1	1	1
**Salmonella**	**enterica**	**subsp. enterica serovar Typhi str. CT18**	**6**	**1**	**1**	**0**	**0**	**1**	**1**	**1**	**1**
Salmonella	enterica	subsp. enterica serovar Typhi str. P-stx-12	7	1	1	0	0	1	1	1	1
Salmonella	enterica	subsp. enterica serovar Typhi str. Ty2	6	1	1	0	0	1	1	1	1
Salmonella	enterica	subsp. enterica serovar Typhimurium str. 14028S	9	1	1	0	0	1	1	1	1
Salmonella	enterica	subsp. enterica serovar Typhimurium str. 798	9	1	1	0	0	1	1	1	1
Salmonella	enterica	subsp. enterica serovar Typhimurium str. D23580	9	1	1	0	0	1	1	1	1
**Salmonella**	**enterica**	**subsp. enterica serovar Typhimurium str. LT2**	**9**	**1**	**1**	**0**	**0**	**1**	**1**	**1**	**1**
Salmonella	enterica	subsp. enterica serovar Typhimurium str. SL1344	9	1	1	0	0	1	1	1	1
Salmonella	enterica	subsp. enterica serovar Typhimurium str. ST4/74	9	1	1	0	0	1	1	1	1
Salmonella	enterica	subsp. enterica serovar Typhimurium str. T000240	9	1	1	0	0	1	1	1	1
Salmonella	enterica	subsp. enterica serovar Typhimurium str. U288	9	1	1	0	0	1	1	1	1
Salmonella	enterica	subsp. enterica serovar Typhimurium str. UK-1	9	1	1	0	0	1	1	1	1
**Serratia**	**marcescens**	**FGI94**	**6**	**1**	**1**	**0**	**0**	**1**	**0**	**1**	**1**
Serratia	marcescens	WW4	7	1	1	0	0	1	0	1	1
**Serratia**	**plymuthica**	**AS9**	**4**	**1**	**1**	**0**	**0**	**1**	**0**	**1**	**1**
**Serratia**	**proteamaculans**	**568**	**6**	**1**	**1**	**0**	**0**	**1**	**0**	**1**	**1**
**Serratia**	**sp.**	**AS12**	**4**	**1**	**1**	**0**	**0**	**1**	**0**	**1**	**1**
**Serratia**	**sp.**	**AS13**	**4**	**1**	**1**	**0**	**0**	**1**	**0**	**1**	**1**
Serratia	symbiotica	str. 'Cinara cedri'	0	0	0	0	0	0	0	0	0
Shigella	boydii	CDC 3083-94	4	1	1	0	0	1	0	1	1
Shigella	boydii	Sb227	2	0	1	0	0	1	0	1	0
Shigella	dysenteriae	Sd197	2	0	0	0	0	0	0	0	0
Shigella	flexneri	2002017	4	1	1	0	0	1	0	1	1
Shigella	flexneri	2a str. 2457T	4	1	1	0	0	1	0	1	1
Shigella	flexneri	2a str. 301	4	1	1	0	0	1	0	1	1
Shigella	flexneri	5 str. 8401	4	1	1	0	0	1	0	1	1
Shigella	sonnei	Ss046	4	1	1	0	0	1	0	1	1
Sodalis	glossinidius	str. 'morsitans'	0	0	1	0	0	0	0	1	1
Wigglesworthia	glossinidia	endosymbiont of Glossina brevipalpis	0	0	0	0	0	0	0	0	0
Wigglesworthia	glossinidia	endosymbiont of Glossina morsitans morsitans (Yale colony)	0	0	0	0	0	0	0	0	0
**Xenorhabdus**	**bovienii**	**SS-2004**	**2**	**1**	**1**	**0**	**0**	**1**	**0**	**1**	**1**
**Xenorhabdus**	**nematophila**	**ATCC 19061**	**2**	**1**	**1**	**0**	**0**	**1**	**0**	**1**	**1**
**Yersinia**	**enterocolitica**	**subsp. enterocolitica 8081**	**12**	**1**	**1**	**0**	**0**	**1**	**1**	**1**	**1**
Yersinia	enterocolitica	subsp. palearctica 105.5R(r)	11	1	1	0	0	1	1	1	1
Yersinia	enterocolitica	subsp. palearctica Y11	11	1	1	0	0	1	1	1	1
Yersinia	pestis	A1122	8	1	1	0	0	1	0	1	1
Yersinia	pestis	Angola	7	1	1	0	0	1	0	1	1
Yersinia	pestis	Antiqua	9	1	1	0	0	1	0	1	1
Yersinia	pestis	biovar Medievalis str. Harbin 35	7	1	1	0	0	1	0	1	1
Yersinia	pestis	biovar Microtus str. 91001	7	1	1	0	0	1	0	1	1
Yersinia	pestis	CO92	6	1	1	0	0	1	0	1	1
Yersinia	pestis	D106004	9	1	1	0	0	1	0	1	1
Yersinia	pestis	D182038	9	1	1	0	0	1	0	1	1
Yersinia	pestis	KIM10+	7	1	1	0	0	1	0	1	1
Yersinia	pestis	Nepal516	9	1	1	0	0	1	0	1	1
Yersinia	pestis	Pestoides F	9	1	1	0	0	1	0	1	1
**Yersinia**	**pestis**	**Z176003**	**9**	**1**	**1**	**0**	**0**	**1**	**0**	**1**	**1**
Yersinia	pseudotuberculosis	IP 31758	8	1	1	0	0	1	0	1	1
**Yersinia**	**pseudotuberculosis**	**IP 32953**	**8**	**1**	**1**	**0**	**0**	**1**	**0**	**1**	**1**
Yersinia	pseudotuberculosis	PB1/+	8	1	1	0	0	1	0	1	1
Yersinia	pseudotuberculosis	YPIII	7	1	1	0	0	1	0	1	1
