# Supplementary material for: Evolutionary Genomics Suggests That CheV Is an Additional Adaptor for Accommodating Specific Chemoreceptors within the Chemotaxis Signaling Complex
Source: PLoS Comput Biol. 2016 Feb 4;12(2):e1004723. doi: 10.1371/journal.pcbi.1004723 (PMC4742279; doi:10.1371/journal.pcbi.1004723)
Supplement: S2 Table — Positions that are 100% conserved within each group and highly conserved group-specific positions are shown. Residue numbers indicated positions in the multiple sequence alignment. Position 280 corresponds to Gly278 in the E. coli Tar chemoreceptor. (PDF) [file pcbi.1004723.s002.pdf]

S2 Table. Highly conserved positions that distinguish COG1 chemoreceptors from COG2 chemoreceptors. Positions that are 100% conserved within each group and highly conserved group-specific positions are shown. Residue numbers indicated positions in the multiple sequence alignment. Position 280 corresponds to Gly287 in the *E. coli* Tar chemoreceptor.

Residue number	Consensus (%)												Overall Identity	
	COG 1						COG 2						COG1	COG2
	100	90	80	70	60	50	100	90	80	70	60	50		
235	t	G	G	G	G	G	G	G	G	G	G	G	0.99	1.00
305	o	T	T	T	T	T	T	T	T	T	T	T	0.99	1.00
315	s	T	T	T	T	T	T	T	T	T	T	T	0.99	1.00
384	l	V	V	V	V	V	V	V	V	V	V	V	0.99	1.00
386	u	A	A	A	A	A	A	A	A	A	A	A	0.99	1.00
391	.	E	E	E	E	E	E	E	E	E	E	E	0.99	1.00
397	.	A	A	A	A	A	A	A	A	A	A	A	0.94	1.00
490	h	L	L	L	L	L	L	L	L	L	L	L	0.99	1.00
497	s	A	A	A	A	A	A	A	A	A	A	A	0.95	1.00
504	p	Q	Q	Q	Q	Q	Q	Q	Q	Q	Q	Q	0.96	1.00
302	L	L	L	L	L	L	l	l	l	L	L	L	1.00	0.71
306	A	A	A	A	A	A	u	A	A	A	A	A	1.00	0.91
308	S	S	S	S	S	S	s	s	o	S	S	S	1.00	0.78
309	M	M	M	M	M	M	h	h	M	M	M	M	1.00	0.87
337	A	A	A	A	A	A	h	s	s	s	A	A	1.00	0.65
340	G	G	G	G	G	G	s	s	s	u	u	u	1.00	0.49
358	S	S	S	S	S	S	s	u	S	S	S	S	1.00	0.85
361	I	I	I	I	I	I	h	h	h	h	h	l	1.00	0.49
394	R	R	R	R	R	R	+	R	R	R	R	R	1.00	0.98
400	A	A	A	A	A	A	u	A	A	A	A	A	1.00	0.98
407	A	A	A	A	A	A	u	A	A	A	A	A	1.00	0.96
413	A	A	A	A	A	A	u	u	u	A	A	A	1.00	0.71
416	E	E	E	E	E	E	-	E	E	E	E	E	1.00	0.93
420	L	L	L	L	L	L	l	L	L	L	L	L	1.00	0.98
442	M	M	M	M	M	M	h	h	h	h	M	M	1.00	0.62
459	I	I	I	I	I	I	l	I	I	I	I	I	1.00	0.98
470	I	I	I	I	I	I	l	l	I	I	I	I	1.00	0.87
472	Q	Q	Q	Q	Q	Q	p	Q	Q	Q	Q	Q	1.00	0.91
476	A	A	A	A	A	A	s	A	A	A	A	A	1.00	0.96
480	M	M	M	M	M	M	h	h	h	h	l	l	1.00	0.47

485	Q	Q	Q	Q	Q	Q	p	Q	Q	Q	Q	Q	1.00	0.98
486	Q	Q	Q	Q	Q	Q	p	Q	Q	Q	Q	Q	1.00	0.98
501	L	L	L	L	L	L	h	L	L	L	L	L	1.00	0.98
508	L	L	L	L	L	L	h	L	L	L	L	L	1.00	0.98
15		l	L	L	L	L		F	F	F	F	F	0.8758	0.9636
247	.	N	N	N	N	N	s	D	D	D	D	D	0.9068	0.9273
280	t	G	G	G	G	G	s	A	A	A	A	A	0.9689	0.9091
