# Supplementary material for: Evolutionary Genomics Suggests That CheV Is an Additional Adaptor for Accommodating Specific Chemoreceptors within the Chemotaxis Signaling Complex
Source: PLoS Comput Biol. 2016 Feb 4;12(2):e1004723. doi: 10.1371/journal.pcbi.1004723 (PMC4742279; doi:10.1371/journal.pcbi.1004723)

S1. Fig. Vertical evolution of CheA in *Enterobacteriales*. Maximum likelihood phylogenetic trees of 16S ribosomal RNA and CheA proteins have nearly identical topology. Each sequence tag contains the first two letters of the genus, the first three letters of the species and the organism id in the MIST database, followed by the locus (for CheA) and accession number. The tag for also includes the chemotaxis class for CheA (e.g. F7).

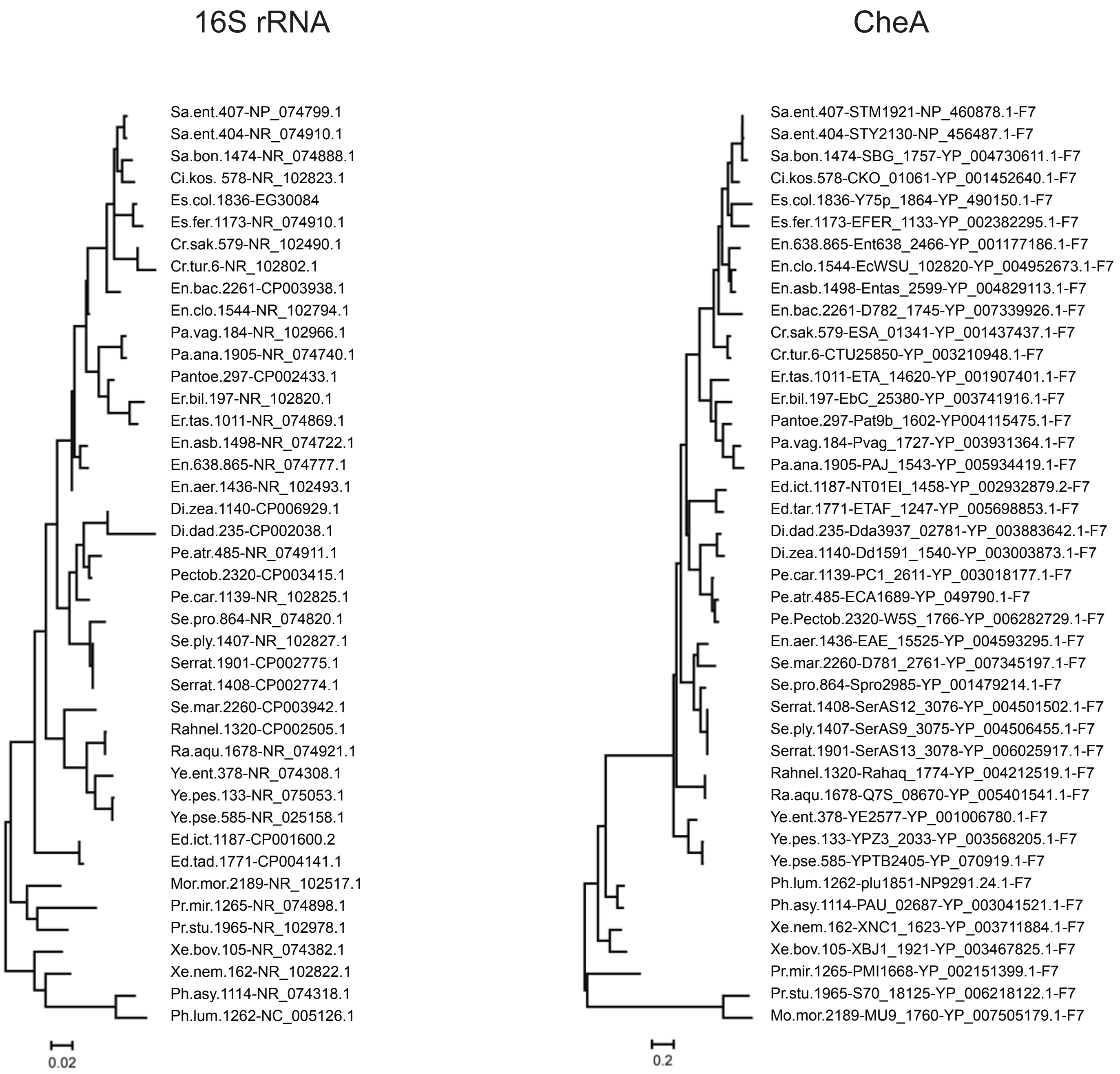

Supplement: S1 Fig — Maximum likelihood phylogenetic trees of 16S ribosomal RNA and CheA proteins have nearly identical topology. Each sequence tag contains the first two letters of the genus, the first three letters of the species and the organism id in the MIST database, followed by the locus (for CheA) and accession number. The tag also includes the chemotaxis class for CheA (e.g. F7). (PDF) [file pcbi.1004723.s003.pdf]
