# Supplementary material for: Evolutionary Genomics Suggests That CheV Is an Additional Adaptor for Accommodating Specific Chemoreceptors within the Chemotaxis Signaling Complex
Source: PLoS Comput Biol. 2016 Feb 4;12(2):e1004723. doi: 10.1371/journal.pcbi.1004723 (PMC4742279; doi:10.1371/journal.pcbi.1004723)
Supplement: S2 Fig — Comparison of the CheA and CheV phylogenetic trees suggests vertical evolution of CheV and supports the hypothesis that CheV was present in the common ancestor of Enterobacteriales. Each sequence tag contains the first two letters of the genus, the first three letters of the species and the organism id in the MIST database, followed by the locus and accession number. The tag also includes the chemotaxis class (e.g. F7). (PDF) [file pcbi.1004723.s004.pdf]

S2 Fig. Vertical evolution of CheV in *Enterobacteriales*. Comparison of the CheA and CheV phylogenetic trees suggests vertical evolution of CheV and supports the hypothesis that CheV was present in the common ancestor of *Enterobacteriales*. Each sequence tag contains the first two letters of the genus, the first three letters of the species and the organism id in the MIST database, followed by the locus and accession number. The tag also includes the chemotaxis class (e.g. F7).

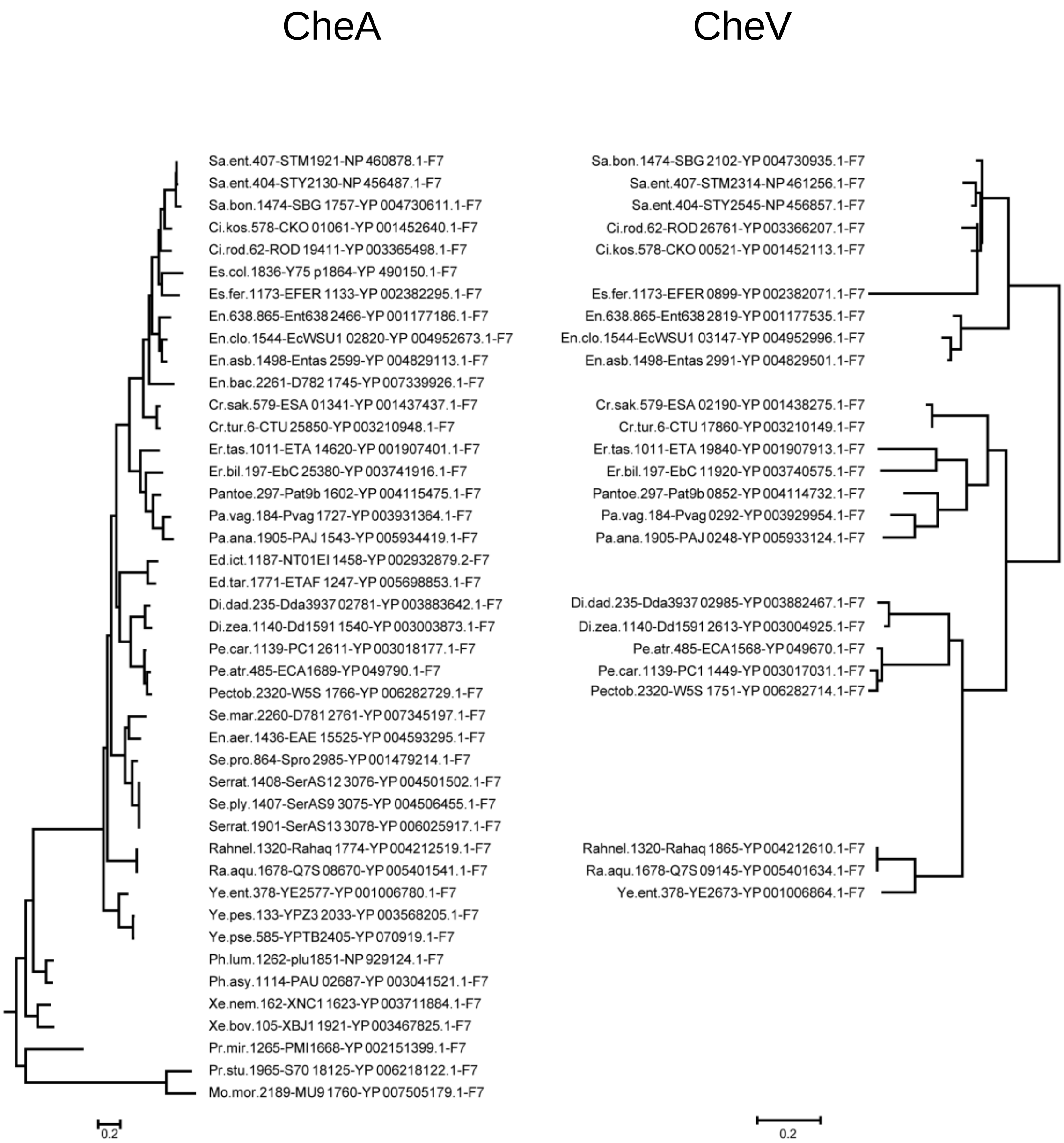

.
