# Supplementary material for: Evolutionary Genomics Suggests That CheV Is an Additional Adaptor for Accommodating Specific Chemoreceptors within the Chemotaxis Signaling Complex
Source: PLoS Comput Biol. 2016 Feb 4;12(2):e1004723. doi: 10.1371/journal.pcbi.1004723 (PMC4742279; doi:10.1371/journal.pcbi.1004723)
Supplement: S3 Fig — Each sequence tag contains the first two letters of the genus, the first three letters of the species and the organism id in the MiST database, followed by the locus and accession number. The tag also includes the chemotaxis class for CheA (e.g. F7) and shows the presence (1CheV) or absence (0CheV) of cheV genes in a corresponding genome. (PDF) [file pcbi.1004723.s005.pdf]

S3 Fig. Multiple sequence alignment of CheA-P2 sequences from the non-redundant *Enterobacteriales* genome set. Each sequence tag contains the first two letters of the genus, the first three letters of the species and the organism id in the MIST database, followed by the locus and accession number. The tag also includes the chemotaxis class for CheA (e.g. F7) and shows the presence (1CheV) or absence (0CheV) of *cheV* genes in a corresponding genome.

```
Ye.ent.378-YE2577-YP_001006780.1-F7-1CheV-1       RIRLSGLKEQEIPLMLEELGNLGEVKDPHQGADSLEATLITSVSEDDISAVLCFVLEPEQISF-
Sa.ent.404-STY2130-NP_456487.1-F7-1CheV-1         RIVLSRLKANEVDLLEEELGNLATLTDVVKGADSLSATLDGSVAEDDIVAVLCFVIEADQIAFE
Sa.ent.407-STM1921-NP_460878.1-F7-1CheV-1         RIVLSRLKANEVDLLEEELGNLATLTDVVKGADSLSATLDGSVAEDDIVAVLCFVIEADQIAFE
Ph.asy.1114-PAU_02687-YP_003041521.1-F7-0CheV-1   RVHLSGLKEREVSLMLEELGNLGEVYDAEQTRDSVEASLVTSATEDDITAVLCFVIEPEQITF-
Ye.pse.585-YPTB2405-YP_070919.1-F7-0CheV-1        RIRLSGLKAPEISLMLEELGNLGEVQDPHQGADSLEATLITSVSEEDISAVLCFVLEPEQISF-
Ye.pes.133-YPZ3_2033-YP_003568205.1-F7-0CheV-1    RIRLSGLKAPEISLMLEELGNLGEVQDPHQGADSLEATLITSVSEEDISAVLCFVLEPEQISF-
Es.col.1836-Y75_p1864-YP_490150.1-F7-0CheV-1      -IILSRLKAGEVDLLEEELGHLTTLTDVVKGADSLSAILPGDIAEDDITAVLCFVIEADQITFE
Pe.atr.485-ECA1689-YP_049790.1-F7-1CheV-1         RIALTGLKSQEIPQMLEELGNLGTVKDPHQTDTSVEVTLVTSESEDDISAVLCFVLEPEQISFK
Pectob.2320-W5S_1766-YP_006282729.1-F7-1CheV-1    RIALTGLKSQEIPQMLEELGNLGTVKDPHQTDTSVEVTLVTSESEDDISAVLCFVLEPEQISFK
Pe.car.1139-PC1_2611-YP_003018177.1-F7-1CheV-1    RIALTGLKSQEIPQMLEELGNLGTVKDPHQTDTSVEVTLVTSESEDDISAVLCFVLEPEQISFK
Sa.bon.1474-SBG_1757-YP_004730611.1-F7-1CheV-1    RIVLSRLKASEVDLLEEELGNLATLTDVVKGADSLSATLDGSVAEEDIVAVLCFVIEADQIAFE
Ra.aqu.1678-Q7S_08670-YP_005401541.1-F7-1CheV-1   RVRLSGLKTQEIPVMLEELGNLGEIKDPVQTDDSVEATLVTTVSEDDIIAVLCFVLEPEQISFT
Rahnel.1320-Rahaq_1774-YP_004212519.1-F7-1CheV-1  RVRLSGLKTQEIPVMLEELGNLGEIKDPVQTDDSVEATLVTTVSEDDIIAVLCFVLEPEQISFT
Di.zea.1140-Dd1591_1540-YP_003003873.1-F7-1CheV-1 RIALTNLKESDIPQLLEELGNLGTVKDTVQTSSSVELTLDTSASEDDISAVLCFVLDPDQISFK
Ci.kos.578-CKO_01061-YP_001452640.1-F7-1CheV-1    RVVLSRLKANEVDLLEEELGNLATLTDVVKGEDSLSATLDGTIAEDDIIAVLCFVIEADQIAFE
Ci.rod.62-ROD_19411-YP_003365498.1-F7-1CheV-1     RIVLSRLKASEVDLLEEELGNLATLTEVVKGSDSLAATLDGDIAEDDIIAVLCFVIEADQIAFE
Di.dad.235-Dda3937_02781-YP_003883642.1-F7-1CheV-1 RIALTNLKESDIPQLLEELGNLGTVKDTAQTSTSVELTLDTSASEDDISAVLCFVLEPDQINFK
Xe.bov.105-XBJ1_1921-YP_003467825.1-F7-0CheV-1    RVHLSGLKEREVSLMKDELGHLGDVYDVEQTADSLEASLITSATEDDITAVMCFVIEPEQITF-
Ed.tar.1771-ETAF_1247-YP_005698853.1-F7-0CheV-1   -ISLTGLKAAEIPLMLEELGNLGTVQQHQESDSALDVVLETTVSEDDITAVLCFVLEPEQIHFT
Ph.lum.1262-plu1851-NP_929124.1-F7-0CheV-1        RVHLSGLKEREVSLMLDELSNLGEVYDAEQTRDSVEASLVTSATEDDITAVLCFVIEPEQITF-
Ed.ict.1187-NT01EI_1458-YP_002932879.2-F7-0CheV-1 -VSLTGLKPAEIPLMLEELGNLGTVQQHQESDSSLEVVLDTTVSEDDITAVLCFVLEPEQIHFT
En.aer.1436-EAE_15525-YP_004593295.1-F7-0CheV-1   RVSLTGLKANEIPLMLEELGNLGEVHDPQQTDNSLEVTLLTTASEEDICAVLCFVLEPEQISFT
Se.pro.864-Spro_2985-YP_001479214.1-F7-0CheV-1    RICLSGLKSSEVPLMLEELGNLGEVKNPQQTENSLEVTLLTSASEDDISAVLCFVLEPEQISF-
Er.tas.1011-ETA_14620-YP_001907401.1-F7-1CheV-1   RIQLTGLKVNEVDLMLEELANLGTVSAAVKGENSLEATLDSSVGKDDIVAVLCFVIDESQIHF-
En.asb.1498-Entas_2599-YP_004829113.1-F7-1CheV-1  RVVLSRLKESEVNLLEEELGNLAKLSNVVKGKDSLAATLEDGISQDDIVAVLCFVIEADQIDFE
Se.ply.1407-SerAS9_3075-YP_004506455.1-F7-0CheV-1 RVCLSGLKPNEIPLMLEELGNLGEVKNPQQTESSLEVTLLTSASEDDISAVLCFVLEPEQISF-
Serrat.1408-SerAS12_3076-YP_004501502.1-F7-0CheV-1 RVCLSGLKPNEIPLMLEELGNLGEVKNPQQTESSLEVTLLTSASEDDISAVLCFVLEPEQISF-
Serrat.1901-SerAS13_3078-YP_006025917.1-F7-0CheV-1 RVCLSGLKPNEIPLMLEELGNLGEVKNPQQTESSLEVTLLTSASEDDISAVLCFVLEPEQISF-
Xe.nem.162-XNC1_1623-YP_003711884.1-F7-0CheV-1    RIHLSGLKEREVALMKDELAHLGEIYDVEQTSNSLEASLITSATEDDITAVLCFVVEPEQITF-
En.638.865-Ent638_2466-YP_001177186.1-F7-1CheV-1  RVVLSRLKESEVNLLEEELGNLATLSNVVKGKDSLAATIDGGIGQDDIVAVLCFVIEADQIAFE
En.clo.1544-EcWSU1_02820-YP_004952673.1-F7-1CheV-1 RVVLSRLKENEVDLLEEELSNLAKLSNVVKGKDSLSATLEEGISQDDIVAVLCFVIEADQIAFE
Cr.sak.579-ESA_01341-YP_001437437.1-F7-1CheV-1    RIKLGKLKGNEIELLQEELGNLGALSQIEKGADHLIATLETSASADDITAVLCFVIEGDQIEF-
Es.fer.1173-EFER_1133-YP_002382295.1-F7-1CheV-1   RIVLSRLKSKEVDLLEAELRNLATLSEVVKGEDSLAATLEGDIAQDDIVAVLCFVIEEDQIHFE
Se.mar.2260-D781_2761-YP_007345197.1-F7-0CheV-1   HISLTDLKPNEIPLLLEELGNLGEVRNPQQSDSSLSVTLLTSVSAEDISAVLCFVLEPEQIAF-
Cr.tur.6-CTU_25850-YP_003210948.1-F7-1CheV-1      RIKLGKLKGTETELLQEELGNLGALSQIEKGEDYLVATLETSASADDITAVLCFVIEGDQIEF-
Er.bil.197-EbC_25380-YP_003741916.1-F7-1CheV-1    RLALVDLKPAEIPLMLEELGNLGTVSDVVKGETSVEVTLDSTVDQDDIVAVLCFVIEEAQIQF-
Pr.mir.1265-PMI1668-YP_002151399.1-F7-0CheV-1     --ILSDLKETDIDLMLDELKHLGEVSQVEKQHHGLEAVLKTTATQEDISAVLCFVIEPEQISF-
En.bac.2261-D782_1745-YP_007339926.1-F7-0CheV-1   -ITLSRLKPGEPEMLQEELRNLATLSDVTLGVDSLSATLEGDVNQDDIVAVLCFVIEADQISF-
Pa.ana.1905-PAJ_1543-YP_005934419.1-F7-1CheV-1    --QLVDLKEKDIDLMLEELGNLGTISDVKKGSNSLECHIT-GVGKDDIVAVLCFVIDESQIRF-
Pa.vag.184-Pvag_1727-YP_003931364.1-F7-1CheV-1    RVQLIDLKEKEVDLMLEEMSNLGTLTNVQKGSTTLDVCID-GVGKDDIVAVLCFVIDEAQIRF-
Pantoe.297-Pat9b_1602-YP_004115475.1-F7-1CheV-1   RLQLVDLKEKEPELLLEELGNLGTLSDVVKGANTLEATID-GVGKDDIVAVLCFVIEEAQIRF-
Pr.stu.1965-S70_18125-YP_006218122.1-F7-0CheV-1   --TLSQLKANEVDLLAEELALFGTLYDTEKTADSLKAWLGTNTVIDDICGVLCFVVDESQIT--
Mo.mor.2189-MU9_1760-YP_007505179.1-F7-0CheV-1    ---LSKLKESEVALLADELSLFGTVYKTEQGTDTLKVWLGTNTAIDDISGVLCFVVDDSQIA--
```
