# Supplementary material for: Evolutionary Genomics Suggests That CheV Is an Additional Adaptor for Accommodating Specific Chemoreceptors within the Chemotaxis Signaling Complex
Source: PLoS Comput Biol. 2016 Feb 4;12(2):e1004723. doi: 10.1371/journal.pcbi.1004723 (PMC4742279; doi:10.1371/journal.pcbi.1004723)

S4 Fig. Conservation patterns in the CheA-P2 domains in organisms with CheV and without CheV. Sequence logos were generated from the multiple sequence alignment shown in S3 Fig.

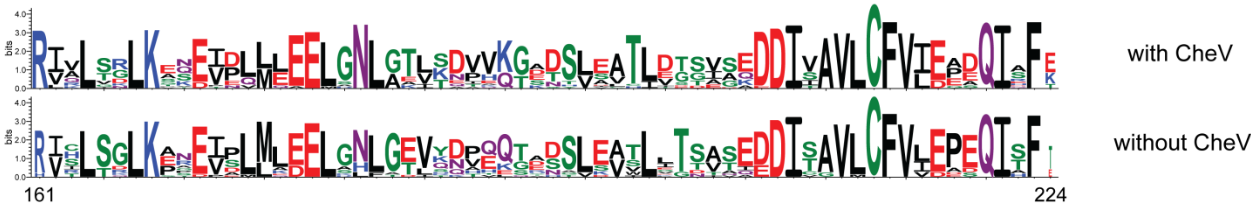

Supplement: S4 Fig — Sequence logos were generated from the multiple sequence alignment shown in S3 Fig (PDF) [file pcbi.1004723.s006.pdf]
