# Supplementary material for: Evolutionary Genomics Suggests That CheV Is an Additional Adaptor for Accommodating Specific Chemoreceptors within the Chemotaxis Signaling Complex
Source: PLoS Comput Biol. 2016 Feb 4;12(2):e1004723. doi: 10.1371/journal.pcbi.1004723 (PMC4742279; doi:10.1371/journal.pcbi.1004723)
Supplement: S6 Fig — Each sequence tag contains the first two letters of the genus, the first three letters of the species and the organism id in the MIST database, followed by the locus and accession number. The tag also includes the chemotaxis class for CheV (e.g. F7) and shows the presence (1CheV) or absence (0CheV) of cheV genes in a corresponding genome. (PDF) [file pcbi.1004723.s008.pdf]

S6 Fig. Multiple sequence alignment of CheY and CheV-RR sequences from the non-redundant *Enterobacteriales* genome set. Each sequence tag contains the first two letters of the genus, the first three letters of the species and the organism id in the MIST database, followed by the locus and accession number. The tag also includes the chemotaxis class for CheV (e.g. F7) and shows the presence (1CheV) or absence (0CheV) of *cheV* genes in a corresponding genome.

```
Pe.car.1139-PC1_2606-YP_003018172.1-CheY-1-1CheV     VVDDFSTMRRIVRNLLKELG------------FNNVEEAEDGADALNKLRAGAFDFVISD
En.clo.1544-EcWSU1_02807-YP_004952660.1-CheY-1-1CheV VVDDFSTMRRIVRNLLKELG------------FNNVEEAEDGVDALNKLQAGGFGFVISD
Pa.vag.184-Pvag_1720-YP_003931357.1-CheY-1-1CheV     VVDDFNTMRRIVRNLLKELG------------FNNVEEAEDGVDALGKLKAGGFDFVISD
Sa.bon.1474-SBG_1752-YP_004730606.1-CheY-1-1CheV     VVDDFSTMRRIVRNLLKELG------------FNNVEEAEDGVDALNKLQAGGFGFIISD
Pe.atr.485-ECA1694-YP_049795.1-CheY-1-1CheV          VVDDFSTMRRIVRNLLKELG------------FNNVEEAEDGSDALNKLRSGAFDFVISD
Pa.ana.1905-PAJ_1537-YP005934413.1-CheY-1-1CheV      VVDDFNTMRRIVRNLLKELG------------FNNVEEAEDGVDALGKLKAGGFDFVISD
En.asb.1498-Entas_2587-YP_004829101.1-CheY-1-1CheV   VVDDFSTMRRIVRNLLKELG------------FNNVEEAEDGVDALNKLQAGGFGFVISD
Sa.ent.407-STM1961-NP_460873.1-CheY-1-1CheV          VVDDFSTMRRIVRNLLKELG------------FNNVEEAEDGVDALNKLQAGGFGFIISD
Es.fer.1173-EFER_1144-YP_002382306.1-CheY-1-1CheV    VVDDFSTMRRIVRNLLKELG------------FHNVEEAEDGVDALNKLQMGGFNFVISD
Ra.aqu.1678-Q7S_08980-YP_005401601.1-CheY-1-1CheV    VVDDFSTMRRIVRNLLKELG------------FNNVEEAEDGVDALNKLRAGGFDFVVSD
Ci.rod.62-ROD_19301-YP_003365487.1-CheY-1-1CheV      VVDDFSTMRRIVRNLLKELG------------FNNVEEAEDGVDALNKLQAGGFGFVISD
Sa.ent.404-STY2125-NP_456482.1-CheY-1-1CheV          VVDDFSTMRRIVRNLLKELG------------FNNVEEAEDGVDALNKLQAGGFGFIISD
Di.zea.1140-Dd1591_1545-YP_003003878.1-CheY-1-1CheV  VVDDFSTMRRIVRNLLKELG------------FNNVEEAEDGADALNKLRSGGFDFVISD
Ci.kos.578-CKO_01070-YP_001452649.1-CheY-1-1CheV     VVDDFSTMRRIVRNLLKELG------------FNNVEEAEDGVDALNKLQASGFDFVISD
Cr.sak.579-ESA_01352-YP_001437448.1-CheY-1-1CheV     VVDDFSTMRRIVRNLLKELG------------FNNVEEAEDGVDALNKLQTGGFGFVISD
Di.dad.235-Dda3937_02776-YP_003883637.1-CheY-1-1CheV VVDDFSTMRRIVRNLLKELG------------FNNVEEAEDGADALNKLRSGGFDFVISD
Ye.ent.378-YE2570-YP_001006774.1-CheY-1-1CheV        VVDDFSTMRRIVRNLLKELG------------FNNVEEAEDGVDALNKLRTGGFDFVVSD
Cr.tur.6-CTU_25740-YP_003210937.1-CheY-1-1CheV       VVDDFSTMRRIVRNLLKELG------------FNNVEEAEDGVDALNKLQTGGFGFVISD
Er.tas.1011-ETA_14700-YP_001907409.1-CheY-1-1CheV    VVDDFNTMRRIVRNLLKELG------------FNNVEEAEDGVDALNKLRAGGFDFVVSD
Er.bil.197-EbC_25310-YP_003741909.1-CheY-1-1CheV     VVDDFNTMRRIVRNLLKELG------------FNNVEEAEDGVDALHKLRAGGFDFVVSD
Pa.ana.1905-PAJ_0248-YP_005933124.1-F7-1-RR_CheV     VAEDSKVARQLLEQGLKSMGIPAIMCNTGLEAWEKIKEMHQQAQAQGESIHDKIALVLTD
Pa.vag.184-Pvag_0292-YP_003929954.1-F7-1-RR_CheV     VAEDSKVARQLLEQGLKSMGIPALMFNTGLEAWEKIKLMSQEAQAAGESIHDKIALVLTD
Er.tas.1011-ETA_19840-YP_001907913.1-F7-1-RR_CheV    VAEDSKVARQMLEQGLKAMGIPAMMYNTGLEAWVKIKQMYQEAQAEGVSIHDKIGLILTD
Pantoe.297-Pat9b_0852-YP_004114732.1-F7-1-RR_CheV    VAEDSKVARQLLEQGLQSMGIPAIMHNTGLEAWQKITQMQQEAQAAGESIHDKIALVLTD
Pectob.2320-W5S_1751-YP_006282714.1-F7-1-RR_CheV     VAEDSKVARSLLEQGLTQMGIPFSMHITGQDAWNKIRQIAQEAQSEGRPISDKISLVLTD
Pe.atr.485-ECA1568-YP_049670.1-F7-1-RR_CheV          VAEDSKVARSLLEQGLTQMGIPFSMHITGQDAWNKIRQIAQEAQSEGRPISDKISLVLTD
Pe.car.1139-PC1_1449-YP_003017031.1-F7-1-RR_CheV     VAEDSKVARSLLEQGLTQMGIPFSMHITGQDAWNKIRQIAQEAQSEGRPISDKISLVLTD
Er.bil.197-EbC_11920-YP_003740575.1-F7-1-RR_CheV     VAEDSKVARQMLEQGLKAMGIPAEMHNTGLEAWNRIRSIQQQAKEEGIPIRDKISMVLTD
Es.fer.1173-EFER_0899-YP_002382071.1-F7-1-RR_CheV    VAEDSKVARALLEKVLNTMQIPHVMHITGKSAWENIQQLARQAKEEGVPVSEKIALVLTD
Cr.tur.6-CTU_17860-YP_003210149.1-F7-1-RR_CheV       VAEDSKVARSMLEHGLKSMGIPALMHTTGLEAWEKIKLMAKEAQAEGQSITDKIAMVLTD
Cr.sak.579-ESA_02190-YP_001438275.1-F7-1-RR_CheV     VAEDSKVARSMLEHGLKSMGIPALMHTTGLEAWEKIKLMAKEAQAEGQSITDKIAMVLTD
Ra.aqu.1678-Q7S_09145-YP_005401634.1-F7-1-RR_CheV    VAEDSKVARQMLEKGLNMMEIPAQMHVTGLEAWNKIRKMAEECKAQGLPISDKISFVLTD
Rahnel.1320-Rahaq_1865-YP_004212610.1-F7-1-RR_CheV   VAEDSKVARQMLEKGLNMMEIPAQMHVTGLEAWNKIRKMAEECKAQGLPISDKISFVLTD
Ye.ent.378-YE2673-YP_001006864.1-F7-1-RR_CheV        VAEDSKVARSMLEQALKMMDIPAVMHITGLEAWNKIKKIADEARAEGRPVSDKISFVLTD
En.638.865-Ent638_2819-YP_001177535.1-F7-1-RR_CheV   VAEDSKVARSMLEKGLEAMNIPAEMHITGKEAWEKIGVLAAQAQAEGVPITDKISLVLTD
Sa.ent.407-STM2314-NP_461256.1-F7-1-RR_CheV          VAEDSKVARAMLEKGLNAMGIPHQMHVTGKDAWERIQQLAQEAEAEGKPISEKIALVLTD
Sa.bon.1474-SBG_2102-YP_004730935.1-F7-1-RR_CheV     VAEDSKVARAMLEKGLNAMEIPHQMHVTGKDAWEKIQQLAQEAETEGKPISEKIALVLTD
Sa.ent.404-STY2545-NP_456857.1-F7-1-RR_CheV          VAEDSKVACAMLEKGLNAMEIPHQMHVTGKDAWERIQQLAQEAEAEGKPISEKIALVLTD
Ci.kos.578-CKO_00521-YP_001452113.1-F7-1-RR_CheV     VAEDSKVARAMLEKGLNAMEIPHQMHVTGQDAWEKIQQLAKEAEAEGKPISEKIALVLTD
En.clo.1544-EcWSU1_03147-YP_004952996.1-F7-1-RR_CheV VAEDSKVARSMLEKGLQAMEIPAQLHITGKDAWEKISVLAAQAQAEGVPITDKIALVLTD
En.asb.1498-Entas_2991-YP_004829501.1-F7-1-RR_CheV   VAEDSKVARSMLEKGLQAMEIPAQLHITGKDAWEKIGVLAAQAQAEGVPITDKIALVLTD
Di.dad.235-Dda3937_02985-YP_003882467.1-F7-1-RR_CheV VAEDSKVARTMLETGLKVMNIPYIMHITGLEAWNKIKAMAEEAKAEGRPISDKIAFVLTD
Ci.rod.62-ROD_26761-YP_003366207.1-F7-1-RR_CheV      VAEDSRVARAMLEKGLNAMDIPHQMHVTGKDAWEKILLLAKEAEAEGKAISEKIALVLTD
Di.zea.1140-Dd1591_2613-YP_003004925.1-F7-1-RR_CheV  VAEDSKVARNMLETGLKVMNIPYIMHITGLEAWNRIKLMAQEAQAEGRPISDKIAFVLTD
```

```
Pe.car.1139-PC1_2606-YP_003018172.1-CheY-1-1CheV     WNMPNMDGLELLQTIRADGSLSSLPVLMVTAEAKKENIIAAAQAGASGYVVKPFTAATLEEKL
En.clo.1544-EcWSU1_02807-YP_004952660.1-CheY-1-1CheV WNMPNMDGLELLKTIRADAGMASLPVLMVTAEAKKENIIAAAQAGASGYVVKPFTAATLEEKL
Pa.vag.184-Pvag_1720-YP_003931357.1-CheY-1-1CheV     WNMPNMDGLQLLQTIRADAAMSSLPVLMVTAEAKKENIIAAAQAGASGYVVKPFTAATLEEKL
Sa.bon.1474-SBG_1752-YP_004730606.1-CheY-1-1CheV     WNMPNMDGLELLKTIRADSAMSALPVLMVTAEAKKENIIAAAQAGASGYVVKPFTAATLEEKL
Pe.atr.485-ECA1694-YP_049795.1-CheY-1-1CheV          WNMPNMDGLELLQTIRADGALSKLPVLMVTAEAKKENIIAAAQAGASGYVVKPFTAATLEEKL
Pa.ana.1905-PAJ_1537-YP005934413.1-CheY-1-1CheV      WNMPNMDGLQLLQTIRADAAMASLPVLMVTAEAKKENIIAAAQAGASGYVVKPFTAATLEEKL
En.asb.1498-Entas_2587-YP_004829101.1-CheY-1-1CheV   WNMPNMDGLELLKTIRADAGMASLPVLMVTAEAKKENIIAAAQAGASGYVVKPFTAATLEEKL
Sa.ent.407-STM1961-NP_460873.1-CheY-1-1CheV          WNMPNMDGLELLKTIRADSAMSALPVLMVTAEAKKENIIAAAQAGASGYVVKPFTAATLEEKL
Es.fer.1173-EFER_1144-YP_002382306.1-CheY-1-1CheV    WNMPNMDGLELLKTIRADGAMSTLPVLMVTAEAKKENIIAAAQAGASGYVVKPFTAATLEEKL
Ra.aqu.1678-Q7S_08980-YP_005401601.1-CheY-1-1CheV    WNMPNMDGLDLLKTIRADAALGKLPVLMVTAEAKKENIVAAAQAGASGYVVKPFTAATLEEKL
Ci.rod.62-ROD_19301-YP_003365487.1-CheY-1-1CheV      WNMPNMDGLELLKTIRADSVMASMPVLMVTAEAKKENIIAAAQAGASGYVVKPFTAATLEEKL
Sa.ent.404-STY2125-NP_456482.1-CheY-1-1CheV          WNMPNMDGLELLKTIRADSAMSALPVLMVTAEAKKENIIAAAQAGASGYVVKPFTAATLEEKL
Di.zea.1140-Dd1591_1545-YP_003003878.1-CheY-1-1CheV  WNMPNMDGLELLQAIRADGSLSKLPVLMVTAEAKKENIIAAAQAGASGYVVKPFTAATLEEKL
Ci.kos.578-CKO_01070-YP_001452649.1-CheY-1-1CheV     WNMPNMDGLELLKTIRADGAMASLPVLMVTAEAKKENIIAAAQAGASGYVVKPFTAATLEEKL
Cr.sak.579-ESA_01352-YP_001437448.1-CheY-1-1CheV     WNMPNMDGLELLKTIRADGAMASMPVLMVTAEAKKENIIAAAQAGASGYVVKPFTAATLEEKL
Di.dad.235-Dda3937_02776-YP_003883637.1-CheY-1-1CheV WNMPNMDGLELLQAIRADGTLSKLPVLMVTAEAKKENIIAAAQAGASGYVVKPFTAATLEEKL
Ye.ent.378-YE2570-YP_001006774.1-CheY-1-1CheV        WNMPNMDGLDLLKTIRADGALGTLPVLMVTAEAKKENIIAAAQAGASGYVVKPFTAATLEEKL
Cr.tur.6-CTU_25740-YP_003210937.1-CheY-1-1CheV       WNMPNMDGLELLKTIRADGGMASMPVLMVTAEAKKENIIAAAQAGASGYVVKPFTAATLEEKL
Er.tas.1011-ETA_14700-YP_001907409.1-CheY-1-1CheV    WNMPNMDGLELLKTIRTDGALSKLPVLMVTAEAKKENIIAAAQAGASGYVVKPFTAATLEEKL
Er.bil.197-EbC_25310-YP_003741909.1-CheY-1-1CheV     WNMPNMDGLELLKTIRADAAMAKLPVLMVTAEAKKENIIAAAQAGASGYVVKPFTAATLEEKL
Pa.ana.1905-PAJ_0248-YP_005933124.1-F7-1-RR_CheV     LEMPEMDGFTLTRNIKRDTTLRHIPVVIHSSLSGSANEDHVRKVGADGYVAK-FELNELSD--
Pa.vag.184-Pvag_0292-YP_003929954.1-F7-1-RR_CheV     LEMPEMDGFTLTRNIKRDPILKVLPVVIHSSLSGSANEDHVRKVGADGYVAK-FELNELSD--
Er.tas.1011-ETA_19840-YP_001907913.1-F7-1-RR_CheV    LEMPEMDGFTLTRNIKSDPNLKNIPVVIHSSLSGSANEDHVRKVGANGYVAK-FEIKQLSA--
Pantoe.297-Pat9b_0852-YP_004114732.1-F7-1-RR_CheV    LEMPEMDGFTLTRNIKRDDVLRHIPVVIHSSLSGSANEDHVRKVGADGYVAK-FEINELS---
Pectob.2320-W5S_1751-YP_006282714.1-F7-1-RR_CheV     LEMPEMDGFTLTRNIKREEYLKNIPVVIHSSLSGSANEDHVRNVGADAYVAK-FEINELSTAI
Pe.atr.485-ECA1568-YP_049670.1-F7-1-RR_CheV          LEMPEMDGFTLTRNIKREEFLKNIPVVIHSSLSGSANEDHVRNVGADAYVAK-FEINELSTAI
Pe.car.1139-PC1_1449-YP_003017031.1-F7-1-RR_CheV     LEMPEMDGFTLTRNIKREEFLKNIPVVIHSSLSGSANEDHVRNVGADAYVAK-FEINELSSAI
Er.bil.197-EbC_11920-YP_003740575.1-F7-1-RR_CheV     LEMPEMDGFTLTRNIKTDINLRDIPVVIHSSLSGSANEDHVRKVGANGYVAK-FEINQLSA--
Es.fer.1173-EFER_0899-YP_002382071.1-F7-1-RR_CheV    LEMPEMDGFTLTRLIKTDEQLKKIPVIIHSSLSGNANEDHVRKVKADGYVAK-FEMNELSA--
Cr.tur.6-CTU_17860-YP_003210149.1-F7-1-RR_CheV       LEMPEMDGFTLTRNIKMDANLKKIPVVIHSSLSGSANEDHVRKVGADGYVAK-FEINELSAAI
Cr.sak.579-ESA_02190-YP_001438275.1-F7-1-RR_CheV     LEMPEMDGFTLTRNIKMDATLKKIPVVIHSSLSGSANEDHVRKVGADGYVAK-FEINELSAAI
Ra.aqu.1678-Q7S_09145-YP_005401634.1-F7-1-RR_CheV    LEMPEMDGFTLTLNIKRDEFLKNIPVIIHSSLSGSANEDHVRKVGADGYVAK-FEINELEAAI
Rahnel.1320-Rahaq_1865-YP_004212610.1-F7-1-RR_CheV   LEMPEMDGFTLTLNIKRDEFLKNIPVIIHSSLSGSANEDHVRKVGADGYVAK-FEINELEAAI
Ye.ent.378-YE2673-YP_001006864.1-F7-1-RR_CheV        LEMPEMDGFTLTLNIKRDEFLKNIPVIIHSSLSGSANEDHVRKVGADAYVAK-FEANELEAAI
En.638.865-Ent638_2819-YP_001177535.1-F7-1-RR_CheV   LEMPEMDGFTLTRKIKTDEILKHIPVVIHSSLSGNANEDHIRKVKADGYVAK-FELNELSA--
Sa.ent.407-STM2314-NP_461256.1-F7-1-RR_CheV          LEMPEMDGFTLTRKIKTDERLKKIPVVIHSSLSGSANEDHIRKVKADGYVAK-FEINELSS--
Sa.bon.1474-SBG_2102-YP_004730935.1-F7-1-RR_CheV     LEMPEMDGFTLTRKIKTDERLKKIPVVIHSSLSGSANEDHVRKVKADGYVAK-FEVNEL----
Sa.ent.404-STY2545-NP_456857.1-F7-1-RR_CheV          LEMPEMDGFTLTRKIKTDERLKKIPVVIHSSLSGSANEDHVRKVKADGYVAK-FEINELSS--
Ci.kos.578-CKO_00521-YP_001452113.1-F7-1-RR_CheV     LEMPEMDGFTLTRKIKTDERLKKIPVVIHSSLSGSANEDHVRKVKADGYVAK-FEVNEL----
En.clo.1544-EcWSU1_03147-YP_004952996.1-F7-1-RR_CheV LEMPEMDGFTLTRKIKTDPLLKDIPVVIHSSLSGNANEDHIRKVKADGYVAK-FELNELS---
En.asb.1498-Entas_2991-YP_004829501.1-F7-1-RR_CheV   LEMPEMDGFTLTRKIKTDPVLKDIPVVIHSSLSGNANEDHIRKVKADGYVAK-FELNELS---
Di.dad.235-Dda3937_02985-YP_003882467.1-F7-1-RR_CheV LEMPEMDGFTLTRNIKRDEVLKSIPVIIHSSLSGTANEDHVRNVGADSYVAK-FEINELAAAI
Ci.rod.62-ROD_26761-YP_003366207.1-F7-1-RR_CheV      LEMPEMDGFTLTRKIKTDERLKKIPVVIHSSLSGSANEDHVRKVKADGYVAK-FEINELSS--
Di.zea.1140-Dd1591_2613-YP_003004925.1-F7-1-RR_CheV  LEMPEMDGFTLTRNIKRDEALKHIPVIIHSSLSGTANEDHVRNVGADSYVAK-FEINELAAAI
```
