# Supplementary material for: Evolutionary Genomics Suggests That CheV Is an Additional Adaptor for Accommodating Specific Chemoreceptors within the Chemotaxis Signaling Complex
Source: PLoS Comput Biol. 2016 Feb 4;12(2):e1004723. doi: 10.1371/journal.pcbi.1004723 (PMC4742279; doi:10.1371/journal.pcbi.1004723)

**S7 Fig. Conservation patterns in the CheA-P domains and in CheW proteins in organisms with CheV and without CheV.**

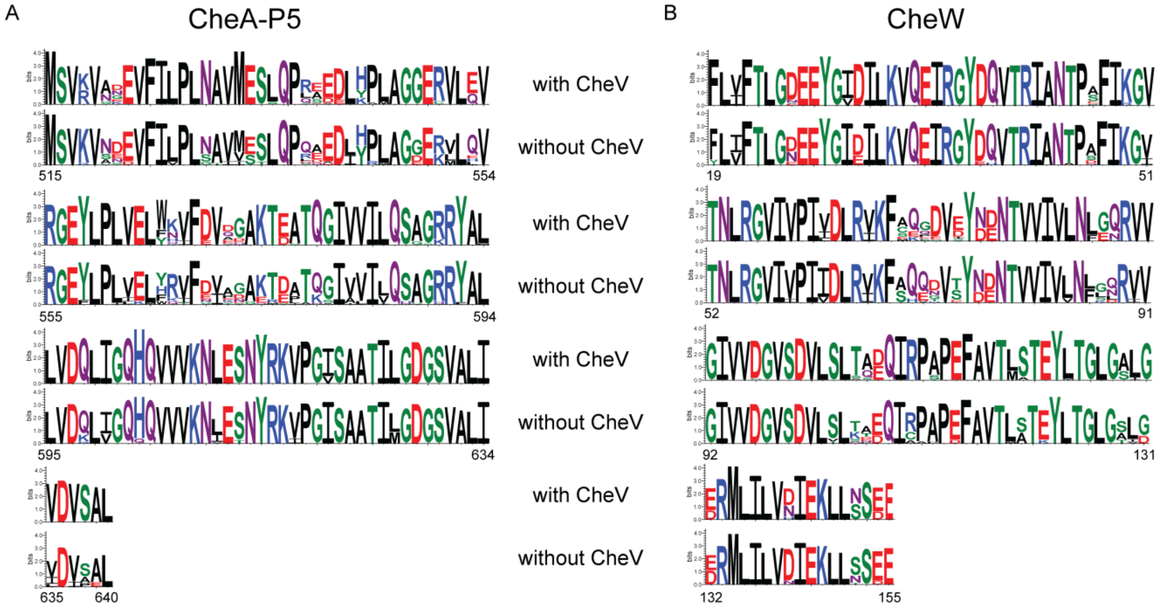

Supplement: S7 Fig — (PDF) [file pcbi.1004723.s009.pdf]
