# Supplementary material for: Evolutionary Genomics Suggests That CheV Is an Additional Adaptor for Accommodating Specific Chemoreceptors within the Chemotaxis Signaling Complex
Source: PLoS Comput Biol. 2016 Feb 4;12(2):e1004723. doi: 10.1371/journal.pcbi.1004723 (PMC4742279; doi:10.1371/journal.pcbi.1004723)

S8 Fig. Complete phylogenetic profile of COGs of MCPs in comparison with CheW in genomes from Enterobacteriaceae. The organism list is ordered by the ribosomal 16S tree (left). The COGs of MCPs are ordered by the largest COG, containing the highest number of genes, to the smallest COG from left to right (Right panel). The color code represent the number of genes from a specific COG present in a given genome.

.

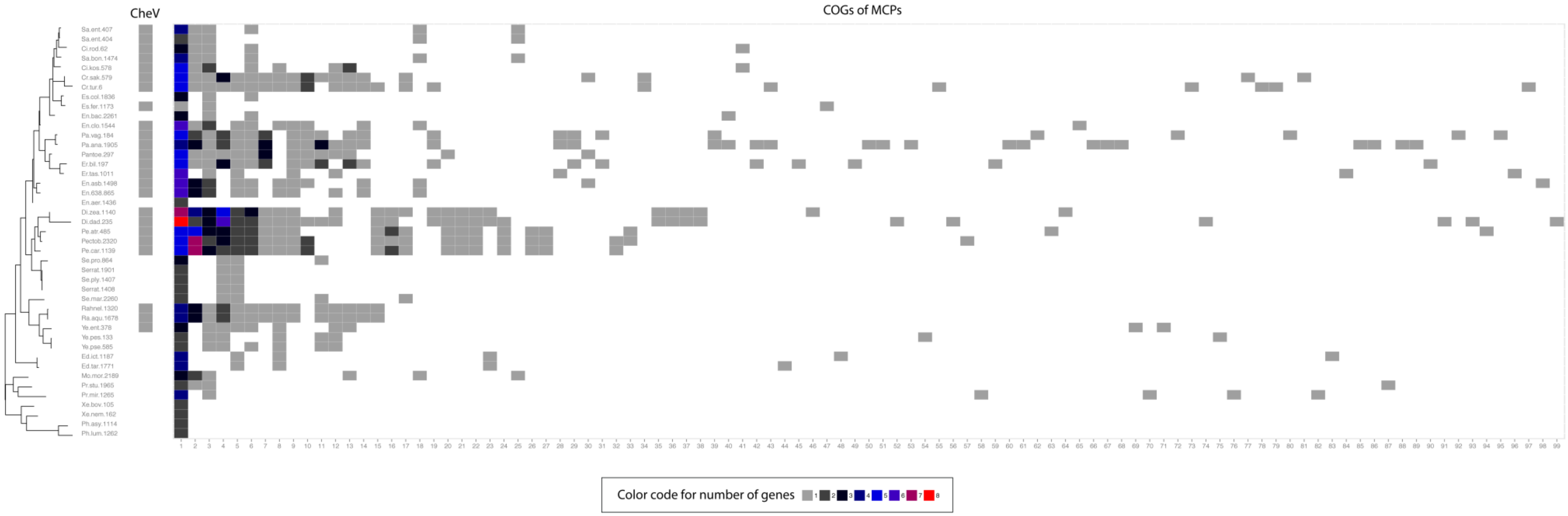

Supplement: S8 Fig — The organism list is ordered by the ribosomal 16S tree (left). The COGs of MCPs are ordered by the largest COG, containing the highest number of genes, to the smallest COG from left to right (Right panel). The color code represents the number of genes from a specific COG present in a given genome. (PDF) [file pcbi.1004723.s010.pdf]
