# Supplementary material for: Evolutionary Genomics Suggests That CheV Is an Additional Adaptor for Accommodating Specific Chemoreceptors within the Chemotaxis Signaling Complex
Source: PLoS Comput Biol. 2016 Feb 4;12(2):e1004723. doi: 10.1371/journal.pcbi.1004723 (PMC4742279; doi:10.1371/journal.pcbi.1004723)
Supplement: S9 Fig — Each sequence tag contains the first two letters of the genus, the first three letters of the species and the organism id in the MiST database, followed by the locus and accession number. The tag also includes the heptad class (e.g. 36H) and the COG to which a given sequence belongs. (PDF) [file pcbi.1004723.s011.pdf]

S9 Fig. Multiple sequence alignment of chemoreceptors from COG1, COG2 and COG6. Each sequence tag contains the first two letters of the genus, the first three letters of the species and the organism id in the MIST database, followed by the locus and accession number. The tag also includes the heptad class (e.g. 36H) and the COG to which a given sequence belongs.

```
Pr.mir.1265-PMI1666-YP_002151397.1--36H-COG1/1-568          ------------------------------------------------------------
Di.dad.235-Dda3937_02779-YP_003883640.1--36H-COG1/1-566     ------------------------------------------------------------
Di.zea.1140-Dd1591_1542-YP_003003875.1--36H-COG1/1-566      ------------------------------------------------------------
Pe.atr.485-ECA1691-YP_049792.1--36H-COG1/1-561              ------------------------------------------------------------
Se.pro.864-Spro_2983-YP_001479212.1--36H-COG1/1-556         ------------------------------------------------------------
Ph.asy.1114-PAU_02685-YP_003041519.1--36H-COG1/1-564        ------------------------------------------------------------
Pr.stu.1965-S70_18135-YP_006218124.1--36H-COG1/1-561        ------------------------------------------------------------
Mo.mor.2189-MU9_1762-YP_007505181.1--36H-COG1/1-561         ------------------------------------------------------------
Pe.car.1139-PC1_2609-YP_003018175.1--36H-COG1/1-561         ------------------------------------------------------------
Xe.nem.162-XNC1_1620-YP_003711881.1--36H-COG1/1-567         ------------------------------------------------------------
Pectob.2320-W5S_1768-YP_006282731.1--36H-COG1/1-561         ------------------------------------------------------------
Pa.vag.184-Pvag_1723-YP_003931360.1--36H-COG1/1-558         ------------------------------------------------------------
Ye.pes.133-YPZ3_2024-YP_003568196.1--36H-COG1/1-557         ------------------------------------------------------------
Ye.pse.585-YPTB2401-YP_070915.1--36H-COG1/1-557             ------------------------------------------------------------
Ye.ent.378-YE2575-YP_001006778.1--36H-COG1/1-557            ------------------------------------------------------------
Pa.ana.1905-PAJ_1540-YP_005934416.1--36H-COG1/1-563         ---------------------------------------------------------MNE
Ph.lum.1262-plu1853-NP_929126.1--36H-COG1/1-564             ------------------------------------------------------------
Serrat.1901-SerAS13_3075-YP_006025914.1--36H-COG1/1-555     ------------------------------------------------------------
Sa.ent.404-STY2128-NP_456485.1--36H-COG1/1-553              ------------------------------------------------------------
Ra.aqu.1678-Q7S_08960-YP_005401597.1--36H-COG1/1-564        ------------------------------------------------------------
Se.mar.2260-D781_2759-YP_007345195.1--36H-COG1/1-555        ------------------------------------------------------------
Rahnel.1320-Rahaq_1830-YP_004212575.1--36H-COG1/1-564       ------------------------------------------------------------
Se.ply.1407-SerAS9_3072-YP_004506452.1--36H-COG1/1-555      ------------------------------------------------------------
Ed.ict.1187-NT01EI_1461-YP_002932882.2--36H-COG1/1-555      ------------------------------------------------------------
En.aer.1436-EAE_15535-YP_004593297.1--36H-COG1/1-556        ------------------------------------------------------------
Xe.bov.105-XBJ1_1923-YP_003467827.1--36H-COG1/1-567         ------------------------------------------------------------
Serrat.1408-SerAS12_3073-YP_004501499.1--36H-COG1/1-555     ------------------------------------------------------------
Er.bil.197-EbC_25350-YP_003741913.1--36H-COG1/1-559         ------------------------------------------------------------
Er.tas.1011-ETA_14650-YP_001907404.1--36H-COG1/1-554        ------------------------------------------------------------
Sa.ent.407-STM4533-NP_463392.1--36H-COG1/1-553              ------------------------------------------------------------
Es.col.1836-Y75_p4240-YP_492486.1--36H-COG1/1-551           ------------------------------------------------------------
En.bac.2261-D782_3913-YP_007342002.1--36H-COG1/1-553        ------------------------------------------------------------
Ci.kos.578-CKO_03442-YP_001454958.1--36H-COG1/1-554         ------------------------------------------------------------
Cr.sak.579-ESA_03402-YP_001439456.1--36H-COG1/1-555         ------------------------------------------------------------
Pantoe.297-Pat9b_1606-YP_004115479.1--36H-COG1/1-558        ------------------------------------------------------------
Cr.tur.6-CTU_05670-YP_003208930.1--36H-COG1/1-555           ------------------------------------------------------------
Sa.bon.1474-SBG_3944-YP_004732724.1--36H-COG1/1-553         ------------------------------------------------------------
En.asb.1498-Entas_0547-YP_004827084.1--36H-COG1/1-554       ------------------------------------------------------------
Ci.rod.62-ROD_48461-YP_003368233.1--36H-COG1/1-554          ------------------------------------------------------------
Ed.tar.1771-ETAF_1249-YP_005698855.1--36H-COG1/1-529        ------------------------------------------------------------
En.638.865-Ent638_0513-YP_001175251.1--36H-COG1/1-586       -------------MTC---------------SHANH--QHDFLKVFTIQAENSVICLMER
Ed.ict.1187-NT01EI_1462-YP_002932883.1--36H-COG1/1-532      ------------------------------------------------------------
Ye.ent.378-YE2573-YP_001006777.1--36H-COG1/1-545            ------------------------------------------------------------
Ed.tar.1771-ETAF_1250-YP_005698856.1--36H-COG1/1-530        ------------------------------------------------------------
Mo.mor.2189-MU9_1763-YP_007505182.1--36H-COG1/1-524         ------------------------------------------------------------
En.aer.1436-EAE_15540-YP_004593298.1--36H-COG1/1-536        ------------------------------------------------------------
En.638.865-Ent638_2456-YP_001177176.1--36H-COG1/1-533       ------------------------------------------------------------
Se.pro.864-Spro_2982-YP_001479211.1--36H-COG1/1-541         ------------------------------------------------------------
Es.col.1836-Y75_p1861-YP_490147.1--36H-COG1/1-533           ------------------------------------------------------------
Serrat.1901-SerAS13_3074-YP_006025913.1--36H-COG1/1-541     ------------------------------------------------------------
Ye.pes.133-YPZ3_2023-YP_003568195.1--36H-COG1/1-536         ------------------------------------------------------------
Serrat.1408-SerAS12_3072-YP_004501498.1--36H-COG1/1-541     ------------------------------------------------------------
Se.mar.2260-D781_2758-YP_007345194.1--36H-COG1/1-534        ------------------------------------------------------------
Ye.pse.585-YPTB2400-YP_070914.1--36H-COG1/1-536             ------------------------------------------------------------
Se.ply.1407-SerAS9_3071-YP_004506451.1--36H-COG1/1-541      ------------------------------------------------------------
Pr.stu.1965-S70_18140-YP_006218125.1--36H-COG1/1-521        ------------------------------------------------------------
En.bac.2261-D782_1749-YP_007339930.1--36H-COG1/1-533        ------------------------------------------------------------
En.asb.1498-Entas_2590-YP_004829104.1--36H-COG1/1-533       ------------------------------------------------------------
En.asb.1498-Entas_3672-YP_004830170.1--36H-COG1/1-545       ------------------------------------------------------------
Ci.kos.578-CKO_01067-YP_001452646.1--36H-COG1/1-537         ----------------------------------------------------------MG
En.clo.1544-EcWSU1_02810-YP_004952663.1--36H-COG1/1-538     -------------M--------------------------------------------MG
Sa.ent.407-STM3152-NP_462067.1--36H-COG1/1-547              ------------------------------------------------------------
Pe.atr.485-ECA1332-YP_049438.1--36H-COG1/1-555              ------------------------------------------------------------
Pe.car.1139-PC1_1208-YP_003016792.1--36H-COG1/1-555         ------------------------------------------------------------
En.638.865-Ent638_3407-YP_001178118.1--36H-COG1/1-545       ------------------------------------------------------------
Pa.vag.184-Pvag_1413-YP_003931052.1--36H-COG1/1-554         ------------------------------------------------------------
Ph.asy.1114-PAU_02684-YP_003041518.1--36H-COG1/1-544        ------------------------------------------------------------
Pa.ana.1905-PAJ_1316-YP_005934192.1--36H-COG1/1-556         ------------------------------------------------------------
Ci.kos.578-CKO_04394-YP_001455885.1--36H-COG1/1-561         -------------MSP--------------------------------DGESLKYGKEIL
Sa.bon.1474-SBG_2750-YP_004731563.1--36H-COG1/1-547         ------------------------------------------------------------
Ra.aqu.1678-Q7S_16025-YP_005402985.1--36H-COG1/1-554        ------------------------------------------------------------
Rahnel.1320-Rahaq_3177-YP_004213898.1--36H-COG1/1-554       ------------------------------------------------------------
Pectob.2320-W5S_3114-YP_006284066.1--36H-COG1/1-555         ------------------------------------------------------------
```

```
Ye.ent.378-YE2971-YP_001007160.1--36H-COG1/1-552          ------------------------------------------------------------
Er.bil.197-EbC_20190-YP_003741400.1--36H-COG1/1-556       ------------------------------------------------------------
Ph.lum.1262-plu1854-NP_929127.1--36H-COG1/1-544           ------------------------------------------------------------
Er.bil.197-EbC_25340-YP_003741912.1--36H-COG1/1-528       ------------------------------------------------------------
Di.zea.1140-Dd1591_2937-YP_003005238.1--36H-COG1/1-564    ------------------------------------------------------------
Er.tas.1011-ETA_14670-YP_001907406.1--36H-COG1/1-518      ------------------------------------------------------------
Sa.ent.404-STY4234-NP_458344.1--36H-COG1/1-547            ------------------------------------------------------------
Sa.ent.407-STM3577-NP_462478.1--36H-COG1/1-547            ------------------------------------------------------------
Sa.bon.1474-SBG_3170-YP_004731982.1--36H-COG1/1-547       ------------------------------------------------------------
En.asb.1498-Entas_2591-YP_004829105.1--36H-COG1/1-555     ------------------------------------------------------------
En.638.865-Ent638_1858-YP_001176586.1--36H-COG1/1-552     ------------------------------------------------------------
Ci.rod.62-ROD_19331-YP_003365490.1--36H-COG1/1-553        ------------------------------------------------------------
Di.dad.235-Dda3937_01559-YP_003882087.1--36H-COG1/1-566   --------------------------------------------------------MEST
Er.bil.197-EbC_37780-YP_003743156.1--36H-COG1/1-553       ------------------------------------------------------------
Pantoe.297-Pat9b_2001-YP_004115872.1--36H-COG1/1-558      ------------------------------------------------------------
En.clo.1544-EcWSU1_01962-YP_004951821.1--36H-COG1/1-553   ------------------------------------------------------------
En.asb.1498-Entas_1879-YP_004828403.1--36H-COG1/1-554     ------------------------------------------------------------
En.bac.2261-D782_1748-YP_007339929.1--36H-COG1/1-547      ------------------------------------------------------------
Es.col.1836-Y75_p1862-YP_490148.1--36H-COG1/1-553         ------------------------------------------------------------
Cr.tur.6-CTU_33010-YP_003211664.1--36H-COG1/1-555         ------------------------------------------------------------
Cr.tur.6-CTU_25780-YP_003210941.1--36H-COG1/1-536         ------------------------------------------------------------
En.clo.1544-EcWSU1_00403-YP_004950264.1--36H-COG1/1-549   ------------------------------------------------------------
En.638.865-Ent638_2457-YP_001177177.1--36H-COG1/1-555     ------------------------------------------------------------
Sa.bon.1474-SBG_1755-YP_004730609.1--36H-COG1/1-553       ------------------------------------------------------------
Pr.mir.1265-PMI1665-YP_002151396.1--36H-COG1/1-548        ------------------------------------------------------------
Cr.sak.579-ESA_01348-YP_001437444.1--36H-COG1/1-556       ------------------------------------------------------------
Cr.sak.579-ESA_00560-YP_001436686.1--36H-COG1/1-555       ------------------------------------------------------------
Sa.ent.407-STM1919-NP_460876.1--36H-COG1/1-553            ------------------------------------------------------------
Ci.kos.578-CKO_01066-YP_001452645.1--36H-COG1/1-552       ------------------------------------------------------------
En.asb.1498-Entas_0424-YP_004826963.1--36H-COG1/1-549     ------------------------------------------------------------
Pantoe.297-Pat9b_1273-YP_004115150.1--36H-COG1/1-553      ------------------------------------------------------------
Ed.tar.1771-ETAF_2264-YP_005699859.1--36H-COG1/1-555      ------------------------------------------------------------
Pa.ana.1905-PAJ_3534-YP_005936409.1--36H-COG1/1-553       ------------------------------------------------------------
Pa.vag.184-Pvag_1203-YP_003930842.1--36H-COG1/1-552       ------------------------------------------------------------
Pantoe.297-Pat9b_1605-YP_004115478.1--36H-COG1/1-556      ------------------------------------------------------------
Cr.sak.579-ESA_00210-YP_001436348.1--36H-COG1/1-557       ------------------------------------------------------------
Er.tas.1011-ETA_pET460340-YP_001905943.1--36H-COG1/1-543 ------------------------------------------------------------
Cr.tur.6-CTU_36610-YP_003212024.1--36H-COG1/1-557         ------------------------------------------------------------
Pe.car.1139-PC1_1159-YP_003016743.1--36H-COG1/1-556       ------------------------------------------------------------
Er.tas.1011-ETA_14640-YP_001907403.1--36H-COG1/1-554      ------------------------------------------------------------
Pa.vag.184-Pvag_1725-YP_003931362.1--36H-COG1/1-556       ------------------------------------------------------------
Ed.ict.1187-NT01EI_3742-YP_002935104.1--36H-COG1/1-525    ------------------------------------------------------------
En.638.865-Ent638_0380-YP_001175119.1--36H-COG1/1-549     ------------------------------------------------------------
Er.bil.197-EbC_25360-YP_003741914.1--36H-COG1/1-556       ------------------------------------------------------------
Er.tas.1011-ETA_16910-YP_001907630.1--36H-COG1/1-543      ------------------------------------------------------------
Es.fer.1173-EFER_3055-YP_002384154.1--36H-COG1/1-603      -------------MYHKQPRQSVTSLKTQADSDKIRLLNVPKHKVFSSQADNQSICLVER
Xe.nem.162-XNC1_1619-YP_003711880.1--36H-COG1/1-520       ------------------------------------------------------------
En.clo.1544-EcWSU1_02811-YP_004952664.1--36H-COG1/1-559   ---------------------------------------------------------MRR
Di.dad.235-Dda3937_03500-YP_003882750.1--36H-COG1/1-553   ------------------------------------------------------------
Xe.bov.105-XBJ1_1924-YP_003467828.1--36H-COG1/1-523       ------------------------------------------------------------
Rahnel.1320-Rahaq_1831-YP_004212576.1--36H-COG1/1-556     ------------------------------------------------------------
Ra.aqu.1678-Q7S_08965-YP_005401598.1--36H-COG1/1-556      ------------------------------------------------------------
Cr.sak.579-ESA_01126-YP_001437230.1--36H-COG1/1-514       ------------------------------------------------------------
Pe.atr.485-ECA2712-YP_050803.1--36H-COG1/1-560            ------------------------------------------------------------
En.clo.1544-EcWSU1_00560-YP_004950421.1--36H-COG1/1-597   -------------MNWG------TGISQRAKKSKNHKFKDDFRKVFPFQAENPVICLMES
Di.dad.235-Dda3937_03498-YP_003882752.1--36H-COG1/1-561   ------------------------------------------------------------
Cr.tur.6-CTU_27880-YP_003211151.1--36H-COG1/1-514         ------------------------------------------------------------
Pe.car.1139-PC1_1669-YP_003017246.1--36H-COG1/1-560       ------------------------------------------------------------
Ra.aqu.1678-Q7S_14450-YP_005402672.1--36H-COG1/1-551      ------------------------------------------------------------
Ci.kos.578-CKO_03622-YP_001455137.1--36H-COG1/1-517       ------------------------------------------------------------
Di.zea.1140-Dd1591_2408-YP_003004728.1--36H-COG1/1-557    ------------------------------------------------------------
Di.zea.1140-Dd1591_2406-YP_003004726.1--36H-COG1/1-559    ------------------------------------------------------------
Ed.ict.1187-NT01EI_2801-YP_002934203.1--36H-COG1/1-554    ------------------------------------------------------------
Pe.car.1139-PC1_1384-YP_003016966.1--36H-COG1/1-559       ------------------------------------------------------------
Rahnel.1320-Rahaq_2866-YP_004213595.1--36H-COG1/1-551     ------------------------------------------------------------
Pe.atr.485-ECA1281-YP_049387.1--36H-COG1/1-556            ------------------------------------------------------------
Pectob.2320-W5S_1886-YP_006282848.1--36H-COG1/1-549       ------------------------------------------------------------
Pectob.2320-W5S_1696-YP_006282659.1--36H-COG1/1-559       ------------------------------------------------------------
Di.zea.1140-Dd1591_2407-YP_003004727.1--36H-COG1/1-555    ------------------------------------------------------------
Pe.atr.485-ECA1509-YP_049611.1--36H-COG1/1-559            ------------------------------------------------------------
Pa.vag.184-Pvag_1724-YP_003931361.1--36H-COG1/1-520       ------------------------------------------------------------
Pa.ana.1905-PAJ_1541-YP_005934417.1--36H-COG1/1-520       ------------------------------------------------------------
Mo.mor.2189-MU9_1596-YP_007505015.1--36H-COG1/1-519       ------------------------------------------------------------
Pr.mir.1265-PMI2808-YP_002152509.1--36H-COG1/1-563        ------------------------------------------------------------
Di.zea.1140-Dd1591_0395-YP_003002764.1--36H-COG1/1-556    ------------------------------------------------------------
En.clo.1544-EcWSU1_03786-YP_004953635.1--36H-COG1/1-515   ------------------------------------------------------------
Pectob.2320-W5S_3170-YP_006284116.1--36H-COG1/1-556       ------------------------------------------------------------
Di.dad.235-Dda3937_02184-YP_003884754.1--36H-COG1/1-556   ------------------------------------------------------------
Ci.rod.62-ROD_32641-YP_003366748.1--36H-COG1/1-517        ------------------------------------------------------------
Di.dad.235-Dda3937_03499-YP_003882751.1--36H-COG1/1-561   -------------MI---------------------------------------NCPEPV
Di.dad.235-Dda3937_00105-YP_003883346.1--36H-COG1/1-561   ------------------------------------------------------------
Pantoe.297-Pat9b_1604-YP_004115477.1--36H-COG1/1-555      ------------------------------------------------------------
Er.tas.1011-ETA_14660-YP_001907405.1--36H-COG1/1-560      ------------------------------------------------------------
Pr.mir.1265-PMI2809-YP_002152510.1--36H-COG1/1-575        ------------------------------------------------------------
Ed.tar.1771-ETAF_3053-YP_005700648.1--36H-COG1/1-516      ------------------------------------------------------------
Se.pro.864-Spro_1415-YP_001477647.1--36H-COG1/1-546       ------------------------------------------------------------
Di.zea.1140-Dd1591_1804-YP_003004134.1--36H-COG1/1-562    ------------------------------------------------------------
Di.dad.235-Dda3937_03501-YP_003882749.1--36H-COG1/1-575   -------------MTMINS-----------------------------RAMHAENDSEPV
```

```
Pantoe.297-Pat9b_0851-YP_004114731.1--36H-COG2/1-524      ------------------------------------------------------------
Pa.vag.184-Pvag_0291-YP_003929953.1--36H-COG2/1-525       ------------------------------------------------------------
En.clo.1544-EcWSU1_03886-YP_004953735.1--36H-COG2/1-519   ------------------------------------------------------------
Cr.tur.6-CTU_04790-YP_003208842.1--36H-COG2/1-515         ------------------------------------------------------------
Ci.kos.578-CKO_04484-YP_001455975.1--36H-COG2/1-521       ------------------------------------------------------------
Cr.sak.579-ESA_03488-YP_001439539.1--36H-COG2/1-515       ------------------------------------------------------------
Ci.rod.62-ROD_48071-YP_003368199.1--36H-COG2/1-521        ------------------------------------------------------------
Sa.bon.1474-SBG_2851-YP_004731664.1--36H-COG2/1-521       ------------------------------------------------------------
Sa.ent.407-STM3216-NP_462130.1--36H-COG2/1-521            ------------------------------------------------------------
En.asb.1498-Entas_3753-YP_004830248.1--36H-COG2/1-519     ------------------------------------------------------------
Mo.mor.2189-MU9_3389-YP_007506806.1--36H-COG2/1-521       ------------------------------------------------------------
Sa.ent.404-STY3394-NP_457606.1--36H-COG2/1-522            ------------------------------------------------------------
En.638.865-Ent638_3526-YP_001178235.1--36H-COG2/1-519     ------------------------------------------------------------
Pe.atr.485-ECA1774-YP_049874.1--36H-COG2/1-554            ------------------------------------------------------------
En.638.865-Ent638_2119-YP_001176845.1--36H-COG2/1-549     ------------------------------------------------------------
Pectob.2320-W5S_2813-YP_006283770.1--36H-COG2/1-554       ------------------------------------------------------------
Di.zea.1140-Dd1591_0695-YP_003003055.1--36H-COG2/1-561    ------------------------------------------------------------
Pa.vag.184-Pvag_pPag30079-YP_003729820.1--36H-COG2/1-546 ------------------------------------------------------------
Er.bil.197-EbC_38940-YP_003743272.1--36H-COG2/1-526       ------------------------------------------------------------
Ra.aqu.1678-Q7S_24891-YP_005419232.1--36H-COG2/1-554      -------------M----------------------------------------------
En.asb.1498-Entas_2287-YP_004828805.1--36H-COG2/1-513     -------------M-------------------------------------------FSI
Rahnel.1320-Rahaq_4869-YP_004215574.1--36H-COG2/1-554     -------------M----------------------------------------------
Di.dad.235-Dda3937_00027-YP_003883574.1--36H-COG2/1-554   ------------------------------------------------------------
Pr.stu.1965-S70_05790-YP_006215722.1--36H-COG2/1-520      ------------------------------------------------------------
En.638.865-Ent638_2100-YP_001176826.1--36H-COG2/1-512     ---------------------------------------------------------MSF
Pe.car.1139-PC1_2526-YP_003018093.1--36H-COG2/1-554       ------------------------------------------------------------
Mo.mor.2189-MU9_702-YP_007504121.1--36H-COG2/1-525        ------------------------------------------------------------
Pe.car.1139-PC1_3443-YP_003018995.1--36H-COG2/1-511       -------------MLR--------------------------------------------
Pa.ana.1905-PAJ_0915-YP_005933791.1--36H-COG2/1-559       -------------MLTPTP-----------------------------------------
Pectob.2320-W5S_3736-YP_006284671.1--36H-COG2/1-510       -------------MLR--------------------------------------------
Pe.atr.485-ECA0183-YP_048310.1--36H-COG2/1-553            ------------------------------------------------------------
Rahnel.1320-Rahaq_3107-YP_004213828.1--36H-COG2/1-503     -------------MNV----------------------------------------LSGI
Pe.car.1139-PC1_4071-YP_003019622.1--36H-COG2/1-553       ------------------------------------------------------------
Di.dad.235-Dda3937_03462-YP_003884507.1--36H-COG2/1-563   ------------------------------------------------------------
Ra.aqu.1678-Q7S_15665-YP_005402913.1--36H-COG2/1-503      -------------MNV----------------------------------------LSGI
Pectob.2320-W5S_4505-YP_006285423.1--36H-COG2/1-555       ------------------------------------------------------------
Pe.atr.485-ECA3642-YP_051730.1--36H-COG2/1-551            ------------------------------------------------------------
Pe.car.1139-PC1_1417-YP_003016999.1--36H-COG2/1-554       ------------------------------------------------------------
En.asb.1498-Entas_4509-YP_004821569.1--36H-COG2/1-559     -------------MDFL--------------------------------LQKWNC-----
Pa.ana.1905-PAJ_3064-YP_005935940.1--36H-COG2/1-544       ------------------------------------------------------------
Pectob.2320-W5S_1733-YP_006282696.1--36H-COG2/1-535       ------------------------------------------------------------
Pe.atr.485-ECA4334-YP_052421.1--36H-COG2/1-556            ------------------------------------------------------------
Di.zea.1140-Dd1591_1602-YP_003003935.1--36H-COG2/1-554    ------------------------------------------------------------
Pectob.2320-W5S_4506-YP_006285424.1--36H-COG2/1-540       ------------------------------------------------------------
Di.zea.1140-Dd1591_1453-YP_003003794.1--36H-COG2/1-532    ------------------------------------------------------------
Pectob.2320-W5S_0106-YP_006281117.1--36H-COG2/1-556       ------------------------------------------------------------
Di.zea.1140-Dd1591_0769-YP_003003127.1--36H-COG2/1-505    -------------MLH--------------------------------------------
Ra.aqu.1678-Q7S_18035-YP_005403387.1--36H-COG2/1-535      ------------------------------------------------------------
Rahnel.1320-Rahaq_3579-YP_004214298.1--36H-COG2/1-535     ------------------------------------------------------------
Pe.car.1139-PC1_4072-YP_003019623.1--36H-COG2/1-540       ------------------------------------------------------------
Pe.car.1139-PC1_3464-YP_003019016.1--36H-COG2/1-551       ------------------------------------------------------------
Pe.car.1139-PC1_0111-YP_003015709.1--36H-COG2/1-556       ------------------------------------------------------------
Pe.atr.485-ECA0182-YP_048309.1--36H-COG2/1-542            ------------------------------------------------------------
Pectob.2320-W5S_3753-YP_006284688.1--36H-COG2/1-551       ------------------------------------------------------------
Pa.ana.1905-PAJ_2666-YP_005935542.1--36H-COG2/1-514       ------------------------------------------------------------
Ci.rod.62-ROD_16161-YP_003365194.1--36H-COG6/1-557        -------------MD-----------------------------------NTSSMQAPAK
En.asb.1498-Entas_2095-YP_004828614.1--36H-COG6/1-563     -------------MD-----------------------------------NTTSMQAQHK
En.638.865-Ent638_1961-YP_001176688.1--36H-COG6/1-563     -------------MD-----------------------------------NTKKMRSPQT
Cr.sak.579-ESA_01710-YP_001437800.1--36H-COG6/1-565       -------------MN-----------------------------------KTTTRDAGA
Cr.tur.6-CTU_22440-YP_003210607.1--36H-COG6/1-565         -------------MK-----------------------------------KTTTRDSGA
Ci.kos.578-CKO_01456-YP_001453025.1--36H-COG6/1-562       -------------MD-----------------------------------NTSTMQAPHK
Rahnel.1320-Rahaq_1930-YP_004212674.1--36H-COG6/1-566     --------------------------------------------------MDTPTQLTTR
Pantoe.297-Pat9b_2403-YP_004116261.1--36H-COG6/1-564      -------------MK------------------------------------TASIDEQNK
Er.bil.197-EbC_26700-YP_003742048.1--36H-COG6/1-567       -------------MK-----------------------------------SSHLNAEQNG
Pectob.2320-W5S_1760-YP_006282723.1--36H-COG6/1-570       -------------MK-----------------------------------ITSRSEQHIK
Pa.ana.1905-PAJ_1621-YP_005934497.1--36H-COG6/1-568       -------------MN-----------------------------------TTLVNEGHSK
En.clo.1544-EcWSU1_02120-YP_004951978.1--36H-COG6/1-576  MFLRILPTRESTVMD-----------------------------------NTTTMQAQRT
Ra.aqu.1678-Q7S_09815-YP_005401764.1--36H-COG6/1-566      --------------------------------------------------MDTPTQLTTR
Ye.pse.585-YPTB2412-YP_070925.1--36H-COG6/1-579           --MKRGSAHKPLDMK-----------------------------------IAPEPNKDTK
Ye.ent.378-YE2588-YP_001006789.1--36H-COG6/1-579          --MKRGSTHKPLDIK-----------------------------------ITPEHHKDTK
Di.dad.235-Dda3937_02787-YP_003883649.1--36H-COG6/1-572   -------------MK-----------------------------------MMSKSEQQGK
Pa.vag.184-Pvag_1800-YP_003931435.1--36H-COG6/1-568       -------------MN-----------------------------------TALDNDGQKS
En.bac.2261-D782_2307-YP_007340470.1--36H-COG6/1-558      -------------MD-----------------------------------KTKTQHQTQK
Pe.atr.485-ECA1683-YP_049784.1--36H-COG6/1-573            -----------MDMK-----------------------------------ITSRSEQHAE
Pe.car.1139-PC1_2617-YP_003018183.1--36H-COG6/1-573       -----------MDMK-----------------------------------ITSRSEQHVE
Di.zea.1140-Dd1591_1535-YP_003003868.1--36H-COG6/1-574    -------------MK-----------------------------------MMSKSEQQGK
Di.zea.1140-Dd1591_3580-YP_003005868.1--36H-COG6/1-572    -------------MH-----------------------------------TTALPSSSSG
Es.col.1836-Y75_p1397-YP_489687.1--36H-COG6/1-546         -------------MN---------------------------------------TTPSQR
Pe.atr.485-ECA3902-YP_051990.1--36H-COG6/1-575            M-M--------VKMR-----------------------------------MQSMSVQKEK
Sa.bon.1474-SBG_1454-YP_004730321.1--36H-COG6/1-537       -------------MG-----------------------------------NTLSIQASHK
Sa.ent.407-STM1626-NP_460585.1--36H-COG6/1-541            -------------MG-----------------------------------NTFSMQASHK
Pe.car.1139-PC1_3679-YP_003019230.1--36H-COG6/1-567       -----------------------------------------------------MSIQKEK
Pectob.2320-W5S_4020-YP_006284955.1--36H-COG6/1-575       --M--------VKMT-----------------------------------MQSMSVQKEK
Di.zea.1140-Dd1591_0523-YP_003002884.1--36H-COG6/1-568    -----------------------------------------------------MAEQKGN
Di.dad.235-Dda3937_02665-YP_003881279.1--36H-COG6/1-572   -------------MN-----------------------------------TTAQSPSSPG
```

```
Pr.mir.1265-PMI1666-YP_002151397.1--36H-COG1/1-568         --MF-SRMKIVTGLLSILLLFGVLQFISGGVFYSNLSETRQDLDKTLTIQKQRERLDESW
Di.dad.235-Dda3937_02779-YP_003883640.1--36H-COG1/1-566    --ML-NRVKVVTGLVIVLGLFIALQIISGGLFFNALKSDRDIFTTTRIINQQKSELESTW
Di.zea.1140-Dd1591_1542-YP_003003875.1--36H-COG1/1-566     --ML-NRVKVVTGLVIILGLFIALQVISGGLFFNALKSDRDIFTTTRIINQQKSELESTW
Pe.atr.485-ECA1691-YP_049792.1--36H-COG1/1-561             --MF-NRIKVVTGLMLVLVLFGALQLISGGLFFSALKNDKDIFSSTQVINQKRSELDSAW
Se.pro.864-Spro_2983-YP_001479212.1--36H-COG1/1-556        --MF-NRMKVVTSLLLVLVLFGALQLVSGGLFFSSLKNDKENFTVLQTIRQQQSELNESW
Ph.asy.1114-PAU_02685-YP_003041519.1--36H-COG1/1-564       --MF-NRMKIVTGLMAVIILFGALQFISGGLFFHELKSNKENLEILDKMREQQTFLNDTW
Pr.stu.1965-S70_18135-YP_006218124.1--36H-COG1/1-561       --MH-SRIKIVSGLLSVIVIFILLQLISSGLMFNKLGDNSDSIKYLNSLQRQRQVLTESW
Mo.mor.2189-MU9_1762-YP_007505181.1--36H-COG1/1-561        --MY-SRIKIVSGLLSVILIFVLLQLASSGLMFSKLGENRDSIHFLDSLQSQRQVLTQSW
Pe.car.1139-PC1_2609-YP_003018175.1--36H-COG1/1-561        --MF-NRIKVVTGLMLVLVLFGALQLISGGLFFSALKNDKDIFSSTQIINQKRSELDSAW
Xe.nem.162-XNC1_1620-YP_003711881.1--36H-COG1/1-567        --MF-NRMKIVTGLISVIILFGVLQFISGGLFFNGLKNDTEAFDLTDKMREQQKLLDESW
Pectob.2320-W5S_1768-YP_006282731.1--36H-COG1/1-561        --MF-NRIKVVTGLMLVLVLFGALQLISGGLFFSALKNDKDIFSSTQVINQKRSELDSAW
Pa.vag.184-Pvag_1723-YP_003931360.1--36H-COG1/1-558        --MF-SRVRVVSGLLCVLALFALLQLFSGGMFFKTVSSDKENFAYNQRLNTLQQAMGTSW
Ye.pes.133-YPZ3_2024-YP_003568196.1--36H-COG1/1-557        --MF-KRMKVVTSLLLVLVLFGALQLVSGGLFFKSLQNDQENFNVLQVIRQQQSVLNESW
Ye.pse.585-YPTB2401-YP_070915.1--36H-COG1/1-557            --MF-KRMKVVTSLLLVLVLFGALQLVSGGLFFKSLQNDQENFNVLQVIRQQQSVLNESW
Ye.ent.378-YE2575-YP_001006778.1--36H-COG1/1-557           --MF-KRMKVVTSLLLVLVLFGALQLVSGGLFFNSLKNDKENFAVLQVIRQQQSVLNESW
Pa.ana.1905-PAJ_1540-YP_005934416.1--36H-COG1/1-563        VSMF-SRVRVVSGLLCVLALFALLQIFSGGVFYKTVTEDKENFAYNQRLSTLQRAMGTSW
Ph.lum.1262-plu1853-NP_929126.1--36H-COG1/1-564            --MF-NRMKIVTGLMAVIILFGALQFISGGLFFHELKSNKENLEILDKMREQQTFLNDTW
Serrat.1901-SerAS13_3075-YP_006025914.1--36H-COG1/1-555    --MF-NRMKVVTSLLLVLVLFGALQLVSGGLFFSSLKNDKENFTVLQTIRQQQSDLNESW
Sa.ent.404-STY2128-NP_456485.1--36H-COG1/1-553             --MF-NRIRVVTMLMMVLGVFALLQLVSGGLLFSSLQHNQQGFVISNELRQQQSELTSTW
Ra.aqu.1678-Q7S_08960-YP_005401597.1--36H-COG1/1-564       --MF-NRMKVVTGIVILLVVFGALQLVSGGLFFKSLKTDKDNFTTSQQIRLQQAEFNASW
Se.mar.2260-D781_2759-YP_007345195.1--36H-COG1/1-555       --MF-NRMKVVTSLLLVLVLFGALQLISGGLFFSSLKSDKENFTVLQTIRQQQSELNGSW
Rahnel.1320-Rahaq_1830-YP_004212575.1--36H-COG1/1-564      --MF-NRMKVVTGIVILLVVFGALQLVSGGLFFKSLKTDKDNFTTSQQIRLQQAEFNASW
Se.ply.1407-SerAS9_3072-YP_004506452.1--36H-COG1/1-555     --MF-NRMKVVTSLLLVLVLFGALQLVSGGLFFSSLKNDKENFTVLQTIRQQQSDLNESW
Ed.ict.1187-NT01EI_1461-YP_002932882.2--36H-COG1/1-555     --ML-NRIKVVTSLLLVLGLFGILQMTSGGVFFDALKSDKESMTVLQTIRQQQAALNASW
En.aer.1436-EAE_15535-YP_004593297.1--36H-COG1/1-556       --MF-NRIKVVTSLLLVLVLFGALQLISGGLFFSSLKSDKENFTVLQTIRQQQLLLSESR
Xe.bov.105-XBJ1_1923-YP_003467827.1--36H-COG1/1-567        --MF-NRMKIVTGLISVIILFGALQFISGGLFFNGLKNDTEAFDLLDKMRQQQKLLDESW
Serrat.1408-SerAS12_3073-YP_004501499.1--36H-COG1/1-555    --MF-NRMKVVTSLLLVLVLFGALQLVSGGLFFSSLKNDKENFTVLQTIRQQQSDLNESW
Er.bil.197-EbC_25350-YP_003741913.1--36H-COG1/1-559        --MF-KRIKVVTGLVSVLVLFALLQFGTGGLFFHTVKNDKDNFAFNQRLRALQQSMGTSW
Er.tas.1011-ETA_14650-YP_001907404.1--36H-COG1/1-554       --MF-KRMKVVTSLILVLVFFGSLQLLTGSLFFQALKGDKDNFAISQQLRLQQSALNQSW
Sa.ent.407-STM4533-NP_463392.1--36H-COG1/1-553             --ML-KRIKIVTSLLLVLALFGLLQLTSGGLFFNSLKNDKENFTVLQTIRQQQSALNATW
Es.col.1836-Y75_p4240-YP_492486.1--36H-COG1/1-551          --ML-KRIKIVTSLLLVLAVFGLLQLTSGGLFFNALKNDKENFTVLQTIRQQQSTLNGSW
En.bac.2261-D782_3913-YP_007342002.1--36H-COG1/1-553       --ML-KRIKIVTSLVLVLVLFGLLQLTSGGLFFNALKNDKENFTVLQTVRQQQSGLNGSW
Ci.kos.578-CKO_03442-YP_001454958.1--36H-COG1/1-554        --ML-KRIKIVTSLLLVLAIFGLLQLTSGGLFFNSLKNDKENFTVLQTIRQQQSTLNASW
Cr.sak.579-ESA_03402-YP_001439456.1--36H-COG1/1-555        --ML-NRIKVVTSLLFVLALFGLLQMASGGLFFNAIKSDKENFTVLQTIRQQQSTLNGSW
Pantoe.297-Pat9b_1606-YP_004115479.1--36H-COG1/1-558       --MF-SRIRVVSGLLCVLALFALLQLFSGGMFFQTVKADKDNFAYNQRLSTLQRAMGTSW
Cr.tur.6-CTU_05670-YP_003208930.1--36H-COG1/1-555          --ML-NRIKVVTSLLFVLALFGLLQMASGGLFFNAIKSDKENFTVLQTIRQQQSTLNGSW
Sa.bon.1474-SBG_3944-YP_004732724.1--36H-COG1/1-553        --ML-KRIKIVTSLLLVLALFGLLQLTSGGLFFNSLKNDKENFTVLQTIRQQQSALNATW
En.asb.1498-Entas_0547-YP_004827084.1--36H-COG1/1-554      --ML-NRIKIVTSLLLVLAIFGLLQLTSGGLFFNALKHDKENFTVLQTIRQQQSTLNGSW
Ci.rod.62-ROD_48461-YP_003368233.1--36H-COG1/1-554         --ML-KRIRIVTSLLLVLGLFGLLQLTSGGIFFSALKNDNENFTVLQNIRLQQSTLNDSW
Ed.tar.1771-ETAF_1249-YP_005698855.1--36H-COG1/1-529       ------------------------------MFFDALKSDKESITVLQTIRQQQAALNGSW
En.638.865-Ent638_0513-YP_001175251.1--36H-COG1/1-586      ENML-NRIKIVTSLLLVLAIFGLLQLTSGGLFFNALKHDKENFTVLQTIRQQQSTLNGSW
Ed.ict.1187-NT01EI_1462-YP_002932883.1--36H-COG1/1-532     --MF-NRIRISTSLFVLVLAFCFLQLSSNGVSFMAIRSDSDNYARVQLSNAQRERLGQTW
Ye.ent.378-YE2573-YP_001006777.1--36H-COG1/1-545           --MF-GRIRISTSFFLLLMLICSIQLISSGLSFTAFRSDYQNLNRVDLSSQQRDALSLSW
Ed.tar.1771-ETAF_1250-YP_005698856.1--36H-COG1/1-530       --MF-NRIRISTSLFALVLAFCFLQLSSNGVSFMAIRSDSDNYARVQISNDQRETLGQTW
Mo.mor.2189-MU9_1763-YP_007505182.1--36H-COG1/1-524        --MFGKRIRISVSLYLLLIFFCGMQIVSSGLSLGIVSSELSSLERVDLGSKKRALLVSSY
En.aer.1436-EAE_15540-YP_004593298.1--36H-COG1/1-536       --MF-NRIRISTSLFLLLISFCIMQLISTGLSYVALRADNHNLERITVSSQQRDALSLSW
En.638.865-Ent638_2456-YP_001177176.1--36H-COG1/1-533      --ML-NRIRISTTLFLILILCGVLQVGSNGLSFWAFRDGYQNLQEVETSNQQRSTLAQTR
Se.pro.864-Spro_2982-YP_001479211.1--36H-COG1/1-541        --MF-NRIRVSTSLFLLLMTFCVMQLATSGLSYTAFRSDNHNLNLITLGSQQRDALSLSW
Es.col.1836-Y75_p1861-YP_490147.1--36H-COG1/1-533          --MF-NRIRISTTLFLILILCGILQIGSNGMSFWAFRDDLQRLNQVEQSNQQRAALAQTR
Serrat.1901-SerAS13_3074-YP_006025913.1--36H-COG1/1-541    --MF-NRIRVSTSLFLLLMTFCVMQLATSGLSYTAFRSDHHNLNLITLGSQQRDALSLSW
Ye.pes.133-YPZ3_2023-YP_003568195.1--36H-COG1/1-536        --MF-GQIRISTSFFLLLMFLCAIQLISSGLSFTAFRSDYQNLNRVELSSQQRDALSLSW
Serrat.1408-SerAS12_3072-YP_004501498.1--36H-COG1/1-541    --MF-NRIRVSTSLFLLLMTFCVMQLATSGLSYTAFRSDHHNLNLITLGSQQRDALSLSW
Se.mar.2260-D781_2758-YP_007345194.1--36H-COG1/1-534       --MF-NRVRISISLYLLMFCFCLMQLISTGLSFSAFRADSRSFMAITQGSQQRDVLTMSR
Ye.pse.585-YPTB2400-YP_070914.1--36H-COG1/1-536            --MF-GQIRISTSFFLLLMFLCAIQLISSGLSFTAFRSDYQNLNRVELSSQQRDALSLSW
Se.ply.1407-SerAS9_3071-YP_004506451.1--36H-COG1/1-541     --MF-NRIRVSTSLFLLLMTFCVMQLATSGLSYTAFRSDHHNLNLITLGSQQRDALSLSW
Pr.stu.1965-S70_18140-YP_006218125.1--36H-COG1/1-521       --MFGKSIRISVSLYLLLVMFCGMQIVSSALSLGIVSSELSSLERIDLGSKKRDLLETTY
En.bac.2261-D782_1749-YP_007339930.1--36H-COG1/1-533       --MF-TRIRISTTLFLILIICGLLQIGSNGLSFWAFRDGQQNLEQIEHSGQQLSSLMAAR
En.asb.1498-Entas_2590-YP_004829104.1--36H-COG1/1-533      --ML-NRIRISTTLFLILILCGVLQVGSNGLSFWAFRDGYQNLQEVEASNQQRSALAQTR
En.asb.1498-Entas_3672-YP_004830170.1--36H-COG1/1-545      MQML-RNFTIRFVMLTILGIFCLMWAGVGLYSTWSLSRVSDGNEVDRQLVQQMTVLSQGN
Ci.kos.578-CKO_01067-YP_001452646.1--36H-COG1/1-537        ALMF-NRIRISTTLFLILILCGVLQIGSNGLSFWAFRDGFQSLNQVEQSNQQRAALAQTR
En.clo.1544-EcWSU1_02810-YP_004952663.1--36H-COG1/1-538    AWML-NRIRISTTLFLILILCGVLQVGSNGLSFWAFRDGYQNLQEVEASNQQRAAVAQTR
Sa.ent.407-STM3152-NP_462067.1--36H-COG1/1-547             MRLL-QNFTIRMVMLTILGLFCLLWSGVGLYSVHALSEVSEGNDIDRHLVRQMTVLSQGN
Pe.atr.485-ECA1332-YP_049438.1--36H-COG1/1-555             MRLI-KNIAIRTAMLWVLGVFCVLWGGVSIYIMFSFKEMTATSKTSTLLVQNMNFINQGN
Pe.car.1139-PC1_1208-YP_003016792.1--36H-COG1/1-555        MRLI-KNIAIRTAMLWVLGIFCVLWGGVSIYTMFSFKEMTATSKTSTLLVQNMNFINQGN
En.638.865-Ent638_3407-YP_001178118.1--36H-COG1/1-545      MHML-RNFTIRFVVLAILGVFCLMWAGVGFYSSWSLSRVSEGNETDRQIVKKMTILGQGN
Pa.vag.184-Pvag_1413-YP_003931052.1--36H-COG1/1-554        MGML-KNFTIRAVMLTILGLFCLLWSGVGLYSIHSLGALSEGNDVDRHLVEQMTVLSKGN
Ph.asy.1114-PAU_02684-YP_003041518.1--36H-COG1/1-544       --ML-GRLRISTSLYLLLMMFCLMQIISSGLSLGIIHTDQAYIERIDLGTQKRDSLGLSW
Pa.ana.1905-PAJ_1316-YP_005934192.1--36H-COG1/1-556        MGML-KNFTIRAVMLTILGLFCLLWSGIGLFSIHSLNSLSDGNDVDRHIVEQMTALSKGN
Ci.kos.578-CKO_04394-YP_001455885.1--36H-COG1/1-561        MRLL-QNFTIRIVMLTILGLFCLLWSGVGLFSVHALSKVAEGNDIDRHIVRQMTVLSQGN
Sa.bon.1474-SBG_2750-YP_004731563.1--36H-COG1/1-547        MRLL-QNFTIRMVMLTILGLFCLLWSGVGLYSVHALSEVSEGNDIDRHLVRQMTVLSQGN
Ra.aqu.1678-Q7S_16025-YP_005402985.1--36H-COG1/1-554       MQFL-KNITIRAALLTILGIFCVMWAGVSLYTVTSLDTLTQNTKNSTELVNNMALINKGN
Rahnel.1320-Rahaq_3177-YP_004213898.1--36H-COG1/1-554      MQFL-KNITIRAALLTILGIFCVMWAGVSLYTVTSLDTLTQNTKNSTELVNNMALINKGN
Pectob.2320-W5S_3114-YP_006284066.1--36H-COG1/1-555        MRLI-KNIAIRTAMLWVLGIFCVLWGGVSIYTLFSFKEMTTTSKTSTLLVQNMNFINQGN
Ye.ent.378-YE2971-YP_001007160.1--36H-COG1/1-552           MQFL-RNISIRAALLWVLGAFCLLWGGVSGYTLLSLNQLTQSSNANSVLVENMNLVNQGT
Er.bil.197-EbC_20190-YP_003741400.1--36H-COG1/1-556        MNLL-KNFTIRSVMLWILGLFCLMWSAVGMYSVYSLNALGHGNEVDRELVNQMTTLSKGN
Ph.lum.1262-plu1854-NP_929127.1--36H-COG1/1-544            --ML-GRLRISTSLYLLLMMFCVMQIISSGLSLGIIHADQSYIEQIDLGTQKRDTLGLSW
Er.bil.197-EbC_25340-YP_003741912.1--36H-COG1/1-528        --MV-SRIRISTAIFLLLGLFCVMQIVGNGLSFNSFRLDARNLQDVEVSSNQREALGETW
Di.zea.1140-Dd1591_2937-YP_003005238.1--36H-COG1/1-564     MQFL-KNIAIRTAMLWVLGIFCALWIAVSVYTMYSFKTMNETSKTSALLVNNMNLVNTGN
Er.tas.1011-ETA_14670-YP_001907406.1--36H-COG1/1-518       --ML-GRLRIAGTIICLFGLFCLMQIVASGLSFNSFRLDAHNLNQVVISGGQRDALGESW
Sa.ent.404-STY4234-NP_458344.1--36H-COG1/1-547             ---M-KNIKVITGVIATLGIFSALLLVTGILFYSAVSSDRLNFQNASALSYQQQELGGSF
Sa.ent.407-STM3577-NP_462478.1--36H-COG1/1-547             ---M-KNIKVITGVIATLGIFSALLLVTGILFYSAVSSDRLNFQNASALSYQQQELGGSF
Sa.bon.1474-SBG_3170-YP_004731982.1--36H-COG1/1-547        ---M-KNIKVMTGVIATLGIFSALLLVTGILFYSAVSSDRLNFQNASALSYQQQELGGSF
En.asb.1498-Entas_2591-YP_004829105.1--36H-COG1/1-555      --ML-NRIRVVTMLMMVLVIFALLQLISGGLFFSSLKNNQDSFAASNDLRLQQSELTSTW
En.638.865-Ent638_1858-YP_001176586.1--36H-COG1/1-552      --ML-KNLHVITGIIFALTLFCLLQVVTGGLFYSAVSNDRQNFQNSGNLNAQQESLSDSV
Ci.rod.62-ROD_19331-YP_003365490.1--36H-COG1/1-553         --MF-NRIRVVTMLMMVLGVFALLQLISGGLLFSSLQQNQDSFIVSNSLRQQQSELTSTW
Di.dad.235-Dda3937_01559-YP_003882087.1--36H-COG1/1-566    MQFL-KNIAIRTAMLWVLGIFCVLWIAISVYTMYSFRTMNETSKTSALLVNNMNLVNTGN
Er.bil.197-EbC_37780-YP_003743156.1--36H-COG1/1-553        --ML-KNIKVVTSIIIMLIVFSALLLLSSGLSFNAIHQDKDNFVRASVLTEQQGQLSDAW
Pantoe.297-Pat9b_2001-YP_004115872.1--36H-COG1/1-558       MGIL-KNFTIRAVMLTILGLFCLLWCAVGLFSVHSLNALGEGNEVDRLLVNQMTVLSKGN
```

```
En.clo.1544-EcWSU1_01962-YP_004951821.1--36H-COG1/1-553 --ML-KNLHVITGIIFALTIFCLLQVVTGGLFYSAVSNDRHNFQNSGVLNAQQESLSDSV
En.asb.1498-Entas_1879-YP_004828403.1--36H-COG1/1-554 --ML-KNLHVITGILFALTIFCLLQVVTGGLFYSAVSNDRHNFQNSGMLNAQQESLSDSV
En.bac.2261-D782_1748-YP_007339929.1--36H-COG1/1-547 --ML-NRIRVVTLLMLVLVVFALLQLFSGGLLFSSLSQNQHSFALSNALRTQQTELTDAW
Es.col.1836-Y75_p1862-YP_490148.1--36H-COG1/1-553 --MI-NRIRVVTLLVMVLGVFALLQLISGSLFFSSLHHSQKSFVVSNQLREQQGELTSTW
Cr.tur.6-CTU_33010-YP_003211664.1--36H-COG1/1-555 --MI-KNIKVVSGIIIILLTFTVLQMVTGSLFYSAVNNDRNNFQNANLLNFQQEQLGDSF
Cr.tur.6-CTU_25780-YP_003210941.1--36H-COG1/1-536 -----------------------MQFISGGVFFSSLNENQQSFAASHQLRLQQAQLNQSW
En.clo.1544-EcWSU1_00403-YP_004950264.1--36H-COG1/1-549 --MF-KRIKVITLLISVLLVLGIMQVISAGIFINALNNDKDNFTVSQLSSQNVAEFTDAW
En.638.865-Ent638_2457-YP_001177177.1--36H-COG1/1-555 --ML-NRIRVVTMLMMVLVIFALLQLISGGLFFSSLKQNQESYTAANDLRLQQRELTSAW
Sa.bon.1474-SBG_1755-YP_004730609.1--36H-COG1/1-553 --MF-NRIRVVTMLMMVLGVFALLQLVSGGLLFSSLQHNQQSFVISNELRQQQSELTSTW
Pr.mir.1265-PMI1665-YP_002151396.1--36H-COG1/1-548 MFRF-RKLKISTSLYLLLMMFCVMQVISSGISLGIIHLNNNQITRIDLDTAKRDELGLSW
Cr.sak.579-ESA_01348-YP_001437444.1--36H-COG1/1-556 --ML-NRIRVVSLLMMVLVVFALLQFISGGMFFSSLNENQQSFAASHQLRLQQAQLNQSW
Cr.sak.579-ESA_00560-YP_001436686.1--36H-COG1/1-555 --MI-KNIKVVSGIIIILLTFTVLQMVTGSLFYSAVNNDRNNFQNANLLNFQQEQLGDSF
Sa.ent.407-STM1919-NP_460876.1--36H-COG1/1-553 --MF-NRIRVVTMLMMVLGVFALLQLVSGGLLFSSLQHNQQGFVISNELRQQQSELTSTW
Ci.kos.578-CKO_01066-YP_001452645.1--36H-COG1/1-552 --MF-NRIRVVTMLMMVLGVFALLQLVSGGLLFSSLQQNQQSFVISNDLRQQQSELTSTW
En.asb.1498-Entas_0424-YP_004826963.1--36H-COG1/1-549 --MF-KRIKVITLLVSVLLVLGIMQLISAGIFINALNNDKDNFTVSQVASQNVAEFTDAW
Pantoe.297-Pat9b_1273-YP_004115150.1--36H-COG1/1-553 --ML-KRMKVVTAIVVILVVFTAMQVVSGSLFLTSLMAGQRNFTTSDQLSHQQRELADGW
Ed.tar.1771-ETAF_2264-YP_005699859.1--36H-COG1/1-555 --MF-ERLKISRGLMAVLALFCGLQIFSGIWSLRDASSTDTRLDQISSGFNQIMAMDRAY
Pa.ana.1905-PAJ_3534-YP_005936409.1--36H-COG1/1-553 --ML-KNLKITTGIVAVLFLFSALLTITGFLFYSSVSQAGRNFSRTEQLTLQQQQLSDSV
Pa.vag.184-Pvag_1203-YP_003930842.1--36H-COG1/1-552 --ML-KNLKITHGILAVLAVFITLLTLTGFLFYNGVSKADKNFVAAEQLTLQQQHLSDAV
Pantoe.297-Pat9b_1605-YP_004115478.1--36H-COG1/1-556 --ML-KRINVVTSLIAVLLVFGALQLISGGLFWSALQKDKEAFAVAQVSTNNVAAMSDAW
Cr.sak.579-ESA_00210-YP_001436348.1--36H-COG1/1-557 --MF-KRIKVITLLITVLIVLGAMQLIAAGVFISALNNDKDNFNVSQISSQNVAEFTDAW
Er.tas.1011-ETA_pET460340-YP_001905943.1--36H-COG1/1-543 MNFL-KNYTIRAVLLWILGIFCFMWGAVGIYSLSSLSQLSKGDAVGHQLIDQMTILSKGN
Cr.tur.6-CTU_36610-YP_003212024.1--36H-COG1/1-557 --MF-KRIKVITLLITVLIVLGAMQLIAAGVFISALNNDKDNFNVSQISSQNVAEFTDAW
Pe.car.1139-PC1_1159-YP_003016743.1--36H-COG1/1-556 --MF-KRIKVITILVILLCVLGISQFLTGALSVRALINDREGFLVSQRSNQNVAAFTDAW
Er.tas.1011-ETA_14640-YP_001907403.1--36H-COG1/1-554 --MF-SRIKVVTSLILVLMIFGLLQLLSGGLFIKALTKDKENFALSQTSSDNVTQFTDAW
Pa.vag.184-Pvag_1725-YP_003931362.1--36H-COG1/1-556 --ML-TKIRVVTSLLLVLLVFGLLQAFSGSVFYSALSHDKQSFAVSQLAMQNTQALNDAY
Ed.ict.1187-NT01EI_3742-YP_002935104.1--36H-COG1/1-525 --MF-KNVRVITGIIVLLAIFILLQGVSGRLFYMAIAQDRGNFSQSRALSYQQEQLADGW
En.638.865-Ent638_0380-YP_001175119.1--36H-COG1/1-549 --MF-KRIKVITLLVSVLLVLGAMQIISASVFINALNNDKDNFTVSQVSSKNVAEFTDAW
Er.bil.197-EbC_25360-YP_003741914.1--36H-COG1/1-556 --MF-TRIKVVTSLLLVLLVFGSLQLISGGIFIGALTSDKDSFSIAQNSSDNVASFTDAW
Er.tas.1011-ETA_16910-YP_001907630.1--36H-COG1/1-543 MNFL-KNYTIRAILLWILGIFCFMWGAVGIYSLSSLSQLSKGDAVGRELIDQMTILSKGN
Es.fer.1173-EFER_3055-YP_002384154.1--36H-COG1/1-603 ENML-KRIKIVTSLLLVLAIFGLLQLTSGGLFFSALKNDKENFTVLQTIRQQQSTLNGSW
Xe.nem.162-XNC1_1619-YP_003711880.1--36H-COG1/1-520 -----------------------MQIISSGLSLGIIHTDQSYIERIDLGTQRRDTLGLSW
En.clo.1544-EcWSU1_02811-YP_004952664.1--36H-COG1/1-559 CCML-NRIRVVTMLMMVLVIFALLQLTSGGLFFSSLKQNQDSFAASNDLRVQQSELTSTW
Di.dad.235-Dda3937_03500-YP_003882750.1--36H-COG1/1-553 MNIL-RHITVRRMLLIILTLFTVIWGMASIFTLNSFSSMSDLLNDNMAQKKSYSTLVKGN
Xe.bov.105-XBJ1_1924-YP_003467828.1--36H-COG1/1-523 -----------------------MQIISSGLSLGIIHTDQSYIERIDLGTKRRDTLGLSW
Rahnel.1320-Rahaq_1831-YP_004212576.1--36H-COG1/1-556 --MF-KHIRVSTCMFLLLIVFFVMQLVNSGLSLQAAHTDKLNFEQISNTAEQRDALSQSW
Ra.aqu.1678-Q7S_08965-YP_005401598.1--36H-COG1/1-556 --MF-KHIRVSTCMFLLLIVFFVMQLVNSGLSLQAAHTDKLNFEQISNTAEQRDALSQSW
Cr.sak.579-ESA_01126-YP_001437230.1--36H-COG1/1-514 MGLL-KDFSIRAVMLTVLGVLCVLWAAVGVYSVYSLSAMADGNRVDRQLVSQMTVLSKGN
Pe.atr.485-ECA2712-YP_050803.1--36H-COG1/1-560 MNFL-KNITIRAMLLTILGLFLVVWGGASFFTSTSLSSMTKLLESGETQRKNVEMLVKGN
En.clo.1544-EcWSU1_00560-YP_004950421.1--36H-COG1/1-597 ENML-NRIKIVTSLMLVLAIFGLLQLTSGGLFFNALKHDKENFTVLQTIRQQQSTLNASW
Di.dad.235-Dda3937_03498-YP_003882752.1--36H-COG1/1-561 MSFL-RDISIRVMMLFIMGFFVVLWGGVSGFSLYSLKQVTTLLHDSSMQGRTYTYLVYGN
Cr.tur.6-CTU_27880-YP_003211151.1--36H-COG1/1-514 MGLL-KDFSIRAVMLTVLGVLCVLWAAVGVYSVYSLSAMADGNRVDRQLVSQMTVLSKGN
Pe.car.1139-PC1_1669-YP_003017246.1--36H-COG1/1-560 MNFL-KNITIRAMLLTILGLFLAVWGGASFFTTTSLSSMTKLLESGETQRKNVEKLVKGN
Ra.aqu.1678-Q7S_14450-YP_005402672.1--36H-COG1/1-551 MRFI-RDIKIRTVLITILITFSLLWAGVSGFALYSLNQLKSELNFTNVQQQNGDIINGAN
Ci.kos.578-CKO_03622-YP_001455137.1--36H-COG1/1-517 --MF-KRIKVITLLIIVLLALGAMQLISAGVFISALNNDKQNFTVSQRSSQNVAEFTDAW
Di.zea.1140-Dd1591_2408-YP_003004728.1--36H-COG1/1-557 MNFL-INITVRRMMLVILTLFTLVWGIASCFTLYSLGNVSRLLGDNQEQKRSYSILVHGN
Di.zea.1140-Dd1591_2406-YP_003004726.1--36H-COG1/1-559 MSFL-RNISIRVMMLFIMGFFVVLWGSVSGYSLYSLKQVTVLLNDNSTQSKTHSYLTYGN
Ed.ict.1187-NT01EI_2801-YP_002934203.1--36H-COG1/1-554 --MF-ERLKISRGLMAVLALFCGLQIFSGIWSLRDASSTDSRLDQISSGFNQIMAMDRAY
Pe.car.1139-PC1_1384-YP_003016966.1--36H-COG1/1-559 MEFL-KNVSIKIMVLVIVASLLVAWGVASGFSLYSLYQVTNLLDKSETQRKTYSHLVYGA
Rahnel.1320-Rahaq_2866-YP_004213595.1--36H-COG1/1-551 MRFI-RDIKIRTVLITILITFSLLWAGVSGFALYSLNQLKSELNFTNVQQQNGDIINGAN
Pe.atr.485-ECA1281-YP_049387.1--36H-COG1/1-556 --MF-KRIKVITILVILLFVLGISQFLTGALSVRALINDRDSFLVSQRSNQNVAAFTDAW
Pectob.2320-W5S_1886-YP_006282848.1--36H-COG1/1-549 ------------MLLTILGLFLIVWGGASFFTSTSLNSMTKLLESSETQRKNVEMLVKGN
Pectob.2320-W5S_1696-YP_006282659.1--36H-COG1/1-559 MGFL-KNVSIKIMVLVIVASLLVAWGIASGFSLYSLYQVTHLLDKSETQQKTYSHLVYGA
Di.zea.1140-Dd1591_2407-YP_003004727.1--36H-COG1/1-555 MNIL-RHITVRRMLLIILTLFTVIWGIASFFTLGSFSSMSDLLHDNMAQKKSYSTLVKGN
Pe.atr.485-ECA1509-YP_049611.1--36H-COG1/1-559 MEFL-RNVSIKMMVLVIVASLLVAWGVASGLSLYSLYQVTNLLDKSETQRKTYSHLVYGA
Pa.vag.184-Pvag_1724-YP_003931361.1--36H-COG1/1-520 --ML-KKIKVVTSLIAVLVIFGALQLISGSLFWSALNNDKEAFALSQISNRNVTSMTDAY
Pa.ana.1905-PAJ_1541-YP_005934417.1--36H-COG1/1-520 --ML-KRIKVVTSLIAVLMLFGAMQLLSGSLFWSALNNDKEAFALSQISTRNVAAMTDAY
Mo.mor.2189-MU9_1596-YP_007505015.1--36H-COG1/1-519 MQLL-RNLSIRFVMLSILGILCLTLGCVSFYSDWSLSRVSDGNATDRQLVRQLTVLNQGN
Pr.mir.1265-PMI2808-YP_002152509.1--36H-COG1/1-563 --ML-KRIKISQGLMCVLTLFCIIQVISGVQSIHDAYQTQNKLKQISYSFDQLRTMDNTY
Di.zea.1140-Dd1591_0395-YP_003002764.1--36H-COG1/1-556 MSII-RNIRIKIMMLAILIIFTLLWGGVSFFSLYSLNSLTDEIGLTNVQQINGDIINGAS
En.clo.1544-EcWSU1_03786-YP_004953635.1--36H-COG1/1-515 MNML-RNFTIRFVMLTILGIFCLMWAGVGLYSTWSLSRVSEGNEVDRQLVKQMTILSQGN
Pectob.2320-W5S_3170-YP_006284116.1--36H-COG1/1-556 --MF-KRIKVITILVILLFVLGVSQFLTGALSVRALMNDKDSFLVSQRSNQNVAAFTDAW
Di.dad.235-Dda3937_02184-YP_003884754.1--36H-COG1/1-556 MSFI-CNVRVKIMMLAILIIFTLLWGGVSFFSLYALNSLTGEIGLTNVQQTNGDIINGAS
Ci.rod.62-ROD_32641-YP_003366748.1--36H-COG1/1-517 --MY-KRIKVITLLIVVLIALGAMQLLSAGVFITALKNDKQNFTVSQLSSQNVAELTDAW
Di.dad.235-Dda3937_03499-YP_003882751.1--36H-COG1/1-561 MTFL-RNITVRRMMLMILAMFTVIWGITSIYTLNSFSSMNNLLDDTIDQKKSYSNLVKGN
Di.dad.235-Dda3937_00105-YP_003883346.1--36H-COG1/1-561 --MF-KNIKIVTGLFSLLLALSVLQLVSYGFFFDVMKSDRNNFVVAQTLRKQSGELNASW
Pantoe.297-Pat9b_1604-YP_004115477.1--36H-COG1/1-555 --MF-TKIRVVTSLLLVLLIFGFLQLASGSLFFKALSDDKTSFNVAQLASKNTAAINDAY
Er.tas.1011-ETA_14660-YP_001907405.1--36H-COG1/1-560 --MF-KRMKVVTSLVLVLVLFGSLQLISGGLFFHSLNGDKKSFTLTQQIRQEQANLTSAW
Pr.mir.1265-PMI2809-YP_002152510.1--36H-COG1/1-575 --ML-KRIKISSGLMFILILFCAIQLFSGATGIRDAYLTNKRISQLADGFEQIKTMDYGY
Ed.tar.1771-ETAF_3053-YP_005700648.1--36H-COG1/1-516 -----------------MAIFTLLQGLSGRLFYTAIAQDRDNFSQSRALSYQQEQLADGW
Se.pro.864-Spro_1415-YP_001477647.1--36H-COG1/1-546 --MF-ERLKISHGLMGILTLFCIIQIFSGAWSILDASGTNNRLTQISSGFNQIMAMDNAY
Di.zea.1140-Dd1591_1804-YP_003004134.1--36H-COG1/1-562 --MF-RNIKIVTGLFSLLLALSALQLVSYGFFFGAMKSDRNNFVVAQTLRKQSGELNAAW
Di.dad.235-Dda3937_03501-YP_003882749.1--36H-COG1/1-575 MNFL-KDITVRRMMLIILALFSVVWGMASSFTLYSLGNVNSLLSDNQEQKKSYSILVRGN
Pantoe.297-Pat9b_0851-YP_004114731.1--36H-COG2/1-524 MLSF-KNLKVGVRLGIAFGLVVALLVVVGITSITKISNIKNGITSIVEDRYVKVRLAYDV
Pa.vag.184-Pvag_0291-YP_003929953.1--36H-COG2/1-525 MFSI-KNMKVGMRLAAAFGIIVTLLIVIGVTSITRINSINTAISSIVDDRYVKVRLAFDV
En.clo.1544-EcWSU1_03886-YP_004953735.1--36H-COG2/1-519 -MFL-HDVKIGTKLFLAFGFFIVLMVVSASLSLLSLNRANNGMQSIITRDYPTTVKANQL
Cr.tur.6-CTU_04790-YP_003208842.1--36H-COG2/1-515 -MSI-RDIKIRTKLIFAFAFFIVLLLISAGLSLGSLSQANNNIQQVIAEDYPTTVKANQL
Ci.kos.578-CKO_04484-YP_001455975.1--36H-COG2/1-521 -MLL-HNMKIRSKLFIAFGLFIVLMIVSSGLSLFSLDRTNTSMQNIITHDYPTTVKANLL
Cr.sak.579-ESA_03488-YP_001439539.1--36H-COG2/1-515 -MSI-RDIKIRTKLIFAFAFFIVLLLISAGLSLGSLSQANNNIQQVIAEDYPTTVKANQL
Ci.rod.62-ROD_48071-YP_003368199.1--36H-COG2/1-521 -MFL-RNVKIRSKLFAAFGLFIVLVTLSSGLSLLSLDRANRGMQNIITNDYLITVKANKL
Sa.bon.1474-SBG_2851-YP_004731664.1--36H-COG2/1-521 -MFL-HNIKIRSKLFIAFGLFIVLMVVSSALSLFSLDRANTGMQNIIANDYPTTVKANLL
Sa.ent.407-STM3216-NP_462130.1--36H-COG2/1-521 -MFL-HNIKIRSKLFMAFGLFIVLMVVSSALSLFSLDRANTGMQNIITNDYPTTVKANLL
En.asb.1498-Entas_3753-YP_004830248.1--36H-COG2/1-519 -MFL-HDVKIGTKLFLAFGFFIVLMVVSATLSLLSLNRANNGMQTIITNDYPTTVKANEL
Mo.mor.2189-MU9_3389-YP_007506806.1--36H-COG2/1-521 -MLL-RSIKISTKLFVAFGFSILLMVISATLSVVSLNNANDGIRDILDEDYPITVKANLL
Sa.ent.404-STY3394-NP_457606.1--36H-COG2/1-522 -MFL-HNIKIRSKLFMAFGLFIVLMVVSSALSLFSLDRANTGMQDIITNDYPTTVKANLL
En.638.865-Ent638_3526-YP_001178235.1--36H-COG2/1-519 -MFL-HGVKIGTKLFLAFGFLILLMVVSASLSLMSQNRANNGMQSIINNDYPTTVKANQL
Pe.atr.485-ECA1774-YP_049874.1--36H-COG2/1-554 -MSL-SNWRIGYRLGAGFSFLVLMLLIIGSVAISKLSDFHEKMDDIVSQSYPLTVKSNKL
En.638.865-Ent638_2119-YP_001176845.1--36H-COG2/1-549 -MKI-SNMRVGTRLSVGFFLVLFMMFIMGMISWQLTQSNSTSVNTMIYQDLQKERLVQEW
```

```
Pectob.2320-W5S_2813-YP_006283770.1--36H-COG2/1-554      -MGL-SNWRIGYRLGAGFSFLVLMLLIIGSVAISKLSDFHQKMDDIVSQNYPLTVKSNTL
Di.zea.1140-Dd1591_0695-YP_003003055.1--36H-COG2/1-561   -MRL-SDWRIGYRLGAGFAVLAVMLVFVGMLSIAKLSGFHDDARDIVQKIYPQTVDANAL
Pa.vag.184-Pvag_pPag30079-YP_003729820.1--36H-COG2/1-546 ---M-NNIRIGTRLGAAFAFILLLLVIVAFTGFSRIQSLGETADTLAGSRYQKASAATNL
Er.bil.197-EbC_38940-YP_003743272.1--36H-COG2/1-526      MASV-KNMKVGVRLGAAFSLVILLLIIVSVTAVVKINTINDAVSSIVSDRYIKVRLAFDV
Ra.aqu.1678-Q7S_24891-YP_005419232.1--36H-COG2/1-554     FKLI-NNMRVGARLGAAFSLVILLLVIVSGIAITKISSINTSIEQIISDRYVKVRLAFDV
En.asb.1498-Entas_2287-YP_004828805.1--36H-COG2/1-513    SLRL-RNIKMSKKLSAGFGVVLLLVAVSTAMSVLRFKEIRDVYQQT-------NLIYNIN
Rahnel.1320-Rahaq_4869-YP_004215574.1--36H-COG2/1-554    FKLI-NNMRVGARLGAAFSLVILLLVIVSGIAITKISSINTSIEQIISDRYVKVRLAFDV
Di.dad.235-Dda3937_00027-YP_003883574.1--36H-COG2/1-554  -MSF-SNFKIGYRLAIGFSFLIIMLLIAGAVALSNLSDFNRKMDYTVSELYPLTAKGNQL
Pr.stu.1965-S70_05790-YP_006215722.1--36H-COG2/1-520     -MSL-KDIKIGVKLFIAFAFFIFLLLLSSALSLISLNKANNGIQDIIYKDYPMTVNANQL
En.638.865-Ent638_2100-YP_001176826.1--36H-COG2/1-512    SQRL-RNIKMSRKLAAGFGLVLLLVAVSTIMSVLRFKEIRDVYQQT-------NLIYNVN
Pe.car.1139-PC1_2526-YP_003018093.1--36H-COG2/1-554      -MSL-SNWRIGYRLGAGFSFLVLMLLVIGSVAISKLSDFHEKMDEIVSQNYPLTVKSNKL
Mo.mor.2189-MU9_702-YP_007504121.1--36H-COG2/1-525       -MNL-KNTTIRVRLSVTFGVLIAILALVSGMAVMNLYKDKEMFTNYVNGTYALQVQAYEV
Pe.car.1139-PC1_3443-YP_003018995.1--36H-COG2/1-511      WMSF-QNLSVTRKLLVGFGLLLIMGVILSMVGFYGLHNSDQSLKRISR-------LGAMY
Pa.ana.1905-PAJ_0915-YP_005933791.1--36H-COG2/1-559      GINM-NNIRIGVRLGITFGFMALLVMFMVLIGIVKINALGNANDDISGSLYTKASTSLSI
Pectob.2320-W5S_3736-YP_006284671.1--36H-COG2/1-510      WMSF-QNLSVTRKLLVGFGLLLIMGVILSMVGFYGLYNSDQSLKRISR-------LGAIY
Pe.atr.485-ECA0183-YP_048310.1--36H-COG2/1-553           -MNL-ANWRIGYRLGAGFAILIVMLFVVSIFSLSKLSGFQDSARSIVKDVYPQTVDANNL
Rahnel.1320-Rahaq_3107-YP_004213828.1--36H-COG2/1-503    AGRV-RHASIASKLGAGFALVLLLGSFIAVSGVYHLTSINQRAEKASLLKTVNDLLSDA-
Pe.car.1139-PC1_4071-YP_003019622.1--36H-COG2/1-553      -MSL-ANWRIGYRLGAGFAILILMLFVVSIFSLSKLSSFQDSARSIVKDVYPQTVDSNSL
Di.dad.235-Dda3937_03462-YP_003884507.1--36H-COG2/1-563  -MRL-SDWRIGYRLGAGFAVLAVMLVVVGILSITRLSSFHDSARDIVQEIYPQTVDANTL
Ra.aqu.1678-Q7S_15665-YP_005402913.1--36H-COG2/1-503     AGRV-RHASIASKLGAGFALVLLLGSFIAVSGVYHLTSINQRAEKASLLKTVNDLLSDA-
Pectob.2320-W5S_4505-YP_006285423.1--36H-COG2/1-555      -MNL-ANWRIGYRLGAGFSILILMLFAVSIFSLSKLSGFQDGARGIVKDVYPQTMDANNL
Pe.atr.485-ECA3642-YP_051730.1--36H-COG2/1-551           ---M-KNYKIGTRLAGGFGLLIALSLAMLTSGIYQLNQVSSSTQQMMQEPLRKERLASDW
Pe.car.1139-PC1_1417-YP_003016999.1--36H-COG2/1-554      MDFF-KNIKIGNRLALGFGLLIILTLVLSSVGYYFMKDIGTEVDEITEDRMVKVDMLRDI
En.asb.1498-Entas_4509-YP_004821569.1--36H-COG2/1-559    IMNV-FNWKIGTRLGGGFCLLLALLIMVSILSLTSLLSLRNDATAIVEKYYPTTVIANHL
Pa.ana.1905-PAJ_3064-YP_005935940.1--36H-COG2/1-544      ---M-NNIRIGIRLVAAFGFMGMLVIFMVLIGITKTRSLADANSEIAGGLYAKASESAML
Pectob.2320-W5S_1733-YP_006282696.1--36H-COG2/1-535      -------------------MLIILTLILSAVGYYFMKNIGAEVDGITEDRMPKVDMVRDI
Pe.atr.485-ECA4334-YP_052421.1--36H-COG2/1-556           MNLI-KNSSLGKMLGTGFTLVIAIGFLVAIFGRIQLDKLGENIQVLSQVRITNLLMMQEF
Di.zea.1140-Dd1591_1602-YP_003003935.1--36H-COG2/1-554   -MNL-SNLKIGYRLAIGFSFLIVMLFISGAVALSNLNEFNRKMNYTVSELYPLTAKGNKL
Pectob.2320-W5S_4506-YP_006285424.1--36H-COG2/1-540      -MNL-ANWRIGYRLGGGFAVLILMLFAVSLFSLSKLSGFQDGARDIVKEVYPQTVDANDL
Di.zea.1140-Dd1591_1453-YP_003003794.1--36H-COG2/1-532   -------MKISTRLWSFTGMLSLGILIMGAIGLFTAEKANQSLKTVYEDRTVVLAQLEKL
Pectob.2320-W5S_0106-YP_006281117.1--36H-COG2/1-556      MNLI-KNSSLGKMLGTGFTLVIAIGFLVAIFGRIQLDKLGENIQVLSQVRIANLLMMEEF
Di.zea.1140-Dd1591_0769-YP_003003127.1--36H-COG2/1-505   HLSL-KNLPVTRKLLLGFGLLLLIGITLSLVGFSGLHNSDQSLKRISR-------LGSMY
Ra.aqu.1678-Q7S_18035-YP_005403387.1--36H-COG2/1-535     -MSF-KHTTVRTQLTLGFGVLVVIVLAVVLLSINALSQANNRFKTHVQQVSAREAAVNLI
Rahnel.1320-Rahaq_3579-YP_004214298.1--36H-COG2/1-535    -MSF-KHTTVRTQLTLGFGVLVVIVLAVVLLSINALSQANNRFKTHVQQVSAREAAVNLI
Pe.car.1139-PC1_4072-YP_003019623.1--36H-COG2/1-540      -MNL-ANWRIGYRLGGGFAILILMLFTVSIFSLSKLSGFQDGARDIVKDVYPQTVDANDL
Pe.car.1139-PC1_3464-YP_003019016.1--36H-COG2/1-551      ---M-KNYKIGTRLAGGFGLLIALSLAMLTSGIYQLNQVSSSTKQMMQEPLRKERLASDW
Pe.car.1139-PC1_0111-YP_003015709.1--36H-COG2/1-556      MNLI-KNSSLGKMLGTGFTLVIAIGFLVAVFGRIQLDKLGENIQLLSQVRITNLIMMQEF
Pe.atr.485-ECA0182-YP_048309.1--36H-COG2/1-542           -MNL-ANWRIGYRLGGGFAILILMLFAVSIFSLSKLSNFQDGARDIVKEVYPQTVDANNL
Pectob.2320-W5S_3753-YP_006284688.1--36H-COG2/1-551      ---M-KNYKIGTRLASGFGLLIALSLVMLTSGIYQLNQVSQRTQEMMQEPLRKERLAADW
Pa.ana.1905-PAJ_2666-YP_005935542.1--36H-COG2/1-514      -------MKISTRLTISFGLLITLFIICTSVAYNGLNNARNGMDNAVNVKMKKYRLGMEM
Ci.rod.62-ROD_16161-YP_003365194.1--36H-COG6/1-557       LGFL-HHIRLVPLFFSILGGILLLFALSAGLAGYFLMQADRDQRDVTDEIQIRMGLSNSS
En.asb.1498-Entas_2095-YP_004828614.1--36H-COG6/1-563    LSFL-HHIRLVPLFSSILGGIILLFALSSGLAGYFLLKADTDQQDVTSEIQVRLGLSNSS
En.638.865-Ent638_1961-YP_001176688.1--36H-COG6/1-563    LSFL-HHIRLVPLFSSILGGIILLFALSSVLAGYFLLQADNDQQDVTQEIQVRTGLSNSS
Cr.sak.579-ESA_01710-YP_001437800.1--36H-COG6/1-565      LSFW-HHIRLVPLFSVILGGILVLFALCGGLAGYFLMQGDSALNNVTQEIQIRSGLADSA
Cr.tur.6-CTU_22440-YP_003210607.1--36H-COG6/1-565        LSFW-HHIRLVPLFSVILGGILVLFALCGGLAGYFLMQGDSALNNVTQEIQIRSGLADSA
Ci.kos.578-CKO_01456-YP_001453025.1--36H-COG6/1-562      LGFL-HHIRLVPLFSSILGGILLLFALSSGLAGYFLLQADRDQRDVTDEIQVRMGLSNSS
Rahnel.1320-Rahaq_1930-YP_004212674.1--36H-COG6/1-566    TSFI-SNIRLVTLISLILTGILFLFAVSIGTSGFFLKQSNAALANATQELQIRLALSNSS
Pantoe.297-Pat9b_2403-YP_004116261.1--36H-COG6/1-564     LSIW-QNLRLMPLFSMIFGGILLLFALCIGLASYFLIQSNNSLKDATDEIQIRMGISNSS
Er.bil.197-EbC_26700-YP_003742048.1--36H-COG6/1-567      ISFW-QHLRLVPLFSAILGGILLLFALCIALASYFLIQSNQSLKDVTEEIQVRMAISNSS
Pectob.2320-W5S_1760-YP_006282723.1--36H-COG6/1-570      AGVL-SNLRLVPLFVIILGGIMLLFAASIGASSYFLQNSNQSLNDVTQEIDTRMGISNSS
Pa.ana.1905-PAJ_1621-YP_005934497.1--36H-COG6/1-568      LSLW-QNIRLVPLFSLIFGGILFLFALCIAISSYFLILSNQSLKDATDEIQVRNGLIDSS
En.clo.1544-EcWSU1_02120-YP_004951978.1--36H-COG6/1-576  LSFL-HHIRLVPLFSSILGGIILLFALSSGLAGYFLLQADADQQDVTSEIQVRMGLSNSS
Ra.aqu.1678-Q7S_09815-YP_005401764.1--36H-COG6/1-566     TSFI-SNIRLVTLISLILTGILFLFAVSIGTSGFFLKQSNAALANATQELQIRLALSNSS
Ye.pse.585-YPTB2412-YP_070925.1--36H-COG6/1-579          VSFI-NNMRLVTLFIVILAGILLLFAAAIGTSGYFLKQSNQSLAQATQEIDIRQGLSNSS
Ye.ent.378-YE2588-YP_001006789.1--36H-COG6/1-579         VFFV-NNMRLVTLFIAILAGILLLFAAAIGTSGYFLKQSNQSLEEATQELDIRLGLSNSS
Di.dad.235-Dda3937_02787-YP_003883649.1--36H-COG6/1-572  TGIL-GNLGLVPLFVIILGGIMLLFALAIGTASYFLVRANQSLDYVTQEIDVRLGLSNSS
Pa.vag.184-Pvag_1800-YP_003931435.1--36H-COG6/1-568      AGIW-QNLRLVPLFSLIFGGILLLFALCIGVASYFLISSNHSLDDATNEIQVRMGLANSS
En.bac.2261-D782_2307-YP_007340470.1--36H-COG6/1-558     LSFL-HHIRLVPLFSAILGGIILLFALSSGLAGYFLLQAEQDQKDVTEELQVRMGLSNSS
Pe.atr.485-ECA1683-YP_049784.1--36H-COG6/1-573           VGML-SNLRLVPLFIIIILGGIMLLFAASIGTSSYFLQSSNQSLDDVTQEIDTRMGISNSS
Pe.car.1139-PC1_2617-YP_003018183.1--36H-COG6/1-573      AGML-SNLRLVPLFVIIILGGIMLLFAASIGTSSYFLQSSNQSLDDVTQEIDTRMGISNSS
Di.zea.1140-Dd1591_1535-YP_003003868.1--36H-COG6/1-574   TGIL-GNLGLVPLFVIILGGIMLLFALAIGTASYFLVRANQSLDYVTQEIDVRLGLSNSS
Di.zea.1140-Dd1591_3580-YP_003005868.1--36H-COG6/1-572   VSSW-HHWRLMPMFSSIIGVILILFALAIGFSVHFLMRSNSSLNDVTAEIQVRLGVSNSS
Es.col.1836-Y75_p1397-YP_489687.1--36H-COG6/1-546        LGFL-HHIRLVPLFACILGGILVLFALSSALAGYFLWQADRDQRDVTAEIEIRTGLANSS
Pe.atr.485-ECA3902-YP_051990.1--36H-COG6/1-575           LSYI-NNIRLVSLFITLFSIILILFALSIGTASYFLKQSNDSLDKANELSDIRAGISSSL
Sa.bon.1474-SBG_1454-YP_004730321.1--36H-COG6/1-537      RGFL-HHIRLVPLFSSILGGILLLFALSAGLAGYFLLQADRDQRDVTDEIQVRMGLSNSA
Sa.ent.407-STM1626-NP_460585.1--36H-COG6/1-541           LGFL-HHIRLVPLFSSILGGILLLFALSAGLAGYFLLQADRDQRDVTDEIQVRMGLSNSA
Pe.car.1139-PC1_3679-YP_003019230.1--36H-COG6/1-567      LSYI-NNIRLVSLFITLFSIILILFALSIGTASYFLKQSNDSLDKANELSDIRAGISSSL
Pectob.2320-W5S_4020-YP_006284955.1--36H-COG6/1-575      LSYI-NNIRLVSLFITLFSIILILFALSIGTASYFLKQSNDSLDKANELSDIRAGISSSL
Di.zea.1140-Dd1591_0523-YP_003002884.1--36H-COG6/1-568   ISSI-NNIRLVTLFITLLTIILALFALSIGTASYFLKQSNASLERANLVSEIRAGISSSM
Di.dad.235-Dda3937_02665-YP_003881279.1--36H-COG6/1-572  VSFW-HHWRLMPMFSSIIGVILILFALAIGFSVHFLMRSNSSLNDVTAEIQVRLGVSNSS
```

```
Pr.mir.1265-PMI1666-YP_002151397.1--36H-COG1/1-568       ANLLQARNNINRAAISYLLQDKDLNVDSSLSVE---YLTGLARKNMSRADDKFKEFEQ--
Di.dad.235-Dda3937_02779-YP_003883640.1--36H-COG1/1-566  SYLLQTRNTLNRAGTRYAMDASG-GASGGVSAK---ELIELAKKQLVIANAHFANYEK--
Di.zea.1140-Dd1591_1542-YP_003003875.1--36H-COG1/1-566   SYLLQTRNTLNRAGTRYAMDASG-GVTGGVSAK---DLIELAKKQLVIANTHFANYEK--
Pe.atr.485-ECA1691-YP_049792.1--36H-COG1/1-561           SYLLQTRNTLNRAGTRFALDVS--GTGAGVGGK---ELLTSAEKQLAVANDYFIRYEK--
Se.pro.864-Spro_2983-YP_001479212.1--36H-COG1/1-556      VNLLQARNTLNRAGIRHMMDTN--NIGSGATVA---ELLARAKSNLVVAEQHYAAYDK--
Ph.asy.1114-PAU_02685-YP_003041519.1--36H-COG1/1-564     VNLLQARNSLNRAGIRYMMKKDE-GVDNYYTLE---QLLTDARSNLSIAEQRFEQYEQ--
Pr.stu.1965-S70_18135-YP_006218124.1--36H-COG1/1-561     VNLLQARSNLNRSVNAFLLEGR--TYQDDASAE---DLIKAAERNFKAADAAYQRYIN--
Mo.mor.2189-MU9_1762-YP_007505181.1--36H-COG1/1-561      VNLIQARANLNRAANGFLLREH--TFKDDATPD---ELIKQATRQLADADAAYARYMK--
Pe.car.1139-PC1_2609-YP_003018175.1--36H-COG1/1-561      SYLLQTRNTLNRAGTRFALDAS--GTGAGAGGK---ELLVSAQKQLVVANDFFSRYEK--
Xe.nem.162-XNC1_1620-YP_003711881.1--36H-COG1/1-567      INLLQARNNLNRIGILMIMKQQ--NLDSDYSID---DIWAEAKVNISRAERRFEQFDQ--
Pectob.2320-W5S_1768-YP_006282731.1--36H-COG1/1-561      SYLLQTRNTLNRAGTRFALDAS--GTGAGVGGK---ELLASAQKQLSVANDFFSRYEK--
Pa.vag.184-Pvag_1723-YP_003931360.1--36H-COG1/1-558      VSLVQARNTLNRAGIRFLQDAQ--MSGSGASVK---ELVALAGEELKQAEINYQIFND--
Ye.pes.133-YPZ3_2024-YP_003568196.1--36H-COG1/1-557      VYLLQTRNTLNRAGVRYMMDIN--HTGSGPTVK---DLISSAKGTLSVAEDRFKRYEK--
Ye.pse.585-YPTB2401-YP_070915.1--36H-COG1/1-557          VYLLQTRNTLNRAGVRYMMDIN--HTGSGPTVK---DLISSAKGTLSVAEDRFKRYEK--
Ye.ent.378-YE2575-YP_001006778.1--36H-COG1/1-557         VNLLQTRNTLNRAGIRYMMDVN--HTGSGPTVN---DLLASAKGTLGVAAERFKSYEQ--
Pa.ana.1905-PAJ_1540-YP_005934416.1--36H-COG1/1-563      VALVQARNTLNRAGIRYLQDGQ--MAGSGATVK---ELVALADSELKRAEEGYAMFNA--
Ph.lum.1262-plu1853-NP_929126.1--36H-COG1/1-564          VNLLQARNNLNRAGIRYMMKKDE-GVDNYYTLE---QLLTDARSNLSIAEQRFEQYEQ--
Serrat.1901-SerAS13_3075-YP_006025914.1--36H-COG1/1-555  VNLLQARNTLNRAGIRYMMDAN--KIGSGASVT---ELLAGAKNSLLVAEQHYAAYDK--
Sa.ent.404-STY2128-NP_456485.1--36H-COG1/1-553           DLMLQTRINLSRSAARMMMDAS--NQQSSAK-T---DLLQNAKTTLAQAAAHYANFKN--
Ra.aqu.1678-Q7S_08960-YP_005401597.1--36H-COG1/1-564     IYLLQTRNTLNRAGIRFMLDAN--QMGSGATVK---ELIDTATKDLQIADQHFASYQK--
Se.mar.2260-D781_2759-YP_007345195.1--36H-COG1/1-555     VSLLQARNTLNRAGIRYMMDAN--KIGSGASVQ---DLLASARSALDDAQQRYAAYEK--
Rahnel.1320-Rahaq_1830-YP_004212575.1--36H-COG1/1-564    IYLLQTRNTLNRAGIRFMLDAN--QMGSGATVK---ELIDTATKDLQIADQHFASYQK--
Se.ply.1407-SerAS9_3072-YP_004506452.1--36H-COG1/1-555   VNLLQARNTLNRAGIRYMMDAN--KIGSGASVT---ELLAGAKNSLLVAEQHYAAYDK--
Ed.ict.1187-NT01EI_1461-YP_002932882.2--36H-COG1/1-555   VALLQTRNTLNRAGIRYMADLN--KTGTGSTVT---DLMQLARTELKTAEGEWQRYQG--
En.aer.1436-EAE_15535-YP_004593297.1--36H-COG1/1-556     VDLLQARNSLNRAGIRYMMDTN--KIGSGATID---ELLAKAKEELARAERNYTAYEK--
Xe.bov.105-XBJ1_1923-YP_003467827.1--36H-COG1/1-567      INLLQARNNLNRVGIIYIMKER--DLDGTYQID---DVLAESKVSLARGERRFEQFEQ--
Serrat.1408-SerAS12_3073-YP_004501499.1--36H-COG1/1-555  VNLLQARNTLNRAGIRYMMDAN--KIGSGASVT---ELLAGAKNSLLVAEQHYAAYDK--
Er.bil.197-EbC_25350-YP_003741913.1--36H-COG1/1-559      VSLVQARNTLNRAGIRYLQDAN--QMGSGATVK---ELLVLVQQELDIADKSMAVFDS--
Er.tas.1011-ETA_14650-YP_001907404.1--36H-COG1/1-554     ISLVQARNTLNRTGIRYMLDAN--RMGSGDSVK---QLLAAAQDQLAVAEKNYQLWNS--
Sa.ent.407-STM4533-NP_463392.1--36H-COG1/1-553           VELLQTRNTLNRAGIRWMMDQS--NIGSGATVA---ELMQGATNTLKLTEKNWEQYEA--
Es.col.1836-Y75_p4240-YP_492486.1--36H-COG1/1-551        VALLQTRNTLNRAGIRYMMDQN--NIGSGSTVA---ELMESASISLKQAEKNWADYEA--
En.bac.2261-D782_3913-YP_007342002.1--36H-COG1/1-553     VALLQTRNTLNRAGIRYMMDQN--NIGSGATVA---ELMQVAQKSLSDAEKRWAEYEA--
Ci.kos.578-CKO_03442-YP_001454958.1--36H-COG1/1-554      VALLQTRNTLNRAGIRYMMDQN--NIGSGATVT---DLMQIATTSLKLAEKHWAEYEA--
Cr.sak.579-ESA_03402-YP_001439456.1--36H-COG1/1-555      VALIQTRNTLNRAGIRHMMDAN--NIGSGATVQ---ELMQIASSSLKQAETHWADYQA--
Pantoe.297-Pat9b_1606-YP_004115479.1--36H-COG1/1-558     VSLVQARNTLNRAGIRYLLDTQ--QAGSGATVK---ELVALASEELKVADQGFAEFNA--
Cr.tur.6-CTU_05670-YP_003208930.1--36H-COG1/1-555        VALIQTRNTLNRAGIRHMMDAS--NIGSGATVQ---ELMQIASSSLKQAETHWAEYQA--
Sa.bon.1474-SBG_3944-YP_004732724.1--36H-COG1/1-553      VELLQTRNTLNRAGIRWMMDQS--NIGSGATVA---ELMQGAKNALKLTEKNWAQYEA--
En.asb.1498-Entas_0547-YP_004827084.1--36H-COG1/1-554    VALLQTRNTLNRAGIRYMMDQS--NIGSGATVS---DLMQIASASLKQAEKNWADYEA--
Ci.rod.62-ROD_48461-YP_003368233.1--36H-COG1/1-554       VALLQARNTLNRAGIRYMMDQN--DIGSGPTVD---ELMHDAAKSLKEAEKFWADYEA--
Ed.tar.1771-ETAF_1249-YP_005698855.1--36H-COG1/1-529     VALLQTRNTLNRAGIRYMADLN--KTGSGATVP---DLMQLASAELKEAEQEWERYQG--
En.638.865-Ent638_0513-YP_001175251.1--36H-COG1/1-586    VALLQTRNTLNRAGIRYMMDQS--NIGSGATVN---DLMQIASTSLKQAEKNWADYEA--
Ed.ict.1187-NT01EI_1462-YP_002932883.1--36H-COG1/1-532   VALLQARNTLNRAGTRVALHI------PQEQID---ALMYRVRAMLKKADGTMQQYMV--
Ye.ent.378-YE2573-YP_001006777.1--36H-COG1/1-545         VSLLQARNTLNRAATRSALKV------PQEKVN---ELMGNARSSLQKADLYFNQFLA--
Ed.tar.1771-ETAF_1250-YP_005698856.1--36H-COG1/1-530     VALLQARNTLNRAGTRAALHI------PQDQID---ALMHRVRAMLKKADDSMQQYMA--
Mo.mor.2189-MU9_1763-YP_007505182.1--36H-COG1/1-524      EDLMQARNLLTQVVVHIKVGS------EESDIR---QVKAAFDLLLDKAAEDYSNFKS--
En.aer.1436-EAE_15540-YP_004593298.1--36H-COG1/1-536     VSLLQARNTLNRAGTRAALKV------PQEQVD---ALMGGARSSLQKADLYFNQFLD--
En.638.865-Ent638_2456-YP_001177176.1--36H-COG1/1-533    AVLLQASTALNKAGTLTALSY------PPDDIK---ALMTTARDSLKQAEVQFKAFSE--
Se.pro.864-Spro_2982-YP_001479211.1--36H-COG1/1-541      VSLLQARNTLNRAGTRAALKL------PQDQVN---ALMGNARSSLQKADLYFNQFLA--
Es.col.1836-Y75_p1861-YP_490147.1--36H-COG1/1-533        AVMLQASTALNKAGTLTALSY------PADDIK---TLMTTARASLTQSTTLFKSFMA--
Serrat.1901-SerAS13_3074-YP_006025913.1--36H-COG1/1-541  VSLLQARNTLNRAGTRSALKL------PQEQVN---ALMGNARSSLQKADLYFNQFLA--
Ye.pes.133-YPZ3_2023-YP_003568195.1--36H-COG1/1-536      VSLLQARNTLNRAATRSALNV------PQEQVN---ALMGSARSALQKAELYFNQFQA--
Serrat.1408-SerAS12_3072-YP_004501498.1--36H-COG1/1-541  VSLLQARNTLNRAGTRSALKL------PQEQVN---ALMGNARSSLQKADLYFNQFLA--
Se.mar.2260-D781_2758-YP_007345194.1--36H-COG1/1-534     VALLQARNSLNRAGTRTALRL------PAEQVN---ALLEDARRSLQSAERDFRRFQA--
Ye.pse.585-YPTB2400-YP_070914.1--36H-COG1/1-536          VSLLQARNTLNRAATRSALNV------PQEQVN---ALMGSARSALQKAELYFNQFQA--
Se.ply.1407-SerAS9_3071-YP_004506451.1--36H-COG1/1-541   VSLLQARNTLNRAGTRSALKL------PQEQVN---ALMGNARSSLQKADLYFNQFLA--
Pr.stu.1965-S70_18140-YP_006218125.1--36H-COG1/1-521     EDLMQARSTLTQIVVHMKVDS------DTASIN---NAKAVFEQFLQQAADDYEKFKA--
En.bac.2261-D782_1749-YP_007339930.1--36H-COG1/1-533     AALLQTSQVLNRTGTLTALGY------PLDEIK---AGMVDARQALKLADSEFAAFKS--
En.asb.1498-Entas_2590-YP_004829104.1--36H-COG1/1-533    AVLLQASTALNKAGTLTALSY------PPDDIK---ALMATARDSLKQADAQFKAFVS--
En.asb.1498-Entas_3672-YP_004830170.1--36H-COG1/1-545    DQYFRFVTRLSRAMEVKASG-------GN-------PDLASAQQALDNMSKKLEQMKA--
Ci.kos.578-CKO_01067-YP_001452646.1--36H-COG1/1-537      AVLLQASTALNKAGTLTALSY------PPEDIK---QLMTTARESLAQSGVVFKGFMA--
En.clo.1544-EcWSU1_02810-YP_004952663.1--36H-COG1/1-538  AVLLQASTALNKAGTLTALSY------PPDDIK---ALMVTARDSLKLADAQFNAFAA--
Sa.ent.407-STM3152-NP_462067.1--36H-COG1/1-547           DQYFRFVTRLSRAMDVKIGG-------GT-------PDFAPARQSLENMRQKLEEMKA--
Pe.atr.485-ECA1332-YP_049438.1--36H-COG1/1-555           DQYFRMVTRLARAVDARRSG-------DNATAD---KEQASALVALDKLKSDLVAFNA--
Pe.car.1139-PC1_1208-YP_003016792.1--36H-COG1/1-555      DQYFRMVTRLARAVDARRSG-------DNATAD---KEQASALVALDKLKSDLVAFNA--
En.638.865-Ent638_3407-YP_001178118.1--36H-COG1/1-545    DQYFRFVTRLSRAMEAKAAG-------GT-------PDLTSAQTALDNMSKRLEEMKA--
Pa.vag.184-Pvag_1413-YP_003931052.1--36H-COG1/1-554      DQYFRVITRLSRVMESKAAG-------KTLTD----EAYAPVQQALDSMTQQLARFKQ--
Ph.asy.1114-PAU_02684-YP_003041518.1--36H-COG1/1-544     AALLQSRNTLNRIAVGKTLQQ------SEDHIS---GMVANVRETLDKAEMHFAEFIS--
Pa.ana.1905-PAJ_1316-YP_005934192.1--36H-COG1/1-556      DQYFRMITRLSRVMEKKAAG-------TAQND----EAFAPVQQALDSMSQQLTLFKQ--
Ci.kos.578-CKO_04394-YP_001455885.1--36H-COG1/1-561      DQYFRFVTRLSRAMDVKIGG-------GT-------PDFVPVQQALDNMSKKLEEMKT--
Sa.bon.1474-SBG_2750-YP_004731563.1--36H-COG1/1-547      DQYFRFVTRLSRAMDVKISG-------GT-------PDFTPARQSLENMRKKLEEMKA--
Ra.aqu.1678-Q7S_16025-YP_005402985.1--36H-COG1/1-554     DQYFRTVTRLSRAVAQLQA--------DPSKGP---EIFDTAKQSLSGLTQALASFKA--
Rahnel.1320-Rahaq_3177-YP_004213898.1--36H-COG1/1-554    DQYFRTVTRLSRAVAQLQA--------DPSKGP---EIFDTAKQSLSGLTQALASFKA--
Pectob.2320-W5S_3114-YP_006284066.1--36H-COG1/1-555      DQYFRMVTRLARAVDARRSG-------DNATAD---KEQASALVALDKLKSDLVAFNA--
Ye.ent.378-YE2971-YP_001007160.1--36H-COG1/1-552         DQYFRMVTRLARSVDYRQSG-------NIADAD---KELKSSNAALENLKQKLAQFKA--
Er.bil.197-EbC_20190-YP_003741400.1--36H-COG1/1-556      DQYFRFITRLTRAVESRQSG-------SASAA----ADMASVQKALDNMSALLADFKA--
Ph.lum.1262-plu1854-NP_929127.1--36H-COG1/1-544          AALLQSRNTLNRIAVGKTLQQ------SEDHIG---SMVANVKETLDRAEMHFAEFIA--
Er.bil.197-EbC_25340-YP_003741912.1--36H-COG1/1-528      AALLSARVTLSRAGTRAALSL------PRDQVT---SLMAKAQEDLNAADNAFKRFNA--
Di.zea.1140-Dd1591_2937-YP_003005238.1--36H-COG1/1-564   DQYFRMVTRLARAIDYRQSG-------DNQKAD---EQQKSSQAALDLLKSNLEAFKK--
Er.tas.1011-ETA_14670-YP_001907406.1--36H-COG1/1-518     AALLSTRLTLSRASTRAAINR------PHDQVV---SLMSKAQADMDVANKAFNDFQA--
Sa.ent.404-STY4234-NP_458344.1--36H-COG1/1-547           QTLIETRVTINRVAIRMLKNQR--DPASLDAMN---TLLTNAGASLNEAEKHFNNYVN--
Sa.ent.407-STM3577-NP_462478.1--36H-COG1/1-547           QTLIETRVTINRVAIRMLKNQR--DPASLDAMN---TLLTNAGASLNEAEKHFNNYVN--
Sa.bon.1474-SBG_3170-YP_004731982.1--36H-COG1/1-547      QTLIETRVTINRVAIRMLKNQR--DPASLDAMN---TLLTSAGVSLNEAEKHFNNYAN--
En.asb.1498-Entas_2591-YP_004829105.1--36H-COG1/1-555    DLMLQTRINLSRSSARMMMDPN--NQQSTAK-T---DLLKNARATLADAAKHYDAFKK--
En.638.865-Ent638_1858-YP_001176586.1--36H-COG1/1-552    NTLVKTRVTVTRVAIRYLKNQR--DPASLAAIN---KLLGNASTSLGKAEAYYSAWHQ--
Ci.rod.62-ROD_19331-YP_003365490.1--36H-COG1/1-553       DLMLQTRINLSRSSARMMMDVE--NQQSSAK-T---ELLANAKATLAQAAAHYNNFKA--
Di.dad.235-Dda3937_01559-YP_003882087.1--36H-COG1/1-566  DQYFRMVTRLARAIDYRQSG-------DNQKAD---EQQKSSQAALDLLKSNLEAFKK--
Er.bil.197-EbC_37780-YP_003743156.1--36H-COG1/1-553      QTLIKTRVTINRAAIRILKKQT--DPAALAGIT---KLLATASTTLDDAAFHFANYQK--
Pantoe.297-Pat9b_2001-YP_004115872.1--36H-COG1/1-558     DQYFRVVTRLSRVMEMRAAG-------TALPA----DAFASVQQALDSMKQQLEQFKA--
```

```
En.clo.1544-EcWSU1_01962-YP_004951821.1--36H-COG1/1-553  NTLIKTRVTVTRVAIRYLKNQR--DPASLAAIN---KLLGTAADSLAKAEAYNKEWQK--
En.asb.1498-Entas_1879-YP_004828403.1--36H-COG1/1-554   NTLVKTRVTVTRVAIRYLKNQR--DPASLAAIN---KLLGTASTSLGNAETYYSAWQK--
En.bac.2261-D782_1748-YP_007339929.1--36H-COG1/1-547    DLMLQTRINLSRSSARMMMDTS--NLQSSAK-T---DLLKNAKSTLAQAQVHITRFNQ--
Es.col.1836-Y75_p1862-YP_490148.1--36H-COG1/1-553       DLMLQTRINLSRSAVRMMMDSS--NQQSNAK-V---ELLDSARKTLAQAATHYKKFKS--
Cr.tur.6-CTU_33010-YP_003211664.1--36H-COG1/1-555       QTLVKTRVTVNRVAIRFLKNQR--DPASLAAID---KLLATASTSLAKAEKHFALYKG--
Cr.tur.6-CTU_25780-YP_003210941.1--36H-COG1/1-536       NLLLQTRINLSRSAARMMMDAS--NQQSSAK-T---DLLNTAKKVLAEADTHFSAFKG--
En.clo.1544-EcWSU1_00403-YP_004950264.1--36H-COG1/1-549  ISLNQARVTLNRGMLRLQSSMA--SQINGGQLN---ELVNTAKNLLADAQSHYDKYYA--
En.638.865-Ent638_2457-YP_001177177.1--36H-COG1/1-555   DLMLQTRINLSRSSARMMMDPN--NQQSSAK-T---DLLKNARGTLADSAKHYDAFKQ--
Sa.bon.1474-SBG_1755-YP_004730609.1--36H-COG1/1-553     DLMLQTRINLSRSAARMMMDAA--NQQSSAK-T---DLLQNAKTTLTQAAAHYTNFKN--
Pr.mir.1265-PMI1665-YP_002151396.1--36H-COG1/1-548      VSLVQARNAINRVAIGMKTEQ------STDYIQ---SIESIALSRLETANTHFQNFLTDI
Cr.sak.579-ESA_01348-YP_001437444.1--36H-COG1/1-556     NLLLQTRINLSRSAARMMMNAN--NQQSSAK-T---DLLNTAKKVLAEADTHFNAFKA--
Cr.sak.579-ESA_00560-YP_001436686.1--36H-COG1/1-555     QTLVKTRVTVNRVAIRFLKNQR--DPASLAAIN---KLLATASTSLGKAEKHFALYKG--
Sa.ent.407-STM1919-NP_460876.1--36H-COG1/1-553          DLMLQTRINLSRSAARMMMDAS--NQQSSAK-T---DLLQNAKTTLAQAAAHYANFKN--
Ci.kos.578-CKO_01066-YP_001452645.1--36H-COG1/1-552     DLMLQTRINLSRSSARMMMDAS--NQQSSAK-N---DLLKNAKSTLEQAATHYANFKN--
En.asb.1498-Entas_0424-YP_004826963.1--36H-COG1/1-549   ISLNQARVTLNRGMLRLQSSVA--TQINGGQLN---ELVETAKNLLADAQAHFDKYYA--
Pantoe.297-Pat9b_1273-YP_004115150.1--36H-COG1/1-553    QTLVKTRVTINRVAIRILKKQT--DEASLAAID---KLLTAAGASLNEAQQHFAQYKS--
Ed.tar.1771-ETAF_2264-YP_005699859.1--36H-COG1/1-555    ASITQIREEIMKDALALSAHS------DEGNAR---VSLGNLLQKLSNVDKEMENFYS--
Pa.ana.1905-PAJ_3534-YP_005936409.1--36H-COG1/1-553     QTLIKTRVTISRVAIRFLKNQK--DPASLASIA---KLLEVAKQSADKADEYFQAYKT--
Pa.vag.184-Pvag_1203-YP_003930842.1--36H-COG1/1-552     KTLIKTRVTINRVAIRFLKNQQ--DPKSLAAMA---ALLEQAGQSAEKADADFKAWQA--
Pantoe.297-Pat9b_1605-YP_004115478.1--36H-COG1/1-556    IELNQTRTVLNRAMLRMQGSMA--AQTNGGQLA---ALIAQTEKQLKAADGYFQRYYN--
Cr.sak.579-ESA_00210-YP_001436348.1--36H-COG1/1-557     ISLNQTRVTLNRGMLRLQGNMA--NQINGGQLS---QLVDTANKLLAEAQSHYDKYYG--
Er.tas.1011-ETA_pET460340-YP_001905943.1--36H-COG1/1-543 DQYFRVITRLARATDSRKP-----------------QDMDAAKQSLENMTALLAEFKA--
Cr.tur.6-CTU_36610-YP_003212024.1--36H-COG1/1-557       ISLNQTRVTLNRGMLRLQGNMA--SQINGGQLN---ELVTTASQQLADAKSHYDKYFK--
Pe.car.1139-PC1_1159-YP_003016743.1--36H-COG1/1-556     IMMNQTRIAIGSIIQNMMMGNA-----DKEAMQ---ALLQQAKTQLAASESSYKHYLS--
Er.tas.1011-ETA_14640-YP_001907403.1--36H-COG1/1-554    ITLNQTRIVLNRGMLRIQSEAA--NAGTSASLT---VLSNRAKQLLNEAQAFHAAYKA--
Pa.vag.184-Pvag_1725-YP_003931362.1--36H-COG1/1-556     MSLNQSRVLLTRIQLRIATSKL---EGKTADIA---GLFGESKGFQQAADGYYKEFKA--
Ed.ict.1187-NT01EI_3742-YP_002935104.1--36H-COG1/1-525  QTLVMTRVTINRVAIRYLKQQN--AGVAQLEIG---SLLNAANASLDSAEGYFKNYRA--
En.638.865-Ent638_0380-YP_001175119.1--36H-COG1/1-549   ISLNQARVTLNRGMLRLQSSAA--SQINGGQLS---ELVATAKNLLANAQGHYDKYYA--
Er.bil.197-EbC_25360-YP_003741914.1--36H-COG1/1-556     IALNQTRITLNRGMLRLQSETA--NSINSASLT---AISDQGKKLLADADKSYARYQA--
Er.tas.1011-ETA_16910-YP_001907630.1--36H-COG1/1-543    DQYFRVITRLARATDSKKP-----------------QDMDAAKQSLENMTALLAEFKA--
Es.fer.1173-EFER_3055-YP_002384154.1--36H-COG1/1-603    VALLQTRNTLNRAGIRYMMDQN--NIGSGATVT---ELMQLASISLKQAEKNWAEYEA--
Xe.nem.162-XNC1_1619-YP_003711880.1--36H-COG1/1-520     AALLQTRNTLNKIAIAQKVGQ------PQTQID---SMEALLKSSLNEAERSFARFIS--
En.clo.1544-EcWSU1_02811-YP_004952664.1--36H-COG1/1-559  DLMLQTRINLSRSSARMMMDPN--NQQSSAK-T---ELLKNARATLADAAKHYDAFKK--
Di.dad.235-Dda3937_03500-YP_003882750.1--36H-COG1/1-553  DQYFRAVTRMLRAVDYLQTG-------DADNAQ---KTLSSAASALKNSEEALAQFKS--
Xe.bov.105-XBJ1_1924-YP_003467828.1--36H-COG1/1-523     AAMLQTRNTLNKIAIGQKVGQ------SQDQID---AMEALLKSSLNEAERNFAQFSA--
Rahnel.1320-Rahaq_1831-YP_004212576.1--36H-COG1/1-556   SYLLQTRNTLNRAATRLALKQ------PQENIT---QLMSDAKASLKKAEDAYAAFLA--
Ra.aqu.1678-Q7S_08965-YP_005401598.1--36H-COG1/1-556    SYLLQTRNTLNRAATRLALKQ------PQENIT---QLMSDAKASLKKAEDAYAAFLA--
Cr.sak.579-ESA_01126-YP_001437230.1--36H-COG1/1-514     DQYFRFVTRLTRAMEN-----------GTPDA----KSLESAQQALDNMSRQLDAFKQ--
Pe.atr.485-ECA2712-YP_050803.1--36H-COG1/1-560          DQYFRTVTRLLRSMDFQQTG-------ETAEAE---KVFTSATTAFKNTSDQLAAFKA--
En.clo.1544-EcWSU1_00560-YP_004950421.1--36H-COG1/1-597  VALLQTRNTLNRAGIRYMMDQS--NIGSGATVN---DLMQIAAASLKQAEKNWAEYEA--
Di.dad.235-Dda3937_03498-YP_003882752.1--36H-COG1/1-561  DQYFRSVTRMARVMDYSQFS-------DTENAK---KTLDSALIAINNTKGALEKFKA--
Cr.tur.6-CTU_27880-YP_003211151.1--36H-COG1/1-514       DQYFRFVTRLTRAMEN-----------GTPDA----KSLESVQQALDNMSRQLDAFKQ--
Pe.car.1139-PC1_1669-YP_003017246.1--36H-COG1/1-560     DQYFRTVTRLLRSMDFQQTG-------ETAEAE---KIFTSATTAFKNTSDQLAAFKA--
Ra.aqu.1678-Q7S_14450-YP_005402672.1--36H-COG1/1-551    AQYYRAVTALERAIAGLEKN-------NNGIYD---IEIKATLIELESLKKGLDQFKN--
Ci.kos.578-CKO_03622-YP_001455137.1--36H-COG1/1-517     ISLNQARVTLNRGMLRLQSSMA--NEINGGQLN---ELVKTANALLADAQRHYDKYYA--
Di.zea.1140-Dd1591_2408-YP_003004728.1--36H-COG1/1-557  DQYSRAVTRMSRIPEFIQQG-------DMDNAQ---KTLESASEALKHAKAGLAQFRN--
Di.zea.1140-Dd1591_2406-YP_003004726.1--36H-COG1/1-559  DQYFRSVTRMARVMDYLQFN-------DTENAK---KTLDMALTAINNTKGALGKFKA--
Ed.ict.1187-NT01EI_2801-YP_002934203.1--36H-COG1/1-554  ASITQIREEIMKDALALSIHN------EEGNAH---VSLGNLLQKLNHVDKDMEKFYS--
Pe.car.1139-PC1_1384-YP_003016966.1--36H-COG1/1-559     DQYFRAVTRMERTMDYLQRN-------EPENAK---QTLDMAQIALKNTKDSLEKFQT--
Rahnel.1320-Rahaq_2866-YP_004213595.1--36H-COG1/1-551   AQYYRAVTALERAIAGLEKN-------NNGIYD---IEIKATLIELESLKKGLDQFKN--
Pe.atr.485-ECA1281-YP_049387.1--36H-COG1/1-556          IMMNQTRIAIGSIIQNMMLGNA-----DKEAMQ---ALLQQAKSQLVASESSYKHYLS--
Pectob.2320-W5S_1886-YP_006282848.1--36H-COG1/1-549     DQYFRTVTRLLRSMDFQQTG-------ETADAE---KVFTSATTALKNTSDQLNAFKA--
Pectob.2320-W5S_1696-YP_006282659.1--36H-COG1/1-559     DQYFRAVTRMERTMDYLQRN-------EPDNAK---QTLEMAQIALKNTRDSLEKFQT--
Di.zea.1140-Dd1591_2407-YP_003004727.1--36H-COG1/1-555  DQYFRSVTRMLRAVDYLQTG-------NKEDAQ---KTLSSADTALKNSQDALAEFKN--
Pe.atr.485-ECA1509-YP_049611.1--36H-COG1/1-559          DQYFRAVTRMERTMDYLQRN-------EPENAK---QTLEMAQRAIKNTKDSLEKFQA--
Pa.vag.184-Pvag_1724-YP_003931361.1--36H-COG1/1-520     IALNNSRTTLNRAMLRLQTSMA--SQMNGGQLD---DLMNKANAQLVEADRHFKIYYD--
Pa.ana.1905-PAJ_1541-YP_005934417.1--36H-COG1/1-520     MALNQSRTTLNRAMLRLQTSLA--SQMNGGQLD---SLTEKAVNEANKGEQYLAEFNA--
Mo.mor.2189-MU9_1596-YP_007505015.1--36H-COG1/1-519     DQYFRFISRLNREMQKKAEG-------EEPD-------LKPSAEAMENMGLHLAEMKR--
Pr.mir.1265-PMI2808-YP_002152509.1--36H-COG1/1-563      AALNTLRTDIIAQAFSYIANPQ----VEDANIT---TFMQTMDARKAEVDTLMNEYVE--
Di.zea.1140-Dd1591_0395-YP_003002764.1--36H-COG1/1-556  DRYYKVKLTMDEALAAED---------NSSRER---ALLNEAAGDVEFLKGGLAQFKV--
En.clo.1544-EcWSU1_03786-YP_004953635.1--36H-COG1/1-515  DQYFRFVTRLSRAMEVKAAG-------GT-------PDLAPAQQALDNMGKKLAEMKA--
Pectob.2320-W5S_3170-YP_006284116.1--36H-COG1/1-556     IMMNQTRIAIGSIIQNMMMGNA-----DKEAMQ---ALLQQAKSQLVASESSYKYYLS--
Di.dad.235-Dda3937_02184-YP_003884754.1--36H-COG1/1-556  DRYYKVKLSMDEAAVVEN---------NSDRVL---ALLNDAAADVDFLKGGLAQFKV--
Ci.rod.62-ROD_32641-YP_003366748.1--36H-COG1/1-517      ISLNQARVTLNRGMLRLQGNMA--GQINGGQLN---ELVQTTDALLAEAKNHYDKYYA--
Di.dad.235-Dda3937_03499-YP_003882751.1--36H-COG1/1-561  DQYFRSVTRMMRVVDYRQSG-------DTDNAQ---KTLTSATTALKNSEEALAQFRS--
Di.dad.235-Dda3937_00105-YP_003883346.1--36H-COG1/1-561  IALIQTRNTMNRAIARMIMETN--NIPSAGKSA---DLIDVANQSLTEADKHFAAYSQ--
Pantoe.297-Pat9b_1604-YP_004115477.1--36H-COG1/1-555    MSLNQSRVLLTRIMLRMANSKL---SGETADLT---SLYQQSKGFQDKAADNYQLFKN--
Er.tas.1011-ETA_14660-YP_001907405.1--36H-COG1/1-560    IALVQTRNTLNRAATRHLLDEK--GQGTGSSVD---ELVALAKTQLNTAEQQFAAYRQ--
Pr.mir.1265-PMI2809-YP_002152510.1--36H-COG1/1-575      AALNKLREDMLNVMFDAHLTPD----LAESKIT---DFLSSYPQRKKEVSSIITQYFT--
Ed.tar.1771-ETAF_3053-YP_005700648.1--36H-COG1/1-516    QTLVMTRVTINRVAIRYLKQQN--AGVAQAEIG---PLLDAANASLDSAEGYFKNYRA--
Se.pro.864-Spro_1415-YP_001477647.1--36H-COG1/1-546     ASVTELREEVLKDALMLTTQP------QSNDTK---KSLGELRQRLAEVDKLMEKFYR--
Di.zea.1140-Dd1591_1804-YP_003004134.1--36H-COG1/1-562  IALIQTRNTMNRAIARMIMETN--NIPSAGKSA---DLIDVANQSLAQADQHFAAYKQ--
Di.dad.235-Dda3937_03501-YP_003882749.1--36H-COG1/1-575  DQYSRAVTRMSRIPELIQQG-------EMDNAQ---KTLISATDALKNTKEALTQFKH--
Pantoe.297-Pat9b_0851-YP_004114731.1--36H-COG2/1-524    RDGVNDQIKYLRGIVIDT---------HRPENNV--KRFQQLAEATDRTNSAMKKIEA--
Pa.vag.184-Pvag_0291-YP_003929953.1--36H-COG2/1-525     RDGVNDQIKYLRGMVIDT---------NRPEMNV--KRYGQLDEATQRTNIAMKKIED--
En.clo.1544-EcWSU1_03886-YP_004953735.1--36H-COG2/1-519  IDNFQEFIGTQQLMLLDE---------QGTYTA---QSQQRLKAISEQITVILGELNT--
Cr.tur.6-CTU_04790-YP_003208842.1--36H-COG2/1-515       IENFQSFVSTQQLMLMDE---------NQRWTQ---QSEAQLSAISGRISALLADLNK--
Ci.kos.578-CKO_04484-YP_001455975.1--36H-COG2/1-521     IDNFQDFVSTQQLMLLDE---------EGRWSQ---ESQKQLDAISQRITVLLDELSA--
Cr.sak.579-ESA_03488-YP_001439539.1--36H-COG2/1-515     IENFQSFVSTQQLMLMDE---------NQRWTQ---QSEAQLSAISGRIGALLADLNK--
Ci.rod.62-ROD_48071-YP_003368199.1--36H-COG2/1-521      IDNFQEFVTTQQQMLMDE---------QGRWTE---ESQAKLNAVSQQISQLLDELNA--
Sa.bon.1474-SBG_2851-YP_004731664.1--36H-COG2/1-521     IDNFNDFIIAQQLMLLDE---------EGRWSQ---SSQKELDEISQRISALLDELSR--
Sa.ent.407-STM3216-NP_462130.1--36H-COG2/1-521          IDNFNDFIIAQQLMLLDE---------EGRWSQ---SSQKELDEISQRITALLDELSS--
En.asb.1498-Entas_3753-YP_004830248.1--36H-COG2/1-519   IDNFQEFISTQQLMLLDE---------QGKYTA---QSQQHLKEISEHITVILGDLNN--
Mo.mor.2189-MU9_3389-YP_007506806.1--36H-COG2/1-521     IDYFHDFVGIQQLLLLDD---------KNPQIR---QLAAGASETSKKITILIDEFNE--
Sa.ent.404-STY3394-NP_457606.1--36H-COG2/1-522          IDNFNDFIIAQQLMLLDE---------EGRWSQ---SSQKELSEISQRISALLDELSR--
En.638.865-Ent638_3526-YP_001178235.1--36H-COG2/1-519   IENFQEFVSTQQIMLLDE---------QGRYTE---QSQQHLKELSERITVILTDLNR--
Pe.atr.485-ECA1774-YP_049874.1--36H-COG2/1-554          IDELNGYLNNQQLLLLLK---------SESEIN---KQLALNKERSGKISELMEYLNQ--
En.638.865-Ent638_2119-YP_001176845.1--36H-COG2/1-549   INLVDRNSGNTIAAMATQ---------DPKLRE---QILNNIKNDTARTSEVQSTLKP--
```

```
Pectob.2320-W5S_2813-YP_006283770.1--36H-COG2/1-554       IDELNGYLNNQQLLLLLK---------SESEIN---KQFALNKERSGKISELMEYLNQ--
Di.zea.1140-Dd1591_0695-YP_003003055.1--36H-COG2/1-561    IEDVNEAVRISQQLMLVS---------GDDRIK---AVSDQIPPLSKDITRLMDALVKHA
Pa.vag.184-Pvag_pPag30079-YP_003729820.1--36H-COG2/1-546 RYYSTDLSRLVRNVVLAD---------APDRKA---VFKKDYDRVHDKITATVDQVDA--
Er.bil.197-EbC_38940-YP_003743272.1--36H-COG2/1-526       RDGVNDQIKFLRGMVIDT---------TRPQYNV--KRFGQLTDATDKTNLAVGKIAQ--
Ra.aqu.1678-Q7S_24891-YP_005419232.1--36H-COG2/1-554      RDGVNDQIKYLRGIVIDT---------KNPEQNK--KRYLQLDDTVKKTNLAMDKIAA--
En.asb.1498-Entas_2287-YP_004828805.1--36H-COG2/1-513     IEVFQAK--INRLKYLYNGDE---------------KSGQLLAGYVKHAEELTAEAKK--
Rahnel.1320-Rahaq_4869-YP_004215574.1--36H-COG2/1-554     RDGVNDQIKYLRGIVIDT---------KNPEQNK--KRYLQLDDTVKKTNLAMDKIAA--
Di.dad.235-Dda3937_00027-YP_003883574.1--36H-COG2/1-554   IDELNNALLGQQLILMLD---------SNADIK---RKNEEIKGYSAEITALLADLSK--
Pr.stu.1965-S70_05790-YP_006215722.1--36H-COG2/1-520      MDNFYSYIGIQELILLDD-----------RDSE---RRRNELKKISSNVVRLLKELDE--
En.638.865-Ent638_2100-YP_001176826.1--36H-COG2/1-512     IEVFQAK--INRLKFLYNGDE---------------KSGQLMAKYVKHAAEITAEAKT--
Pe.car.1139-PC1_2526-YP_003018093.1--36H-COG2/1-554       IDELNGYLNNQQLLLLLK---------SESEIN---KQLALNKERSGKISELMEYLNQ--
Mo.mor.2189-MU9_702-YP_007504121.1--36H-COG2/1-525        RHATEERAIMLRDMINATDKKD-----KTEELN---RFKEEDGKVSKRTQTLLDMVNE--
Pe.car.1139-PC1_3443-YP_003018995.1--36H-COG2/1-511       DKTVVAREGNFGYALER----------KAQYLE---QHDNAIGNINDELSALLKEIAT--
Pa.ana.1905-PAJ_0915-YP_005933791.1--36H-COG2/1-559       RYYTSDMSRLARNAILLK---------DVAKRE---KAISDYRGERHEVDSLLSLIGK--
Pectob.2320-W5S_3736-YP_006284671.1--36H-COG2/1-510       DKTVVAREGNFGYALER----------KAQYLE---QHDNAIGNISGELSALLKEIDT--
Pe.atr.485-ECA0183-YP_048310.1--36H-COG2/1-553            IDNVTSILVAYQRLMLVS---------DSVQIQ---TNVTRVNEYRQEIGRLLDKLER--
Rahnel.1320-Rahaq_3107-YP_004213828.1--36H-COG2/1-503     --------KFTRLSYMASKDP---------------KFVEENRQALDKLEARQADVEA--
Pe.car.1139-PC1_4071-YP_003019622.1--36H-COG2/1-553       IDNVNSILVAYQRLMLVS---------DQTQIQ---TNVARVNEFRQEIGRLLDKLES--
Di.dad.235-Dda3937_03462-YP_003884507.1--36H-COG2/1-563   IDNVNEGVRVFQQLLLVS---------GDDRIK---AVSDQIPPISKEITRLMDALEKHA
Ra.aqu.1678-Q7S_15665-YP_005402913.1--36H-COG2/1-503      --------KFTRLSYMASKDP---------------KFVEENRQALDKLEARQADVEA--
Pectob.2320-W5S_4505-YP_006285423.1--36H-COG2/1-555       IDNVTSILVAYQRLMLVS---------GEEQIQ---TNVTRVNEFRQEIGRLLEKLES--
Pe.atr.485-ECA3642-YP_051730.1--36H-COG2/1-551            HATLVAG--VQRSMAVARSN-------DDSLVE---LFAAENTRASKESGKRQEDFAS--
Pe.car.1139-PC1_1417-YP_003016999.1--36H-COG2/1-554       QDILNTNVRTLRDIILLPAD-------RNQEKQ---ALRAVIVKTTNSASEIYKNLDG--
En.asb.1498-Entas_4509-YP_004821569.1--36H-COG2/1-559     IDGVNKNYTLTYQAVAAT---------DDNERK---LIISELDKQTALNHELMQQLKKD-
Pa.ana.1905-PAJ_3064-YP_005935940.1--36H-COG2/1-544       SFYAQDMSRLARIAVLQP---------DPAKTE---KALNSYQQVRSQADSLMQVLDK--
Pectob.2320-W5S_1733-YP_006282696.1--36H-COG2/1-535       QDILNTNVRTLRDIILLPGD-------KVQEKQ---ALKAIIVKTTNSAGEIYKTLDS--
Pe.atr.485-ECA4334-YP_052421.1--36H-COG2/1-556            KDNINTNAIAVRNLTMQE---------DDRLVQ---EEKTRIEEMISRNNALLSKIHD--
Di.zea.1140-Dd1591_1602-YP_003003935.1--36H-COG2/1-554    IDELNDALLGQQLVLMLD---------TNADIQ---QKNKETKNHSAVITNLLADLGK--
Pectob.2320-W5S_4506-YP_006285424.1--36H-COG2/1-540       IDNVNGHFVAYLQMMLVS---------GQEQRQ---GYVDRIMAYRKEISRLLDNLES--
Di.zea.1140-Dd1591_1453-YP_003003794.1--36H-COG2/1-532    EWLVQRNRVLIMDMLLLP---------NTLNKQ---QSTDELNQNMSHIGQVWAEYRA--
Pectob.2320-W5S_0106-YP_006281117.1--36H-COG2/1-556       KDNINANAIAVRNLVLLE---------DERQMQ---EEKARIEELISRNNALLLKIDD--
Di.zea.1140-Dd1591_0769-YP_003003127.1--36H-COG2/1-505    DKTVVAREANFGYALER----------KAQYLE---QHDTAINTISQELTALLQEIRA--
Ra.aqu.1678-Q7S_18035-YP_005403387.1--36H-COG2/1-535      LSGVKDSSLNIYGLVLVS---------EAADIE---AGKRQVAAAEEKTAAALNRLQAAI
Rahnel.1320-Rahaq_3579-YP_004214298.1--36H-COG2/1-535     LSGVKDSSLNIYGLVLVS---------EAADIE---AGKRQVAAAEEKTAAALNRLQAAI
Pe.car.1139-PC1_4072-YP_003019623.1--36H-COG2/1-540       IDNVNGHFVAYLQMMLVS---------GQEQRQ---GYVDRIMAYRKEISRLLDNLER--
Pe.car.1139-PC1_3464-YP_003019016.1--36H-COG2/1-551       HATLVAG--VQRSMAVARSN-------DDSLVE---LFAAENTRASKESGKRQEQFAS--
Pe.car.1139-PC1_0111-YP_003015709.1--36H-COG2/1-556       KDNINTNAIAIRNLTMQE---------DSRQMQ---QEKVRIEEMVSRNNALLTKIHD--
Pe.atr.485-ECA0182-YP_048309.1--36H-COG2/1-542            IDNVNGHFVAYLQMMLVS---------GQEQRQ---GYVDRIMAYRKEIGRLLDNLES--
Pectob.2320-W5S_3753-YP_006284688.1--36H-COG2/1-551       HATLVAG--VQRSMAVARSN-------DDSLVE---LFAAENTRASKESAKRQEDFAS--
Pa.ana.1905-PAJ_2666-YP_005935542.1--36H-COG2/1-514       YGGLRDMAVIVRNLALLT---------EADQMQKEWQRIVTQKARYIENRERLAAMVA--
Ci.rod.62-ROD_16161-YP_003365194.1--36H-COG6/1-557        NHLRTARINMIHAGAASRIA-------EMNDVK---ANIAAAEKRMKQSEEGFNAYMA--
En.asb.1498-Entas_2095-YP_004828614.1--36H-COG6/1-563     NHLRTARINMIHAGAASRIA-------EMDAMK---QNIHEAETRIKQSQESFASYMN--
En.638.865-Ent638_1961-YP_001176688.1--36H-COG6/1-563     NHLRTARINMIHAGAASRIA-------EMDAMK---QNIAEAETRIKQSQTSFAMYMN--
Cr.sak.579-ESA_01710-YP_001437800.1--36H-COG6/1-565       NQLRTARINMIHAGAASRVA-------EMDAMK---RNIADAEKAIAQANAGFKQYVD--
Cr.tur.6-CTU_22440-YP_003210607.1--36H-COG6/1-565         NQLRTARINMIHAGAASRVA-------EMDAMK---RNIADAEKAIAQANVGFKQYVD--
Ci.kos.578-CKO_01456-YP_001453025.1--36H-COG6/1-562       NHLRTARINMIHAGAASRIA-------EMDDMK---ANIAEAEKRIKQSQEGFNVFMA--
Rahnel.1320-Rahaq_1930-YP_004212674.1--36H-COG6/1-566     NHLRTARLLLIQAAAAARIG-------DQDVFS---QNLKDAASRIKQSQTSFDMYIN--
Pantoe.297-Pat9b_2403-YP_004116261.1--36H-COG6/1-564      NHLRTARLNVIQSGAAARIG-------EMDSYR---ADLARTEQRIEQARAGFKLYMD--
Er.bil.197-EbC_26700-YP_003742048.1--36H-COG6/1-567       NHLRTARLNIIHAGASARIG-------EMDAFN---DNVKQTEKRIQQATESFNAYLN--
Pectob.2320-W5S_1760-YP_006282723.1--36H-COG6/1-570       NHLRTARLLLIQAAAAARIG-------DSQVFN---DNLTQAQQRLEQSKKAFLVYEQ--
Pa.ana.1905-PAJ_1621-YP_005934497.1--36H-COG6/1-568       SHMRSARLNIIQAGAAARIG-------EMDEFN---ANLAATADRIKQAEAGLKIYLN--
En.clo.1544-EcWSU1_02120-YP_004951978.1--36H-COG6/1-576   NHLRTARINMIHAGAASRIA-------EMDAMK---QNISEAEKRIKQSQDGFNAYMN--
Ra.aqu.1678-Q7S_09815-YP_005401764.1--36H-COG6/1-566      NHLRTARLLLIQAAAAARIG-------DQDVFS---QNLKDAASRIKQSQTSFDMYIN--
Ye.pse.585-YPTB2412-YP_070925.1--36H-COG6/1-579           NHLRTARLILIQAASSARIG-------DATGYQ---QGLKNAEGRIAQSQQMFDLYYN--
Ye.ent.378-YE2588-YP_001006789.1--36H-COG6/1-579          NHLRTARLILIQAASSARIG-------DATGYQ---QGLKNAENRISQSQQMFNLYYN--
Di.dad.235-Dda3937_02787-YP_003883649.1--36H-COG6/1-572   NHLRTARLLIIQAGAAVRVG-------DTEVFN---NNLKQAEQRIASSKDAFKVYEN--
Pa.vag.184-Pvag_1800-YP_003931435.1--36H-COG6/1-568       NHLRTARLTIIQAGAAARIG-------DMDEFR---ANVAAAEKRIQQAKDGFAVYQA--
En.bac.2261-D782_2307-YP_007340470.1--36H-COG6/1-558      NHLRTTRIELIHAGAASRIA-------EMDEMK---RNIAMAESRMQQSQTAFTQYRE--
Pe.atr.485-ECA1683-YP_049784.1--36H-COG6/1-573            NHLRTARLLLIQAAAAARIG-------DSQVFN---DNLKQAEQRLDQSKKAFLVYEE--
Pe.car.1139-PC1_2617-YP_003018183.1--36H-COG6/1-573       NHLRTARLLLIQAAAAARIG-------DSQVFN---DNLKQAEQRLEQSKKAFLVYEE--
Di.zea.1140-Dd1591_1535-YP_003003868.1--36H-COG6/1-574    NHLRTARLLIIQAGAAVRVG-------DTDVFN---NNLKQAEQRIASSKDAFQVYEN--
Di.zea.1140-Dd1591_3580-YP_003005868.1--36H-COG6/1-572    NHLRTARLMLIQAAAAAKEG-------DKDTAN---SSIQQAEGRLKQSADSFAAYQS--
Es.col.1836-Y75_p1397-YP_489687.1--36H-COG6/1-546         DFLRSARINMIQAGAASRIA-------EMEAMK---RNIAQAESEIKQSQQGYRAYQN--
Pe.atr.485-ECA3902-YP_051990.1--36H-COG6/1-575            DQLRVARLLLIQAGAANRIS-------DHEVFK---AASDQASGRVRASQKRLDEYLA--
Sa.bon.1474-SBG_1454-YP_004730321.1--36H-COG6/1-537       NHLRTARINMIHAGAASRIA-------EMDEMK---ANIAAAEMQIKQSQDGFNAYMS--
Sa.ent.407-STM1626-NP_460585.1--36H-COG6/1-541            NHLRTARINMIHAGAASRIA-------EMDEMK---ANIAAAETRIKQSQDGFNAYMS--
Pe.car.1139-PC1_3679-YP_003019230.1--36H-COG6/1-567       DQLRVARLLLIQAGAANRIS-------DHEVFK---SASDQAAGRVRASQKRLDEYLA--
Pectob.2320-W5S_4020-YP_006284955.1--36H-COG6/1-575       DQLRVARLLLIQAGAANRIS-------DHEVFK---AASDQAAGRVRASQKRLDEYLA--
Di.zea.1140-Dd1591_0523-YP_003002884.1--36H-COG6/1-568    DNFRVSRQLLIQAVASSRIG-------DTDSFK---QAFAEGAERIKSSQKRFDDYMA--
Di.dad.235-Dda3937_02665-YP_003881279.1--36H-COG6/1-572   NHLRTARLMLIQAAAAAKDG-------DKDTAS---SSIQQAEGRLKQSADSFAAYQS--
```

```
Pr.mir.1265-PMI1666-YP_002151397.1--36H-COG1/1-568         -HINAYQIHSE--EEIRNLKGRFIEYFS---ALNQLEILLQDKNLKDFFEQPT---TSYQ
Di.dad.235-Dda3937_02779-YP_003883640.1--36H-COG1/1-566    --IPYTNQQDP--AVAQVVKDNYTVLNS---ALSDLIVFISNGKLKEFFDQPT---QSFQ
Di.zea.1140-Dd1591_1542-YP_003003875.1--36H-COG1/1-566     --IPYTDQQDP--AIAQVVKDNYTALNS---ALSELIVFISTGKLKEFFDQPT---QGFQ
Pe.atr.485-ECA1691-YP_049792.1--36H-COG1/1-561             --MPQDARQDD--SISRGVKENYVALNA---ALTELIQFLNAGEFKKFIEQPT---QRFQ
Se.pro.864-Spro_2983-YP_001479212.1--36H-COG1/1-556        --IPLNARQDL--QSAEKLKQQYGILHG---ALTELTVLLGEGKINAFFDQPT---QSYQ
Ph.asy.1114-PAU_02685-YP_003041519.1--36H-COG1/1-564       --TAKLPIHET--ESLNRLRKAYDVYIS---ALHDLIVLIEHDRAVEFFNQPT---SKYQ
Pr.stu.1965-S70_18135-YP_006218124.1--36H-COG1/1-561       --LMAETSHDK--TKFEQLFSTYETYRD---ALIQLGKYAQKGQLDEFYAHRT---SGYQ
Mo.mor.2189-MU9_1762-YP_007505181.1--36H-COG1/1-561        --MMEGTSYDK--AKFEKLLSTYETYRN---ALTRLMSYVREDQLEQFYTYAT---SGYQ
Pe.car.1139-PC1_2609-YP_003018175.1--36H-COG1/1-561        --VPQDARQDE--AVTRGVKENYVALNG---ALTELIQFLNAGEFKKFVEQPT---QSFQ
Xe.nem.162-XNC1_1620-YP_003711881.1--36H-COG1/1-567        --LPKLSIHDP--ERLQTLKQTYRDYLN---ALKELDILLTKKDFKAFFEQPT---TGFQ
Pectob.2320-W5S_1768-YP_006282731.1--36H-COG1/1-561        --LPQDARQSE--SVTRDVKENYVALNG---ALIELIQFLNAGEFKKFIDQPT---QRFQ
Pa.vag.184-Pvag_1723-YP_003931360.1--36H-COG1/1-558        --NLSEQGKTA--ENVVALQANYKAYHD---ALAELMVFFTTGNFKGFVDQPT---QSFQ
Ye.pes.133-YPZ3_2024-YP_003568196.1--36H-COG1/1-557        --IPQNSSQDP--EGAKKLKQSYEEYFS---ALTELINLMETNKINEFFNHPT---TSFQ
Ye.pse.585-YPTB2401-YP_070915.1--36H-COG1/1-557            --IPQNSSQDP--EGAKKLKQSYEEYFS---ALTELINLMETNKINEFFNHPT---TSFQ
Ye.ent.378-YE2575-YP_001006778.1--36H-COG1/1-557           --IPLDSQQDP--ESAKKLKQTYEQYFG---ALTELIQLMEAAKINEFFDQPT---SSFQ
Pa.ana.1905-PAJ_1540-YP_005934416.1--36H-COG1/1-563        --NLSEKGKSA--ENVLTLQANYKQFHD---GLAELIVFLGNGNFKGFVDQPT---QSYQ
Ph.lum.1262-plu1853-NP_929126.1--36H-COG1/1-564            --TAKLPIHES--ESLNRLKKAYDVYIS---ALHELIVLLEHDRAVEFFNQPT---GKYQ
Serrat.1901-SerAS13_3075-YP_006025914.1--36H-COG1/1-555    --IPLSPRQDA--QSAEKLKQQYGILHG---ALSELVQLLGEGKINAFFDQPT---QSYQ
Sa.ent.404-STY2128-NP_456485.1--36H-COG1/1-553             --MTPLPAM-A--EASANVDEKYQRYQA---ALAELIQFLDNGNMDAYFAQPT---QGMQ
Ra.aqu.1678-Q7S_08960-YP_005401597.1--36H-COG1/1-564       --IPADASQNP--QFAADAKKQYQVLHD---ALSELIELIGLGRINDFFEQPT---QQYQ
Se.mar.2260-D781_2759-YP_007345195.1--36H-COG1/1-555       --VPLGERQDR--QDAEKLKQQYGVLYG---ALSELIQLLSDGKINAFFDQPT---QSYQ
Rahnel.1320-Rahaq_1830-YP_004212575.1--36H-COG1/1-564      --IPADASQNP--QFAADAKKQYQVLHD---ALSELIELIGLGRINDFFEQPT---QQYQ
Se.ply.1407-SerAS9_3072-YP_004506452.1--36H-COG1/1-555     --IPLSPRQDA--QSAEKLKQQYGILHG---ALSELVQLLGEGKINAFFDQPT---QSYQ
Ed.ict.1187-NT01EI_1461-YP_002932882.2--36H-COG1/1-555     --LPRDPRQSE--EAMEAMEQNYGTLHN---ALGELIQLLSAGHINEFFEQPT---QSYQ
En.aer.1436-EAE_15535-YP_004593297.1--36H-COG1/1-556       --IPQDPRQDP--QATEKLKQQYGILYG---ALSELIQLLGEGKINAFFDQPT---QKYQ
Xe.bov.105-XBJ1_1923-YP_003467827.1--36H-COG1/1-567        --LKKLPIHDP--DHLQRLKQTHHEYLN---ALKELDGFLAKKQYKEFFAQKT---TDYQ
Serrat.1408-SerAS12_3073-YP_004501499.1--36H-COG1/1-555    --IPLSPRQDA--QSAEKLKQQYGILHG---ALSELVQLLGEGKINAFFDQPT---QSYQ
Er.bil.197-EbC_25350-YP_003741913.1--36H-COG1/1-559        --NLSEKGKTA--ENVKQLHANYKQLHD---ALADLIVMMQTGKFTDFIAQPT---QSYQ
Er.tas.1011-ETA_14650-YP_001907404.1--36H-COG1/1-554       --HLSEAGKTS--ANVQAVQKNYAILHD---ALSELIPLIGSGKITEFFGQPT---QRYQ
Sa.ent.407-STM4533-NP_463392.1--36H-COG1/1-553             --LPRDPRQSE--AAFLEIKRTYDIYHG---ALAELIQLLGAGKINEFFDQPT---QSYQ
Es.col.1836-Y75_p4240-YP_492486.1--36H-COG1/1-551          --LPRDPRQST--AAAAEIKRNYDIYHN---ALAELIQLLGAGKINEFFDQPT---QGYQ
En.bac.2261-D782_3913-YP_007342002.1--36H-COG1/1-553       --MPRDPRQSE--SAALEIKRNYDIYHG---ALAELIQLLGAGKINDFFDQPT---QGYQ
Ci.kos.578-CKO_03442-YP_001454958.1--36H-COG1/1-554        --LPRDPRQSE--TAAVEIKRNYDIYHN---ALAELIQLLGAGKINDFFDQPT---QGYQ
Cr.sak.579-ESA_03402-YP_001439456.1--36H-COG1/1-555        --LPRDPRQSE--EYAQEIKRNYDIYHG---ALAELIQLLAAGKINEFFDQPT---QKYQ
Pantoe.297-Pat9b_1606-YP_004115479.1--36H-COG1/1-558       --NLSEKGKTA--ENVLTLQANYNAYHG---ALQELIDFLNTGNFKGFVDQPT---QGFQ
Cr.tur.6-CTU_05670-YP_003208930.1--36H-COG1/1-555          --LPRDPRQSE--EYAQEIKRNYDIYHG---ALAELIQLLAAGKINEFFDQPT---QKYQ
Sa.bon.1474-SBG_3944-YP_004732724.1--36H-COG1/1-553        --LPRDPRQSE--AAFLEIKRTYDIYHG---ALAELIQLLGAGKINEFFDQPT---QSYQ
En.asb.1498-Entas_0547-YP_004827084.1--36H-COG1/1-554      --LPRDPRQSD--AAAMEIKRNYDIYHG---ALAELIQLLGAGKINAFFDQPT---QSYQ
Ci.rod.62-ROD_48461-YP_003368233.1--36H-COG1/1-554         --LPHDPRQSE--AASAEIKRNYITFHN---ALTKLNQLVGAGKVNEFFEQPT---QSYQ
Ed.tar.1771-ETAF_1249-YP_005698855.1--36H-COG1/1-529       --LPRDPRQSD--EAVQAMSQSYDTLHS---ALGELIQMLTAGRINDFFEQPT---QSYQ
En.638.865-Ent638_0513-YP_001175251.1--36H-COG1/1-586      --LPRDPRQSD--AAAQEIKRNYDIYHG---ALAELIQLLGAGKINDFFDQPT---QSYQ
Ed.ict.1187-NT01EI_1462-YP_002932883.1--36H-COG1/1-532     --YSDGNDEER--ALSQDTQQHYRALRT---ALLQLIDLVEKGDLQGFMDHPT---QQIQ
Ye.ent.378-YE2573-YP_001006777.1--36H-COG1/1-545           --VPRLDESDTGGELLDATKNSYQNLRS---SLRELIDFLEAGNLQSFMDQPT---QKTQ
Ed.tar.1771-ETAF_1250-YP_005698856.1--36H-COG1/1-530       --CSDSDEEDR--ALSQEALQHYQALRT---ALLQLVSLLEKGDLQGFMDQPT---QRIQ
Mo.mor.2189-MU9_1763-YP_007505182.1--36H-COG1/1-524        --QPVLSEKED----FAGIEEQYQSYLG---VIKELRGYIDNANIPAYVEHPT---QMYQ
En.aer.1436-EAE_15540-YP_004593298.1--36H-COG1/1-536       --TPRADEQEQ--QLADATRDSYENLRG---ALRELIVFLENRNLQAFMDQPT---QKIQ
En.638.865-Ent638_2456-YP_001177176.1--36H-COG1/1-533      --QEAVSEKGK--ALTAEMQKGYTQWHS---DLEHQATWLENNQLSDFLTAPV---QDSQ
Se.pro.864-Spro_2982-YP_001479211.1--36H-COG1/1-541        --VPRNSEQEQ--QLTATTKASYDRLRN---TLRELIGFLENDNLQAFMDQPT---QKTQ
Es.col.1836-Y75_p1861-YP_490147.1--36H-COG1/1-533          --MTAGNEHVR--GLQKETEKSFARWHN---DLEHQATWLESNQLSDFLTAPV---QGSQ
Serrat.1901-SerAS13_3074-YP_006025913.1--36H-COG1/1-541    --VPRHSEQEQ--QLTETTQASYNRLRS---ALRELIGFLENNNLQAFMDQPT---QKTQ
Ye.pes.133-YPZ3_2023-YP_003568195.1--36H-COG1/1-536        --VPRLDESESSGQLLDATKNSYQNLRS---ALRELIDFLEAGDLQAFMDQPT---QKTQ
Serrat.1408-SerAS12_3072-YP_004501498.1--36H-COG1/1-541    --VPRHSEQEQ--QLTETTQASYNRLRS---ALRELIGFLENNNLQAFMDQPT---QKTQ
Se.mar.2260-D781_2758-YP_007345194.1--36H-COG1/1-534       --LVPAGERSR--SLT----LRYQQLHA---ALLELSGFLASGNLQAFMDQPT---QQIQ
Ye.pse.585-YPTB2400-YP_070914.1--36H-COG1/1-536            --VPRLDESESSGQLLDATKNSYQNLRS---ALRELIDFLEAGDLQAFMDQPT---QKTQ
Se.ply.1407-SerAS9_3071-YP_004506451.1--36H-COG1/1-541     --VPRHSEQEQ--QLTETTQASYNRLRS---ALRELIGFLENNNLQAFMDQPT---QKTQ
Pr.stu.1965-S70_18140-YP_006218125.1--36H-COG1/1-521       --QPLLSEHEN----LAVIDEKYQAFLA---ILKELRALIDKNDIPGYVALPA---QQHQ
En.bac.2261-D782_1749-YP_007339930.1--36H-COG1/1-533       --IEQRSTESR--QLGERTEASFSAWRN---GLEHQATWLETNQLADFLGSPV---QKMQ
En.asb.1498-Entas_2590-YP_004829104.1--36H-COG1/1-533      --QEAVSEKGK--ALKVAMKKNFDQWHS---DLDHQATWLENNQLSDFMTAPV---QESQ
En.asb.1498-Entas_3672-YP_004830170.1--36H-COG1/1-545      ---ISPGPMDE--QVSAQVISSWQALLD--QGVAPQMQQAQQGQLDGYRQQANNVTPPLS
Ci.kos.578-CKO_01067-YP_001452646.1--36H-COG1/1-537        --TEASTEEGK--ALQAATEKSYTQWYS---DLEHQATWLESNQLSDFLTAPV---QASQ
En.clo.1544-EcWSU1_02810-YP_004952663.1--36H-COG1/1-538    --QEAGSAEVK--ALKVAMKKRFDQWHS---DLDHQATWLENNQLSDFMTAPV---QASQ
Sa.ent.407-STM3152-NP_462067.1--36H-COG1/1-547             ---LSPGPMNP--DISREVLSNWQALLE--KGVVPQMQLAQQGSLTAWSEHASTVTPALS
Pe.atr.485-ECA1332-YP_049438.1--36H-COG1/1-555             ---IDHASLDD--ALVQAVSRDWSNLIV--QGVEPLYQKAAANTLDDYQNQAKDVVPPLS
Pe.car.1139-PC1_1208-YP_003016792.1--36H-COG1/1-555        ---IDHAGLDP--ALVQAVSRDWNNLVT--QGVEPLYQKAVANALDDYQNQAKNVVPPLS
En.638.865-Ent638_3407-YP_001178118.1--36H-COG1/1-545      ---VSHGAMNE--AVFAEVISTWQALLE--QGIAPQMVKAQQGDDAGYRQQANEITPPLS
Pa.vag.184-Pvag_1413-YP_003931052.1--36H-COG1/1-554        ---LAPGPMNP--DVVDGVIQSWQKLLD--EGVTPQLQLARQASPDAYRQQANNVTPPLS
Ph.asy.1114-PAU_02684-YP_003041518.1--36H-COG1/1-544       --LPKLNEEDGSSVLLASVETSYKEYIK---ALRELSVFLEKGNYDAFLDQPT---EKYQ
Pa.ana.1905-PAJ_1316-YP_005934192.1--36H-COG1/1-556        ---LSPGPMDA--AVANDVIDKWQKLLD--QGVTVQLQRARQDSLDSYRQQANDVTPALS
Ci.kos.578-CKO_04394-YP_001455885.1--36H-COG1/1-561        ---LSPGPMDP--EIAAAVLANWQALLD--KGVAPQMQLAQQGTLTEFAEQANNVTPALS
Sa.bon.1474-SBG_2750-YP_004731563.1--36H-COG1/1-547        ---LSPGPMNS--DISMAVLSNWQALLD--KGVMPQMQLAQQGSLTAWSEHASTVTPALS
Ra.aqu.1678-Q7S_16025-YP_005402985.1--36H-COG1/1-554       ---APHPGLED--SSVQELITSWENLIT--QGVTPLFDAARSKNTESYNQLANSTVPALS
Rahnel.1320-Rahaq_3177-YP_004213898.1--36H-COG1/1-554      ---APHPGLED--SSVQELITSWENLIT--QGVTPLFDAARSKNTESYNQLANSTVPALS
Pectob.2320-W5S_3114-YP_006284066.1--36H-COG1/1-555        ---IDHAGLDN--ALVQAVSRDWNSLVV--QGVEPLYQKAVANALDDYQNQAKNIVPPLS
Ye.ent.378-YE2971-YP_001007160.1--36H-COG1/1-552           ---IDHAQIDP--TLVNGVIDGWSGLID--QGVTPLYQAAMANNSAAYQDLAKKTVPALS
Er.bil.197-EbC_20190-YP_003741400.1--36H-COG1/1-556        ---QSPGPMDA--GYSAAVIGSWQKLLD--NGVLPQMQLAQQGNMDAFRRQATGVTPALS
Ph.lum.1262-plu1854-NP_929127.1--36H-COG1/1-544            --LPKLNDEDGSSVLLASVETSYKEYIN---ALRELSAFLESGNYDAFLDQPT---EKYQ
Er.bil.197-EbC_25340-YP_003741912.1--36H-COG1/1-528        --IPETTPAGG--ALKKSMNEAYDVYAH---ELSQLIAYLQNYQLQDFLDSPT---QGKQ
Di.zea.1140-Dd1591_2937-YP_003005238.1--36H-COG1/1-564     ---IDHVGMSP--ELVEAVTRDWSNLIT--QGVEPMFQRAVDKKFDEYDKYAKDVVPVFS
Er.tas.1011-ETA_14670-YP_001907406.1--36H-COG1/1-518       --IPAVTPEGE--VLKQQIATAYGIYYN---ELAQLAEYLRNDQLQAFLDSPT---QGKQ
Sa.ent.404-STY4234-NP_458344.1--36H-COG1/1-547             --SEAIAGKDP--ALDAQAEASFKQMYD---VLQQSIHYLKADNYAAYGNLDA---QKAQ
Sa.ent.407-STM3577-NP_462478.1--36H-COG1/1-547             --SEAIAGKDP--ALDAQAEASFKQMYD---VLQQSIHYLKADNYAAYGNLDA---QKAQ
Sa.bon.1474-SBG_3170-YP_004731982.1--36H-COG1/1-547        --SDAIAGKDP--ALDARAEASFKQMYD---VLQQSIHYLKADNYEAYGNLDA---QKAQ
En.asb.1498-Entas_2591-YP_004829105.1--36H-COG1/1-555      --IAPQPAM-E--QVSSNIDEKYTAYFA---GLTELIQFLESGNMDAYFAQPT---QGMQ
En.638.865-Ent638_1858-YP_001176586.1--36H-COG1/1-552      --MPPVNGQSQ--ELTAEMQKAYTQMHE---VMRLSIEYLSADNYQAYGDLDA---QEAQ
Ci.rod.62-ROD_19331-YP_003365490.1--36H-COG1/1-553         --IAPHPDM-A--EASKDVDEKYRTYYT---ALAELVQFLENGNMDAYFAQPT---QSMQ
Di.dad.235-Dda3937_01559-YP_003882087.1--36H-COG1/1-566    ---IDHAGMSP--ELVDAVIRDWGNLIT--QGVEPMFQRAADKKFDDYDKFAKDIVPAFS
Er.bil.197-EbC_37780-YP_003743156.1--36H-COG1/1-553        --APQQSGQNA--QLATTVANNYQQMYD---TMKMSIQYLGANNYEAYGNLDA---QKAQ
Pantoe.297-Pat9b_2001-YP_004115872.1--36H-COG1/1-558       ---MAPGPMDA--DTANNVITNWQKLLD--EGIAPQVSLAQQGTLEAYRAQANNVTPALS
```

```
En.clo.1544-EcWSU1_01962-YP_004951821.1--36H-COG1/1-553  --MPQVNGQNA--ALTDEMQKSWSQMHE---VMRLSIAYLRADNYQAYGDLDA---QQAQ
En.asb.1498-Entas_1879-YP_004828403.1--36H-COG1/1-554    --MPQVSGQNA--ALTEEMQKAWSQMHE---VMRLSIEYLRDDNYQAYGDLDA---QQAQ
En.bac.2261-D782_1748-YP_007339929.1--36H-COG1/1-547     --AAVEPEM-A--QVSQRVAQQTQAYFA---ALQELIGFLEQGNMDAYFAQPT---QGKQ
Es.col.1836-Y75_p1862-YP_490148.1--36H-COG1/1-553        --MAPLPEM-V--ATSRNIDEKYKNYYT---ALTELIDYLDYGNTGAYFAQPT---QGMQ
Cr.tur.6-CTU_33010-YP_003211664.1--36H-COG1/1-555        --SPRLNGQDE--ATASQIAEKYQALHD---ILQASIGYLGANNYEAYGNLDA---QKAQ
Cr.tur.6-CTU_25780-YP_003210941.1--36H-COG1/1-536        --VKVDVPEIN--AVAGDVEQSYRDYYQ---ALSELVQYLETSNMDAYFAQAT---QGKQ
En.clo.1544-EcWSU1_00403-YP_004950264.1--36H-COG1/1-549  --LPNTPGLDE--NLANRLEEQYRIYSA---TLTQMNVLLGQGNLEDMFKQNA---EQKQ
En.638.865-Ent638_2457-YP_001177177.1--36H-COG1/1-555    --ITPQPAM-E--QASLNIDEKYKNYAA---GLAELIQFLESGNTDAYFAQPT---QGMQ
Sa.bon.1474-SBG_1755-YP_004730609.1--36H-COG1/1-553      --MAPLPAM-V--EASANVDEKYQRYHA---ALAELIQFLDNGNMDAYFAQPT---QGMQ
Pr.mir.1265-PMI1665-YP_002151396.1--36H-COG1/1-548       -NQEVIPQEEK--EIFNTVKNDYHVLYS---ALVELHGMLKKGDFQGFLDQPT---ERYQ
Cr.sak.579-ESA_01348-YP_001437444.1--36H-COG1/1-556      --VTVDVPEIN--AAAGDVEQSYRDYYQ---ALSELVQYLETGNMDAYFAQAT---QGKQ
Cr.sak.579-ESA_00560-YP_001436686.1--36H-COG1/1-555      --SPRLNGQDE--ATASKITEKYQALHD---TLQASIGYLSANNYEAYGNLDA---QKAQ
Sa.ent.407-STM1919-NP_460876.1--36H-COG1/1-553           --MTPLPAM-A--EASANVDEKYQRYQA---ALAELIQFLDNGNMDAYFAQPT---QGMQ
Ci.kos.578-CKO_01066-YP_001452645.1--36H-COG1/1-552      --INPLPEM-A--EASGNVDEKYQQYHA---ALAELVQFLENGNMDAYFAQPT---QGMQ
En.asb.1498-Entas_0424-YP_004826963.1--36H-COG1/1-549    --LPNTPGLGE--DLPKQLEEQYRIYSA---TLTQMNVLLGQGNLEDMFKQNA---EQKQ
Pantoe.297-Pat9b_1273-YP_004115150.1--36H-COG1/1-553     --LPRIKGQSD--AAAALVEAKFSAMAD---LLQQSAAFLKANDYPSYGNLDA---QQAQ
Ed.tar.1771-ETAF_2264-YP_005699859.1--36H-COG1/1-555     --LSLSSGHDN--GRAEKVRGLYGKARA---DLVSMIECLQSNDIAGFQSVLK---NHSS
Pa.ana.1905-PAJ_3534-YP_005936409.1--36H-COG1/1-553      --APALPGQHA--EVTKNVEAAYNKMHE---TMLASVDFLKNNNYQGYGNLDA---QEAQ
Pa.vag.184-Pvag_1203-YP_003930842.1--36H-COG1/1-552      --MPRKAGQSE--AQSTQVQTAYQHMRD---TMLASVTFLKQGNYPAYGNLEA---QAAQ
Pantoe.297-Pat9b_1605-YP_004115478.1--36H-COG1/1-556     --LPATPGFPV--ELRDRLEADYAAYNN---GLKAMLARLKADDLQGMFAQNI---EAKQ
Cr.sak.579-ESA_00210-YP_001436348.1--36H-COG1/1-557      --LPETPGMDE--RLVDRLEEQYRVYSS---TLAQMNKFLAEGNLEGMFKQNA---EQKQ
Er.tas.1011-ETA_pET460340-YP_001905943.1--36H-COG1/1-543 ---HTPGPLDR--QYATAATERWQKLLE--EGVIPQVRLAQQPALDDYRLHARTVTPALS
Cr.tur.6-CTU_36610-YP_003212024.1--36H-COG1/1-557        --LPETPGMDE--RLVDRLEEQYRIYSS---TLAKMNKFLAEGNLEGMFKQNA---EQKQ
Pe.car.1139-PC1_1159-YP_003016743.1--36H-COG1/1-556      --LPNTPGLDD--ALSKKLEASYTAYDK---LLNDIVDSLSGGLAGAAMKLSSG-ATPLN
Er.tas.1011-ETA_14640-YP_001907403.1--36H-COG1/1-554     --TPDTPGIDP--KLSEELEQNYEQFSS---ALQEMTQQMENKQLDAMFAQNI---EQKQ
Pa.vag.184-Pvag_1725-YP_003931362.1--36H-COG1/1-556      --IPDTPGQDA--TLNDQLDQRFSAYDG---ALNQLQTALEANDPIAAAKAPV---APSQ
Ed.ict.1187-NT01EI_3742-YP_002935104.1--36H-COG1/1-525   --APRLPGQDA--ALAAEVEHRFTALYR---LLRDSSHYLRAGDYPAYGKLET---QEAQ
En.638.865-Ent638_0380-YP_001175119.1--36H-COG1/1-549    --LPETPGMDE--NLAKQLEEQYRIYSS---TLAQMNSLLAQGNLDDMFKQNA---EKKQ
Er.bil.197-EbC_25360-YP_003741914.1--36H-COG1/1-556      --IPNTKGVDP--KVVDDLEQNYVSYSN---ALSTMLDLMQSKQLPEMFKLNI---EQKQ
Er.tas.1011-ETA_16910-YP_001907630.1--36H-COG1/1-543     ---HTPGPLDR--QYATAATERWQKLLE--EGVIPQVRLAQQPALDDYRLHARTVTPALS
Es.fer.1173-EFER_3055-YP_002384154.1--36H-COG1/1-603     --LPLDPRQSE--TAAIEIKRNYDIYHN---ALAELIQLLGAGKINEFFDQPT---QSYQ
Xe.nem.162-XNC1_1619-YP_003711880.1--36H-COG1/1-520      --LPQIGDNQKRDTLLVEVEQAHREYFH---ALKELKDFLYGGDFQAFLDQPT---EDHQ
En.clo.1544-EcWSU1_02811-YP_004952664.1--36H-COG1/1-559  --ITPQPAM-A--QVSQNIDEKYNAYYA---GLTELIQFLESGNMDAYFAQPT---QGMQ
Di.dad.235-Dda3937_03500-YP_003882750.1--36H-COG1/1-553  ---SEHIGVDK--DVVQQMTDVWGRLLQ--SGVEPMLTAVKDGRMDDFRQLFRKQYPPLS
Xe.bov.105-XBJ1_1924-YP_003467828.1--36H-COG1/1-523      --LPQIEKNDQSVALLAAVERSYQEYIN---ALKELKIYLYEGNFQAFLDQPT---ENYQ
Rahnel.1320-Rahaq_1831-YP_004212576.1--36H-COG1/1-556    --LPRRTEKAV--ALTAVNKASYQQLHD---MLQTLTELLSSGDMNGFLNGPA---QKYQ
Ra.aqu.1678-Q7S_08965-YP_005401598.1--36H-COG1/1-556     --LPRRTEKAV--ALTAVNKASYQQLHD---MLQTLTEMLSSGDMNGFLNGPA---QKYQ
Cr.sak.579-ESA_01126-YP_001437230.1--36H-COG1/1-514      ---LSPGPLDP--AVSQQVIADWQKLLD--DGVIPQMKLAREGTPDAYRAHAGKVTPVFS
Pe.atr.485-ECA2712-YP_050803.1--36H-COG1/1-560           ---AGHVGVDK--EAADNMIASWTQLLD--NGLTPMLNAARDNRQEEFRQLFRKAYPPLS
En.clo.1544-EcWSU1_00560-YP_004950421.1--36H-COG1/1-597  --LPRDPRQND--ADATEIKRNYDIYHG---ALAELIQLLGAGKINAFFDQPT---QSYQ
Di.dad.235-Dda3937_03498-YP_003882752.1--36H-COG1/1-561  ---ADQVGVDK--ALVDNMITTWSALIS--NGIDPMYRALQSNNLDEFRRVFRTVYPPAS
Cr.tur.6-CTU_27880-YP_003211151.1--36H-COG1/1-514        ---LSPGPLDP--AVSQKVIADWQKLLD--EGVIPQMKLAREGTPDAYRAHAGKVTPVFS
Pe.car.1139-PC1_1669-YP_003017246.1--36H-COG1/1-560      ---VEHVGVSK--ETADNMIAAWTQLLD--NGLTPMLNAARENRQEEFRQLFRKTYPPLS
Ra.aqu.1678-Q7S_14450-YP_005402672.1--36H-COG1/1-551     ---IDHGNLDS--NTVDDIYNSSFTLFN--SAVFPMYESAAVKNTSLFASLKNDKYLPLR
Ci.kos.578-CKO_03622-YP_001455137.1--36H-COG1/1-517      --LPETPGLDE--HLADRLEEQYGIYSA---TLAKMNTFLAAGQLEEMFKQNA---EQKQ
Di.zea.1140-Dd1591_2408-YP_003004728.1--36H-COG1/1-557   ---SPQTGISD--GLVRQLVTSWEQILG--NAVEPMMTALKEGKMDEFKRIFLKQYPPMS
Di.zea.1140-Dd1591_2406-YP_003004726.1--36H-COG1/1-559   ---APHIGVEQ--KITDEMVSSWSALIA--TAIEPMYTALQKNDLDAFRNVFRKTYPPAS
Ed.ict.1187-NT01EI_2801-YP_002934203.1--36H-COG1/1-554   --LSLSSGHDN--GRAEKVRGLYGKARA---DLVTMIEFLQNNDVASFQSVLK---NRSS
Pe.car.1139-PC1_1384-YP_003016966.1--36H-COG1/1-559      ---AEHVGVEP--ATVDAVSKTWSTLIA--TAIDPMNAALQRNDYEGFRQIFRSVYPSIS
Rahnel.1320-Rahaq_2866-YP_004213595.1--36H-COG1/1-551    ---IDHGNLDS--NTVDDIYNSSFTLFN--SAVFPMYESAAVKNTSLFASLKNDKYLPLR
Pe.atr.485-ECA1281-YP_049387.1--36H-COG1/1-556           --LPNTPGLDD--ALSKKLETSYTAYDK---LLNDIVDSLSGGLAGAAMKLSSE-ATPFN
Pectob.2320-W5S_1886-YP_006282848.1--36H-COG1/1-549      ---VGHDGVDK--ETANNMIAAWTQLLD--NGLTPMLNAARDNRQEEFRQLFRTVYPPLS
Pectob.2320-W5S_1696-YP_006282659.1--36H-COG1/1-559      ---AEHVGVEH--ATVDAVSKTWSALIA--TAIEPMNAALQRNDYEGFRQIFRSTYPPIS
Di.zea.1140-Dd1591_2407-YP_003004727.1--36H-COG1/1-555   ---SEHIGVDK--EIVRQMIDVWGRLLQ--SGVEPMLTSIKNGRMDDFRQQFRNQYPPLS
Pe.atr.485-ECA1509-YP_049611.1--36H-COG1/1-559           ---SEHVGVNQ--ATVDAVKNTWNTLIS--SSIEPMNSALQRNDPEAFRQIFRSVYPPVS
Pa.vag.184-Pvag_1724-YP_003931361.1--36H-COG1/1-520      --LPATPGLEV--ALRDKLEADYAQYEK---GLKGMVTSLQARDLEGMFKQNI---EQKQ
Pa.ana.1905-PAJ_1541-YP_005934417.1--36H-COG1/1-520      --IPETPGADT--EMRDRLQNDFKAYLT---GLRGMIASLKAKDLEGMFKQNI---EQKQ
Mo.mor.2189-MU9_1596-YP_007505015.1--36H-COG1/1-519      ---ISPGPMDV--QMSEKVIARWQALYD--EGVKIQMQLAEQGDQAGYRKQSHDVTPQLS
Pr.mir.1265-PMI2808-YP_002152509.1--36H-COG1/1-563       --LSAQTGFDQ--QRMAEIKQLYQKSRY---DLDLLVQYLNERNTNAIRLILK---SPAN
Di.zea.1140-Dd1591_0395-YP_003002764.1--36H-COG1/1-556   ---TDHANIST--KSIDDIYNSSYQLFS--EAIVPMLDAARQNNHDAYAKLMSEKYNPLR
En.clo.1544-EcWSU1_03786-YP_004953635.1--36H-COG1/1-515  ---ISPGPMDE--QVSAQVIGSWQALLE--QGVTPQMQQAKQSSLEGYRQQANNITPPLS
Pectob.2320-W5S_3170-YP_006284116.1--36H-COG1/1-556      --LPNTPGLDE--SLSKKLEESYTAYDK---LLNDIVDSLSGGLAGAAMKLSSG-ATPFN
Di.dad.235-Dda3937_02184-YP_003884754.1--36H-COG1/1-556  ---TDHANISS--TTIDDIYNSSYQLFS--EAIVPMLDAVRQNNHDAYNKLVREKYNPLR
Ci.rod.62-ROD_32641-YP_003366748.1--36H-COG1/1-517       --LPETPGLEE--HLADRLEEQYGIYSA---TLAKMNVLLAEGKLEEMFQQNA---EQKQ
Di.dad.235-Dda3937_03499-YP_003882751.1--36H-COG1/1-561  ---SEHVGVDE--ALAKQMVDVWGRLLQ--NGMAPMLAAVNEGRNDDFQQLYRQQYMPLS
Di.dad.235-Dda3937_00105-YP_003883346.1--36H-COG1/1-561  --IPPLPVQDA--ALAKNINDNYQALRS---VLQRIVELIKTQDVKTIQALPT---QKMQ
Pantoe.297-Pat9b_1604-YP_004115477.1--36H-COG1/1-555     --TPDTPGQDV--QLNKHLDETYAAYAS---ALNQMQAALQANEIEKAGQLPV---APSQ
Er.tas.1011-ETA_14660-YP_001907405.1--36H-COG1/1-560     --ELPAGVDNQ--PHVQAVNDHFNILHN---ALSELMRMLTSGDFKGFVDQPT---TGFQ
Pr.mir.1265-PMI2809-YP_002152510.1--36H-COG1/1-575       --SAENADFDR--EKVAKMKKLFQRVIY---DLDQLVSCLEIRDYYGFQSLYA---HNTN
Ed.tar.1771-ETAF_3053-YP_005700648.1--36H-COG1/1-516     --APRLPGQDA--ALAAEVERRFTALYS---LLRDSSRYLRAGDYPAYGNLET---QAAQ
Se.pro.864-Spro_1415-YP_001477647.1--36H-COG1/1-546      --LSQSEQSEN--ERTKNVKNLYEKARG---DLLQLIISLEDGDITRFQSIMH---NSSS
Di.zea.1140-Dd1591_1804-YP_003004134.1--36H-COG1/1-562   --IPLLSVQDA--ALAKDINDNYLALRR---ILQQIVELIKTQDVKTIQALPT---QKAQ
Di.dad.235-Dda3937_03501-YP_003882749.1--36H-COG1/1-575  ---SRQVGVSD--EQVQQLIASWEQVLG--NAVEPMMSALKDGRIDEFKRVFLKQYPPMS
Pantoe.297-Pat9b_0851-YP_004114731.1--36H-COG2/1-524     ---IQITAVGK--KKIAGLLDASHLFEQ---QKDQLVALVKSGDLEASSTFVLKKITDTQ
Pa.vag.184-Pvag_0291-YP_003929953.1--36H-COG2/1-525      ---IQVTPVGK--KKIAGLLASSQAFEQ---VKTELIGLIKAGNFDAASAYVLKSMTATQ
En.clo.1544-EcWSU1_03886-YP_004953735.1--36H-COG2/1-519  ---ALQDPESQ--RVLAEIRGVRQQYLD---SRYRILQAVQNNDRAGAIQEMMTNTLKLQ
Cr.tur.6-CTU_04790-YP_003208842.1--36H-COG2/1-515        ---TLTDDASR--QALAQIADVRKQYLA---SRFRILDAVKRNDRNAALEEMMSTTVTLQ
Ci.kos.578-CKO_04484-YP_001455975.1--36H-COG2/1-521      ---SRHDEASK--AIIAGIRDVRQQYLA---SRFRILQEIQNYNRPAAIQEMMTTTVNIQ
Cr.sak.579-ESA_03488-YP_001439539.1--36H-COG2/1-515      ---TLTDDASR--QALAQIAEVRKQYLA---SRFRILDAVKRNDRNAALEEMMSTTVALQ
Ci.rod.62-ROD_48071-YP_003368199.1--36H-COG2/1-521       ---LPLNDDSR--KLLAGIREVREQYLA---SRTRILDALRNHDRQAAMQEMFSTTMKIQ
Sa.bon.1474-SBG_2851-YP_004731664.1--36H-COG2/1-521      ---HRHDAASQ--KIITEIREARQQYLE---SRFRILRDIQSHNRQAAIQEMMTKTVQVQ
Sa.ent.407-STM3216-NP_462130.1--36H-COG2/1-521           ---NRHDAASQ--KIITEIREARQQYLE---SRFRILQDIQSHNRQAAIQEMMTRTVQVQ
En.asb.1498-Entas_3753-YP_004830248.1--36H-COG2/1-519    ---ELHDRKSQ--QVLADIRGVRQQYLD---SRYRILQAVQNNDRAGAIQEMMTNTLNLQ
Mo.mor.2189-MU9_3389-YP_007506806.1--36H-COG2/1-521      ---TLKDEASQ--NILKEIRSVRQQYLT---SQRRMVQFSQNGDEASAINEMVNTTAGIQ
Sa.ent.404-STY3394-NP_457606.1--36H-COG2/1-522           --ENSHDADSQ--KIINEIREARQQYLE---SRFRILKDIQSNNRQAAIQEMMTRTVQVQ
En.638.865-Ent638_3526-YP_001178235.1--36H-COG2/1-519    ---SLQDKKSQ--QILAGIAAVRQEYLD---SRYRILQAVQNNDRAGAIQEMMTKTVDLQ
Pe.atr.485-ECA1774-YP_049874.1--36H-COG2/1-554           ---SVNDDRSV--AVLRDISDIRRDFLG---SANKLSSLISAGNTDAAAEEYFNVTRVTQ
En.638.865-Ent638_2119-YP_001176845.1--36H-COG2/1-549    ---LLRFEKGK--AFFRAIQETRGKYLV---LRKEGLEMAEQGKYENMSEFISTRFMPVT
```

```
Pectob.2320-W5S_2813-YP_006283770.1--36H-COG2/1-554      ---SVNDDKSV--TVLRDIGDIRRDFLG---SANKLSSLVSAGNTDAAAEEYFNVTRVTQ
Di.zea.1140-Dd1591_0695-YP_003003055.1--36H-COG2/1-561   --DESQDAKSQ--ALISDIHRIRVQFLA---SGQKIGELVKNHDREAALEEFNQHLDPTQ
Pa.vag.184-Pvag_pPag30079-YP_003729820.1--36H-COG2/1-546 ---MLKAPKSR--ELIASIRSTGTQYLA---FSDDVVALGMAGKRDEAAQLLLGPRYQTQ
Er.bil.197-EbC_38940-YP_003743272.1--36H-COG2/1-526      ---LQTTPVGI--KKIQAVKEAAQTFET---AKSTLLEMIRAGKQDEAAEFALQKMTPAQ
Ra.aqu.1678-Q7S_24891-YP_005419232.1--36H-COG2/1-554     ---IQSTVTGK--QKIKTLQEAGETFET---AKEQLIALARAGDMDGATEFVLRKLTTSQ
En.asb.1498-Entas_2287-YP_004828805.1--36H-COG2/1-513    ---LSWTAKEA--PIVDAIAEHLAQFGAGVQDMQTAMAAVKANKGDATAEQAL---KTAE
Rahnel.1320-Rahaq_4869-YP_004215574.1--36H-COG2/1-554    ---IQSTVTGK--QKIKTLQEAGETFET---AKEQLIALARAGDMDGATEFVLRKLTTSQ
Di.dad.235-Dda3937_00027-YP_003883574.1--36H-COG2/1-554  ---SAKDARSI--SLLNDIQQIRSEYSV---SGNKLMDLASRGNKQEAVAELINVSLSLQ
Pr.stu.1965-S70_05790-YP_006215722.1--36H-COG2/1-520     ---NAKDPQSI--AVLAEIYDVRERFLQ---SQARLSEFLNNNNRSAAIDEMMNKTSAIQ
En.638.865-Ent638_2100-YP_001176826.1--36H-COG2/1-512    ---LEWTPQEG--VIINAIAENLARFESSVAEMQTAMAALNANKDDTTAQQAL---KVAE
Pe.car.1139-PC1_2526-YP_003018093.1--36H-COG2/1-554      ---SVSDDKSV--AVLRDIGDIRRDFLG---SANKLSSLISAGNTEAASEEYFNVTRVTQ
Mo.mor.2189-MU9_702-YP_007504121.1--36H-COG2/1-525       ---FGMDPQGR--ALLDKMIADEKHYAA---VTREVLELDQQGRHEEAIAKMMDAEYAAS
Pe.car.1139-PC1_3443-YP_003018995.1--36H-COG2/1-511      ---GNWPAEDK--SAIQDISASLNAYLT---QRQQVMSPTVS---QQTLSELNNQMAQLQ
Pa.ana.1905-PAJ_0915-YP_005933791.1--36H-COG2/1-559      ---QVNTPQGS--EIFTRLKARAELFLP---FIDEVVALAQQGKSDDATQLLFGPRYQTQ
Pectob.2320-W5S_3736-YP_006284671.1--36H-COG2/1-510      ---GYWPAEDK--SAIQDISASLNAYLT---QRQQVMSPDVS---QQRLSELNDQMALLQ
Pe.atr.485-ECA0183-YP_048310.1--36H-COG2/1-553           ---QTVEERSV--TQLRAIRAIRTEFLK---SGDKIISEVVAGNREAAIEEFNNNLNVVQ
Rahnel.1320-Rahaq_3107-YP_004213828.1--36H-COG2/1-503    ---YSWDPQDQ--RLVETMPENIRRYRE---SWQHTLNPPAGSDAEKITAQLAEAGLALT
Pe.car.1139-PC1_4071-YP_003019622.1--36H-COG2/1-553      ---QTVEERSV--SQLRDIRAIRNEFLK---SGDKIISEVVAGNRDAAIEEFNNNLNVTQ
Di.dad.235-Dda3937_03462-YP_003884507.1--36H-COG2/1-563  --NESNDARNQ--QLIGDIRLIRAKFLE---SGQKVIALVKANNREEALNEFNQRLNPAQ
Ra.aqu.1678-Q7S_15665-YP_005402913.1--36H-COG2/1-503     ---YSWDPQDQ--RLVETMPENIRRYRE---SWQHTLNPPAGSDAEKITAQLAEAGLALT
Pectob.2320-W5S_4505-YP_006285423.1--36H-COG2/1-555      ---QTVEERSV--TQLRAIRAIRTEFLK---SGDKIISDVVAGNREAAIEEFNNNLNVVQ
Pe.atr.485-ECA3642-YP_051730.1--36H-COG2/1-551           ---LISTPEEK--ALFDKVGEYRQSYIK---KRDAIITEKGAGNFDRARTLFDNEFVPAS
Pe.car.1139-PC1_1417-YP_003016999.1--36H-COG2/1-554      ---SITSQKGR--ELFNQLIDIRKQYSV---SINKAIDYAMDEQDALATELVFGDIAAIQ
En.asb.1498-Entas_4509-YP_004821569.1--36H-COG2/1-559    --IGGEGGRAE--KELDEVMESSDKLVN---ASNHVFEALNAGHVDEAKRQLNSEAVPLS
Pa.ana.1905-PAJ_3064-YP_005935940.1--36H-COG2/1-544      ---EVASVTGR--QLFSNIQSHAALFLP---LMDKVVALAQQGKKEEADQLLQASGDNIQ
Pectob.2320-W5S_1733-YP_006282696.1--36H-COG2/1-535      ---RINAGKGR--ELFNQLIDIRKQYSL---SINKAVDYAMEGEDALATELVFGDIATIQ
Pe.atr.485-ECA4334-YP_052421.1--36H-COG2/1-556           ---STAEKHAQ--ELVAELQRVRPAYSS---SMANAITLAMANKNSEAQHLLLTDVRAKQ
Di.zea.1140-Dd1591_1602-YP_003003935.1--36H-COG2/1-554   ---SVKDEHSA--SLLHEIQQIRSEYSV---SGNKIMDLASRGNKQEAIAELMSVSQDLQ
Pectob.2320-W5S_4506-YP_006285424.1--36H-COG2/1-540      ---DASGERSR--KQVGVVRGFRAEFIK---SGDQIISDALAGNNDVAIAEFNNTLNVIQ
Di.zea.1140-Dd1591_1453-YP_003003794.1--36H-COG2/1-532   ---TYLTPEET--RLADQLAENLQRYNR--EGVQPTDAAIRAGQTDMALRLYDDKVSALE
Pectob.2320-W5S_0106-YP_006281117.1--36H-COG2/1-556      ---STVDKHAK--GLVTILEQVRPAYSV---AMKEAITLAMTGKNNEARDLLLTDVKAKQ
Di.zea.1140-Dd1591_0769-YP_003003127.1--36H-COG2/1-505   ---NAWPEEDE--SGIRQVRTMLEAYTS---QRKQVLKPDAS---QQVLSDLNAQMATLQ
Ra.aqu.1678-Q7S_18035-YP_005403387.1--36H-COG2/1-535     KEHSDVTDKDR--QFVTAISNAEEGYRP---VAKHIVDLALEEKKAEAIDRMNRELRPQL
Rahnel.1320-Rahaq_3579-YP_004214298.1--36H-COG2/1-535    KEHSDVTDKDR--QFVTAISNAEEGYRP---VAKHIVDLALEEKKAEAIDRMNRELRPQL
Pe.car.1139-PC1_4072-YP_003019623.1--36H-COG2/1-540      ---NTSGELAR--KQVGVVRGFRAEFIK---SGDKIISDALAGNNDVAVAEFNNTLNVIQ
Pe.car.1139-PC1_3464-YP_003019016.1--36H-COG2/1-551      ---LISTPEEK--ALFDKVGEYRQSYIK---KRDAIIAEKGAGNFDRARSLFDNEFVPAS
Pe.car.1139-PC1_0111-YP_003015709.1--36H-COG2/1-556      ---SATEKHAQ--ELVAELERVRPAYSS---SYTNAITLAMTNKNSEAQHLLLTDVRAKQ
Pe.atr.485-ECA0182-YP_048309.1--36H-COG2/1-542           ---NASGERSR--KQIETIRVLRAEFIK---SGDKIISDALAGNNDVAVEEFNSNLNAIQ
Pectob.2320-W5S_3753-YP_006284688.1--36H-COG2/1-551      ---LISTPEEK--ALFDKVGEYRQSYIK---KRDAIITEKGAGNFDRARTLFDNEFVPAS
Pa.ana.1905-PAJ_2666-YP_005935542.1--36H-COG2/1-514      ---EDSTPQAR--EAFDKIVSSEGPVLA---VLDHAGQLGLQNKQQEAISYLIKTVRPLQ
Ci.rod.62-ROD_16161-YP_003365194.1--36H-COG6/1-557       --RAVKTPADE--ALDAELQQRYKAYLA---GLQPMLKYAQNGMFEAIINHESEQARLLD
En.asb.1498-Entas_2095-YP_004828614.1--36H-COG6/1-563    --RAVRTPADE--ALDADLKARYDAYIA---GLQPMLKYAKNGMFEAIINHENETARPLD
En.638.865-Ent638_1961-YP_001176688.1--36H-COG6/1-563    --RSVRTPADE--ALDNDLKTRFDAYIT---GLQPMLKYAKNGMFEAIINHENESARPLD
Cr.sak.579-ESA_01710-YP_001437800.1--36H-COG6/1-565      --RASHEDAEA--ALDSELKARYDAYIS---GLAPMLKYAKNGMFEAIIEHENETARKLD
Cr.tur.6-CTU_22440-YP_003210607.1--36H-COG6/1-565        --RESHAESEA--ALDSELKARYDAYIS---GLTPMLKYAKNGMFEAIIEHENETARKLD
Ci.kos.578-CKO_01456-YP_001453025.1--36H-COG6/1-562      --RAVKTPADE--ALDAELNTRFKAYID---GLQPMLKYARNGMFEAIINHENEQARPLD
Rahnel.1320-Rahaq_1930-YP_004212674.1--36H-COG6/1-566    --RPNKTDKDR--ALDEPLKKAYDAYLT--QGMAPMLESAKQAHFEEVISQEAETVRGLD
Pantoe.297-Pat9b_2403-YP_004116261.1--36H-COG6/1-564     --RKTKTAEDL--ALDAPLTERFNAYID--KGLKPMIDSAKQGSFEGIIAQETDVTRKLD
Er.bil.197-EbC_26700-YP_003742048.1--36H-COG6/1-567      --RRVKTPEDM--ALDGELKTKFDAYVT--QGLLPMVKSAKDGSFEGVIAQETDYTRKFD
Pectob.2320-W5S_1760-YP_006282723.1--36H-COG6/1-570      --RPVKTPQDV--ALDEDLRKSYDAYVN--QGLMLMLTAAKQGLFEEVITLESEETRILD
Pa.ana.1905-PAJ_1621-YP_005934497.1--36H-COG6/1-568      --RKSKTPEGI--KLDEQLQARFKEYVT--KGLMPMIESGKQGSFESIIAQETDTTRKLD
En.clo.1544-EcWSU1_02120-YP_004951978.1--36H-COG6/1-576  --RPVRTAEDE--ALDAELKTRFDAYIA---GLQPMVKFAKNGMFEAIINHENESARPLD
Ra.aqu.1678-Q7S_09815-YP_005401764.1--36H-COG6/1-566     --RPNKTDKDR--ALDEPLKKAYDAYLT--QGMAPMLESAKQAHFEEVISQEAETVRGLD
Ye.pse.585-YPTB2412-YP_070925.1--36H-COG6/1-579          --RPTKSETDM--ALDVPLKKAYEQYRD--DGMKPMLAATKEGHFEEVISLDAEKISLLD
Ye.ent.378-YE2588-YP_001006789.1--36H-COG6/1-579         --RPIKSETDT--ALDGPLKKAYEQYRD--DGMKLMLEATKEGHFEEVISLEAEKLNQLD
Di.dad.235-Dda3937_02787-YP_003883649.1--36H-COG6/1-572  --RAVKTDTDL--ALEPELNKAYNDYVE--KGIMPMLKAAKDGYFEEILTHESEEVRVLD
Pa.vag.184-Pvag_1800-YP_003931435.1--36H-COG6/1-568      --REVKTPADM--ELDTTLQARFNDYIN--KGLIPMINAGKQGSFEGIIAQETDVTRKLD
En.bac.2261-D782_2307-YP_007340470.1--36H-COG6/1-558     --RRVKTAADS--ALDKELTARYTAYID---GLKPMLKYAKNGMFEAIINHENEQAKPLD
Pe.atr.485-ECA1683-YP_049784.1--36H-COG6/1-573           --RPVKTPQDM--ALDGDLRKSYDAYVN--QGLMLMLTAAKQGLFEEVITLESEETRILD
Pe.car.1139-PC1_2617-YP_003018183.1--36H-COG6/1-573      --RPVKTPQDM--ALDDELRKSYDAYVN--QGLMLMLTAAKQGLFEEVITLESEETRVLD
Di.zea.1140-Dd1591_1535-YP_003003868.1--36H-COG6/1-574   --RSVKTDMDL--ALEPELNKAYNEYVE--KGIMPMLKAAKDGYFEEILTHEAEEVRVLD
Di.zea.1140-Dd1591_3580-YP_003005868.1--36H-COG6/1-572   --REVKTPADL--ALDDALQQRYNDYVS--NGVRPMLEAVKAGRYDDVVAIEWKQTRKLD
Es.col.1836-Y75_p1397-YP_489687.1--36H-COG6/1-546        --RPVKTPADE--ALDTELNQRFQAYIT---GMQPMLKYAKNGMFEAIINHESEQIRPLD
Pe.atr.485-ECA3902-YP_051990.1--36H-COG6/1-575           --RPDRLESEK--ALDEDILKAYTNYRD--NAIVLMQKATSDGEFEDLVSLESTLARTLD
Sa.bon.1474-SBG_1454-YP_004730321.1--36H-COG6/1-537      --RAVKTPEDE--ALDNELNARYTAYIN---GLQPMLKFAKNGMFEAIINHENEHAKQLD
Sa.ent.407-STM1626-NP_460585.1--36H-COG6/1-541           --RAVKTPADD--ALDNELNARYTAYIN---GLQPMLKFAKNGMFEAIINHENEQAKQLD
Pe.car.1139-PC1_3679-YP_003019230.1--36H-COG6/1-567      --RPDKLESEK--ALDEDILKAYNNYRD--NAIVVMQKATSDGEFEDLVSLESTVARQLD
Pectob.2320-W5S_4020-YP_006284955.1--36H-COG6/1-575      --RPDRLESEK--ALDEDILKAYNNYRD--NAIVVMQKATTEGEFEDLVSLESTLARTLD
Di.zea.1140-Dd1591_0523-YP_003002884.1--36H-COG6/1-568   --RTDKSSEEK--SLDSDLTTHYIAYRD--KVMVPMVDFVRNGEFENTIELETTLARQLD
Di.dad.235-Dda3937_02665-YP_003881279.1--36H-COG6/1-572  --REVKTPADL--ALDDVLQQRYNDYVN--NGVRPMLDAVKAGRYDDVVTTEWKQTRKLD
```

```
Pr.mir.1265-PMI1666-YP_002151397.1--36H-COG1/1-568        DGFYKEYLAYVMR-NEKFNNQLNSALEASHQRSIITMVILAIIVVTALIICWFALRNALI
Di.dad.235-Dda3937_02779-YP_003883640.1--36H-COG1/1-566   DKFEKAYYSYKES-YDKVYANAVEENNSAYSTALWLLISVAILVVVMALLVWLGINRSLI
Di.zea.1140-Dd1591_1542-YP_003003875.1--36H-COG1/1-566    DRFEKAYYSYKDS-YDKVYANAVEENNSAYSTALWLLISVAILVVVMALVVWLGINRSLV
Pe.atr.485-ECA1691-YP_049792.1--36H-COG1/1-561            DNFEKAYYVYKAE-NDKLYQAGIAKNDAAYDSALWILGSIITLVFALALFSWFGIQQLLI
Se.pro.864-Spro_2983-YP_001479212.1--36H-COG1/1-556       DAFEDIYNTYLAQ-NDKLYQSAVEDSNHSFNFAIWTLAVVLLTVLAVIVLVWSGIHHILV
Ph.asy.1114-PAU_02685-YP_003041519.1--36H-COG1/1-564      SAFEEEYNFYLTQ-NDHLYSDVVFDAGISYRNAMIMLGIIMLILVSAMIFSWVGIKHSLL
Pr.stu.1965-S70_18135-YP_006218124.1--36H-COG1/1-561      IKFEEQFNAYFAD-ISEDFQSEVKRAEANYRESLYMIIVLSIVLALIGFFSYRYVRKGII
Mo.mor.2189-MU9_1762-YP_007505181.1--36H-COG1/1-561       MKFEEQFNSYFDS-ISAAFRGEVQNAEDNFSESLYMLIALAIILALISLISYRYVQRGII
Pe.car.1139-PC1_2609-YP_003018175.1--36H-COG1/1-561       DNFEKAYYAYKAE-SDKLYQAGIAKNNEAYDSALWILGFIVVLVFAVALVSWIGIQQLLI
Xe.nem.162-XNC1_1620-YP_003711881.1--36H-COG1/1-567       NAFGAEYAFYITQ-NDKLYNEVSDAANIVYRNVMISLISVFVLLIGVMAVSWIGLRKVLI
Pectob.2320-W5S_1768-YP_006282731.1--36H-COG1/1-561       DNFEKAYYTYKAE-SDKLYQAGIAKNDEAYDSALWILGFIIVLVFALALISWIGIQQLLI
Pa.vag.184-Pvag_1723-YP_003931360.1--36H-COG1/1-558       DKFQKDYSAWLEN-NKALSQQGVEANQQAYSRSITIVIATLAVTLLMIILVWNVMRSVLI
Ye.pes.133-YPZ3_2024-YP_003568196.1--36H-COG1/1-557       NEFEQNYNNYLTQ-NDGLYDSAVEDSNQSFSFAMGVVITVLVVVLVMMLLVWLGMQQILI
Ye.pse.585-YPTB2401-YP_070915.1--36H-COG1/1-557           NEFEQNYNNYLTQ-NDGLYDSAVEDSNQSFSFAMGVVITVLVVVLVMMLLVWLGMQQILI
Ye.ent.378-YE2575-YP_001006778.1--36H-COG1/1-557          NAFEHDYNTYLTQ-NDRLYSGAVEDSNRSFTYAMSVIVFVLIAVFVVIVGVWLGMQHILI
Pa.ana.1905-PAJ_1540-YP_005934416.1--36H-COG1/1-563       DKFQKDYDAWLNY-NLQLSQQGVDANNAAYQRMIMLVVGTLLVTLLVIVLVWNLMRSLLI
Ph.lum.1262-plu1853-NP_929126.1--36H-COG1/1-564           SAFEEEYNFYLTQ-NDHLYSDVVFDASISYRNAVIMLGIIMLILVSAMIFSWVSIKHSLL
Serrat.1901-SerAS13_3075-YP_006025914.1--36H-COG1/1-555   NGFEDIYNTYLAQ-NDKLYQSAVEDSNSSFSFAIWTLVVVLVVVLAVIVLVWSGIHHILV
Sa.ent.404-STY2128-NP_456485.1--36H-COG1/1-553            NALGEALGNYARV-SENLYRQTFDQSAHDYRFAQWQLGVLAVVLVLILMVVWFGIRHALL
Ra.aqu.1678-Q7S_08960-YP_005401597.1--36H-COG1/1-564      DNFEKAFVTYIDQ-NDRLYNEAVKVSENSFTHAIWMLGTVLIVLAIFILLAWTGVQRILI
Se.mar.2260-D781_2759-YP_007345195.1--36H-COG1/1-555      DAFEQAYVTYLLK-NGELYHDAVEASEHSYSSAIWTLASVLLVVLAAIVLVWTGIHHILV
Rahnel.1320-Rahaq_1830-YP_004212575.1--36H-COG1/1-564     DNFEKAFVTYIDQ-NDRLYNEAVKVSENSFTHAIWMLGTVLIVLAIFILLAWTGVQRILI
Se.ply.1407-SerAS9_3072-YP_004506452.1--36H-COG1/1-555    NGFEDIYNTYLAQ-NDKLYQSAVEDSNSSFSFAIWTLVVVLVVVLAVIVLVWSGIHHILV
Ed.ict.1187-NT01EI_1461-YP_002932882.2--36H-COG1/1-555    NSFEKSYTTYLAQ-NDRLYKGAMSDNNDAYHSALWTLFGVMVVVIAAIVLVWMGIYRALS
En.aer.1436-EAE_15535-YP_004593297.1--36H-COG1/1-556      DDFEQTYNAYLQQ-NGKLYQIAVDASNSSYSSAIWTLIVVIIVVLAAIVGVWMGIHHILV
Xe.bov.105-XBJ1_1923-YP_003467827.1--36H-COG1/1-567       NAFNVEYDYYLNQ-NQKLYDEVAREADIAYRNIIISLISVFVLLISVMTVSWIGLRKALI
Serrat.1408-SerAS12_3073-YP_004501499.1--36H-COG1/1-555   NGFEDIYNTYLAQ-NDKLYQSAVEDSNSSFSFAIWTLVVVLVVVLAVIVLVWSGIHHILV
Er.bil.197-EbC_25350-YP_003741913.1--36H-COG1/1-559       NQFEKTYNDWLET-NNQLTAEGVEANSRAYEHTLWLLFAVLVISLLVIALAWSGIYKILL
Er.tas.1011-ETA_14650-YP_001907404.1--36H-COG1/1-554      DDFNAAYDIWLAK-NDTLAALGVAENQTAFHQAIWIILLGLVVTLLVIAGVWIGMRRILL
Sa.ent.407-STM4533-NP_463392.1--36H-COG1/1-553            DAFEKQYMAYMQQ-NDRLYDIAVEDNNSSYNQAMWVLVSVLIAVLVVIIAVWFGIKLSLI
Es.col.1836-Y75_p4240-YP_492486.1--36H-COG1/1-551         DGFEKQYVAYMEQ-NDRLHDIAVSDNNASYSQAMWILVGVMIVVLAVIFAVWFGIKASLV
En.bac.2261-D782_3913-YP_007342002.1--36H-COG1/1-553      DRFEQAYVDYLQQ-NDRLYDLAVADNNRSYDQAMWILAGVMIAVLLAIAIVWFGIRKTLI
Ci.kos.578-CKO_03442-YP_001454958.1--36H-COG1/1-554       DGFEKQYVNYMQQ-NDRLYDIAVEDNNRSYSQAMWVLASVLIVVLLVIVAVWFGIKLSLI
Cr.sak.579-ESA_03402-YP_001439456.1--36H-COG1/1-555       DGFEKAYTDYMAQ-NDRLYDIAVEGSNSSYSMAIWILIGVLAVVLGVIVCVWFGIQNTLI
Pantoe.297-Pat9b_1606-YP_004115479.1--36H-COG1/1-558      DKFAKDYDAWLGY-NKILALRGIDDNLAAYHQSVWMIVAMLLVTLVLIALVWSGMRAALI
Cr.tur.6-CTU_05670-YP_003208930.1--36H-COG1/1-555         DGFEKAYTEYMAQ-NDRLYDIAVEGSNSSYSMAIWILVGVLAVVLGVIVCVWFGIQNTLI
Sa.bon.1474-SBG_3944-YP_004732724.1--36H-COG1/1-553       DAFEKQYMAYMQQ-NDRLYDIAVEDNNSSYNQAMWVLVSVLIAVLVVIVAVWFGIKLSLI
En.asb.1498-Entas_0547-YP_004827084.1--36H-COG1/1-554     DGFEKQYVNYLQQ-NDQLYKTAVEDSNSSYTQAIWVLISVLIAVLVVIVAVWLGIKQALI
Ci.rod.62-ROD_48461-YP_003368233.1--36H-COG1/1-554        DSFEKHYDAYQAD-IDHLYQLAVSDNNHSYGQAMWVLSIILIAVLAVIIAVWFGIKVSLI
Ed.tar.1771-ETAF_1249-YP_005698855.1--36H-COG1/1-529      DSFEKAYETYLAQ-NDSLYHQAVASNNDAYHMALWILLAVMVVVAAAIVLVWMGIHHTLI
En.638.865-Ent638_0513-YP_001175251.1--36H-COG1/1-586     DGFEKEYVNYLQQ-NDHLYETAVADSNSSYTQAIWVLISVLIAVLVVIVSVWLGIKQSLI
Ed.ict.1187-NT01EI_1462-YP_002932883.1--36H-COG1/1-532    DQFEASVAKLTQH-VEGELKEGAEINHRSYQLSMVLNLTAVVLLVLLVLIAIWWLRNMLL
Ye.ent.378-YE2573-YP_001006777.1--36H-COG1/1-545          DLFEADFLQYLQY-ANEVIAEAGTQNQQAYHLAMWIFAGAILMVIAMAISSLIWLRTMFV
Ed.tar.1771-ETAF_1250-YP_005698856.1--36H-COG1/1-530      DQFEASVAKLTQH-VEGELKQGAEINRNSYHLSMALNLAAVVLLALVMLIAIWWLRNMLL
Mo.mor.2189-MU9_1763-YP_007505182.1--36H-COG1/1-524       DRMGAVVNDYLAL-LDEESRSAIESGTFYNRMAWIMFFGVTATILLISGLAHFWMRYKVI
En.aer.1436-EAE_15540-YP_004593298.1--36H-COG1/1-536      DRFEADFVQYLQL-AKATTDEASASSQQAYGWSIWLVAGAVLMLLVVTLSAMWWLRTMLV
En.638.865-Ent638_2456-YP_001177176.1--36H-COG1/1-533     AAFDKSFNAWQQD-INQFVAKASGDSQTSYHMSGVIFATMVVLAALLTGASLLWSRRMIV
Se.pro.864-Spro_2982-YP_001479211.1--36H-COG1/1-541       DLFEADFVQYLQL-VSANVSEANAANQRSFTLSGWLVAGAVLMLIVVTGSAMWWLRNMLV
Es.col.1836-Y75_p1861-YP_490147.1--36H-COG1/1-533         NAFDVNFEAWQLE-INHVLEAASAQSQRNYQISALVFISMIIVAAIYISSALWTRKMIV
Serrat.1901-SerAS13_3074-YP_006025913.1--36H-COG1/1-541   DLFEADFVQYLQL-VNANVDAANAANQQSFTLSGWLVGGAVLMLILVTGCALWWLRNMLV
Ye.pes.133-YPZ3_2023-YP_003568195.1--36H-COG1/1-536       DLFEADFIQYLQY-ANEVIADAGQQNQQAYQLSIWIFSGAILMVITMAISSLIWLRNMFV
Serrat.1408-SerAS12_3072-YP_004501498.1--36H-COG1/1-541   DLFEADFVQYLQL-VNANVDAANAANQQSFTLSGWLVGGAVLMLILVTGCALWWLRNMLV
Se.mar.2260-D781_2758-YP_007345194.1--36H-COG1/1-534      TQFETELTHYLQR-LGGEMTQAQQTNQRAYTLGSWMLTGAVLMLFVVAGCAIWWQQSVLL
Ye.pse.585-YPTB2400-YP_070914.1--36H-COG1/1-536           DLFEADFIQYLQY-ANEVIADAGQQNQQAYQLSIWIFSGAILMVITMAISSLIWLRNMFV
Se.ply.1407-SerAS9_3071-YP_004506451.1--36H-COG1/1-541    DLFEADFVQYLQL-VNANVDAANAANQQSFTLSGWLVGGAVLMLILVTGCALWWLRNMLV
Pr.stu.1965-S70_18140-YP_006218125.1--36H-COG1/1-521      EQMAAVIDQYLNL-LNTDSQNSLDSANFYNKVAWFMFFAVTFIILAVSGLAHFWMKRKLI
En.bac.2261-D782_1749-YP_007339930.1--36H-COG1/1-533      ARFDADYQGWQRH-IDAYIDDARAESQRDYQRSQLMFLTMVVLAVLITAGSLLWSRRMIV
En.asb.1498-Entas_2590-YP_004829104.1--36H-COG1/1-533     AAFDGSFNAWQQD-INQFVERAGAASRTSYHMSGVIFAVVVILAALLTGGALFWSRRMIV
En.asb.1498-Entas_3672-YP_004830170.1--36H-COG1/1-545     RAFGAAAESFNKA-AGDRLDSTRVVVDGLTAMTRTVIIAATIIGLLILLFTDRYLVAMLV
Ci.kos.578-CKO_01067-YP_001452646.1--36H-COG1/1-537       DAFDANVTTWQQD-INRVLETASEQGKDNYHRSAIIFITMVVVAALLTTGALFWSRKMIV
En.clo.1544-EcWSU1_02810-YP_004952663.1--36H-COG1/1-538   AAFDASFNAWQQN-INQFVQRAGNDSRKNYHMSGVIFAVVVILAVLLTGGALYWSRRMIV
Sa.ent.407-STM3152-NP_462067.1--36H-COG1/1-547            RAFGASAERFSHE-AGAMLDNTRVMVDGKTYTIRILLITAVILGIAILIFTDRYLVAMMV
Pe.atr.485-ECA1332-YP_049438.1--36H-COG1/1-555            RQFGASLSAFSNA-SAEKFDAAGVRFEQITTVGQNILLSGLFIGLIMLFLTDRYLVVCLV
Pe.car.1139-PC1_1208-YP_003016792.1--36H-COG1/1-555       RQFGASLSAFSNA-SAEKFGAAGVRFEQITTIGQNILLSGLVIGLIMLFLTDRYLVVCLV
En.638.865-Ent638_3407-YP_001178118.1--36H-COG1/1-545     RAFGAAAEKFNHE-AGNTLEETRVVVDGLTSMTRTVIISAMVLGLLILIFTDRYLVTMLV
Pa.vag.184-Pvag_1413-YP_003931052.1--36H-COG1/1-554       RAFGISAEKFNQG-AAEKLDKTRVEVDHLTAMTKTIITITVIIGLLILLFTDRYLVAMLV
Ph.asy.1114-PAU_02684-YP_003041518.1--36H-COG1/1-544      QQLESDFNHYMQY-IGEEITTAVQDGRFYYQIATAMFAGAILMVLVVAWAAHWWLKRNLL
Pa.ana.1905-PAJ_1316-YP_005934192.1--36H-COG1/1-556       RAFGASTENFNKV-ASVKLGQTRVEVDRMTAMTKTLISVTVVIGLLILLFTDRYLVKMLV
Ci.kos.578-CKO_04394-YP_001455885.1--36H-COG1/1-561       RAFGASAERFNSA-AGTMLDSTRVMVDGKTSIIRTLIITAVILGIVILFFTDRYLVTMLV
Sa.bon.1474-SBG_2750-YP_004731563.1--36H-COG1/1-547       RAFGASAERFNHE-AGGMLDNTRVMVDGKTYTIRVLLITAVILGIAILIFTDRYLVAMMV
Ra.aqu.1678-Q7S_16025-YP_005402985.1--36H-COG1/1-554      RSFGSSMEKVSGQ-IAVDFDAAQRQSTHLTSFSKVILLTAFGIGLVILLLTDRYLMALV
Rahnel.1320-Rahaq_3177-YP_004213898.1--36H-COG1/1-554     RSFGSSMEKVSGQ-IAVDFDAAQRQSTHLTSFSKVILLTAFGIGLVILLLTDRYLVMALV
Pectob.2320-W5S_3114-YP_006284066.1--36H-COG1/1-555       RQFGASLSAFSSA-SAEKFAAAGVRFEQITTVGQNILLSGLFIGLIMLFLTDRYLVVCLV
Ye.ent.378-YE2971-YP_001007160.1--36H-COG1/1-552          RQYGSVAENFNQA-ASKAIGVAKEQFAHLTKVSSMTLISALVAGLVILLATDRYLLANLV
Er.bil.197-EbC_20190-YP_003741400.1--36H-COG1/1-556       REFGATVDKFNLA-AGQKLDTTRVTVDHLTDVTKIVIVLSVIVGLFILLFTDRYLVKMLV
Ph.lum.1262-plu1854-NP_929127.1--36H-COG1/1-544           QQLESDFNHYMQY-IGEVITTAVQDGRLYYQISVAMFVGAILMVLVVAWAAHWWLKKNLL
Er.bil.197-EbC_25340-YP_003741912.1--36H-COG1/1-528       DKFQVQYNQWMKH-IDLLREHANQAGKGFYAQSKIIFIVAVVLCLLVTFSGIWWVRRAVI
Di.zea.1140-Dd1591_2937-YP_003005238.1--36H-COG1/1-564    RQFNVSFTNFNKA-GSTMFVDAGERLETITGVGQNILLAGLIAGLLMLFLTDRYLVAYLV
Er.tas.1011-ETA_14670-YP_001907406.1--36H-COG1/1-518      DQFLLQYNLWMKH-IDTLRDHAGDASKGFYFQSKVIFAASVVLSLLLTLGALIWVKRALI
Sa.ent.404-STY4234-NP_458344.1--36H-COG1/1-547            DDMEQVYDQWLSQ-NAQLIKLASDQNQSSFTQMQWTLGIILLIVLIVLAFIWLGLQRVLL
Sa.ent.407-STM3577-NP_462478.1--36H-COG1/1-547            DDMEQVYDQWLSQ-NAQLIKLASDQNQSSFTQMQWTLGIILLIVLIVLAFIWLGLQRVLL
Sa.bon.1474-SBG_3170-YP_004731982.1--36H-COG1/1-547       DDLEQIYKQWLSQ-NAQLIKLASDQNQSSFTQMQWTLGIILLIVLAVLAFIWQGLQHVLL
En.asb.1498-Entas_2591-YP_004829105.1--36H-COG1/1-555     NALGAALGEYAKA-SNDLYHTSFTESQNDYRFAKWQMAVLALALVIVLVGVWYGIRHILL
En.638.865-Ent638_1858-YP_001176586.1--36H-COG1/1-552     DKMEAVYTRWRAE-NNTLLKAAALENQNSFTSMQWTLGTILLVVIGVLIVFWMGLQHLLL
Ci.rod.62-ROD_19331-YP_003365490.1--36H-COG1/1-553        HALDETLDKYATA-SEKLYRHEFDESARDYRFAQWQLGILSVVLASILVVAWYGIRQILL
Di.dad.235-Dda3937_01559-YP_003882087.1--36H-COG1/1-566   RQFNVSFTNFNKA-GSTMFVDAGNRLESITGVGQNILLAGLIAGLVMLFLTDRYLVACLV
Er.bil.197-EbC_37780-YP_003743156.1--36H-COG1/1-553       DDLETSYDSWRTQ-NRQLLAVGMTENQQGFSHMMWTLATVVVVLLLLVVAVWVVIKRVLL
Pantoe.297-Pat9b_2001-YP_004115872.1--36H-COG1/1-558      RAFGASAESFNQA-AGIKLDDTRVTVDKLTNVTKVIILTAVVVGLLILLFTDRYLVAMLV
```

```
En.clo.1544-EcWSU1_01962-YP_004951821.1--36H-COG1/1-553  DDMEGVYNRWRAE-NNTLLKAATEENQSSFTQMQWTLAAIFLAVIAVLVVIWQGLQHLLL
En.asb.1498-Entas_1879-YP_004828403.1--36H-COG1/1-554    DNMEAVYNRWRAE-NNTLLKAAAEENQSSFTHMQWTLAAIFLAVVAVLVIIWQGLQHLLL
En.bac.2261-D782_1748-YP_007339929.1--36H-COG1/1-547     NAMGEAMGVYRAQ-SESLYQQAFTTSSHDYQFARWQLMILALVLAAVLLAVWFGIRRILL
Es.col.1836-Y75_p1862-YP_490148.1--36H-COG1/1-553        NAMGEAFAQYALS-SEKLYRDIVTDNADDYRFAQWQLAVIALVVVLILLVAWYGIRRMLL
Cr.tur.6-CTU_33010-YP_003211664.1--36H-COG1/1-555        DEMEEVYTTWRAQ-NTTLLQAAAQENQQSFTGMLWTLGIIAIIVVLAIATIWQGLRHLLL
Cr.tur.6-CTU_25780-YP_003210941.1--36H-COG1/1-536        NALEKAVTRYDDV-NVRLAQQAWDESRSDFRLAQWQTAILAVLVVLVIALVWYGIRHVLL
En.clo.1544-EcWSU1_00403-YP_004950264.1--36H-COG1/1-549  TAMQNVYREWREA-QAVLTDRGIKDNENDYKRILWILSAVMLLVIAVIVSSWIAMRRVLL
En.638.865-Ent638_2457-YP_001177177.1--36H-COG1/1-555    NALGTALKEYDTA-SSQQYLTALTETRDDYRFAKWQMAILALALVIVLVGVWYGIRHILL
Sa.bon.1474-SBG_1755-YP_004730609.1--36H-COG1/1-553      NALGEALGNYARA-SEKQYRQTFDQSAHDYRFAQWQLGILAVVLVLILVVVWYGIRQALL
Pr.mir.1265-PMI1665-YP_002151396.1--36H-COG1/1-548       TAMENSFNTYMNY-VQHEVADSIEQGHHFYTIAILMFIGAITMVIVVSIASHRWLSFNII
Cr.sak.579-ESA_01348-YP_001437444.1--36H-COG1/1-556      NALEKAVMRYDDV-NVRLAQQAWDESRSDFRVAQWQTGILAVLVVLVIALVWYGIRHVLL
Cr.sak.579-ESA_00560-YP_001436686.1--36H-COG1/1-555      DEMEEIYTTWRAQ-NTTLLQAAAQENQQSFTGMLWTLGVIAVIVILAIATIWQGLRHLLL
Sa.ent.407-STM1919-NP_460876.1--36H-COG1/1-553           NALGEALGNYARV-SENLYRQTFDQSAHDYRFAQWQLGVLAVVLVLILMVVWFGIRHALL
Ci.kos.578-CKO_01066-YP_001452645.1--36H-COG1/1-552      NALGEALGKYAGV-SEKLYSKAFDESARDYHFAQWQLGILAVVLVLILVVVWYGIRHTLL
En.asb.1498-Entas_0424-YP_004826963.1--36H-COG1/1-549    NAMQKVYHEWRTA-QAKLANKGIKDNESDYKRILWALSVIMLIVVGVIIASWIAMRRVLL
Pantoe.297-Pat9b_1273-YP_004115150.1--36H-COG1/1-553     DDLEDAYKQWRAQ-NVELLAQGSAENESAWQHVLAMVAVMAVVVLLLLAAVWVLVRRILL
Ed.tar.1771-ETAF_2264-YP_005699859.1--36H-COG1/1-555     SGFMSSLDEASDYVANQIISPASQAAAANYRQMIPLTIGFMILFLAMTALVLYWVRNHII
Pa.ana.1905-PAJ_3534-YP_005936409.1--36H-COG1/1-553      DNLDKAYQTWRSA-NYQLMKQIAAENDSNYSNMLWTLLIIAIIALVTVATVWAGLRTLLL
Pa.vag.184-Pvag_1203-YP_003930842.1--36H-COG1/1-552      DQLDTAYEAWRAV-NIQLMKDTSLHNQRSLTDALWALLAIGLITGVIGVAVWMGLQKLLI
Pantoe.297-Pat9b_1605-YP_004115478.1--36H-COG1/1-556     VAMKGTYETWRTK-QGELAAAGVAQNERAFTTMMWLLGTMVAVVIAVIVGCWFGLRSVLI
Cr.sak.579-ESA_00210-YP_001436348.1--36H-COG1/1-557      NAMQVVYREWRAE-QAKLASKGVKDNESDYERILWILSAIMVMVLAVIIISWVAMRRVLL
Er.tas.1011-ETA_pET460340-YP_001905943.1--36H-COG1/1-543 REFGASVEKFNTA-AAAKLDQTRINGEHLISTTKTVLILAMVLGIAILLLTDRYLVAMLV
Cr.tur.6-CTU_36610-YP_003212024.1--36H-COG1/1-557        NAMQEVYREWRAD-QAKLSSQGVKDNESDYQRILWILSTIMVMVLAVIIISWVAMRRVLL
Pe.car.1139-PC1_1159-YP_003016743.1--36H-COG1/1-556      VAMQDAYITWRAA-QNQLSDDGLKENHAAFETMLWLLGALSVVVVLVIILSWIGLQRILL
Er.tas.1011-ETA_14640-YP_001907403.1--36H-COG1/1-554     VAMQAVYTKWRGQ-QNRLIEQGIKDNQQAYNHMLWAVAAMITLVLALIVLCWFGLRSILI
Pa.vag.184-Pvag_1725-YP_003931362.1--36H-COG1/1-556      SAFLTLYREWRAD-QSRLTTLGVAENSSAYTRMMWILSAIMAAVVAVIVLCWFGLRKIVI
Ed.ict.1187-NT01EI_3742-YP_002935104.1--36H-COG1/1-525   EAMAKIYAQWRAQ-NHGLLQQGIDANQRSFGRMVWTLAGIALAVVLLIIILAGVAARGILL
En.638.865-Ent638_0380-YP_001175119.1--36H-COG1/1-549    QAMQKVYREWREA-QAKLTDKGIVDNENDYQRILWILSAIMIVVVGVIITSWIAMRRVLL
Er.bil.197-EbC_25360-YP_003741914.1--36H-COG1/1-556      NDMQQAYTLWRTA-LNEITVQGVQANEAAYSKMLWVIGTIMVLVVALIALCWVGLRKILI
Er.tas.1011-ETA_16910-YP_001907630.1--36H-COG1/1-543     REFGASVEKFNTA-AAAKLDQTRINGEHLISTTKTVLMLAMVLGIAILLLTDRYLVAMLV
Es.fer.1173-EFER_3055-YP_002384154.1--36H-COG1/1-603     NGFEKQYIEWMQQ-NDRLYDIAVEDNDSSYSHAMLLLAGILIAVLAVIVAVWFGIKHSLV
Xe.nem.162-XNC1_1619-YP_003711880.1--36H-COG1/1-520      RRMEIAYNNYMQY-LNSNIVEVRADAAFYYKIALAMFFGSILMVFTVAITAHWWLKRNLL
En.clo.1544-EcWSU1_02811-YP_004952664.1--36H-COG1/1-559  NALGAALGEYAKA-SGDLYHSAFTQSQNDYRFAKWQMAVMALALVIVLVGVWYGIRHILL
Di.dad.235-Dda3937_03500-YP_003882750.1--36H-COG1/1-553  VEFGGVADKYVTA-IQS--DDAIISAEKHIAINKDLLLVALIIGVIVLFLSDRYLVNYLV
Xe.bov.105-XBJ1_1924-YP_003467828.1--36H-COG1/1-523      RQLEVAYNNYMQY-LGHNISTAVSDGAFYYKVSIAMFFGSILMVFVVSASAHWWMRRNLL
Rahnel.1320-Rahaq_1831-YP_004212576.1--36H-COG1/1-556    DQFQKDVGDYTAY-VSARFGEAQTQTAASYQNVMINTSVALVLLISLTVLALVVTRRILF
Ra.aqu.1678-Q7S_08965-YP_005401598.1--36H-COG1/1-556     DQFQKDVGDYTAY-VSARFGEAQTQTAASYQNVMINTSVALVLLIALTVLALVVTRRILF
Cr.sak.579-ESA_01126-YP_001437230.1--36H-COG1/1-514      RAFGASAEKFNAA-AGAKLDATRERVDSMTVTTKTVIIIAVIIGLLLLLLTDRYLVAMLV
Pe.atr.485-ECA2712-YP_050803.1--36H-COG1/1-560           VAFGSSMDKYQAA-IISSSETSMKEVYDLVDWSNYILLAAVIIGLLILLLTDRYLVNYLV
En.clo.1544-EcWSU1_00560-YP_004950421.1--36H-COG1/1-597  DGFEKQYVNYLQQ-NDKLYQMAVENSNSSYSQAIWVLISVLIAVLVVIVAVWLGIKQALI
Di.dad.235-Dda3937_03498-YP_003882752.1--36H-COG1/1-561  VAFGEVAQKYAAV-VTN--SDYIDTVNSHNSWTRNVLILALVISVVIFLVSERYLTKYLV
Cr.tur.6-CTU_27880-YP_003211151.1--36H-COG1/1-514        RAFGASAEKFNAA-AGAKLDATRERVDSMTVTTKTVIIIAVIIGLLLLLLTDRYLVALLV
Pe.car.1139-PC1_1669-YP_003017246.1--36H-COG1/1-560      VAFGANMEKYQTA-ILSSSETSMNEVYGLVGWSNHILLGAAVLGLLILLLTDRYLVNYLV
Ra.aqu.1678-Q7S_14450-YP_005402672.1--36H-COG1/1-551     RNFSAAIEKYNDK-ITSLNAEANHRITQWVAWCQYILIGAMALSVVIMLLSDRYLANFLV
Ci.kos.578-CKO_03622-YP_001455137.1--36H-COG1/1-517      TAMQDVYLEWRAA-QAELTNAGIQQNEQDYQRILWILAAVMLAVVAVIVISWLAMQRVLL
Di.zea.1140-Dd1591_2408-YP_003004728.1--36H-COG1/1-557   VEFGELTEKYAAA-IQS--DNTIMDVEHYIAISKNILLAALAIGVGVLFLSDRYLVNYLV
Di.zea.1140-Dd1591_2406-YP_003004726.1--36H-COG1/1-559   WAFGDAAQKYTAA-VTN--ADFLGTVNEHNSWTRNVLILALAISILIFLISERYLTRYLV
Ed.ict.1187-NT01EI_2801-YP_002934203.1--36H-COG1/1-554   SGFMASLDEASDYVANQIINPASQAAAANYRQMIPLTIGFMILFLVMTALVLYWVRNHII
Pe.car.1139-PC1_1384-YP_003016966.1--36H-COG1/1-559      LAFGEDIKRYSDG-ITA--SSLIPSVNEHNTQNRNAMIAVMVIGLIVLIFTEYYLRNYLV
Rahnel.1320-Rahaq_2866-YP_004213595.1--36H-COG1/1-551    RNFSAAIEKYNDK-ITSLNAEANHRITQWVAWCQYILIGAMALSVVIMLLSDRYLANFLV
Pe.atr.485-ECA1281-YP_049387.1--36H-COG1/1-556           VAMQDAYITWRAA-QNQLSDDGLQENHVAFETMLWLLGALSVVVVLVIILSWIGLQRILL
Pectob.2320-W5S_1886-YP_006282848.1--36H-COG1/1-549      VAFGNNMDKYQTA-IMSSSETSMSEVYGLVNWSNYILLCAVVLGLLILLVTDRYLVHYLV
Pectob.2320-W5S_1696-YP_006282659.1--36H-COG1/1-559      LTFGDDIKRYSDG-ITA--SSLIPTVNEHNDKNRNALIAVMVLGLIVLIFTEYYLRNYLV
Di.zea.1140-Dd1591_2407-YP_003004727.1--36H-COG1/1-555   VEFGGIAEKYATA-IQS--DEAIIQASQRIAINKNVLLTALIMGIIVLFLSDRYLVNYLV
Pe.atr.485-ECA1509-YP_049611.1--36H-COG1/1-559           LTFGDDIKRYSDG-ITA--SSLIPSVNEHNDKNRNVLIVVMLIGVIVLIFTEYYLRNYLV
Pa.vag.184-Pvag_1724-YP_003931361.1--36H-COG1/1-520      VAMQGSYGEWRAR-QTELSDQGMAQNHKAFTQMMWLLGTIGVAVIVVILACWFGLRQVLI
Pa.ana.1905-PAJ_1541-YP_005934417.1--36H-COG1/1-520      QAMQATYSAWRLK-QSELSAHGAEQNQQAFTQMMWMLGMVGVLVIVVILGCWFGLRQLLI
Mo.mor.2189-MU9_1596-YP_007505015.1--36H-COG1/1-519      KEFGAASLEFTQV-AETRIEQTRTLVDGLMTTTRIVIISGTLFGLLILIFADRYLVKMMQ
Pr.mir.1265-PMI2808-YP_002152509.1--36H-COG1/1-563       DNFTNSLYEATDFITDDVVNPSVIEAEDNYHEMLWIASLFMGIFLIFTTLVLFWIRKHII
Di.zea.1140-Dd1591_0395-YP_003002764.1--36H-COG1/1-556   VKFTTAINEYNQI-IKNLKTEAQGRISAWVYWCKVTLIVAMIIGLGVVLLTDRYLAIHMG
En.clo.1544-EcWSU1_03786-YP_004953635.1--36H-COG1/1-515  RAFGAAAENFNNA-AARALDSTRVVVDSLTSMTRTVIITATIIGLLILLFTDRYLMAMLV
Pectob.2320-W5S_3170-YP_006284116.1--36H-COG1/1-556      VAMQDAYITWRAA-QNQLSDDGLQENHVAFETMLWLLGALSVVVVLVIILSWIGLQRILL
Di.dad.235-Dda3937_02184-YP_003884754.1--36H-COG1/1-556  VKFTTAINEYNQI-IKNLKTDAQARISAWVYWCKVTLIVAMIVGLCVVVLTDRYLAIQMG
Ci.rod.62-ROD_32641-YP_003366748.1--36H-COG1/1-517       KAMQSVYREWRAA-QTELANAGIQDNEKDYQRILWILSFILLAVMAVITISWMAMQRVLL
Di.dad.235-Dda3937_03499-YP_003882751.1--36H-COG1/1-561  VEFGDVANKYVNA-IQS--DNTIVALKAHITINKNVLLAALIIGVIVLLLSDRYLVNYLV
Di.dad.235-Dda3937_00105-YP_003883346.1--36H-COG1/1-561  DAFQQSFDAYFRQ-NDALNDSAVSDSGQNYHYSIIVIAVCVLILFFATTSAWVYIRRVLL
Pantoe.297-Pat9b_1604-YP_004115477.1--36H-COG1/1-555     SAFLLDYTQWRAD-QDRLTEAGVAANLTAYKRMMWLLGLVMAVVVAVIVLCWFGLRKVLI
Er.tas.1011-ETA_14660-YP_001907405.1--36H-COG1/1-560     NGFEQAYDNWLQH-TAQLLSAGSAENDHAYQRAIWVLLSVLVVVLALIAAAWSGIHHILL
Pr.mir.1265-PMI2809-YP_002152510.1--36H-COG1/1-575       ERFTQSLYEATDHLSDNVVNHAIEAAQGNYQRALIFAFIFMSVFILFTVFVMLWIRHHIV
Ed.tar.1771-ETAF_3053-YP_005700648.1--36H-COG1/1-516     DAMAEIYAQWRAQ-NHGLLQQGIDANQRSFERMVWTLAGIALAVVLLIIILAGAAARGILL
Se.pro.864-Spro_1415-YP_001477647.1--36H-COG1/1-546      TYFMSALDDTSAYVANDIITPAAAAAQTSYRQMVPLSLAFIFIFILLTGAVLVWIRKYVL
Di.zea.1140-Dd1591_1804-YP_003004134.1--36H-COG1/1-562   DAFQHAFDAYFQQ-NDALNDNAVNDSGQNYYYSIIVIAVCVLILMLATSSAWVYIRRILL
Di.dad.235-Dda3937_03501-YP_003882749.1--36H-COG1/1-575  VEFGELTEKYAAA-IQA--DSTITNVGRYIEISKNVLLAALIIGVIVLLLSDRYLVNYLV
Pantoe.297-Pat9b_0851-YP_004114731.1--36H-COG2/1-524     NTYLDSANAFAAS-QSEQLQNEGISIIADGSTAIMVTLIFSAVAIVASIILGFLLTRSIV
Pa.vag.184-Pvag_0291-YP_003929953.1--36H-COG2/1-525      NKFLQDAVNFANS-QDSQLRSEGQGIVENGQSAITITLIFSALAILAAIMLGYFLTRSIV
En.clo.1544-EcWSU1_03886-YP_004953735.1--36H-COG2/1-519  QAYKANVQALIAI-QNQEMQSAGVQVEGDFKSNRLLLILITLFSVATGSLIGWFIVRSIT
Cr.tur.6-CTU_04790-YP_003208842.1--36H-COG2/1-515        NEYKSRVQALIKI-QDNHMREAGVTVAKDFSGNRALTIGLTLLCVVLGSLMGWLIVRAIT
Ci.kos.578-CKO_04484-YP_001455975.1--36H-COG2/1-521      QAYKAKVQELIAI-EDTLMRNAGASVDQDFRTNRAQLILLALISIAAGVIMGWYIVRSIT
Cr.sak.579-ESA_03488-YP_001439539.1--36H-COG2/1-515      NEYKSRVQALIKI-QDNHMREAGVTVAKDFSSNRALTIGLTLLCVVLGSLMGWLIVRAIT
Ci.rod.62-ROD_48071-YP_003368199.1--36H-COG2/1-521       RAYKEKVQAFIAI-EDGQMREAGIQVEKDFNANLLLLVGMVLAGVIVSCIAGLYIARSIT
Sa.bon.1474-SBG_2851-YP_004731664.1--36H-COG2/1-521      KIYKDKVQELIAV-QDAQMQHAGVQVEGDFKTNRTLLITLALISIAAGSVMGWYIVRSIT
Sa.ent.407-STM3216-NP_462130.1--36H-COG2/1-521           KVYKDKVQELIAV-QDAQMHNAGVQVEGDFKTNRTLLITLALISIAAGCVMGWYIVRSIT
En.asb.1498-Entas_3753-YP_004830248.1--36H-COG2/1-519    KAYKDKVKELISV-QNGQMQSAGAQVDGDFKNNRLWLIVITLFSVGAGSLIGWYIVRSIT
Mo.mor.2189-MU9_3389-YP_007506806.1--36H-COG2/1-521      RAYRDNVMQLIAI-QDTHMKEAGNQVESNFKTNRLLLIALTIISAIISAVLARIIVVSIT
Sa.ent.404-STY3394-NP_457606.1--36H-COG2/1-522           KVYKDKVQELIAV-QDALMHEASVQVKEDFKNNRTLLITLALISIAAGGVIGWYIVRSIT
En.638.865-Ent638_3526-YP_001178235.1--36H-COG2/1-519    QAYKAKVQELIAV-QNNEMQNAGQQVDSDFKSNRLLLVLLTVLSVIIGSTIGWFIVRSIT
Pe.atr.485-ECA1774-YP_049874.1--36H-COG2/1-554           AKYTSKVNEFIDI-QDDKMSSSAQEVGESYRNALMVLATIIIISALAGLVIASLITRSVT
En.638.865-Ent638_2119-YP_001176845.1--36H-COG2/1-549    QEYNKTLRSLLEL-QNQIINDTHASIKKASNLTELVIVLSVLFGIVLGSLIAWYITRSIT
```

```
Pectob.2320-W5S_2813-YP_006283770.1--36H-COG2/1-554      EKYTSKVKEFIDI-QDDKMSSSAQEVSDSYKNALMVLATIILISALAGLIIASLITRSVT
Di.zea.1140-Dd1591_0695-YP_003003055.1--36H-COG2/1-561   REYRNAVRRLVDY-HNKAMLGTIDTMSSTYHDIRLVLLMILGGGVLLSIWVATAITRSVT
Pa.vag.184-Pvag_pPag30079-YP_003729820.1--36H-COG2/1-546 VDYLKTIADLVSF-QEEQMQTARSAAEDERNTATILLTSLSAAAVLLAAAAAWLITRSIT
Er.bil.197-EbC_38940-YP_003743272.1--36H-COG2/1-526      NTYLNLASAFADS-QSQQLVAEGALAGTEGNSAIQITLIFSALAILAAVLLGYFLARSIV
Ra.aqu.1678-Q7S_24891-YP_005419232.1--36H-COG2/1-554     NAYLDLATAFANS-QDQQLQAEGKTAIADGATAIQLTLIFSALAILMAAALGYFLTRSIT
En.asb.1498-Entas_2287-YP_004828805.1--36H-COG2/1-513    DNVKTAGDNSSAA-IRDIIALVKAHNDALAYSSSTITAIVGVIAILFGILVSWWVTRQIT
Rahnel.1320-Rahaq_4869-YP_004215574.1--36H-COG2/1-554    NAYLDLATAFANS-QDQQLQAEGKTAIADGATAIQLTLIFSALAILMAAALGYFLTRSIT
Di.dad.235-Dda3937_00027-YP_003883574.1--36H-COG2/1-554  QKYKDKVAEFIDY-QDDQMTSAREEVQRSYTDIRMVLWLVFIISIVAGGLIAWWITRSVT
Pr.stu.1965-S70_05790-YP_006215722.1--36H-COG2/1-520     REYREKVQQFIEV-QNHRMQASGVIVDGDYHFNKYLLITLAIISIVIACIMGWFITLSIT
En.638.865-Ent638_2100-YP_001176826.1--36H-COG2/1-512    DKVKTAGDDSSAS-IREIIALVKIHNDSLANSSSTITAIVGVVAVLFGILVSWWVTRQIT
Pe.car.1139-PC1_2526-YP_003018093.1--36H-COG2/1-554      AKYTSKVNEFIGI-QDDKMASSAQAVGESYKNALMVLATIILISALAGLIIASLITRSVT
Mo.mor.2189-MU9_702-YP_007504121.1--36H-COG2/1-525       DAYILSFRASRDH-GTETGNQLIQEFNTNYERQSNIILVFALLALTLAIVSGVMIIRNLT
Pe.car.1139-PC1_3443-YP_003018995.1--36H-COG2/1-511      EN------------INKLYFTEESRAGRNVINSDIQLGVITLIAIILGLTTAIVISRQIV
Pa.ana.1905-PAJ_0915-YP_005933791.1--36H-COG2/1-559      GDFMASLKEMQTF-QEARMSSAAAQARADRSGALMMLVVAGVVALLLAAVSAWVITRSIT
Pectob.2320-W5S_3736-YP_006284671.1--36H-COG2/1-510      EN------------INKLYFTEESRAGRNVISSDIQLGVITLIAIILGLTTAIVISRQIV
Pe.atr.485-ECA0183-YP_048310.1--36H-COG2/1-553           RQYRDAVKQLVNY-QDDAMDTSVEAMAEVYSNTRMILLLILALGAVFGALIAWSITRSVI
Rahnel.1320-Rahaq_3107-YP_004213828.1--36H-COG2/1-503    SA------------SNTLLDHEMNNLHNEVTRTISEMLVIIVATVVAGVLISWRMTRQLT
Pe.car.1139-PC1_4071-YP_003019622.1--36H-COG2/1-553      RQYRDAVRQLVNY-QDDAMDTSVEAMAEVYSETRIILLLILALGAVFGALIAWSITRSVT
Di.dad.235-Dda3937_03462-YP_003884507.1--36H-COG2/1-563  REYRNAVRQLVDY-QDNAMMSTVDAMSATYHDIRLVLLAILGIGVVLSVWVAMAITRSVT
Ra.aqu.1678-Q7S_15665-YP_005402913.1--36H-COG2/1-503     SA------------SNTLLDHEMNNLHNEVTRTISEMLVIIVATVVAGVLISWRMTRQLT
Pectob.2320-W5S_4505-YP_006285423.1--36H-COG2/1-555      RQYRDAVKQLVNY-QDDAMDTSIEAMAEVYSETRIILLLILALGAVFGALIAWSITRSVT
Pe.atr.485-ECA3642-YP_051730.1--36H-COG2/1-551           NGYLASVEALRDH-QRASIDQMGKNINAGASRGDLILAVTGVLSAIIGVLIAWVLTRSIV
Pe.car.1139-PC1_1417-YP_003016999.1--36H-COG2/1-554      SLYFTKLNELTAL-QVSFVDHAKQVTKEYIHDALLWMLILSVFSTVLGCLTAWLLTRSVT
En.asb.1498-Entas_4509-YP_004821569.1--36H-COG2/1-559    EDHIKQITDIISY-SNDMMKEGHDDIIKTYSKSLRLIISMVVLSLITGSLISWILTRSIL
Pa.ana.1905-PAJ_3064-YP_005935940.1--36H-COG2/1-544      TGYIASLQALKDH-QEARMQVAEQTSHSDRTNALRLLAGAGAVALFLAGLSAWLITRSIT
Pectob.2320-W5S_1733-YP_006282696.1--36H-COG2/1-535      TLYFTKLSELAAL-QVSFVDHSKQVTKEYIHDALLWMLVLSVFSTVLGCFTAWLLTRSVT
Pe.atr.485-ECA4334-YP_052421.1--36H-COG2/1-556           DAVFNALNDMVNW-QEKLTVEIANQSLKNATNAGSLMVIIALLSVALGAMISWWITRTIK
Di.zea.1140-Dd1591_1602-YP_003003935.1--36H-COG2/1-554   KKYKAKVAEFIDY-QDDQMSSAREAVQQSYSDIRIVLWLVVIVSIVAGGLIAWGITRSVT
Pectob.2320-W5S_4506-YP_006285424.1--36H-COG2/1-540      RNYRDSVKQLVNY-QDEAMNTSVETMADVYSSTRLILLLILVLGAAAGALIAWLITRSVT
Di.zea.1140-Dd1591_1453-YP_003003794.1--36H-COG2/1-532   KQGHALLSQLVAL-QVEVARDEYTSSVQRYQWLKTLAILAVLAGIGGAVVFGWLMVRGIV
Pectob.2320-W5S_0106-YP_006281117.1--36H-COG2/1-556      DSVFNALNNMVNW-QEKLTVEIADQSLKNANNAGTLMVIIALLSVVLGILISWWITRTIK
Di.zea.1140-Dd1591_0769-YP_003003127.1--36H-COG2/1-505   ES------------INQLYFTEETRAAQNISSSDIRLAIITLVAVILGLTAAIVISRQIV
Ra.aqu.1678-Q7S_18035-YP_005403387.1--36H-COG2/1-535     ALLLNTTNDYLQY-SNVRAKESLNTAQDAYQQTRLIFIVISLVALSLAVILGGIIIRSLF
Rahnel.1320-Rahaq_3579-YP_004214298.1--36H-COG2/1-535    ALLLNTTNDYLQY-SNVRAKESLNTAQDAYQQTRLIFIVISLVALSLAVILGGIIIRSLF
Pe.car.1139-PC1_4072-YP_003019623.1--36H-COG2/1-540      RNYRDSVKQLVNY-QNDAMDNSVETMAEVYNSTRMILLLILALGAVFGALIAWAITRSVT
Pe.car.1139-PC1_3464-YP_003019016.1--36H-COG2/1-551      NGYLASVEALRDH-QRASIDQMGQDINTGASRGDLILAVTGALSAIIGVLIAWVLTRSIV
Pe.car.1139-PC1_0111-YP_003015709.1--36H-COG2/1-556      EAVFKALNDMVNW-QEKLTVDIADQSLKNATHAGTLMVIIALLSVVLGAMISWWITRTIK
Pe.atr.485-ECA0182-YP_048309.1--36H-COG2/1-542           RKYRDSVKQLVNY-QDDAMNTSVEAMAEVYNSTRMILLIIILALGAVFGALVAWAITRSVT
Pectob.2320-W5S_3753-YP_006284688.1--36H-COG2/1-551      NGYLTSVEALRDH-QRASIDQMGQDINAGASRGDLILAVTGVLSAIIGVLIAWVLTRSIV
Pa.ana.1905-PAJ_2666-YP_005935542.1--36H-COG2/1-514      GQLLEALKEMTNI-QMEDSERSVENTSQNVHRATTILMILASLSVAISIAAGFIITRVLM
Ci.rod.62-ROD_16161-YP_003365194.1--36H-COG6/1-557       NGYNSVLLKAIQI-RMARANQLSEQAHQRTQLGMMFMTGAFGLALLLTLMTFMVIRRIVI
En.asb.1498-Entas_2095-YP_004828614.1--36H-COG6/1-563    DGYNDVLMKAIKI-RTDRANALTAQAHTRTQLGLMFMVGAFALALVLTAMTFIVLRRTVI
En.638.865-Ent638_1961-YP_001176688.1--36H-COG6/1-563    DAYNEVLLKAIKI-RTDRANLLTSEAHKRTQLGLMFMVGAFALALLLTAMTFVVLRRTVI
Cr.sak.579-ESA_01710-YP_001437800.1--36H-COG6/1-565      DDYNQVLAKALAS-RTARVEQLRSDASSRTHFSLMVMVAAFIVALVMSLLTFVVLRRVVI
Cr.tur.6-CTU_22440-YP_003210607.1--36H-COG6/1-565        DDYNQVLAKALAS-RTERVEQLRSDASTRTHFSLMVMVAAFAVALVMTLLTFVVLRRVVI
Ci.kos.578-CKO_01456-YP_001453025.1--36H-COG6/1-562      TAYNTVLLKAIEL-RTARANQLSDQAHQRSQLGLMFMIGAFALALVLTLLTFIALRRTVI
Rahnel.1320-Rahaq_1930-YP_004212674.1--36H-COG6/1-566    LANNVPLLAAIAY-RTQTANTINDKAQRNAVLGYTLMGGGFLLALVLTFITYLVMRKVII
Pantoe.297-Pat9b_2403-YP_004116261.1--36H-COG6/1-564     DAYNAVLLKAITI-RTNRADAINAEAAHQSRIGFIAMAVAFAAALVLVLLTFLFLRRVVI
Er.bil.197-EbC_26700-YP_003742048.1--36H-COG6/1-567      DAYNAVLLKAIAI-RTQRADAINKQAEAQTRLGFIMMAAAFAAALLLTLATFIFLRRVVI
Pectob.2320-W5S_1760-YP_006282723.1--36H-COG6/1-570      LAYNKYLLEAVAY-RTQRAKELNETAHKNALLGYSLMGSSFALATILTLLTFFLLRGVLI
Pa.ana.1905-PAJ_1621-YP_005934497.1--36H-COG6/1-568      NAYKAVLMEAIKL-RDVRSAQINDQAAEQSRIGFIAMGSAFVAALVLVLLTFVFLRRVVI
En.clo.1544-EcWSU1_02120-YP_004951978.1--36H-COG6/1-576  DAYNAVLLKAIKI-RTDRANALTAQAHSRTQLGLMFMVGAFALALVLTALTFIVLRRTVI
Ra.aqu.1678-Q7S_09815-YP_005401764.1--36H-COG6/1-566     LANNVPLLAAIAY-RTQTANTINDKAQRNAVLGYTLMGGGFLLALVLTFITYLVMRKVII
Ye.pse.585-YPTB2412-YP_070925.1--36H-COG6/1-579          DGYNEPLLKAVKY-RTEQANQINQSAHQEARLGYILMAGAFVLVILLTMIAFLVISKVII
Ye.ent.378-YE2588-YP_001006789.1--36H-COG6/1-579         DAYNEPLLKAVKY-RTERANEINQSAQQEARLGYMLMGGAFILAILLTLIAFLVISKVII
Di.dad.235-Dda3937_02787-YP_003883649.1--36H-COG6/1-572  EAYNKPLLKAIAF-RTERAKALNSNAQYQAMMGYTLMAVSFAIAIAMTLLTFLFLRGTLI
Pa.vag.184-Pvag_1800-YP_003931435.1--36H-COG6/1-568      DDYNAVLLKAIKI-RTDRASAITAEADRQSRIGFISMAVAFSAALVLVLMTFVFLRRVVI
En.bac.2261-D782_2307-YP_007340470.1--36H-COG6/1-558     DRYNAVLLKVIQI-RTDRANHLTELSLERTRLGLMFMAAAFVLALVLTIITFVVLRRIVI
Pe.atr.485-ECA1683-YP_049784.1--36H-COG6/1-573           LAYNKFLLEAVSY-RTQRAKELNETAHKNALLGYSLMGGSFALATILTLLTFFLLRGTLI
Pe.car.1139-PC1_2617-YP_003018183.1--36H-COG6/1-573      LAYNKFLLEAVAY-RTQRAKELNETAQRNALLGYSLMGGSFALATILTLLTFFLLRGILI
Di.zea.1140-Dd1591_1535-YP_003003868.1--36H-COG6/1-574   EAYNKPLLKAIAF-RTERAKALNNNAQHQAMLGYILMAVSFAIAIVMTLLTFLFLRGTLI
Di.zea.1140-Dd1591_3580-YP_003005868.1--36H-COG6/1-572   LAYNEVLLKVVAI-RTQRAEALNSEAQQESVFGYSVMAASFVVAVLVSLFTYLCLRRVII
Es.col.1836-Y75_p1397-YP_489687.1--36H-COG6/1-546        NAYTDILNKAVKI-RSTRANQLAELAHQRTRLGGMFMIGAFVLALVMTLITFMVLRRIVI
Pe.atr.485-ECA3902-YP_051990.1--36H-COG6/1-575           EEFSVPVRKKVTE-LTKAAQDINQRAEQNAQLGYWMMAGSFALSVIMAIMAYIMVRNVIL
Sa.bon.1474-SBG_1454-YP_004730321.1--36H-COG6/1-537      TAYNHVLLKAIEL-RTGRAKLLGEQAYQRTRLGMMFMIGAFTLALVLTLMTFMVLRRTVI
Sa.ent.407-STM1626-NP_460585.1--36H-COG6/1-541           AAYNHVLLKAIEL-RTERARLLSEQAYQRTRLGMMFMIGAFTLALVLTLMTFMVLRRTVI
Pe.car.1139-PC1_3679-YP_003019230.1--36H-COG6/1-567      EAFSVPVRKKVTE-LTKAAQDINLQAEQNAKLGYWMMAGSFALSIIMAIMAYIMVRNVIL
Pectob.2320-W5S_4020-YP_006284955.1--36H-COG6/1-575      EAFSVPVRKKVTE-LTKAAQDINDRAAENATLGNWMMAGSFALSIIMAIMAYIMVRNVIL
Di.zea.1140-Dd1591_0523-YP_003002884.1--36H-COG6/1-568   TNYAVQVRKEVKY-LTDHANNINAQAAGNARLGNILMGVSFILAIILAALTYLIIRRAIL
Di.dad.235-Dda3937_02665-YP_003881279.1--36H-COG6/1-572  LAYNEVLLKVVAI-RTQRAEALNSEAQQESVLGYSVMAASFVVAVLVSLFTYLCLRRVII
```

```
Pr.mir.1265-PMI1666-YP_002151397.1--36H-COG1/1-568       RPL----NSLLLNIKAFSEGDL--RPDIEV--HGRNEMSLLASGLKHMQQELIHTVRGVY
Di.dad.235-Dda3937_02779-YP_003883640.1--36H-COG1/1-566  QPL----TNLIEHIRHMAKGDL--TTRIDF--HGTNEMGILADSLRHMQTEFFTTVSAVR
Di.zea.1140-Dd1591_1542-YP_003003875.1--36H-COG1/1-566   QPL----HNLIEHIRHMAKGDL--STRIDF--HGTNEMGILADSLRHMQTEFVTTVSAVR
Pe.atr.485-ECA1691-YP_049792.1--36H-COG1/1-561           RPL----NSIVEHIRHIAKGDL--TKTTDF--HSSNEMGILADSIRHMQSEFVSTVSAVR
Se.pro.864-Spro_2983-YP_001479212.1--36H-COG1/1-556      RPL----NRMIEHIKMIAAGDL--TQPIAV--TSRNEMGVLAASLKHMQNELIDTVSGVR
Ph.asy.1114-PAU_02685-YP_003041519.1--36H-COG1/1-564     NPL----NKLLENIKALSTGDL--TQNIAI--SGRNEMSMLAVGLKHMQKEFINTVSSVR
Pr.stu.1965-S70_18135-YP_006218124.1--36H-COG1/1-561     EPL----RALIQRITTFAAGDL--TPSVDT--KATNEIGELARGVEHMQKELINTVRGVR
Mo.mor.2189-MU9_1762-YP_007505181.1--36H-COG1/1-561      HPL----NGLVDRIKIFAAGDL--TPTVAV--NGKNEIADLGRGVQHMQQELINTVRGVR
Pe.car.1139-PC1_2609-YP_003018175.1--36H-COG1/1-561      RPL----NNIVEHIRHIAKGDL--TKTTHF--HSSNEMGILADSIRHMQSEFVATVSAVR
Xe.nem.162-XNC1_1620-YP_003711881.1--36H-COG1/1-567      NPL----HKLLNNIKAFSEGDL--TQTITV--SGNNEMGMLAIGLKHMQEELINTVRSVH
Pectob.2320-W5S_1768-YP_006282731.1--36H-COG1/1-561      RPL----NNIVEHIRHIAKGDL--TKTTNF--HSSNEMGILADSIRHMQSEFVSTVSAVR
Pa.vag.184-Pvag_1723-YP_003931360.1--36H-COG1/1-558      RPL----RQSIEHIQHIARGDL--TQPVEI--TVRNEIGELLTSLQHMQQELARTVRTVR
Ye.pes.133-YPZ3_2024-YP_003568196.1--36H-COG1/1-557      HPL----NHLIAHIKHIANGDL--TQNIEV--HGRNEVGTLAASLKHMQSELVTIVGEVR
Ye.pse.585-YPTB2401-YP_070915.1--36H-COG1/1-557          HPL----NHLIAHIKHIANGDL--TQNIEV--HGRNEVGTLAASLKHMQSELVTIVGEVR
Ye.ent.378-YE2575-YP_001006778.1--36H-COG1/1-557         NPL----KHLIEHIKHIANGDL--TQNIEV--HSRNEMGTLAASLKHMQSELVTTVSDVR
Pa.ana.1905-PAJ_1540-YP_005934416.1--36H-COG1/1-563      RPL----KESIEHIQHIAGGDL--TQPINI--SVRNEIGDLLTSLQDMQRSLVRTVSTVR
Ph.lum.1262-plu1853-NP_929126.1--36H-COG1/1-564          NPL----NKLLENIKALSTGDL--TQNVTV--SGRNEMSTLAVGLKHMQKEFITTVSSVR
Serrat.1901-SerAS13_3075-YP_006025914.1--36H-COG1/1-555  RPL----NRMIEHIKMIAAGDL--TQPIAV--TSRNEMGVLAASLKHMQNELIETVSGVR
Sa.ent.404-STY2128-NP_456485.1--36H-COG1/1-553           NPL----ARVITHIREIASGDL--TKTLTV--SGRNEIGELAGTVEHMQRSLIDTVTQVC
Ra.aqu.1678-Q7S_08960-YP_005401597.1--36H-COG1/1-564     HPL----KNIVDTIRKIATGDL--THEIVV--KSRNEIGQLADSLKYMQDELIRTVSGVR
Se.mar.2260-D781_2759-YP_007345195.1--36H-COG1/1-555     RPL----NRIIEHIKHIAAGDL--TQSIDV--SSRNEMGVLAASLQHMQAELTNTVSGVR
Rahnel.1320-Rahaq_1830-YP_004212575.1--36H-COG1/1-564    HPL----KNIVDTIRKIATGDL--THEIVV--KSRNEIGQLADSLKYMQDELIRTVSGVR
Se.ply.1407-SerAS9_3072-YP_004506452.1--36H-COG1/1-555   RPL----NRMIEHIKMIAAGDL--TQPIAV--TSRNEMGVLAASLKHMQNELIETVSGVR
Ed.ict.1187-NT01EI_1461-YP_002932882.2--36H-COG1/1-555   RPL----NSLMGNIRQFAVGDL--SGRIEV--RGSNEMGQLADSLRHMQSEFIRTVSDVR
En.aer.1436-EAE_15535-YP_004593297.1--36H-COG1/1-556     RPL----NRMIEHIKRIASGDL--TQPIPV--TSRNEIGVLAASLKHMQNELIETVSGVR
Xe.bov.105-XBJ1_1923-YP_003467827.1--36H-COG1/1-567      NPL----NKLLENIKAFSKGDL--TQTINV--SGTNEMGMLAVGLRHMQDELIHTVRSVH
Serrat.1408-SerAS12_3073-YP_004501499.1--36H-COG1/1-555  RPL----NRMIEHIKMIAAGDL--TQPIAV--TSRNEMGVLAASLKHMQNELIETVSGVR
Er.bil.197-EbC_25350-YP_003741913.1--36H-COG1/1-559      RPL----RTNIEHIRHIASGDL--TQSIES--EGRNEMTQLADSLREMQQSLVRTVSDVR
Er.tas.1011-ETA_14650-YP_001907404.1--36H-COG1/1-554     RPL----QTSIDHIRRIASGDL--TGKITV--EGQNEMSSLAASLQHMQDELIQTVSAVR
Sa.ent.407-STM4533-NP_463392.1--36H-COG1/1-553           APM----NRLIESIRHIASGDL--VKRIDV--EGSNEMGQLAENLRHMQSELMRTVGDVR
Es.col.1836-Y75_p4240-YP_492486.1--36H-COG1/1-551        APM----NRLIDSIRHIAGGDL--VKPIEV--DGSNEMGQLAESLRHMQGELMRTVGDVR
En.bac.2261-D782_3913-YP_007342002.1--36H-COG1/1-553     LPL----QSLIESIRHIAGGDL--VRRIDV--EGRNEMGQLASTLRHMQGELARTVGEVR
Ci.kos.578-CKO_03442-YP_001454958.1--36H-COG1/1-554      APM----NRLIESIRHIANGDL--VKRIDV--EGSNEMGQLAQSLRHMQGELVRTVGDVR
Cr.sak.579-ESA_03402-YP_001439456.1--36H-COG1/1-555      TPL----NRIIESIRHIASGDL--ARQIDV--DGTNEMGQLAASIKHMQGELVRTVGEVR
Pantoe.297-Pat9b_1606-YP_004115479.1--36H-COG1/1-558     RPL----QQSIEHIRHIARGDL--TQQVEV--NVRNEMGDLLSSLQHMQQELARTVRTVR
Cr.tur.6-CTU_05670-YP_003208930.1--36H-COG1/1-555        APL----NRLIDSIRHIAGGDL--AREIEV--DGTNEMGQLAASIKHMQGELVRTVGEVR
Sa.bon.1474-SBG_3944-YP_004732724.1--36H-COG1/1-553      APM----NRLIESIRHIASGDL--VKRIDV--EGSNEMGQLADNLRHMQSELVRTVGDVR
En.asb.1498-Entas_0547-YP_004827084.1--36H-COG1/1-554    SPL----NRLIDSIRHIASGDL--VKRIDV--EGTNEMGELADTLRHMQGELVRTVGDVR
Ci.rod.62-ROD_48461-YP_003368233.1--36H-COG1/1-554       APL----NRLIDSIRHIASGDL--VKRIEV--EGSNEMGQLAESLRHMQGELVRTVGDVR
Ed.tar.1771-ETAF_1249-YP_005698855.1--36H-COG1/1-529     RPL----NSLMGNIRQFAAGDL--SGRIEV--CGSNEMGQLADSLRHMQTEFVRTVSDVR
En.638.865-Ent638_0513-YP_001175251.1--36H-COG1/1-586    SPL----NRLIDSIRHIASGDL--VKRIDV--EGTNEMGQLADTLRHMQSELVSTVGDVR
Ed.ict.1187-NT01EI_1462-YP_002932883.1--36H-COG1/1-532   APL----TLMRTHFERIARGDL--SAEIKV--DGRNEISQLYASLRSMQQDLAGTVLSVR
Ye.ent.378-YE2573-YP_001006777.1--36H-COG1/1-545         SPL----KTMRSHFDRIAKGDL--SAQISV--TGRNEISQMFASLRTMQQSLITTVSHVR
Ed.tar.1771-ETAF_1250-YP_005698856.1--36H-COG1/1-530     APL----TVMRAHFERIARGDL--SAEIKV--YGRNEISQLYASLRNMQQALAGTVLSVR
Mo.mor.2189-MU9_1763-YP_007505182.1--36H-COG1/1-524      NPL----TSMSEHFMQVAQGKL--NQKIEV--TTRDEIGVVFGQLKLMQESLAESVSNVR
En.aer.1436-EAE_15540-YP_004593298.1--36H-COG1/1-536     QPL----NIIRGHFERIASGDL--SAPIEV--YGRNEISQLFASLQRMQQSLIGTVGAVR
En.638.865-Ent638_2456-YP_001177176.1--36H-COG1/1-533    LPL----AIISSHFDSIAKGNL--ARPVSV--YGKNEISAIFASLKAMQGSLRETVTDVR
Se.pro.864-Spro_2982-YP_001479211.1--36H-COG1/1-541      RPL----NIMRSHFDRIAAGDL--ATPIQV--YGRNEISQLFASLQRMQQSLIGTVGAVR
Es.col.1836-Y75_p1861-YP_490147.1--36H-COG1/1-533        QPL----AIIGSHFDSIAAGNL--ARPIAV--YGRNEITAIFASLKTMQQALRGTVSDVR
Serrat.1901-SerAS13_3074-YP_006025913.1--36H-COG1/1-541  QPL----NIMRRHFDRIAAGDL--ATPIQV--YGRNEISQLFASLQHMQRSLIGTVGAVR
Ye.pes.133-YPZ3_2023-YP_003568195.1--36H-COG1/1-536      RPL----ATMREHFGRIAQGDL--SGQISV--TGRNEISQMFASLRTMQQSLISTVSNVR
Serrat.1408-SerAS12_3072-YP_004501498.1--36H-COG1/1-541  QPL----NIMRRHFDRIAAGDL--ATPIQV--YGRNEISQLFASLQHMQRSLIGTVGAVR
Se.mar.2260-D781_2758-YP_007345194.1--36H-COG1/1-534     GPL----KSMRRHFERIAGGDL--ATPVVV--TGNNEISALFAGLRQMQQSLTHTVGAVQ
Ye.pse.585-YPTB2400-YP_070914.1--36H-COG1/1-536          RPL----ATMREHFGRIAQGDL--SGQISV--TGRNEISQMFASLCTMQQSLISTVSNVR
Se.ply.1407-SerAS9_3071-YP_004506451.1--36H-COG1/1-541   QPL----NIMRRHFDRIAAGDL--ATPIQV--YGRNEISQLFASLQHMQRSLIGTVGAVR
Pr.stu.1965-S70_18140-YP_006218125.1--36H-COG1/1-521     YPL----MSMSDHFMKLAKGKL--NQDIRV--TTRDEIGEVFEQLKLMQESLATSVINLR
En.bac.2261-D782_1749-YP_007339930.1--36H-COG1/1-533     SPL----KIVSDHFDSIAKGDL--ARPIAV--YGKNEISAIFAGLKAMQDALRNTVSQVR
En.asb.1498-Entas_2590-YP_004829104.1--36H-COG1/1-533    QPL----AIISSHFDSIAKGNL--ARPVAV--YGKNEISAIFASLKAMQGSLRETVTDVR
En.asb.1498-Entas_3672-YP_004830170.1--36H-COG1/1-545    KPL----DRIRQQFRQIAQGDL--SHPIEP--FGRNCVGQLVPLLSAMQDSLREAVSTIR
Ci.kos.578-CKO_01067-YP_001452646.1--36H-COG1/1-537      QPL----AIIGSHFDSIADGNL--ARPIAV--YGRNEISAIFASLKAMQQALRETVSDVR
En.clo.1544-EcWSU1_02810-YP_004952663.1--36H-COG1/1-538  QPL----AIVTSHFDSIAKGNL--ARPVAV--YGKNEISAIFASLKAMQGALRETVSDVR
Sa.ent.407-STM3152-NP_462067.1--36H-COG1/1-547           KPL----ERIRQQFQRIAQGDL--SQPIEA--LGRNCVGRLVPLLRAMQDSLREAVSTIR
Pe.atr.485-ECA1332-YP_049438.1--36H-COG1/1-555           RPL----NDLRYHFSVIASGHL--GKPILD--FGNNCVGRLFPLLREMQGSLATTVSTIR
Pe.car.1139-PC1_1208-YP_003016792.1--36H-COG1/1-555      RPL----NDLRYHFSVIAAGNL--GKPILD--FGNNCVGRLFPLLREMQGSLASTVSTIR
En.638.865-Ent638_3407-YP_001178118.1--36H-COG1/1-545    KPL----GRIREHFRQIAQGDL--SQSVEP--FGRNCVGQLVPLLTAMQDSLREAVSSIR
Pa.vag.184-Pvag_1413-YP_003931052.1--36H-COG1/1-554      RPL----ESIRTHFAVIATGDL--SQPVAD--FGRNCVGKLVPLLSAMQDSLREAVSAIR
Ph.asy.1114-PAU_02684-YP_003041518.1--36H-COG1/1-544     RPF----ANMRSHFQHVAEGKL--NKEVAV--FTNDEIGEVFLKLRDMQHSLVNSITLIK
Pa.ana.1905-PAJ_1316-YP_005934192.1--36H-COG1/1-556      KPL----EKIREHFSVIAKGDL--SKPVED--MGRNCVGKLVPLLSAMQDSLREAVSAIR
Ci.kos.578-CKO_04394-YP_001455885.1--36H-COG1/1-561      KPL----ARIRQQFRQIAQGDL--SQPIEE--FGRNCVGRLVPLLSAMQDSLREAVSSIR
Sa.bon.1474-SBG_2750-YP_004731563.1--36H-COG1/1-547      RPL----DRIRQQFHQIAQGDL--SQPIEA--LGRNCVGRLVPLLRAMQDSLREAVSTIR
Ra.aqu.1678-Q7S_16025-YP_005402985.1--36H-COG1/1-554     KPL----DKIRGHFAVIASGDL--TFYLEE--FGKNCAGKLIPLLNEMQLSLVNTVQTIR
Rahnel.1320-Rahaq_3177-YP_004213898.1--36H-COG1/1-554    KPL----DKIRGHFAVIASGDL--TFYLEE--FGKNCAGKLIPLLNEMQLSLVNTVQTIR
Pectob.2320-W5S_3114-YP_006284066.1--36H-COG1/1-555      RPL----NDLRYHFGVIASGHL--GKPILD--FGNNCVGRLFPLLREMQGSLASTVSTIR
Ye.ent.378-YE2971-YP_001007160.1--36H-COG1/1-552         RPL----DDIRAHFRVIASGQL--GQPITD--FGRNCVGKLFPLLRDVQASLANTVKAIR
Er.bil.197-EbC_20190-YP_003741400.1--36H-COG1/1-556      RPL----DGMREHFRLIADGDL--SHPLED--IGRNCVGKLVPLLRAMQDSLREAVSAIR
Ph.lum.1262-plu1854-NP_929127.1--36H-COG1/1-544          RPF----ANMRSHFQNVAEGKL--NKEVSV--FTQDEIGEVFLKLREMQRSLVNSITLVK
Er.bil.197-EbC_25340-YP_003741912.1--36H-COG1/1-528      APL----SRMRGHFERIASGNL--GGTIET--EGRNELSQLFISLNAMQKSLAETVSSVR
Di.zea.1140-Dd1591_2937-YP_003005238.1--36H-COG1/1-564   RPL----NDLRDHFLVIASGRL--GKPILD--FGNNCVGKLFPLLRDMQTSLANTVSTIR
Er.tas.1011-ETA_14670-YP_001907406.1--36H-COG1/1-518     APL----ARMRSHFERISHGDL--NGQIEK--WGSNELGQLFTSLQHMQQALASTVRDVR
Sa.ent.404-STY4234-NP_458344.1--36H-COG1/1-547           RPL----QRIMAHIQTIADGDL--THEIEA--EGRSEMGQLAAGLKTMQQSLIRTVSAVR
Sa.ent.407-STM3577-NP_462478.1--36H-COG1/1-547           RPL----QRIMAHIQTIADGDL--THEIEA--EGRSEMGQLAAGLKTMQQSLIRTVSAVR
Sa.bon.1474-SBG_3170-YP_004731982.1--36H-COG1/1-547      RPL----QRIMTHIQTIADGDL--TRVIEA--EGRSEMGQLAAGLKTMQQSLIRTVSAVR
En.asb.1498-Entas_2591-YP_004829105.1--36H-COG1/1-555    NPL----GRVIAHIREIASGDL--TKTLTV--SGRNEITELANSVDHMQRSLIDTVTNVR
En.638.865-Ent638_1858-YP_001176586.1--36H-COG1/1-552    RPL----RSVMNHIRTIASGDL--THTISI--DSRNEMGQLAAGLHEMQQSLVTTVSAVR
Ci.rod.62-ROD_19331-YP_003365490.1--36H-COG1/1-553       NPL----GRVIVHIREIASGNL--TNTLTV--AGRNEIGELASTVSHMQRSLVATVAEVR
Di.dad.235-Dda3937_01559-YP_003882087.1--36H-COG1/1-566  RPL----NDLRDHFQVIANGRL--GKPILD--FGNNCVGRLFPLLRDMQGSLANTVSTIR
Er.bil.197-EbC_37780-YP_003743156.1--36H-COG1/1-553      TPL----KQVLAHIQRIAAGDL--SETLVV--EGRSEMGMLASNLTAMQQSLIVTVGHVR
Pantoe.297-Pat9b_2001-YP_004115872.1--36H-COG1/1-558     KPL----DRIRDHFAIIAQGDL--SQPVQE--FGRNCVGKLVPLLRAMQDSLREAVSAIR
```

```
En.clo.1544-EcWSU1_01962-YP_004951821.1--36H-COG1/1-553  KPL----HMIMNHIRAIAGGDL--TQDIAL--SGRNEMGQLAAGLHEMQQSLVTTVSAVR
En.asb.1498-Entas_1879-YP_004828403.1--36H-COG1/1-554    KPL----KIIMNHIRTIAGGDL--THNITL--GSRNEMGQLAVGLHEMQQSLVSTVSAVR
En.bac.2261-D782_1748-YP_007339929.1--36H-COG1/1-547     TPL----NHVMTHIRHIANGDL--TPALSL--KGRNELTQLADTVNHMQHALIDTVTRVR
Es.col.1836-Y75_p1862-YP_490148.1--36H-COG1/1-553        TPL----AKIIAHIREIAGGNL--ANTLTI--DGRSEMGDLAQSVSHMQRSLTDTVTHVR
Cr.tur.6-CTU_33010-YP_003211664.1--36H-COG1/1-555        GPL----REAMEHIRAIAGGDL--TRQIAV--EGRNEMGQLAASLQEMQQALAGTVTAVR
Cr.tur.6-CTU_25780-YP_003210941.1--36H-COG1/1-536        TPL----ASVIHHIREIAGGNL--TETITV--SGRNEITELSASVQHMQQALIQTVSSVR
En.clo.1544-EcWSU1_00403-YP_004950264.1--36H-COG1/1-549  LPL----QDVIGHIRAIAAGDL--TQPIQA--EGKNEMAILARNVQEMQTSLANTVGVVR
En.638.865-Ent638_2457-YP_001177177.1--36H-COG1/1-555    NPL----GNVIGHIREIAGGDL--TKKLTI--SGRNEITELASSVDHMQRSLTDTVANVR
Sa.bon.1474-SBG_1755-YP_004730609.1--36H-COG1/1-553      TPL----GRVIAHIREIASGDL--TKTLTV--SGRNEIGELAGTVDHMQRSLIETVTQVR
Pr.mir.1265-PMI1665-YP_002151396.1--36H-COG1/1-548       KPF----ASLSRYFNDVATGKL--NREILV--FTDDEIGDIFRRLREMRGELARSIRIVR
Cr.sak.579-ESA_01348-YP_001437444.1--36H-COG1/1-556      KPL----AGVIHHIREIAGGNL--TETITV--TGRNEMTELSASVQHMQQALIQTVSTVR
Cr.sak.579-ESA_00560-YP_001436686.1--36H-COG1/1-555      GPL----REAMEHIRAIAGGDL--TRQIAV--EGRNEMGQLAASLLAMQQALAGTVTAVR
Sa.ent.407-STM1919-NP_460876.1--36H-COG1/1-553           NPL----ARVITHIREIASGDL--TKTLTV--SGRNEIGELAGTVEHMQRSLIDTVTQVR
Ci.kos.578-CKO_01066-YP_001452645.1--36H-COG1/1-552      NPL----ARVIAHIRDIASGNL--TNTLTV--SGRNEIGELAGTVDHMQRSLIETVTQVR
En.asb.1498-Entas_0424-YP_004826963.1--36H-COG1/1-549    LPL----QDVIGHIRAIAAGDL--TQPVEA--SGKNEMALLAHNVQEMQKALANTVGVVR
Pantoe.297-Pat9b_1273-YP_004115150.1--36H-COG1/1-553     IPM----KQLQKQMERFAEGDL--SGSLHV--EGRSEMAALAASLNHMQQALLSTVNNVR
Ed.tar.1771-ETAF_2264-YP_005699859.1--36H-COG1/1-555     ARI----QQVIAYQAEIAHGNL--AIDIQA--RGDNEVSQLMQGLRHMRDELAHMVGAVR
Pa.ana.1905-PAJ_3534-YP_005936409.1--36H-COG1/1-553      KPL----SMATDYMRAISAGDL--TQEIAS--ESQNETGRLLRQLEEMRSALVVTIRSVD
Pa.vag.184-Pvag_1203-YP_003930842.1--36H-COG1/1-552      NPL----ARLTAHMRAISAGDL--TSAIQH--EGRNEMGQLIQELQQMRDALVVTITSVD
Pantoe.297-Pat9b_1605-YP_004115478.1--36H-COG1/1-556     QPL----QQLLAHIRHIAAGDL--TQTIQV--EGRNEMSQLAEGLLEMQQELVRTVSNVR
Cr.sak.579-ESA_00210-YP_001436348.1--36H-COG1/1-557      LPL----HDVMGHIRAIAAGDL--TQPIDA--QGHNEMALLARNVHEMQQALARTVSTVR
Er.tas.1011-ETA_pET460340-YP_001905943.1--36H-COG1/1-543 RPL----NNLREHFRLITEGDL--THTLRD--LSRNCVGRLVPMVREMQDSLREAVSSIR
Cr.tur.6-CTU_36610-YP_003212024.1--36H-COG1/1-557        LPL----HEVISHIRAIAAGDL--TQPIDA--QGHNEMALLARNVHEMQQSLARTVSTVR
Pe.car.1139-PC1_1159-YP_003016743.1--36H-COG1/1-556      HPL----RTLMQHISAIAEGNL--THPIDT--TGRNEMSQLAAGLHHMQQSLIRTVSLVR
Er.tas.1011-ETA_14640-YP_001907403.1--36H-COG1/1-554     RPL----QATIGHISRIAEGDL--TQNVEV--KGRNEMAQLAGSVKNMQEALVKTVSVVR
Pa.vag.184-Pvag_1725-YP_003931362.1--36H-COG1/1-556      TPL----NGSINHIQHIAQGDL--TQPIAV--EGRNEMTQLAASLHEMQQSLVRTVTNVR
Ed.ict.1187-NT01EI_3742-YP_002935104.1--36H-COG1/1-525   RPL----AQLHAHLRRLTAGDL--SATL-A--GGRNELGSLAHSISEMQQALISIVGQVR
En.638.865-Ent638_0380-YP_001175119.1--36H-COG1/1-549    KPL----DEVISHIRAIAAGDL--TRPIDA--HGKNEMAVLGRNVQEMQTALANTVGVVR
Er.bil.197-EbC_25360-YP_003741914.1--36H-COG1/1-556      TPL----QNNIAHISHIAGGDL--THTIEV--EGRNEMAQLANSLLEMQQSLVRTVSDVR
Er.tas.1011-ETA_16910-YP_001907630.1--36H-COG1/1-543     RPL----NNLREHFRLITEGDL--THPLHD--LNRNCVGRLVPLVREMQDSLREAVSSIR
Es.fer.1173-EFER_3055-YP_002384154.1--36H-COG1/1-603     EPM----NRLVDSIRHIAGGDL--VKPIDV--DGDNEVGQLADSLRHMQSELVRTVGDVR
Xe.nem.162-XNC1_1619-YP_003711880.1--36H-COG1/1-520      KPF----ENMRTCFQDVAEGRL--DKEVAV--TTDDEIGDIFRKLRDMQGSLIKSISAVR
En.clo.1544-EcWSU1_02811-YP_004952664.1--36H-COG1/1-559  NPL----GRVIAHIREIAGGDL--TKTLTV--SGRNEITELANSVDHMQRSLIETVANVR
Di.dad.235-Dda3937_03500-YP_003882750.1--36H-COG1/1-553  KPI----GQIKRHLELLTSGKL--GVELDE--FGRNCAGQLIPYIRAMQHSLRNTVQTIH
Xe.bov.105-XBJ1_1924-YP_003467828.1--36H-COG1/1-523      RPF----DNMRACFQDVAEGRL--NKEIAV--TTSDEIGDIFRKLRDMQQSLIKSIASVR
Rahnel.1320-Rahaq_1831-YP_004212576.1--36H-COG1/1-556    TPL----TIMRGHFDRIGAGDI--SSPIVA--EGRYEIKVMLESLQQMQTSLADTVRTVR
Ra.aqu.1678-Q7S_08965-YP_005401598.1--36H-COG1/1-556     TPL----TIMRGHFDRIGAGDI--SSPIVA--EGRYEIKVMLESLQQMQTSLADTVRTVR
Cr.sak.579-ESA_01126-YP_001437230.1--36H-COG1/1-514      KPL----EKIRNHFTLIAGGDL--SQPVEP--FGRNCVGKLVPLLTAMQDQLREAVSAIR
Pe.atr.485-ECA2712-YP_050803.1--36H-COG1/1-560           KPL----NMIKGYFQVLAAGQL--GHPLDE--FGRNCAGQLIPYLKEMQGSLVNTVSIIR
En.clo.1544-EcWSU1_00560-YP_004950421.1--36H-COG1/1-597  SPL----TRLIDNIRHIASGDL--VKRIEV--QGANEMGELADSLRHMQGELVRTVGDVR
Di.dad.235-Dda3937_03498-YP_003882752.1--36H-COG1/1-561  NPI----AVIKAHLGKLIAGRL--DQHLAE--FGRNCAGRIIPDIKQLQHSLKDTVALIS
Cr.tur.6-CTU_27880-YP_003211151.1--36H-COG1/1-514        KPL----EKIRNHFTLIAGGDL--SQPVEP--FGRNCVGKLVPLLTAMQDQLREAVSAIR
Pe.car.1139-PC1_1669-YP_003017246.1--36H-COG1/1-560      KPL----NMIKGYFQVLAAGQL--NHPIDE--FGRNCAGQLIPYLKEMQGSLVNTVSIIR
Ra.aqu.1678-Q7S_14450-YP_005402672.1--36H-COG1/1-551     KPL----NKVKAHLESLATGIL--DSKIAF--QGKNCVGQLIPYINKMQDNWAKTVYDIR
Ci.kos.578-CKO_03622-YP_001455137.1--36H-COG1/1-517      KPL----HDVMEHIRHIAAGDL--TRNIHT--EGSNEMALLAHNVHEMQQALANTVSVVR
Di.zea.1140-Dd1591_2408-YP_003004728.1--36H-COG1/1-557   KPI----GQIKRHLELLTSGKL--GVELEE--FGRNCAGQLIPYIRAMQHSLRNTVQTIH
Di.zea.1140-Dd1591_2406-YP_003004726.1--36H-COG1/1-559   SPI----SAIQAHLGKLIAGRL--DQHLDE--FGRNCAGRMIPDIKKLQLSLKDTVSLIR
Ed.ict.1187-NT01EI_2801-YP_002934203.1--36H-COG1/1-554   ARI----QKVIAYQAEIAHGNL--AIDIQT--HGDNEISQLMQGLRHMRDELAHMVGSVR
Pe.car.1139-PC1_1384-YP_003016966.1--36H-COG1/1-559      IPI----SVLKSHLAQLTAGRL--GCELAE--FGRNCAGRLIPDIKRLQKSLRDTVTVIR
Rahnel.1320-Rahaq_2866-YP_004213595.1--36H-COG1/1-551    KPL----NKVKAHLESLATGIL--DSKIAF--QGKNCVGQLIPYINKMQDNWAKTVYDIR
Pe.atr.485-ECA1281-YP_049387.1--36H-COG1/1-556           HPL----RTLMRHISAIAEGNL--THHIDT--AGRNEMSQLAAGLHDMQQSLIRTVSLVR
Pectob.2320-W5S_1886-YP_006282848.1--36H-COG1/1-549      KPL----NMIKGYFQVLAAGQL--GHPLDE--FGRNCAGQLIPYLKEMQGSLVNTVSIIR
Pectob.2320-W5S_1696-YP_006282659.1--36H-COG1/1-559      IPI----SVLKSHLAQLTAGRL--GCELVE--FGRNCAGRLIPDIKALQKSLRDTVTVIR
Di.zea.1140-Dd1591_2407-YP_003004727.1--36H-COG1/1-555   KPI----GQIKRHLELLTSGKL--GVELEE--FGRNCAGQLIPYIRAMQQSLRDTVQTIH
Pe.atr.485-ECA1509-YP_049611.1--36H-COG1/1-559           IPI----AVLKSHLSQLTAGRL--GCELAE--FGKNCAGRLIPDIKRLQKSLRDTVTVIR
Pa.vag.184-Pvag_1724-YP_003931361.1--36H-COG1/1-520      QPL----QQLLAQIRAIASGDL--TQPIVV--EGRNEMSQLAAGIQEMQQSLVMTVGNVR
Pa.ana.1905-PAJ_1541-YP_005934417.1--36H-COG1/1-520      QPL----QRLLVDIRTIASGDL--TQPILV--EGRNEMSQLAAGIHEMQHSLVSTVGEVR
Mo.mor.2189-MU9_1596-YP_007505015.1--36H-COG1/1-519      RPL----NNVRQHFRRIAGGDL--SQPVESDAAGRNCVGKLFPLLSEMQDSLRDAVSSIR
Pr.mir.1265-PMI2808-YP_002152509.1--36H-COG1/1-563       VRI----NQMIEYQEAIASGDL--ISRIDHDITGRNEIDQLMLGLQQMRARLKEMVSAIR
Di.zea.1140-Dd1591_0395-YP_003002764.1--36H-COG1/1-556   RPL----EQVKAHLQILSNGQL--DTSVDE--MGRNEVGQLIPFVHTMQNNWIKTVAEIR
En.clo.1544-EcWSU1_03786-YP_004953635.1--36H-COG1/1-515  KPL----ARIRQQFRQIAQGDL--SQPIEP--FGRNCVGQLVPLLSAMQDSLREAVSSIR
Pectob.2320-W5S_3170-YP_006284116.1--36H-COG1/1-556      HPL----RTLMRHISAIAEGNL--TLHIDA--SGRNEMSQLAAGLHHMQQSLTRTVSLVR
Di.dad.235-Dda3937_02184-YP_003884754.1--36H-COG1/1-556  RPL----EKVKAHLQILSNGQL--DSTIGE--MGRNEIGQLIPFVNAMQQNWIKTVADIR
Ci.rod.62-ROD_32641-YP_003366748.1--36H-COG1/1-517       KPL----HEVMDHIRHIASGDL--THVIQA--DGSNEMALLARNLHEMQQSLANTVSVVR
Di.dad.235-Dda3937_03499-YP_003882751.1--36H-COG1/1-561  KPI----GQIKRHLELLTSGKL--GVELDE--FGRNCAGQLIPYIRAMQHSLSDTVQTIH
Di.dad.235-Dda3937_00105-YP_003883346.1--36H-COG1/1-561  QPL----NRIITHLHHISTGDL--TQSLHI--QAKNEIGQLADSVRHMQHSLTDTVDKVR
Pantoe.297-Pat9b_1604-YP_004115477.1--36H-COG1/1-555     NPL----NSNIRHIQHIAQGDL--TQTIAI--EGRNEMTQLATNLHEMQQSLVRTVSNVR
Er.tas.1011-ETA_14660-YP_001907405.1--36H-COG1/1-560     RPL----KQNIEHIRGIARGDL--TQAIEA--QGHNEMSQLAASLQHMQASLVTTVSAVR
Pr.mir.1265-PMI2809-YP_002152510.1--36H-COG1/1-575       LRI----KQVIDYMSDISQGNLLENSTIKA--KGNNEIDQLINGIQYMRSELSLIVNAIR
Ed.tar.1771-ETAF_3053-YP_005700648.1--36H-COG1/1-516     RPL----AQLHAHLRRLTAGDL--SATL-A--GGRNELGSLARSISEMQQALISIVGQVR
Se.pro.864-Spro_1415-YP_001477647.1--36H-COG1/1-546      NKI----NQIIDYQAEISNRNL--DIEINA--GGNNEIGRLINGLINMRNELANTVCTVR
Di.zea.1140-Dd1591_1804-YP_003004134.1--36H-COG1/1-562   QPL----NQVMVHLHHIATGDL--TQSLTI--EAKNEIGQLAERVRHMQHALIDTVGKVR
Di.dad.235-Dda3937_03501-YP_003882749.1--36H-COG1/1-575  KPI----GQIKRHLELLTSGKL--GVELDE--FGRNCAGQLIPYIRAMQHSLRNTVQTIH
Pantoe.297-Pat9b_0851-YP_004114731.1--36H-COG2/1-524     RPL----NEAVEIAGKVAAGDL--STHIEV--KSTDETGVLMRALQSMNDNLLTIVTDVR
Pa.vag.184-Pvag_0291-YP_003929953.1--36H-COG2/1-525      RPL----VAAVDVAERVAAGDL--STQMQV--TSRDETGVLMQALQRMNDNLLSIVTEVR
En.clo.1544-EcWSU1_03886-YP_004953735.1--36H-COG2/1-519  RPL----GEAVDFAKAIAEGDL--TGSITP--HGKDETGLLLHALMEMKTRLLDIVQQVQ
Cr.tur.6-CTU_04790-YP_003208842.1--36H-COG2/1-515        RPL----DQAVRFAGAIAEGDL--TRSVEV--QARDETGVLLHALMDMKQRLQAIVQEVQ
Ci.kos.578-CKO_04484-YP_001455975.1--36H-COG2/1-521      RPL----NDAVQFAGAIAEGDL--TRTIHI--AQKDETGVLLQALMEMKNRLLVIVQEVQ
Cr.sak.579-ESA_03488-YP_001439539.1--36H-COG2/1-515      RPL----DQAVRFAGAIAEGDL--TRSVDV--QSRDETGVLLNALMDMKQRLQAIVQEVQ
Ci.rod.62-ROD_48071-YP_003368199.1--36H-COG2/1-521       RPL----EEAVQFAKAIAGGDL--TRSIAT--DQRDETGVLLQALMAMKTRLLEIVQQVQ
Sa.bon.1474-SBG_2851-YP_004731664.1--36H-COG2/1-521      QPL----DEAVRFAEAIADGDL--TRHITT--NDKDETGVLLQALMAMKTRLLEIVQEVQ
Sa.ent.407-STM3216-NP_462130.1--36H-COG2/1-521           RPL----DEAVRFAEAIADGDL--TRHITT--DYKDETGVLLQALMAMKTRLLDIVQEVQ
En.asb.1498-Entas_3753-YP_004830248.1--36H-COG2/1-519    RPL----GTAVAFAEAIAQGDL--TRTIAS--HGKDETGHLLNALMEMKTRLLEIVQQVQ
Mo.mor.2189-MU9_3389-YP_007506806.1--36H-COG2/1-521      RPL----DKAVTLARAIAQGDL--TQQVNV--THHDETGVLLNALAEMQVHLQDIVTEVK
Sa.ent.404-STY3394-NP_457606.1--36H-COG2/1-522           RPL----DDAVRFAEAIADGDL--TRHITT--DYKDETGVLLQALMAMKTRLLDIVQEVQ
En.638.865-Ent638_3526-YP_001178235.1--36H-COG2/1-519    RPL----GEAVSFAEAIAEGDL--TRNITT--RSKDETGLLLHALMEMKTRLLEIVQQVQ
Pe.atr.485-ECA1774-YP_049874.1--36H-COG2/1-554           QPI----REALGVAEKVAKGDL--TSEIYT--DRTDETGQLLSALNNMNGSLRQIVSQVR
En.638.865-Ent638_2119-YP_001176845.1--36H-COG2/1-549    RPL----KEAVTVASRAAQGDL--TTDVKV--HSTDELGQLMSALKEMVSELGSTVLQVR
```

```
Pectob.2320-W5S_2813-YP_006283770.1--36H-COG2/1-554      QPL----QEALVIAENVAKGDL--TSEIYT--DRKDETGQLLSALNNMNGSLRQIVSQVR
Di.zea.1140-Dd1591_0695-YP_003003055.1--36H-COG2/1-561   QPI----QQALVMADRVSQGDL--SSHISS--HRKDEAGRLLQALDHMNASLRQIVGQVR
Pa.vag.184-Pvag_pPag30079-YP_003729820.1--36H-COG2/1-546 RPL----NITLAAAQRIARGDL--SQAVPV--SGRDETGMLLTAVAEMQDSLAQTVSTVR
Er.bil.197-EbC_38940-YP_003743272.1--36H-COG2/1-526      RPL----REAVTIAENVAAGDL--RTVIRV--NSTDETGQLMQALKHMNENLLRIVTEVR
Ra.aqu.1678-Q7S_24891-YP_005419232.1--36H-COG2/1-554     RPL----HTAVKVAENVAAGDL--TTQIQV--MSQDETGQLMQGLKNMNENLLKIVTEVR
En.asb.1498-Entas_2287-YP_004828805.1--36H-COG2/1-513    RPV----KHNLLLAERIASGDL--SSDIRA--QGNDELGKLTGAMGRMNDTLRSMIGDVR
Rahnel.1320-Rahaq_4869-YP_004215574.1--36H-COG2/1-554    RPL----HTAVKVAENVAAGDL--TTQIQV--MSQDETGQLMQGLKNMNENLLKIVTEVR
Di.dad.235-Dda3937_00027-YP_003883574.1--36H-COG2/1-554  RPL----QEALTLAEKVAKGDL--TTDASA--CAQDETGKLLQALYGMNDSLRSIVSQVR
Pr.stu.1965-S70_05790-YP_006215722.1--36H-COG2/1-520     RPL----GAAVGFAQAIAEGDL--TQHVEI--KNQDETGVLLRALVEMKKRLLEIVQEVQ
En.638.865-Ent638_2100-YP_001176826.1--36H-COG2/1-512    HPV----RHNLQLAERIASGDL--SSDIHP--QGDDELGKLTGAMGRMNDKLRAMIGEVR
Pe.car.1139-PC1_2526-YP_003018093.1--36H-COG2/1-554      QPL----QEALGVAENVAKGDL--TSEIYT--GRKDETGQLLSALNNMNSSLRQIVSQVR
Mo.mor.2189-MU9_702-YP_007504121.1--36H-COG2/1-525       RSLGAEPAQLKEVAERVASGDL--SPSDEI--HPVPD-GSVLSTLKAMQRNLASIVGTVR
Pe.car.1139-PC1_3443-YP_003018995.1--36H-COG2/1-511      RPL----HQALHATRAIAEGDL--TVQLHN--ERRDELGQLISAMGQMNNNLHSMIDKIR
Pa.ana.1905-PAJ_0915-YP_005933791.1--36H-COG2/1-559      RPL----NVAVEAARRVARGDL--TGTVTA--DRKDETGILLSAISDMQASLINTVSLVR
Pectob.2320-W5S_3736-YP_006284671.1--36H-COG2/1-510      RPL----HQALHATRAIAEGDL--TVQLHN--ERRDELGQLISAMGQMNNNLHSMIDKIR
Pe.atr.485-ECA0183-YP_048310.1--36H-COG2/1-553           RPI----QQALQVADRVAQGDL--TSRITI--TSKDETGLLLQSLDHMNTSLSSIVGQVR
Rahnel.1320-Rahaq_3107-YP_004213828.1--36H-COG2/1-503    LPL----RRTLQTAERIAAGDL--STHATS--HSFDEVGQLSRAIETMNQNLHGIIGDIR
Pe.car.1139-PC1_4071-YP_003019622.1--36H-COG2/1-553      QPI----QQALQVADRVAQGDL--TSRITV--TSKDETGLLLQSLDHMNTSLSTIVGQVR
Di.dad.235-Dda3937_03462-YP_003884507.1--36H-COG2/1-563  QPI----QQALVMADRVSQGDL--TSQISS--NRKDEAGLLLQALDHMNASLRQIVGQVR
Ra.aqu.1678-Q7S_15665-YP_005402913.1--36H-COG2/1-503     LPL----RRTLQTAERIAAGDL--STHATS--HSFDEVGQLSRAIETMNQNLHGIIGDIR
Pectob.2320-W5S_4505-YP_006285423.1--36H-COG2/1-555      RPI----QQALQVADRVAQGDL--TSRITV--TSKDETGLLLQSLDHMNTSLSTIVGQVR
Pe.atr.485-ECA3642-YP_051730.1--36H-COG2/1-551           QPL----ARAVSATQAVAAGDL--THNIQP--EGRDEAAQLLHALQDMTVRLRSIVGEVR
Pe.car.1139-PC1_1417-YP_003016999.1--36H-COG2/1-554      TPL----SIALSSAKRISEGDL--SGSIVV--NNRDETGLLLEAMQEMQAALTRMVVGVR
En.asb.1498-Entas_4509-YP_004821569.1--36H-COG2/1-559    KPV----QEALSAIEASGRGDM--RTTFRKD-YHSDETGQLLSGLKDTVVNISFMLNQVR
Pa.ana.1905-PAJ_3064-YP_005935940.1--36H-COG2/1-544      RPL----NQALIAAQRVAKGDL--SEPVQV--THTDETGKLLVAIREMQEALVQTVSLVR
Pectob.2320-W5S_1733-YP_006282696.1--36H-COG2/1-535      GPL----SIALSSAKRISVGDL--SGSIVV--NNRDETGLLLEAMQEMQAALTRMVVGVR
Pe.atr.485-ECA4334-YP_052421.1--36H-COG2/1-556           RQLGGEPAYTLEVTRQVAQGNL--AFTIEL--RDGDTTS-VLAAMEDMRQNLSNLVGQVH
Di.zea.1140-Dd1591_1602-YP_003003935.1--36H-COG2/1-554   QPL----QAALTLAGKVAKGDL--TTEASA--CTRDETGQLLQALYGMNESLRNIVSQVR
Pectob.2320-W5S_4506-YP_006285424.1--36H-COG2/1-540      RPI----EQALHVADRVAQGDL--TSHITV--ASKDETGLLLQSLDRMNTSLSSIVGQVR
Di.zea.1140-Dd1591_1453-YP_003003794.1--36H-COG2/1-532   RSL----SQAQAAAAAVANGDL--TYPINA--SGRDEITTVLNTLSHMQESLGSMVTQVR
Pectob.2320-W5S_0106-YP_006281117.1--36H-COG2/1-556      RQIGGEPAYTLEVTRQVAQGNL--AVTIEL--RDGDTTS-VLASIEDMRQNLSNLVGQVH
Di.zea.1140-Dd1591_0769-YP_003003127.1--36H-COG2/1-505   RPL----HEALQATRAISEGDL--TVRLRS--ERRDELGQLICAMAKMNDNLHGMIDKIR
Ra.aqu.1678-Q7S_18035-YP_005403387.1--36H-COG2/1-535     RALGEEPLVLGQVVQRIAAGDL--SEVKGA--ENAPQ-NSVLAELGGMQAKLHQLINQVA
Rahnel.1320-Rahaq_3579-YP_004214298.1--36H-COG2/1-535    RALGEEPLVLGQVVQRIAAGDL--SEVKGA--ENAPQ-NSVLAELGGMQAKLHQLINQVA
Pe.car.1139-PC1_4072-YP_003019623.1--36H-COG2/1-540      RPI----AQALQVADRVAQGDL--TSHITV--TSKDETGLLLQSLDRMNTSLSSIVGQVR
Pe.car.1139-PC1_3464-YP_003019016.1--36H-COG2/1-551      QPL----ARAVRATQAVAAGDL--THNVQP--EGRDEAAQLLHALQDMTVRLRTIVGEVR
Pe.car.1139-PC1_0111-YP_003015709.1--36H-COG2/1-556      RQLGGEPAYTLEVTRQVAQGNL--AVAIEL--RDGDTTS-VLAAMEDMRQNLSNLVGQVH
Pe.atr.485-ECA0182-YP_048309.1--36H-COG2/1-542           RPI----EQALQVADRVAQGDL--ISRITV--TSKDETGLLLQSLDRMNTSLSLIVGQVR
Pectob.2320-W5S_3753-YP_006284688.1--36H-COG2/1-551      QPL----SRAVQATQAVAAGDL--THNIQP--EGRDEAAQLLHALQDMTVRLRSIVGEVR
Pa.ana.1905-PAJ_2666-YP_005935542.1--36H-COG2/1-514      RQLGGEPAQAQALSAAIASGDL--TAPVIL---RHNDSSSLLASLANMQSSLRSLVTQIK
Ci.rod.62-ROD_16161-YP_003365194.1--36H-COG6/1-557       RPL----QHAALRIEQIAAGDL--TMPDEP--TGRSEIGRLSRHLQQMQHSLVKTVGSVR
En.asb.1498-Entas_2095-YP_004828614.1--36H-COG6/1-563    NPL----QRAAKRIENIAKGDL--TMPDDA--TGRSEIGRLTRDLQTMQHSLVTTVGTVR
En.638.865-Ent638_1961-YP_001176688.1--36H-COG6/1-563    NPL----QRAARRIELIAKGDL--TMADDL--TGRSEIGRLTHDLQTMQHSLVNTVGTVR
Cr.sak.579-ESA_01710-YP_001437800.1--36H-COG6/1-565      VPL----QHAATRIETIASGDL--TLPEEA--TGRSEIGRLSQHLQMMQRSLVKTVSAVR
Cr.tur.6-CTU_22440-YP_003210607.1--36H-COG6/1-565        VPL----QHAATRIETIASGDL--TLPEEA--TGRSEIGRLSQHLQMMQRSLVKTVSAVR
Ci.kos.578-CKO_01456-YP_001453025.1--36H-COG6/1-562      NPL----QRAAQRIEQIAAGDL--TMPDDP--TGRSEIGRLSRHLQKMQSSLGQTVGTVR
Rahnel.1320-Rahaq_1930-YP_004212674.1--36H-COG6/1-566    HPL----NRMVMRIQKIAEGDL--TQPTRA--HGRNEIGVLSNNVDQMQRSLASTVLTVR
Pantoe.297-Pat9b_2403-YP_004116261.1--36H-COG6/1-564     NPL----RQSVARIERIAQGDL--TAPEQA--WGRSEIGSLLHNLQLMQASLVRTVGVVR
Er.bil.197-EbC_26700-YP_003742048.1--36H-COG6/1-567      TPL----RQAVERIEHISRGDL--TAPLQN--WGRSEIGTLGSNLQNMQQSLVKTVGTVR
Pectob.2320-W5S_1760-YP_006282723.1--36H-COG6/1-570      KPM----NQLVMRIQRIAQGDL--TQVSDR--YGKNEIGTLASNVHQMQSSLVTTVTTVR
Pa.ana.1905-PAJ_1621-YP_005934497.1--36H-COG6/1-568      NPL----RNSVAHIERIAQGDL--TAPTVP--HGRNEIGLLLDNLQRMQQSLIKTVGTVR
En.clo.1544-EcWSU1_02120-YP_004951978.1--36H-COG6/1-576  NPL----QRAATRIENIAKGDL--TMPDEV--AGRSEIGRLTRDLQTMQHSLETTVGTVR
Ra.aqu.1678-Q7S_09815-YP_005401764.1--36H-COG6/1-566     HPL----NQMVMRIQKIAEGDL--TQPTRA--HGRNEIGVLSNNVDQMQRSLASTVLTVR
Ye.pse.585-YPTB2412-YP_070925.1--36H-COG6/1-579          NPI----NWLVTRIQRIAQGDL--TQSPVS--FGRNEIGVLGSNIQQMQDALAITVEAVR
Ye.ent.378-YE2588-YP_001006789.1--36H-COG6/1-579         KPI----NRLVARIQRIAQGDL--TQHPAP--FGRNEIGVLGNNIQQMQDSLSSTVEAVR
Di.dad.235-Dda3937_02787-YP_003883649.1--36H-COG6/1-572  KPM----NRLVQRIQRIAHGDL--TQPNEV--YGKNEIGILGQNIQQMQESLVHTVSTVR
Pa.vag.184-Pvag_1800-YP_003931435.1--36H-COG6/1-568      NPL----RQSVLRIERIAQGDL--TSAPLP--FGRSEIGTLIHNLQLMQQALVTTVGTVR
En.bac.2261-D782_2307-YP_007340470.1--36H-COG6/1-558     NPL----QHAGQRIAQIASGDL--TQPDES--VGRSEIGQLSFNLQKMQHALVQTVGTVR
Pe.atr.485-ECA1683-YP_049784.1--36H-COG6/1-573           KPI----NQLVMRIQRIAQGDL--TQMSDR--YGKNEIGTLASNVQQMQSSLVTTVTTVR
Pe.car.1139-PC1_2617-YP_003018183.1--36H-COG6/1-573      KPM----NQLVARIQRIAQGDL--TQMSDR--YGRNEIGTLASNVQQMQSSLVTTVTTVR
Di.zea.1140-Dd1591_1535-YP_003003868.1--36H-COG6/1-574   KPM----HRLVQRIQRIAQGDL--TQPNEA--YGKNEIGILGQNIQQMQASLVHTVSTVR
Di.zea.1140-Dd1591_3580-YP_003005868.1--36H-COG6/1-572   NPM----RALVERIEHIASGDL--TMPLAQ--WGRGEIGQLGHYLQNMQQSLARTVSNVR
Es.col.1836-Y75_p1397-YP_489687.1--36H-COG6/1-546        RPL----QHAAQRIEKIASGDL--TMNDEP--AGRNEIGRLSRHLQQMQHSLGMTVGTVR
Pe.atr.485-ECA3902-YP_051990.1--36H-COG6/1-575           API----NRLVERIQKIAAGDL--TLPPMA--MGRNEIGTLGTNIQNMQAELTNTVTIVR
Sa.bon.1474-SBG_1454-YP_004730321.1--36H-COG6/1-537      RPL----QQAALRIERIAAGDL--TMDDEP--AGRSEIGRLSRHLQQMQHSLQQTVGAVR
Sa.ent.407-STM1626-NP_460585.1--36H-COG6/1-541           QPL----QQSASRIERIAAGDL--TMADEP--TGRSEIGRLSHHLQQMQHALQQTVGAVR
Pe.car.1139-PC1_3679-YP_003019230.1--36H-COG6/1-567      API----NRLVERIQKIAAGDL--TQPPMA--MGRNEIGILGTNIQNMQAELTDTVTIVR
Pectob.2320-W5S_4020-YP_006284955.1--36H-COG6/1-575      API----NRLVERIQKIAAGDL--TQPPMV--MGRNEIGILGTNIQNMQAELTDTVTIVR
Di.zea.1140-Dd1591_0523-YP_003002884.1--36H-COG6/1-568   VPI----HVLIARIQRIAQGDL--TQPAED--LGRNEIGILGQNLHHMQASLTNTVTVVR
Di.dad.235-Dda3937_02665-YP_003881279.1--36H-COG6/1-572  KPM----RSLVERIEHIASGDL--TMPLAQ--WGRGEVGQLGQYLQNMQQSLARTVSNVR
```

```
Pr.mir.1265-PMI1666-YP_002151397.1--36H-COG1/1-568       QSTENIYNSTSEIAAGNNDLSSRTEEQVASLEETAASMEQLTATVKQNADNARQASNLAN
Di.dad.235-Dda3937_02779-YP_003883640.1--36H-COG1/1-566  QGAEAIYTGASEISAGNSDLSSRTEQQAAALEETAASMEQLTSTVKQNAENARQASQLAL
Di.zea.1140-Dd1591_1542-YP_003003875.1--36H-COG1/1-566   QSSEAIYTGASEISAGNSDLSSRTEQQAAALEETAASMEQLTSTVKQNAENARQASQLAL
Pe.atr.485-ECA1691-YP_049792.1--36H-COG1/1-561           QGADAIYTGATEISAGNNDLSSRTEQQAASLEETAASMEQLTATVKQNAENARQASQLAL
Se.pro.864-Spro_2983-YP_001479212.1--36H-COG1/1-556      QGADAIYSGASEIAAGNNDLSSRTEQQAASLEETAASMEQLTATVKQNAENARQASQLAL
Ph.asy.1114-PAU_02685-YP_003041519.1--36H-COG1/1-564     QSSESIYDGTSEIAAGNNDLSSRTEEQVASLEETAASMEQLTATVKQNAENARQASNLAD
Pr.stu.1965-S70_18135-YP_006218124.1--36H-COG1/1-561     DGSETIYQGTSEIAAGNNDLSARTQQQVACLEETAASMSELTATVKQNTEYAHQASEYAS
Mo.mor.2189-MU9_1762-YP_007505181.1--36H-COG1/1-561      DGSENIYQGTSEIAAGNNDLSARTQEQVACLEQTAASMEELTATVKQNTEYAHQASEFAG
Pe.car.1139-PC1_2609-YP_003018175.1--36H-COG1/1-561      QGADAIYTGATEISAGNNDLSSRTEQQAASLEETAASMEQLTATVKQNAENARQASQLAL
Xe.nem.162-XNC1_1620-YP_003711881.1--36H-COG1/1-567      QSTATIYTGTSEIAAGNNDLSARTEQQVASLEETAASMEQLTATVKQNADNARQASNLAD
Pectob.2320-W5S_1768-YP_006282731.1--36H-COG1/1-561      QGADAIYTGATEISAGNNDLSSRTEQQAASLEETAASMEQLTATVKQNAENARQASQLAL
Pa.vag.184-Pvag_1723-YP_003931360.1--36H-COG1/1-558      DGSDAIYTGASEISIGNNDLSSRTEQQAASLEQTAASMEQLTATVKQNAENARQASKLAL
Ye.pes.133-YPZ3_2024-YP_003568196.1--36H-COG1/1-557      VGADAIYSGASEIAVGNNDLSARTEQQAASLEETAASMEQLTATVKQNAENARQASQLAL
Ye.pse.585-YPTB2401-YP_070915.1--36H-COG1/1-557          VGADAIYSGASEIAAGNNDLSARTEQQAASLEETAASMEQLTATVKQNAENARQASQLAL
Ye.ent.378-YE2575-YP_001006778.1--36H-COG1/1-557         LGADAIYSGASEIAAGNNDLSARTEQQAASLEETAASMEQLTATVKQNAENARQASQLAL
Pa.ana.1905-PAJ_1540-YP_005934416.1--36H-COG1/1-563      DGADAIYTGASEIAVGNNDLSSRTEQQAASLEQTAASMEELTATVKQNAENARQASKLAL
Ph.lum.1262-plu1853-NP_929126.1--36H-COG1/1-564          QSSESIYNGTSEIAAGNNDLSSRTEEQVASLEETAASMEQLTATVRQNAENARQASNLAD
Serrat.1901-SerAS13_3075-YP_006025914.1--36H-COG1/1-555  QGADAIYSGASEIAAGNNDLSSRTEQQAASLEETAASMEQLTATVKQNAENARQASQLAL
Sa.ent.404-STY2128-NP_456485.1--36H-COG1/1-553           EGSDAIYSGTSEIAAGNTDLSSRTEQQASALEETAASMEQLTATVKQNADNARQASQLAQ
Ra.aqu.1678-Q7S_08960-YP_005401597.1--36H-COG1/1-564     HGADAIYSGASEISAGNSDLSSRTEQQASALEETAASMEQLTATVKQNAENARQASQLAL
Se.mar.2260-D781_2759-YP_007345195.1--36H-COG1/1-555     QGADAIYSGASEIAAGNNDLSSRTEQQAASLEETAASMEQLTATVKQNAENARQASQLAL
Rahnel.1320-Rahaq_1830-YP_004212575.1--36H-COG1/1-564    HGADAIYSGASEISAGNSDLSSRTEQQASALEETAASMEQLTATVKQNAENARQASQLAL
Se.ply.1407-SerAS9_3072-YP_004506452.1--36H-COG1/1-555   QGADAIYSGASEIAAGNNDLSSRTEQQAASLEETAASMEQLTATVKQNAENARQASQLAL
Ed.ict.1187-NT01EI_1461-YP_002932882.2--36H-COG1/1-555   QGANAIYSGASEISAGNHDLSSRTEQQAASLEETAASMEQLTATVKQNAENARQASQLAL
En.aer.1436-EAE_15535-YP_004593297.1--36H-COG1/1-556     QGADAIYSGASEIAAGNNDLSSRTEQQAASLEETAASMEQLTATVKQNAENARQASQLAL
Xe.bov.105-XBJ1_1923-YP_003467827.1--36H-COG1/1-567      QSTETIYTGTSEIAAGNNDLSSRTEQQVASLEETAASMEQLTATVKQNADNARQASNLAD
Serrat.1408-SerAS12_3073-YP_004501499.1--36H-COG1/1-555  QGADAIYSGASEIAAGNNDLSSRTEQQAASLEETAASMEQLTATVKQNAENARQASQLAL
Er.bil.197-EbC_25350-YP_003741913.1--36H-COG1/1-559      DGSDAIYTGASEISAGNNDLSSRTEQQAASLEQTAASMEQLTATVKQNAENARQASQLAL
Er.tas.1011-ETA_14650-YP_001907404.1--36H-COG1/1-554     NGSDAIYSGASEISSGNNDLSSRTEQQAASLEQTAASMEELTATVKQNAENARQASQLAL
Sa.ent.407-STM4533-NP_463392.1--36H-COG1/1-553           NGANAIYSGASEIAMGNNDLSSRTEQQAASLEETAASMEQLTATVKQNAENARQASHLAL
Es.col.1836-Y75_p4240-YP_492486.1--36H-COG1/1-551        NGANAIYSGASEIATGNNDLSSRTEQQAASLEETAASMEQLTATVKQNAENARQASHLAL
En.bac.2261-D782_3913-YP_007342002.1--36H-COG1/1-553     NGADAIYSGASEIAMGNNDLSSRTEQQAASLEETAASMEELTATVKQNAENARQASNLAL
Ci.kos.578-CKO_03442-YP_001454958.1--36H-COG1/1-554      NGANAIYSGASEIAMGNNDLSSRTEQQAASLEETAASMEELTATVKQNAENARQASHLAL
Cr.sak.579-ESA_03402-YP_001439456.1--36H-COG1/1-555      NGADAIYSGASEISAGNNDLSSRTEEQAASLEETAASMEELTATVKQNAENARQASQLAL
Pantoe.297-Pat9b_1606-YP_004115479.1--36H-COG1/1-558     DGSDAIYTGASEIAMGNNDLSSRTEQQAASLEETAASMEQLTATVKQNAENARQASQLAL
Cr.tur.6-CTU_05670-YP_003208930.1--36H-COG1/1-555        NGADAIYSGASEISAGNNDLSSRTEEQAASLEETAASMEELTATVKQNAENARQASQLAL
Sa.bon.1474-SBG_3944-YP_004732724.1--36H-COG1/1-553      NGANAIYSGASEIAMGNNDLSSRTEQQAASLEETAASMEQLTATVKQNAENARQASHLAL
En.asb.1498-Entas_0547-YP_004827084.1--36H-COG1/1-554    NGANAIYSGASEISMGNNDLSSRTEQQAASLEETAASMEQLTATVKQNAENARQASNLAL
Ci.rod.62-ROD_48461-YP_003368233.1--36H-COG1/1-554       NGANAIYSGASEIAMGNNDLSSRTEQQAASLEETAASMEQLTATVKQNAENARQASHLAL
Ed.tar.1771-ETAF_1249-YP_005698855.1--36H-COG1/1-529     QGANAIYSGASEISAGNNDLSSRTEQQAASLEETAASMEQLTATVKQNAENARQASQLAL
En.638.865-Ent638_0513-YP_001175251.1--36H-COG1/1-586    NGANAIYSGASEISVGNNDLSSRTEQQAASLEETAASMEQLTATVKQNAENARQASNLAL
Ed.ict.1187-NT01EI_1462-YP_002932883.1--36H-COG1/1-532   QGTDAIYTGLQEIGAGNNDLSSRTEQQAASLEETAASMEQLTATVKQNADNARQASQLAQ
Ye.ent.378-YE2573-YP_001006777.1--36H-COG1/1-545         DGTESMLTGIQEISAGNNDLSARTEQQAASLEQTAASMEQLTATVKQNADNARQATQLAQ
Ed.tar.1771-ETAF_1250-YP_005698856.1--36H-COG1/1-530     QGTDAIYTGLQEIGAGNNDLSSRTEQQAASLEETAASMEQLTATVKQNADNARQASQLAQ
Mo.mor.2189-MU9_1763-YP_007505182.1--36H-COG1/1-524      LNTDQMYTGIREIALGNNNLSSRTEQQAASLEETAASMEELTATVKQNADNARQASELAV
En.aer.1436-EAE_15540-YP_004593298.1--36H-COG1/1-536     DGAESILIGLQEIAEGNNDLSSRTEQQAASLEETAASMEQLTATVKQNADNARQASQLAR
En.638.865-Ent638_2456-YP_001177176.1--36H-COG1/1-533    QGSYAMHTGISEIAAGNNDLSSRTEQQAASLAQTAASMEELTATVSQNADNARQASDLAK
Se.pro.864-Spro_2982-YP_001479211.1--36H-COG1/1-541      DGAESILIGLQEISEGNNDLSSRTEQQAASLEETAASMEQLTATVKQNADNARQASQLAR
Es.col.1836-Y75_p1861-YP_490147.1--36H-COG1/1-533        KGSQEMHIGIAEIVAGNNDLSSRTEQQAASLAQTAASMEQLTATVGQNADNARQASELAK
Serrat.1901-SerAS13_3074-YP_006025913.1--36H-COG1/1-541  DGAESILIGLQEISEGNNDLSSRTEQQAASLEETAASMEQLTATVKQNADNARQASQLAR
Ye.pes.133-YPZ3_2023-YP_003568195.1--36H-COG1/1-536      EGTESMLTGIQEISAGNNDLSARTEQQAASLEQTAASMEQLTATVKQNADNAHQATVLAQ
Serrat.1408-SerAS12_3072-YP_004501498.1--36H-COG1/1-541  DGAESILIGLQEISEGNNDLSSRTEQQAASLEETAASMEQLTATVKQNADNARQASQLAR
Se.mar.2260-D781_2758-YP_007345194.1--36H-COG1/1-534     DGTEAMLIGLQEIAEGNNDLSSRTEQQAASLEETAASMEQLTSTVKQNADNARQATQLAR
Ye.pse.585-YPTB2400-YP_070914.1--36H-COG1/1-536          EGTESMLTGIQEISAGNNDLSARTEQQAASLEQTAASMEQLTATVKQNADNAHQATVLAQ
Se.ply.1407-SerAS9_3071-YP_004506451.1--36H-COG1/1-541   DGAESILIGLQEISEGNNDLSSRTEQQAASLEETAASMEQLTATVKQNADNARQASQLAR
Pr.stu.1965-S70_18140-YP_006218125.1--36H-COG1/1-521     TNTDQMYTGIREIALGNNNLSSRTEQQAASLEETAASMEQLTATVKQNADNARQASELAI
En.bac.2261-D782_1749-YP_007339930.1--36H-COG1/1-533     QSSYAMHTGISEIAAGNNDLSSRTEQQAASLAETAASMEQLTATVGQNADNARQASELAS
En.asb.1498-Entas_2590-YP_004829104.1--36H-COG1/1-533    QGSYAMHTGISEIAAGNNDLSSRTEQQAASLAQTAASMEQLTATVSQNADNARQASDLSK
En.asb.1498-Entas_3672-YP_004830170.1--36H-COG1/1-545    SGSENIWRGATEISSGNNDLSSRTEEQAAALEETAASMEQLTATVKLNAESARQASQLAD
Ci.kos.578-CKO_01067-YP_001452646.1--36H-COG1/1-537      QGSLAMHTGISEIAMGNNDLSSRTEQQAASLAQTAASMEQLTATVGQNADNARQASGLAK
En.clo.1544-EcWSU1_02810-YP_004952663.1--36H-COG1/1-538  QGSYAMHTGISEIAAGNNDLSSRTEQQAASLAQTAASMEQLTATVSQNADNARQASDLSR
Sa.ent.407-STM3152-NP_462067.1--36H-COG1/1-547           AGSDNIWRGATEISTGNNDLSSRTEEQAAALEETAASMEQLTATVKMNAEHARQASQLAD
Pe.atr.485-ECA1332-YP_049438.1--36H-COG1/1-555           NSTDSIYQGASEIAAGNNDLSSRTEEQASALEETAASMEQLTATVKQNAENANHASQLAL
Pe.car.1139-PC1_1208-YP_003016792.1--36H-COG1/1-555      NSTDSIYQGASEIAAGNNDLSSRTEEQASALEETAASMEQLTATVKQNAENANHASQLAL
En.638.865-Ent638_3407-YP_001178118.1--36H-COG1/1-545    SGSDNIWRGATEISSGNNNLSSRTEEQAAALEETAASMEQLTATVKLNADNARQASELAD
Pa.vag.184-Pvag_1413-YP_003931052.1--36H-COG1/1-554      SGTENISRGAAEISAGNNDLSSRTEEQAAALEQTAASMEQLTATVKFNADNARQASLLAE
Ph.asy.1114-PAU_02684-YP_003041518.1--36H-COG1/1-544     ENTNLMYSGIQEITQGNTDLSSRTEEQAASLEETAASMEQLTATVKQNAENARQASELAV
Pa.ana.1905-PAJ_1316-YP_005934192.1--36H-COG1/1-556      SGTENISRGAAEISIGNNDLSSRTEEQAAALEQTAASMEQLTATVKFNAENARQASTLTE
Ci.kos.578-CKO_04394-YP_001455885.1--36H-COG1/1-561      SGSDNIWRGATEISSGNNDLSSRTEEQAAALEETAASMEQLTATVKLNADNARQASQLAD
Sa.bon.1474-SBG_2750-YP_004731563.1--36H-COG1/1-547      AGSDNIWRGATEISTGNNDLSSRTEEQAAALEETAASMEQLTATVKLNADHARQASQLAD
Ra.aqu.1678-Q7S_16025-YP_005402985.1--36H-COG1/1-554     NCTNHIYQGASEISAGNNDLSSRTEEQASALEETAASMEELTATVKHNADNAHHASQLVM
Rahnel.1320-Rahaq_3177-YP_004213898.1--36H-COG1/1-554    NCTNHIYQGASEISAGNNDLSSRTEEQASALEETAASMEELTATVKHNADNAHHASQLVM
Pectob.2320-W5S_3114-YP_006284066.1--36H-COG1/1-555      NSTDSIYQGASEIAAGNNDLSSRTEEQASALEETAASMEQLTATVKQNAENANHASQLAL
Ye.ent.378-YE2971-YP_001007160.1--36H-COG1/1-552         SSTDGIYHGAAEISAGNTDLSSRTEQQAAALEETAASMEQLTATVKHNADNAHHASQLAA
Er.bil.197-EbC_20190-YP_003741400.1--36H-COG1/1-556      SGTDNIYRGAAEISSGNNDLSSRTEEQAAALEQTAASMEQLTATVKFNADNARQASDLAQ
Ph.lum.1262-plu1854-NP_929127.1--36H-COG1/1-544          ENTNLMYNGIQEITQGNTDLSSRTEEQAASLEETAASMEQLTATVRQNAENARQASELAV
Er.bil.197-EbC_25340-YP_003741912.1--36H-COG1/1-528      DGTHAMHTGIREIASGNNDLSARTEQQAASLAETAASMEQLTATVTGNAENARQASQLAK
Di.zea.1140-Dd1591_2937-YP_003005238.1--36H-COG1/1-564   SSADSIYQGVSEIASGNNDLAARTESQAAALEESAASMEQLTSTVKQNAENASHASQLAR
Er.tas.1011-ETA_14670-YP_001907406.1--36H-COG1/1-518     DGSRSMQSGIQEIALGNNDLSARTEQQAASLAQTAASMEQLTATVAANADNARRASELAR
Sa.ent.404-STY4234-NP_458344.1--36H-COG1/1-547           DNADSIYTGAGEISAGSSDLSSRTEQQASALEETAASMEQLTATVRQNTDNARQATGLAK
Sa.ent.407-STM3577-NP_462478.1--36H-COG1/1-547           DNADSIYTGAGEISAGSSDLSSRTEQQASALEETAASMEQLTATVRQNTDNARQATGLAK
Sa.bon.1474-SBG_3170-YP_004731982.1--36H-COG1/1-547      DNADSIYTGAGEISAGSSDLSSRTEQQASALEETAASMEQLTATVRQNTDNARQATGLAK
En.asb.1498-Entas_2591-YP_004829105.1--36H-COG1/1-555    QGSDAIYTGTSEIAMGNNDLSSRTEQQASALEETAASMEELTATVKQNADNARQASQLAE
En.638.865-Ent638_1858-YP_001176586.1--36H-COG1/1-552    GSTDSIYTGAGEIAMGSNDLSARTEQQASSLEETAASMEELTATVKQNSDNARQATLLAK
Ci.rod.62-ROD_19331-YP_003365490.1--36H-COG1/1-553       EGSDAIYAGTSEIAAGNTDLSSRTEQQASALEETAASMEELTATVKQNADNARQASQLAL
Di.dad.235-Dda3937_01559-YP_003882087.1--36H-COG1/1-566  TSADSIYQGVSEIASGNNDLAARTESQAAALEESAASMEQLTSTVKQNAENASHASQLAR
Er.bil.197-EbC_37780-YP_003743156.1--36H-COG1/1-553      DSSNAIFTGASEISLGNNDLSSRTEQQAASLEETAASMEQLTATVKQNAENARQASQLAK
Pantoe.297-Pat9b_2001-YP_004115872.1--36H-COG1/1-558     SGTENIYRGAAEISAGNNDLSSRTEEQASALEQTAASMEQLTATVKFNADNARQASSLAE
```

```
En.clo.1544-EcWSU1_01962-YP_004951821.1--36H-COG1/1-553  GSTDSIYTGAGEIAAGSNDLSARTEQQAASLEETAASMEELTATVKQNSDNARQATLLAK
En.asb.1498-Entas_1879-YP_004828403.1--36H-COG1/1-554    GSTDSIYTGAGEIAAGSNDLSARTEQQAASLEETAASMEELTATVKQNSDNARQATLLAK
En.bac.2261-D782_1748-YP_007339929.1--36H-COG1/1-547     EGTDAIYSGTSEIAAGNNDLSSRTEQQSASLEETAASMEQLTATVKQNADNARQASRLAQ
Es.col.1836-Y75_p1862-YP_490148.1--36H-COG1/1-553        EGSDAIYAGTREIAAGNTDLSSRTEQQASALEETAASMEQLTATVKQNADNARQASQLAQ
Cr.tur.6-CTU_33010-YP_003211664.1--36H-COG1/1-555        DSSESIYTGAGEISAGSNDLSSRTEQQAASLEETAASMEQLTATVKQNSDNARQASQLAK
Cr.tur.6-CTU_25780-YP_003210941.1--36H-COG1/1-536        EGTDAIYSGTSEIAAGNTDLSSRTEEQASALEQTAASMEELTATVKQNAENARQASQLAL
En.clo.1544-EcWSU1_00403-YP_004950264.1--36H-COG1/1-549  EGADTIYTGAGEISAGSNDLSSRTEQQAASLEETAASMEQLTATVKQNADNARQASRLAL
En.638.865-Ent638_2457-YP_001177177.1--36H-COG1/1-555    QGSDAIYTGTSEIAMGNNDLSSRTEQQASALEETAASMEQLTATVKQNADNARQASQLAE
Sa.bon.1474-SBG_1755-YP_004730609.1--36H-COG1/1-553      EGSDAIYSGTSEIAAGNTDLSSRTEQQASALEETAASMEQLTATVKQNADNARQASQLAQ
Pr.mir.1265-PMI1665-YP_002151396.1--36H-COG1/1-548       DNSQAMYSGIQEISKGNTDLSSRTEQQAASLEETAASMEQLTATVKQNADNALQASKLAE
Cr.sak.579-ESA_01348-YP_001437444.1--36H-COG1/1-556      EGTDAIYSGTSEIAAGNTDLSSRTEEQASALEQTAASMEELTATVKQNAENARQASQLAL
Cr.sak.579-ESA_00560-YP_001436686.1--36H-COG1/1-555      DSTESIYTGAGEISAGSNDLSARTEQQAASLEETAASMEQLTATVKQNSDNARQASQLAK
Sa.ent.407-STM1919-NP_460876.1--36H-COG1/1-553           EGSDAIYSGTSEIAAGNTDLSSRTEQQASALEETAASMEQLTATVKQNADNARQASQLAQ
Ci.kos.578-CKO_01066-YP_001452645.1--36H-COG1/1-552      EGSDAIYSGTSEIATGNTDLSSRTEQQASALEETAASMEQLTATVKQNADNARQASHLAQ
En.asb.1498-Entas_0424-YP_004826963.1--36H-COG1/1-549    QGADTIYTGAGEISAGSNDLSSRTEQQAASLEETAASMEQLTATVKQNADNARQASRLAL
Pantoe.297-Pat9b_1273-YP_004115150.1--36H-COG1/1-553     DSADAIFSSVSEIATGNNDLSSRTEQQAASLEETAASMEQLTATVKQNADNARQATQLAR
Ed.tar.1771-ETAF_2264-YP_005699859.1--36H-COG1/1-555     AGTQNIFTGVQEIAAGNNDLSSRTEEQASSLEETASSMEQISSTVKNNADSAREATDLVQ
Pa.ana.1905-PAJ_3534-YP_005936409.1--36H-COG1/1-553      DATQSIFTGAAEVSAGSTDLSSRTEQQAAALEETAASMEQLTSTVKLNAENAQQATTMAK
Pa.vag.184-Pvag_1203-YP_003930842.1--36H-COG1/1-552      DATRSIFTGAAEISAGSTDLSSRTEQQAAALEQTAASMEQLTSTVKLNADNAQQATTVSK
Pantoe.297-Pat9b_1605-YP_004115478.1--36H-COG1/1-556     DGSDAIFTGASEISAGNNDLSARTEEQAASLEQTAASMEQLTATVKQNAENARQASQLAL
Cr.sak.579-ESA_00210-YP_001436348.1--36H-COG1/1-557      DSTDTIFTGASEISAGSNDLSSRTEQQAASLEETAASMEQLTATVKQNADNARQATQLAR
Er.tas.1011-ETA_pET460340-YP_001905943.1--36H-COG1/1-543 SGTDNIHLGAAEISSGNNDLSSRTEQQASALEQTAASMEQLTATVKLNADHARQASTLAD
Cr.tur.6-CTU_36610-YP_003212024.1--36H-COG1/1-557        DSTDTIFTGASEISAGSNDLSSRTEQQAASLEETAASMEELTATVKQNADNARQATQLAR
Pe.car.1139-PC1_1159-YP_003016743.1--36H-COG1/1-556      DSSHSIHSGASEISVGSNDLSSRTEQQAASLQETAASMEQLTSTVKQNSDNARQATQLAK
Er.tas.1011-ETA_14640-YP_001907403.1--36H-COG1/1-554     DGSDAIYTGASEISSGNNDLSARTEQQAASLEQTAASMEELTATVKQNAENARQASKLAL
Pa.vag.184-Pvag_1725-YP_003931362.1--36H-COG1/1-556      EGSDAIFTGASEISAGNNDLSARTEQQAASLEQTAASMEQLTATVKQNAENARQASQLAL
Ed.ict.1187-NT01EI_3742-YP_002935104.1--36H-COG1/1-525   NGADTLLHGSSDITVGNADLSSRTEQQAAALEETAASMEQLTATVRQNADNARQASQLAS
En.638.865-Ent638_0380-YP_001175119.1--36H-COG1/1-549    EGADTIFTGAGEISAGSNDLSSRTEQQAASLEETAASMEQLTATVKQNADNARQASRLAK
Er.bil.197-EbC_25360-YP_003741914.1--36H-COG1/1-556      DGSDAIYTGASEIAAGNNDLSARTEQQAASLEETAASMEQLTATVKQNAENARQASQLAL
Er.tas.1011-ETA_16910-YP_001907630.1--36H-COG1/1-543     SGTDNIHRGAAEISSGNNDLSSRTEQQASALEQTAASMEQLTATVKLNADHARQASTLAD
Es.fer.1173-EFER_3055-YP_002384154.1--36H-COG1/1-603     NGADAIYSGASEIAMGNNDLSSRTEQQAASLEETAASMEQLTATVKQNAENARQASHLAL
Xe.nem.162-XNC1_1619-YP_003711880.1--36H-COG1/1-520      DNTNQMYIGIQEITQGNTDLSSRTEEQAASLEETAASMEELTVTVKQNAENARQASELAV
En.clo.1544-EcWSU1_02811-YP_004952664.1--36H-COG1/1-559  NGSEAIYTGTSEIAMGNNDLSSRTEQQASALEETAASMEQLTATVKQNADNARQASQLAE
Di.dad.235-Dda3937_03500-YP_003882750.1--36H-COG1/1-553  ASSSVIYTGTSEIRQGNDELSRRTDQQAAALQETAASMEELTSTVKNNADNVRQARQISE
Xe.bov.105-XBJ1_1924-YP_003467828.1--36H-COG1/1-523      DNTNQMYAGIQEITQGNTDLSSRTEEQAASLEETAASMEELTVTVKQNAENARQASELAV
Rahnel.1320-Rahaq_1831-YP_004212576.1--36H-COG1/1-556    HGADTMFSGLKGIAAGNTDLSSRTEQQASALEETAASMEQLTATVKQNAENARQATQLAR
Ra.aqu.1678-Q7S_08965-YP_005401598.1--36H-COG1/1-556     HGADTMFSGLKGIAAGNTDLSSRTEQQASALEETAASMEQLTATVKQNAENARQATQLAR
Cr.sak.579-ESA_01126-YP_001437230.1--36H-COG1/1-514      HGSENIWRGASEISAGNNDLSSRTEEQAAALEQTAASMEELTSTVRLNAENARQASELAH
Pe.atr.485-ECA2712-YP_050803.1--36H-COG1/1-560           DSSASIHQGSSEIKSGNNDLSARTEQQAAALEQTAASMEELSATVKQNADNVHQATKLAQ
En.clo.1544-EcWSU1_00560-YP_004950421.1--36H-COG1/1-597  NGANAIYSGASEIAMGNNDLSSRTEQQAASLEETAASMEQLTATVKQNAENARQASNLAL
Di.dad.235-Dda3937_03498-YP_003882752.1--36H-COG1/1-561  STAEAIYQGATEIRQGNNDLSSRTEQQASALQETAASMEQLSSTVQHNAENVHQATKLAH
Cr.tur.6-CTU_27880-YP_003211151.1--36H-COG1/1-514        HGSENIWRGASEISAGNNDLSSRTEEQAAALEQTAASMEELTSTVRLNAENARQASELAH
Pe.car.1139-PC1_1669-YP_003017246.1--36H-COG1/1-560      DSSASIHQGSSEIKSGNNDLSARTEQQAAALEQTAASMEELSATVKQNADNVHQATKLAQ
Ra.aqu.1678-Q7S_14450-YP_005402672.1--36H-COG1/1-551     NSADSIYKGSSEISTGNTDLSSRTEEQASALEETAASMEQLSSTVRHNADNASQASTLAE
Ci.kos.578-CKO_03622-YP_001455137.1--36H-COG1/1-517      EGADTIYTGAGEIATGSSDLSSRTEQQAASLEETAASMEQLTATVRQNADNARQASSLAK
Di.zea.1140-Dd1591_2408-YP_003004728.1--36H-COG1/1-557   AGSSVIYTGTSEIRQGNGELSRRTDQQAAALQETAASMEELTSTVKNNADNVRQARQISE
Di.zea.1140-Dd1591_2406-YP_003004726.1--36H-COG1/1-559   DTAEAIYQGATEIRQGNNDLSGRTEQQASALQETAASMEQLSSTVQHNAENVHQATQLAQ
Ed.ict.1187-NT01EI_2801-YP_002934203.1--36H-COG1/1-554   AGTQNIFTGVQEIAAGNNDLSSRTEEQASSLEETASSMEQISSTVKNNADSAREATDLVQ
Pe.car.1139-PC1_1384-YP_003016966.1--36H-COG1/1-559      QSTTEINNGTSSIKDGNDDLSSRTEQQAAALQQTAASMEEISSTVRQTTDHVHQARKLAK
Rahnel.1320-Rahaq_2866-YP_004213595.1--36H-COG1/1-551    NSADSIYKGSSEISTGNTDLSSRTEEQASALEETAASMEQLSSTVRHNADNASQASTLAE
Pe.atr.485-ECA1281-YP_049387.1--36H-COG1/1-556           DSSNSIHSGASEISVGSNDLSSRTEQQAASLQETAASMEQLTSTVKQNSDNARQATLLAK
Pectob.2320-W5S_1886-YP_006282848.1--36H-COG1/1-549      DSSASIHQGSSEIKSGNNDLSARTEQQAAALEQTAASMEELSATVKQNADNVHQATKLAQ
Pectob.2320-W5S_1696-YP_006282659.1--36H-COG1/1-559      QSTSEINNGTSSIKDGNDNLSSRTEEQAAALQQTAASMEEISSTIRQTTDHVHQARKLAQ
Di.zea.1140-Dd1591_2407-YP_003004727.1--36H-COG1/1-555   GSSTVIYTGTSEIRQGNDELSRRTDQQAAALQETAASMEELTSTVKNNADNVRQARQISE
Pe.atr.485-ECA1509-YP_049611.1--36H-COG1/1-559           QSTTEINNGTSSIKDGNDNLSSRTEQQAAALQETAASMEEISSTVRQTTDHVHQVRQLAK
Pa.vag.184-Pvag_1724-YP_003931361.1--36H-COG1/1-520      DGSDAIFTGASEIAAGNNDLSARTEEQAASLEQTAASMEQLTATVKQNADNARQASQLAL
Pa.ana.1905-PAJ_1541-YP_005934417.1--36H-COG1/1-520      SGCDAIFTGASEIAAGNNDLSARTEEQAASLEQTAASMEQLTATVKQNADNARQASQLAQ
Mo.mor.2189-MU9_1596-YP_007505015.1--36H-COG1/1-519      EGSDTLRDSASEIARGNTDLSSRTEQQAAALEETAASMEEITATVKLNADHAHHASELAE
Pr.mir.1265-PMI2808-YP_002152509.1--36H-COG1/1-563       NSSTTIYSGVQEIAAGNNDLSSRTEEQASALEETASSMEQLTATVRNNTESAREVTQLVM
Di.zea.1140-Dd1591_0395-YP_003002764.1--36H-COG1/1-556   RSASEIYHGSSEIAAGNSDLSSRTEEQAAALSETAASMEQLSAVVKQNADNAQQASVLAK
En.clo.1544-EcWSU1_03786-YP_004953635.1--36H-COG1/1-515  SGSENIWRGATEISSGNNDLSSRTEEQAAALEETAASMEQLTATVKLNAESARQASQLAD
Pectob.2320-W5S_3170-YP_006284116.1--36H-COG1/1-556      DSSHSIHSGASEISAGSHDLSSRTEQQAASLQETAASMEQLTSTVRQNSDNARQATLLAK
Di.dad.235-Dda3937_02184-YP_003884754.1--36H-COG1/1-556  RSASEIYHGSSEIAAGNADLSSRTEEQAAALSETAASMEQLSAVVKQNADNAHQASSLAR
Ci.rod.62-ROD_32641-YP_003366748.1--36H-COG1/1-517       DGADTIYTGAGEISAGSNDLSSRTEQQAASLEETAASMEQLTATVRQNADNARQATQLAI
Di.dad.235-Dda3937_03499-YP_003882751.1--36H-COG1/1-561  TSSSVIHTGTSEIRQGNDELSRRTDQQASALQETAASMEELTSTVKNNADNVRQARQISE
Di.dad.235-Dda3937_00105-YP_003883346.1--36H-COG1/1-561  VSAHEIYRSASEIAGGNSDLSSRTEQQSASLVQTAASMEQLTATVKQNADNARHASQLAR
Pantoe.297-Pat9b_1604-YP_004115477.1--36H-COG1/1-555     DGSDAIFTGASEISAGNNDLSARTEEQAASLEQTAASMEQLTATVKQNAENARQASQLAL
Er.tas.1011-ETA_14660-YP_001907405.1--36H-COG1/1-560     DGSEAIYTGASEISSGNNDLSSRTEQQAASLEQTAASMEELTATVKQNAENARQASQLAL
Pr.mir.1265-PMI2809-YP_002152510.1--36H-COG1/1-575       GTSHHIYNGVQELSAGNNDLSSRTQEQASALEETASSMEQLTATVKNNTESAREVSHLIN
Ed.tar.1771-ETAF_3053-YP_005700648.1--36H-COG1/1-516     GGADALLHGSGDITVGNADLSSRTEQQAAALEETAASMEQLTATVRQNADNARQASQLAS
Se.pro.864-Spro_1415-YP_001477647.1--36H-COG1/1-546      AGTQNIYTGVQEIAAGNNDLSSRTEEQASSLEETAASMEQLSATVKNNADSAAAATSLVK
Di.zea.1140-Dd1591_1804-YP_003004134.1--36H-COG1/1-562   ISADNIYHRASEIAGGNNDLSSRTEQQSAALVQTAASMEQLTATVKQNADNARHASQLAR
Di.dad.235-Dda3937_03501-YP_003882749.1--36H-COG1/1-575  ASSSVIYTGTSEIRQGNDELSRRTDQQAAALQETAASMEELTSTVKNNADNVRQARQISE
Pantoe.297-Pat9b_0851-YP_004114731.1--36H-COG2/1-524     AGSDTIAVASNQISSGNLDLSSRTEQQASSLEETASAMEQMTSTVKHNAENAREANKLVA
Pa.vag.184-Pvag_0291-YP_003929953.1--36H-COG2/1-525      TGSDTIAVASNQISSGNLDLSTRTEQQASSLEETASAMEQMTSTVKHNADNAREANHLVS
En.clo.1544-EcWSU1_03886-YP_004953735.1--36H-COG2/1-519  TGSENISSAAAQIVAGNQDLAARTEEQASSVEQTAASMEQITATVKNTASHTGEATHLSA
Cr.tur.6-CTU_04790-YP_003208842.1--36H-COG2/1-515        YSAESISSAASQIVAGNQDLAARTEEQASSVEQTAASMEQITSTVKNTADNTSEATRLSS
Ci.kos.578-CKO_04484-YP_001455975.1--36H-COG2/1-521      NGSENISSAAAQIVAGNQDLAARTEEQASSVEQTAASMEQITATVKNTADHTSEATHLSA
Cr.sak.579-ESA_03488-YP_001439539.1--36H-COG2/1-515      YSAESISSAASQIVAGNQDLAARTEEQASSVEQTAASMEQITSTVKNTADNTSEATKLSS
Ci.rod.62-ROD_48071-YP_003368199.1--36H-COG2/1-521       NGSENISSAAAQIVAGHKDLAARTEEQVSSVEQTAASMEQITATVKNTAEHTAEATKLSA
Sa.bon.1474-SBG_2851-YP_004731664.1--36H-COG2/1-521      NGSESISTAAAQIVAGNQDLAARTEEQASSVEETAASMEQITATVKNTADHTSEATKLSA
Sa.ent.407-STM3216-NP_462130.1--36H-COG2/1-521           NGSESISTAAAQIVAGNQDLAARTEEQASSVEETAASMEQITATVKNTADHTSEATKLSA
En.asb.1498-Entas_3753-YP_004830248.1--36H-COG2/1-519    TGSENISSAAAQIVAGNQDLAARTEEQASSVEQTAASMEQITATVKNTASHTGEATNLSA
Mo.mor.2189-MU9_3389-YP_007506806.1--36H-COG2/1-521      NGAVSLSATAEQIVAGNQNLAARTEEQAASVEETAASMEQITATVKNTAANTLKATALSA
Sa.ent.404-STY3394-NP_457606.1--36H-COG2/1-522           NGSESISTAAAQIVAGNQDLAARTEEQASSVEETAASMEQITATVKNTADHTSEATKLSA
En.638.865-Ent638_3526-YP_001178235.1--36H-COG2/1-519    TGSENISSAAAQIVAGNQDLAARTEEQASSVEQTAASMEQITATVKNTAAHTGEATNLSA
Pe.atr.485-ECA1774-YP_049874.1--36H-COG2/1-554           DGAETISSAASQIAAGNQDLSARTEEQASSLEETASSMEQLTSTIRNTADNTTQATDLAA
En.638.865-Ent638_2119-YP_001176845.1--36H-COG2/1-549    HGAESISVAADQIDAGNQDLASRTEEQAAGVVETAATLEQLTSTIKSTADNVRRVNELFT
```

```
Pectob.2320-W5S_2813-YP_006283770.1--36H-COG2/1-554      DGAETISSAASQIAAGNQDLSARTEEQASSLEETASSMEQLTSTIRNTADNTTQATDLAA
Di.zea.1140-Dd1591_0695-YP_003003055.1--36H-COG2/1-561   DGAEAISSAASQIAAGNQDLSSRTEEQASSLEQTAASMEELVSTIKNTADNTRQATSIAN
Pa.vag.184-Pvag_pPag30079-YP_003729820.1--36H-COG2/1-546 RNAESVAAASLQIAQGNSDLSQRTEEQASALEQTAATMSELSVTVRNNADNARQAAQLAV
Er.bil.197-EbC_38940-YP_003743272.1--36H-COG2/1-526      SGTDLIHNASSEISAGNMDLSSRTEQQASSLEETASAMEQMTSTVKQNADNARQANELAA
Ra.aqu.1678-Q7S_24891-YP_005419232.1--36H-COG2/1-554     AGTNAITSASSEIAAGNLDLSSRTEQQASSLEETASAMEQMTATVKQNADNARQANLLAA
En.asb.1498-Entas_2287-YP_004828805.1--36H-COG2/1-513    NSVAQVSQAASDIAEGNTDLSSRTEQQAAAVVETAASMEELTATVKNNADNARHASQLAA
Rahnel.1320-Rahaq_4869-YP_004215574.1--36H-COG2/1-554    AGTNAITSASSEIAAGNLDLSSRTEQQASSLEETASAMEQMTATVKQNADNARQANLLAA
Di.dad.235-Dda3937_00027-YP_003883574.1--36H-COG2/1-554  DGAETISSAASQIAAGNQDLSARTEEQASSLEQTAASIEQLTATIKNTAANTEHAASLAG
Pr.stu.1965-S70_05790-YP_006215722.1--36H-COG2/1-520     NGSESISAAAGQIVAGNQHLAARTEEQATSVEETASSMEQITSTVQNTAEHTHQATQLAA
En.638.865-Ent638_2100-YP_001176826.1--36H-COG2/1-512    TSVAQVSLAASEIAEGNTDLSSRTEQQAAAVVETAASMEELTATVKNNADNARHASKLAA
Pe.car.1139-PC1_2526-YP_003018093.1--36H-COG2/1-554      DGAETISSAASQIAAGNQDLSARTEEQASSLEETASSMEQLTSTIRNTADNTTQATDLAA
Mo.mor.2189-MU9_702-YP_007504121.1--36H-COG2/1-525       NNSDSIVTGTMQITSGNADLSQRTESQASSLQQTAASMEQLSATVKSSAEIAAKANMMAA
Pe.car.1139-PC1_3443-YP_003018995.1--36H-COG2/1-511      LSANQISYAAGEISAGNADLSSRTTQQAAALEETAASMEQLTSTVKQNADNAHQANRLVM
Pa.ana.1905-PAJ_0915-YP_005933791.1--36H-COG2/1-559      SNAESVASASTQIAQGNTDLSQRTEEQASALEQTSATMTQLGVTVKNNADNARQAGQLAV
Pectob.2320-W5S_3736-YP_006284671.1--36H-COG2/1-510      LSANQISSAAGEITAGNADLSVRTTQQAAALEETAASMEQLTSTVKQNADNAHHANRLVM
Pe.atr.485-ECA0183-YP_048310.1--36H-COG2/1-553           DGAETISSAASQIAAGNQDLSSRTEEQASSLEETAASMEQLTSTIKNTAENTQQATDIAN
Rahnel.1320-Rahaq_3107-YP_004213828.1--36H-COG2/1-503    EGVTQVTRASGEIAAGNIDLSARTEEQAAALGQTAASMEQLTSTVKQNADNAAQASQLAS
Pe.car.1139-PC1_4071-YP_003019622.1--36H-COG2/1-553      DGAETISTAASQIAAGNQDLSARTEEQASSLEETAASMEQLTSTIKNTAENTQQATEIAN
Di.dad.235-Dda3937_03462-YP_003884507.1--36H-COG2/1-563  DGAEAISTAASQIAAGNQDLSSRTEEQASSLEQTAASMEELVSTIKNTAENTQQATSIAN
Ra.aqu.1678-Q7S_15665-YP_005402913.1--36H-COG2/1-503     EGVTQVTRASGEIAAGNIDLSARTEEQAAALGQTAASMEQLTSTVKQNADNAAQASQLAS
Pectob.2320-W5S_4505-YP_006285423.1--36H-COG2/1-555      DGAETISTAASQIAAGNQDLSSRTEEQASSLEETAASMEQLTSTIKNTAENTQQATDIAN
Pe.atr.485-ECA3642-YP_051730.1--36H-COG2/1-551           QGSESIAGASSQLAAGNIDLSSRTEEQASALQETAASIEQLSSTVRQNADNARQANQLAQ
Pe.car.1139-PC1_1417-YP_003016999.1--36H-COG2/1-554      NNAESVATASMQIAQGNADLSSRTEEQASALEETSSTMTQLGMTVKNNADNALQANVLAK
En.asb.1498-Entas_4509-YP_004821569.1--36H-COG2/1-559    TSALSVSAASSQIATGNQNLSARTDEQASALAETASALEQLTATVGNTAENARHAQQLVS
Pa.ana.1905-PAJ_3064-YP_005935940.1--36H-COG2/1-544      SNAESVASASSQIAQGNADLSQRTEEQASALEQTAATMTQLGMTVKNNASNAQQASQLAV
Pectob.2320-W5S_1733-YP_006282696.1--36H-COG2/1-535      NNAESVATASMQIAQGNADLSSRTEEQASALEETSSTMTQLGMTVKNNADNARQANTLAQ
Pe.atr.485-ECA4334-YP_052421.1--36H-COG2/1-556           QSSESIATGATQIAMGNTDLSQRTEEQAANLQETAASMEQMNTTVKQNAATVRTATELAH
Di.zea.1140-Dd1591_1602-YP_003003935.1--36H-COG2/1-554   DGAETISSAASQIAAGNQDLSARTEEQASSLEQTAASIEQLTSTIKNTAANTDHAASLAG
Pectob.2320-W5S_4506-YP_006285424.1--36H-COG2/1-540      DGAETISTAASQITAGNQDLSSRTEEQASSLEETAASMEQLASTIKNTAENTQQATEIAN
Di.zea.1140-Dd1591_1453-YP_003003794.1--36H-COG2/1-532   HGAESVRMASGEIAHGNHDLSARTESQASALEQTAASMEQLSATVKQNADNARQANQLAQ
Pectob.2320-W5S_0106-YP_006281117.1--36H-COG2/1-556      QSSESIATGATQIAMGNTDLSQRTEEQAANLQETAASMEQMNTTVKQNAATVRTATELAH
Di.zea.1140-Dd1591_0769-YP_003003127.1--36H-COG2/1-505   LSADQISHAAGEIVAGNTDLSSRTEQQAAAIEQTAASMEQLTSTVKQNADNAHHANTLVV
Ra.aqu.1678-Q7S_18035-YP_005403387.1--36H-COG2/1-535     NSAESIVSASTEITLGNEDLSRRTEAQASSLEQTSASMEELTATVKHNADNAHQGNLMAT
Rahnel.1320-Rahaq_3579-YP_004214298.1--36H-COG2/1-535    NSAESIVSASTEITLGNEDLSRRTEAQASSLEQTSASMEELTATVKHNADNAHQGNLMAT
Pe.car.1139-PC1_4072-YP_003019623.1--36H-COG2/1-540      DGAETISTAASQITAGNQDLSSRTEEQASSLEETAASMEQLASTIKNTAENTQQATEIAN
Pe.car.1139-PC1_3464-YP_003019016.1--36H-COG2/1-551      QGSESIAGASSQLAAGNIDLSSRTEEQASALQETAASIEQLSSTVRQNADNARQANQLAQ
Pe.car.1139-PC1_0111-YP_003015709.1--36H-COG2/1-556      QSSESIATGATQIAMGNTDLSQRTEEQAANLQETAASMEQMNTTVKQNAATVRTATELAH
Pe.atr.485-ECA0182-YP_048309.1--36H-COG2/1-542           DGAETISTAASQITAGNQDLSSRTEEQASSLEETAASMEQLASTIKNTAENTQQATEIAN
Pectob.2320-W5S_3753-YP_006284688.1--36H-COG2/1-551      QGSESIAGASSQLAAGNIDLSSRTEEQASALQETAASIEQLSSTVRQNADNARQANQLAQ
Pa.ana.1905-PAJ_2666-YP_005935542.1--36H-COG2/1-514      ESAGSVASASDEIAQGNTELSSRTEQQAAALQETAASMEQLTATVKSNTASAQHTATVAR
Ci.rod.62-ROD_16161-YP_003365194.1--36H-COG6/1-557       QGAEEIYRGTSEISAGNTDLSSRTEEQAAAIEQTAASMEELTATVKQNADNAHHASKLAE
En.asb.1498-Entas_2095-YP_004828614.1--36H-COG6/1-563    QGAEEIYRGTSEISAGNTDLSSRTEQQAAAIEQTAASMEELTATVKQNADNAHHASKLAE
En.638.865-Ent638_1961-YP_001176688.1--36H-COG6/1-563    QGAEEIYRGTSEISAGNTDLSSRTEQQAAAIEQTAASMEELTATVKQNADNAHHASKLAE
Cr.sak.579-ESA_01710-YP_001437800.1--36H-COG6/1-565      EGAQAIYQGTSEISAGNTDLSSRTEQQAAALEQTAASMEQLTATVKQNADNAHHASKLAQ
Cr.tur.6-CTU_22440-YP_003210607.1--36H-COG6/1-565        EGAQAIYQGTSEISAGNTDLSSRTEQQAAALEQTAASMEQLTATVKQNADNAHHASKLAQ
Ci.kos.578-CKO_01456-YP_001453025.1--36H-COG6/1-562      QGAEEIYRGTSEISAGNTDLSSRTEEQAAAIEQTAASMEELTATVKQNADNAHHASKLAE
Rahnel.1320-Rahaq_1930-YP_004212674.1--36H-COG6/1-566    EGAVSIYQGSTEIASGNTDLSSRTEQQAAALEETAASMEQLTATVKQNSENAHHASKLAA
Pantoe.297-Pat9b_2403-YP_004116261.1--36H-COG6/1-564     EGAVAIYQGSSEISAGNTDLSSRTEQQASALEQTAASMEQLTATVKQNAENAHHASQLAA
Er.bil.197-EbC_26700-YP_003742048.1--36H-COG6/1-567      EGAVAIYQGSSEISAGNTDLSSRTEQQAAALEQTAASMEQLTATVKQNAENAHHASQLAA
Pectob.2320-W5S_1760-YP_006282723.1--36H-COG6/1-570      ESADSIYQGSTEISSGNTDLSSRTEQQAAALEQTAASMEQLTATVKQNSENAHHASQLAA
Pa.ana.1905-PAJ_1621-YP_005934497.1--36H-COG6/1-568      EGAIAIYQGSSEISAGNTDLSSRTEQQAAALEQTAASMEQLTATVKQNADNAHHASQLAA
En.clo.1544-EcWSU1_02120-YP_004951978.1--36H-COG6/1-576  QGAEEIYRGTSEISAGNTDLSSRTEQQAAAIEQTAASMEQLTATVKQNADNAHHASKLAE
Ra.aqu.1678-Q7S_09815-YP_005401764.1--36H-COG6/1-566     EGAVSIYQGSTEIASGNTDLSSRTEQQAAALEETAASMEQLTATVKQNSENAHHASKLAA
Ye.pse.585-YPTB2412-YP_070925.1--36H-COG6/1-579          SSAESIYQGSSEIALGNTDLSARTEQQAASLEQTAASMEQLTATVKQNAENAHHASQLAA
Ye.ent.378-YE2588-YP_001006789.1--36H-COG6/1-579         SSAESIYQGSSEIALGNTDLSARTEQQAASLEQTAASMEQLTATVKQNAENAHHASQLAA
Di.dad.235-Dda3937_02787-YP_003883649.1--36H-COG6/1-572  DSADSIYQGTTEITAGNSDLSSRTEQQAAAIEETAASMEQLTATVKQNSDNAHHASQLAS
Pa.vag.184-Pvag_1800-YP_003931435.1--36H-COG6/1-568      EGAVAIYQGSSEISAGNTDLSSRTEQQAAALEQTAASMEQLTATVKQNAENAHHASQLAA
En.bac.2261-D782_2307-YP_007340470.1--36H-COG6/1-558     QGAEEIYRGTSEISVGNTDLSARTEQQAAAIEQTAASMEELTATVKQNADNAHHASKLAE
Pe.atr.485-ECA1683-YP_049784.1--36H-COG6/1-573           ESADSIYQGSTEISSGNTDLSSRTEQQAAALEQTAASMEQLTATVKQNSENAHHASQLAA
Pe.car.1139-PC1_2617-YP_003018183.1--36H-COG6/1-573      ESADSIYQGSTEISSGNTDLSSRTEQQAAALEQTAASMEQLTATVKQNSENAHHASQLAA
Di.zea.1140-Dd1591_1535-YP_003003868.1--36H-COG6/1-574   DSADSIYQGTTEITAGNSDLSSRTEQQAAAIEQTAASMEQLTATVKQNSDNAHNASQLAS
Di.zea.1140-Dd1591_3580-YP_003005868.1--36H-COG6/1-572   HGVEAIYQGITEISAGNSDLSSRTEEQASALEQTAASMEQLTSTVRHNADNANHASQLAG
Es.col.1836-Y75_p1397-YP_489687.1--36H-COG6/1-546        QGAEEIYRGTSEISAGNADLSSRTEEQAAAIEQTAASMEQLTATVKQNADNAHHASKLAQ
Pe.atr.485-ECA3902-YP_051990.1--36H-COG6/1-575           EGADSIYQGSSEITAGNVDLSSRTEQQAAALEETAASMEQLTATVKQNSDNAHHASQLAK
Sa.bon.1474-SBG_1454-YP_004730321.1--36H-COG6/1-537      QSAEEIYRGTSEITAGNTDLSSRTEEQAAAIEQTAASMEQLTATVKQNADNAHHASKLAE
Sa.ent.407-STM1626-NP_460585.1--36H-COG6/1-541           QGAEEIYRGTSEITAGNTDLSSRTEEQAAAIEQTAASMEQLTATVKQNADNAHHASKLAE
Pe.car.1139-PC1_3679-YP_003019230.1--36H-COG6/1-567      EGADSIYQGSSEITAGNVDLSSRTEQQAAALEETAASMEQLTATVKQNSDNAHHASQLAK
Pectob.2320-W5S_4020-YP_006284955.1--36H-COG6/1-575      EGADSIYQGSSEITAGNVDLSSRTEQQAAALEETAASMEQLTATVKQNSDNAHHASQLAK
Di.zea.1140-Dd1591_0523-YP_003002884.1--36H-COG6/1-568   EGADSIYQGASEISAGNVDLSSRTEQQAAALEQTAASMEELTSTVKQNSDNANHASQLAR
Di.dad.235-Dda3937_02665-YP_003881279.1--36H-COG6/1-572  HGVEAIYQGITEISAGNSDLSSRTEEQASALEQTASSMEELTSTVRHNADNANHASQLAG
```

```
Pr.mir.1265-PMI1666-YP_002151397.1--36H-COG1/1-568       DASDIARQGGKVVANVVQTMHDIAGSSQKITDITAVIDGIAFQTNILALNAAVEAARAGE
Di.dad.235-Dda3937_02779-YP_003883640.1--36H-COG1/1-566  SASETAQKGGKVVDNVVKTMHNIAGSSQKIADITSVIDGIAFQTNILALNAAVEAARAGE
Di.zea.1140-Dd1591_1542-YP_003003875.1--36H-COG1/1-566   SASETAQKGGKVVDNVVKTMHNIAGSSQKIADITSVIDGIAFQTNILALNAAVEAARAGE
Pe.atr.485-ECA1691-YP_049792.1--36H-COG1/1-561           SASETAQKGGKVVDNVVRTMHNIAGSSQKIADITSVIDGIAFQTNILALNAAVEAARAGE
Se.pro.864-Spro_2983-YP_001479212.1--36H-COG1/1-556      SASETAQKGGKVVANVVQTMHDIAGSSQKIADITGVIDGIAFQTNILALNAAVEAARAGE
Ph.asy.1114-PAU_02685-YP_003041519.1--36H-COG1/1-564     SASEIARQGGKVVANVVQTMHEIAGSSQKISDITSVIDAIAFQTNILALNAAVEAARAGE
Pr.stu.1965-S70_18135-YP_006218124.1--36H-COG1/1-561     QASAIAKEGGKVVSNVVNTMLNIAESSQKISDITAVIDSIAFQTNILALNAAVEAARAGE
Mo.mor.2189-MU9_1762-YP_007505181.1--36H-COG1/1-561      QASDIAREGGKVVSNVVNTMLNIADSSQKISDITAVIDSIAFQTNILALNAAVEAARAGE
Pe.car.1139-PC1_2609-YP_003018175.1--36H-COG1/1-561      SASETAQKGGKVVDNVVKTMHNIAGSSQKIADITSVIDGIAFQTNILALNAAVEAARAGE
Xe.nem.162-XNC1_1620-YP_003711881.1--36H-COG1/1-567      NASDIARQGGKVVANVVQTMHEIADSSRKISDITGVIDAIAFQTNILALNAAVEAARAGE
Pectob.2320-W5S_1768-YP_006282731.1--36H-COG1/1-561      NASETAQKGGKVVDNVVKTMHNIAGSSQKIADITSVIDGIAFQTNILALNAAVEAARAGE
Pa.vag.184-Pvag_1723-YP_003931360.1--36H-COG1/1-558      TASETAQQGGKVVDGVVTTMKEIAGSSKKIADITSVIDGIAFQTNILALNAAVEAARAGE
Ye.pes.133-YPZ3_2024-YP_003568196.1--36H-COG1/1-557      SASETAQKGGKVVADVVQTMHEIAGSSQKIADITSVIDGIAFQTNILALNAAVEAARAGE
Ye.pse.585-YPTB2401-YP_070915.1--36H-COG1/1-557          SASETAQKGGKVVADVVQTMHEIAGSSQKIADITSVIDGIAFQTNILALNAAVEAARAGE
Ye.ent.378-YE2575-YP_001006778.1--36H-COG1/1-557         SASETAQKGGKVVANVVQTMHDIAGSSQKIADITSVIDGIAFQTNILALNAAVEAARAGE
Pa.ana.1905-PAJ_1540-YP_005934416.1--36H-COG1/1-563      NASETAQQGGKVVDGVVTTMKEIAGSSRKIADIISVIDGIAFQTNILALNAAVEAARAGE
Ph.lum.1262-plu1853-NP_929126.1--36H-COG1/1-564          SASEVARQGGKVVANVVQTMREIAGSSQKISDITSVIDAIAFQTNILALNAAVEAARAGE
Serrat.1901-SerAS13_3075-YP_006025914.1--36H-COG1/1-555  SASETAQKGGKVVANVVQTMHDIAGSSQKIADITGVIDGIAFQTNILALNAAVEAARAGE
Sa.ent.404-STY2128-NP_456485.1--36H-COG1/1-553           SASETARHGGKVVDGVVNTMHEIADSSKKIADIISVIDGIAFQTNILALNAAVEAARAGE
Ra.aqu.1678-Q7S_08960-YP_005401597.1--36H-COG1/1-564     SASETAQKGGKVVANVVDTMNDIASSSKKITDITGVIDGIAFQTNILALNAAVEAARAGE
Se.mar.2260-D781_2759-YP_007345195.1--36H-COG1/1-555     SASETAQKGGKVVADVVQTMHDIAGSSQKIADITGVIDGIAFQTNILALNAAVEAARAGE
Rahnel.1320-Rahaq_1830-YP_004212575.1--36H-COG1/1-564    SASETAQKGGKVVANVVDTMNDIASSSKKITDITGVIDGIAFQTNILALNAAVEAARAGE
Se.ply.1407-SerAS9_3072-YP_004506452.1--36H-COG1/1-555   SASETAQKGGKVVANVVQTMHDIAGSSQKIADITGVIDGIAFQTNILALNAAVEAARAGE
Ed.ict.1187-NT01EI_1461-YP_002932882.2--36H-COG1/1-555   SASETAQRGGKVVANVVQTMHDIAGSSQKIADITGVIDGIAFQTNILALNAAVEAARAGE
En.aer.1436-EAE_15535-YP_004593297.1--36H-COG1/1-556     SASETAQKGGKVVANVVETMHDIASSSQKIADITGVIDGIAFQTNILALNAAVEAARAGE
Xe.bov.105-XBJ1_1923-YP_003467827.1--36H-COG1/1-567      NASDIARQGGKVVANVVQTMHDIADSSRKISDITGVIDAIAFQTNILALNAAVEAARAGE
Serrat.1408-SerAS12_3073-YP_004501499.1--36H-COG1/1-555  SASETAQKGGKVVANVVQTMHDIAGSSQKIADITGVIDGIAFQTNILALNAAVEAARAGE
Er.bil.197-EbC_25350-YP_003741913.1--36H-COG1/1-559      SASETAQKGGKVVDGVVKTMNDIAGSSKKIADITSVIDGIAFQTNILALNAAVEAARAGE
Er.tas.1011-ETA_14650-YP_001907404.1--36H-COG1/1-554     SASETALKGGKVVDGVVKTMSEISTSSKQIADIISVIDGIAFQTNILALNAAVEAARAGE
Sa.ent.407-STM4533-NP_463392.1--36H-COG1/1-553           SASETAQKGGKVVDNVVQTMRDIASSSQKIADIISVIDGIAFQTNILALNAAVEAARAGE
Es.col.1836-Y75_p4240-YP_492486.1--36H-COG1/1-551        SASETAQRGGKVVDNVVQTMRDISTSSQKIADIISVIDGIAFQTNILALNAAVEAARAGE
En.bac.2261-D782_3913-YP_007342002.1--36H-COG1/1-553     SASETAQKGGKVVDNVVQTMRDITASSQKIADIISVIDGIAFQTNILALNAAVEAARAGE
Ci.kos.578-CKO_03442-YP_001454958.1--36H-COG1/1-554      SASETAQKGGKVVDNVVQTMRDIASSSQKIADIISVIDGIAFQTNILALNAAVEAARAGE
Cr.sak.579-ESA_03402-YP_001439456.1--36H-COG1/1-555      SASETAQKGGKVVDNVVQTMRDIAGSSQKIADITSVIDGIAFQTNILALNAAVEAARAGE
Pantoe.297-Pat9b_1606-YP_004115479.1--36H-COG1/1-558     TASETAQQGGKVVDGVVTTMKEIAGSSKKIADITSVIDGIAFQTNILALNAAVEAARAGE
Cr.tur.6-CTU_05670-YP_003208930.1--36H-COG1/1-555        SASETAQKGGKVVDNVVQTMRDIAGSSQKIADITSVIDGIAFQTNILALNAAVEAARAGE
Sa.bon.1474-SBG_3944-YP_004732724.1--36H-COG1/1-553      SASETAQKGGKVVDNVVQTMRDIASSSQKIADIISVIDGIAFQTNILALNAAVEAARAGE
En.asb.1498-Entas_0547-YP_004827084.1--36H-COG1/1-554    SASETAQKGGKVVDNVVQTMRDIAGSSQKIADIISVIDGIAFQTNILALNAAVEAARAGE
Ci.rod.62-ROD_48461-YP_003368233.1--36H-COG1/1-554       SASETAQKGGKVVDNVVETMRDIATSSQKIADIISVIDGIAFQTNILALNAAVEAARAGE
Ed.tar.1771-ETAF_1249-YP_005698855.1--36H-COG1/1-529     SASETAQRGGKVVANVVQTMHDIASSSQKIADITGVIDGIAFQTNILALNAAVEAARAGE
En.638.865-Ent638_0513-YP_001175251.1--36H-COG1/1-586    SASETAQKGGKVVDNVVQTMRDIAGSSQKIADIISVIDGIAFQTNILALNAAVEAARAGE
Ed.ict.1187-NT01EI_1462-YP_002932883.1--36H-COG1/1-532   EASHTASEGGEIVSQVVRTMHNITQSSQKIGDITGVIDGIAFQTNILALNAAVEAARAGE
Ye.ent.378-YE2573-YP_001006777.1--36H-COG1/1-545         DASGTAAKGGELAGSVVTTMHDIATSSQKIGAITSVIDGIAFQTNILALNAAVEAARAGE
Ed.tar.1771-ETAF_1250-YP_005698856.1--36H-COG1/1-530     EASHTAGEGGEIVSQVVRTMHDITQSSQKIGDITGVIDGIAFQTNILALNAAVEAARAGE
Mo.mor.2189-MU9_1763-YP_007505182.1--36H-COG1/1-524      TASKTASRGGQITDSVIETMTSIANSSQKISAIISVIDGIAFQTNILALNAAVEAARAGE
En.aer.1436-EAE_15540-YP_004593298.1--36H-COG1/1-536     DASSTAAKGGELADDVVTTMHDIANSSQKIGAITSVIDGIAFQTNILALNAAVEAARAGE
En.638.865-Ent638_2456-YP_001177176.1--36H-COG1/1-533    QAAHTAKKGGDQASHVATTMQDIATSSQKIGDIISVIDGIAFQTNILALNAAVEAARAGE
Se.pro.864-Spro_2982-YP_001479211.1--36H-COG1/1-541      DASTTAAKGGELAGDVVTTMHDIANSSQKIGAITSVIDGIAFQTNILALNAAVEAARAGE
Es.col.1836-Y75_p1861-YP_490147.1--36H-COG1/1-533        NAATTAQAGGVQVSTMTHTMQEIATSSQKIGDIISVIDGIAFQTNILALNAAVEAARAGE
Serrat.1901-SerAS13_3074-YP_006025913.1--36H-COG1/1-541  DASATAAKGGEMAGDVVTTMQDIANSSQKIGAITSVIDGIAFQTNILALNAAVEAARAGE
Ye.pes.133-YPZ3_2023-YP_003568195.1--36H-COG1/1-536      EASGTAAKGGELTASVVTTMHAIATSSQKIGAITSVIDGIAFQTNILALNAAVEAARAGE
Serrat.1408-SerAS12_3072-YP_004501498.1--36H-COG1/1-541  DASATAAKGGEMAGDVVTTMQDIANSSQKIGAITSVIDGIAFQTNILALNAAVEAARAGE
Se.mar.2260-D781_2758-YP_007345194.1--36H-COG1/1-534     DASAIAAKGGELAGEVVNTMHAIASSSQKIGAITGVIDGIAFQTNILALNAAVEAARAGE
Ye.pse.585-YPTB2400-YP_070914.1--36H-COG1/1-536          EASGTAAKGGELTASVVTTMHAIATSSQKIGAITSVIDGIAFQTNILALNAAVEAARAGE
Se.ply.1407-SerAS9_3071-YP_004506451.1--36H-COG1/1-541   DASATAAKGGEMAGDVVTTMQDIANSSQKIGAITSVIDGIAFQTNILALNAAVEAARAGE
Pr.stu.1965-S70_18140-YP_006218125.1--36H-COG1/1-521     SASKTATKGGDITVDVIDTMTEISHSSQKISAIISVIDGIAFQTNILALNAAVEAARAGE
En.bac.2261-D782_1749-YP_007339930.1--36H-COG1/1-533     NAARTARMGGDQASNVAHTMQSIATSSQKIGDIISVIDGIAFQTNILALNAAVEAARAGE
En.asb.1498-Entas_2590-YP_004829104.1--36H-COG1/1-533    QAAQTAKKGGDQATHVASTMHEIAASSQKIGDIISVIDGIAFQTNILALNAAVEAARAGE
En.asb.1498-Entas_3672-YP_004830170.1--36H-COG1/1-545    VASTTASRGGSLVDDVVTTMSGISDSSKKIAEITNVINSIAFQTNILALNAAVEAARAGE
Ci.kos.578-CKO_01067-YP_001452646.1--36H-COG1/1-537      SAADTAKKGGDQASRMASTMQDIAASSQKIGDIIGVIDSIAFQTNILALNAAVEAARAGE
En.clo.1544-EcWSU1_02810-YP_004952663.1--36H-COG1/1-538  QAAMTAKKGGDQASHVASTMQDIAASSQKIGDIISVIDGIAFQTNILALNAAVEAARAGE
Sa.ent.407-STM3152-NP_462067.1--36H-COG1/1-547           AASLTAGKGGELVSDVVETMNGISASSQQIAEITTVINSIAFQTNILALNAAVEAARAGE
Pe.atr.485-ECA1332-YP_049438.1--36H-COG1/1-555           QASTTAKKGGEIVENVVKTMAEISGSSRKIAEITTVINGIAFQTNILALNAAVEAARAGE
Pe.car.1139-PC1_1208-YP_003016792.1--36H-COG1/1-555      QASTTAKKGGEIVENVVKTMAEISGSSRKIAEITTVINGIAFQTNILALNAAVEAARAGE
En.638.865-Ent638_3407-YP_001178118.1--36H-COG1/1-545    VASSTASRGGSLVQEVVTTMSGISASSKKIAEITTVINGIAFQTNILALNAAVEAARAGE
Pa.vag.184-Pvag_1413-YP_003931052.1--36H-COG1/1-554      TATTTAQRGGQLVSEVVSTMEGISGSSKKIAEITNVINGIAFQTNILALNAAVEAARAGE
Ph.asy.1114-PAU_02684-YP_003041518.1--36H-COG1/1-544     SASKTASKGGELTAGVVETMDEIANSSQKISAIISVIDGIAFQTNILALNAAVEAARAGE
Pa.ana.1905-PAJ_1316-YP_005934192.1--36H-COG1/1-556      AATITAKQGGSLVGEVVTTMNGISDSSKKIAEITNVINSIAFQTNILALNAAVEAARAGE
Ci.kos.578-CKO_04394-YP_001455885.1--36H-COG1/1-561      AASATAGKGGTLVSEVVETMDGISASSRQIAEITNVINSIAFQTNILALNAAVEAARAGE
Sa.bon.1474-SBG_2750-YP_004731563.1--36H-COG1/1-547      AASVTAGKGGELVSDVVETMEGISASSQQIAEITNVINSIAFQTNILALNAAVEAARAGE
Ra.aqu.1678-Q7S_16025-YP_005402985.1--36H-COG1/1-554     EASTTAQKGGNIVADVVSTMKDISGSSKKIAEITSVINSIAFQTNILALNAAVEAARAGE
Rahnel.1320-Rahaq_3177-YP_004213898.1--36H-COG1/1-554    EASTTAQKGGNIVADVVSTMKDISGSSKKIAEITSVINSIAFQTNILALNAAVEAARAGE
Pectob.2320-W5S_3114-YP_006284066.1--36H-COG1/1-555      QASTTAKKGGQIVENVVKTMAEISGSSRKIAEITTVINGIAFQTNILALNAAVEAARAGE
Ye.ent.378-YE2971-YP_001007160.1--36H-COG1/1-552         NASITAKKGGALVADVVHTMDEISASSRKIAEITTVINSIAFQTNILALNAAVEAARAGE
Er.bil.197-EbC_20190-YP_003741400.1--36H-COG1/1-556      NASVTASKGGKLVNDVVTTMQGIAGSSKKIAEITSVINSIAFQTNILALNAAVEAARAGE
Ph.lum.1262-plu1854-NP_929127.1--36H-COG1/1-544          SASKTASKGGELTVDVVETMDAIANSSQKISAIISVIDGIAFQTNILALNAAVEAARAGE
Er.bil.197-EbC_25340-YP_003741912.1--36H-COG1/1-528      EASTTAHKGGQLTGKVVTTMSEIASSSKKIGDITGVIDGIAFQTNILALNAAVEAARAGE
Di.zea.1140-Dd1591_2937-YP_003005238.1--36H-COG1/1-564   QASTTASKGGELVDNVVKTMAEISGSSKKIAEITSVINGIAFQTNILALNAAVEAARAGE
Er.tas.1011-ETA_14670-YP_001907406.1--36H-COG1/1-518     SASGTAHKGGTLTDNVVTTMNGIADSSKKIADITSVIDGIAFQTNILALNAAVEAARAGE
Sa.ent.404-STY4234-NP_458344.1--36H-COG1/1-547           TASETARKGGRVVDNVVSTMNDIAESSEKIVDITSVIDGIAFQTNILALNAAVEAARAGE
Sa.ent.407-STM3577-NP_462478.1--36H-COG1/1-547           TASETARKGGRVVDNVVSTMNDIAESSEKIVDITSVIDGIAFQTNILALNAAVEAARAGE
Sa.bon.1474-SBG_3170-YP_004731982.1--36H-COG1/1-547      TASETARKGGRVVDNVVSTMNDIAESSEKIVDITSVIDGIAFQTNILALNAAVEAARAGE
En.asb.1498-Entas_2591-YP_004829105.1--36H-COG1/1-555    SASDTAQRGGRVVDGVVKTMHEIADSSKKIADIISVIDGIAFQTNILALNAAVEAARAGE
En.638.865-Ent638_1858-YP_001176586.1--36H-COG1/1-552    NASETADRGGMVVDNVIRTMTEIADSSQQIAHITSVIDSIAFQTNILALNAAVEAARAGE
Ci.rod.62-ROD_19331-YP_003365490.1--36H-COG1/1-553       SASETAKHGGKVVDNVVKTMHDIADSSKKIADITSVIDGIAFQTNILALNAAVEAARAGE
Di.dad.235-Dda3937_01559-YP_003882087.1--36H-COG1/1-566  QASTTASKGGDLVENVVKTMAEISGSSKKIAEITSVINGIAFQTNILALNAAVEAARAGE
Er.bil.197-EbC_37780-YP_003743156.1--36H-COG1/1-553      SASETAGKGGKVVDGVVKTMSEIAGSSKKIADITSVIDGIAFQTNILALNAAVEAARAGE
Pantoe.297-Pat9b_2001-YP_004115872.1--36H-COG1/1-558     TATGTAQQGGRLVGEVVTTMAGISGSSKKIAEITNVINSIAFQTNILALNAAVEAARAGE
```

```
En.clo.1544-EcWSU1_01962-YP_004951821.1--36H-COG1/1-553  NASETAARGGHVVDNVVRTMTEIADSSQQIAHITGVIDSIAFQTNILALNAAVEAARAGE
En.asb.1498-Entas_1879-YP_004828403.1--36H-COG1/1-554    NASETAARGGQVVDNVVRTMTEISDSSQQIAHITGVIDSIAFQTNILALNAAVEAARAGE
En.bac.2261-D782_1748-YP_007339929.1--36H-COG1/1-547     SASQTARRGGDVVDNVVSTMHDIAGSSQKIADITSVIDGIAFQTNILALNAAVEAARAGE
Es.col.1836-Y75_p1862-YP_490148.1--36H-COG1/1-553        SASDTAQHGGKVVDGVVKTMHEIADSSKKIADIISVIDGIAFQTNILALNAAVEAARAGE
Cr.tur.6-CTU_33010-YP_003211664.1--36H-COG1/1-555        NASETADKGGRVVDNVVQTMKSIAESSQQIAHITSVIDSIAFQTNILALNAAVEAARAGE
Cr.tur.6-CTU_25780-YP_003210941.1--36H-COG1/1-536        SASDTAQRGGKVVDGVVNTMHDIAASSKKISDITSVIDGIAFQTNILALNAAVEAARAGE
En.clo.1544-EcWSU1_00403-YP_004950264.1--36H-COG1/1-549  DASSTAKKGGNVVEGVVRTMDDIATSSSKIAQITNVIDGIAFQTNILALNAAVEAARAGE
En.638.865-Ent638_2457-YP_001177177.1--36H-COG1/1-555    RASETALHGGKVVDGVVKTMQEITGSSKKIADIISVIDGIAFQTNILALNAAVEAARAGE
Sa.bon.1474-SBG_1755-YP_004730609.1--36H-COG1/1-553      SASDTARHGGKVVDGVVNTMHDIADSSKKIADIISVIDGIAFQTNILALNAAVEAARAGE
Pr.mir.1265-PMI1665-YP_002151396.1--36H-COG1/1-548       SASETALRGGEITHSVVEMMDAITQSSQKIGAIISVIDGIAFQTNILALNAAVEAARAGE
Cr.sak.579-ESA_01348-YP_001437444.1--36H-COG1/1-556      SASETAQRGGKVVDGVVNTMHDIAASSKKISDITSVIDGIAFQTNILALNAAVEAARAGE
Cr.sak.579-ESA_00560-YP_001436686.1--36H-COG1/1-555      NASETADKGGRVVENVVQTMKSIAESSQQIAHITSVIDSIAFQTNILALNAAVEAARAGE
Sa.ent.407-STM1919-NP_460876.1--36H-COG1/1-553           SASETARHGGKVVDGVVNTMHEIADSSKKIADIISVIDGIAFQTNILALNAAVEAARAGE
Ci.kos.578-CKO_01066-YP_001452645.1--36H-COG1/1-552      SASDTAQHGGKVVDGVVNTMHEIADSSKKIADIISVIDGIAFQTNILALNAAVEAARAGE
En.asb.1498-Entas_0424-YP_004826963.1--36H-COG1/1-549    DASSTAKKGGSVVEGVVRTMDEIATSSSKIAQITNVIDGIAFQTNILALNAAVEAARAGE
Pantoe.297-Pat9b_1273-YP_004115150.1--36H-COG1/1-553     TASDTADKGGNVVAGVVKTMHDIADSSKKIADITSVIDGIAFQTNILALNAAVEAARAGE
Ed.tar.1771-ETAF_2264-YP_005699859.1--36H-COG1/1-555     KASTIAVNGGKITRQMVSTMTEIAESSRKIGDITGVIDGIAFQTNILALNAAVEAARAGE
Pa.ana.1905-PAJ_3534-YP_005936409.1--36H-COG1/1-553      DASVMASQGGQIVAKVIANIEQISESSKQIASIIGIIDSIAFQTNILALNAAVEAARAGE
Pa.vag.184-Pvag_1203-YP_003930842.1--36H-COG1/1-552      EASATALQGGETVARVIANMDQISESSNQIAGIIAIIDSIAFQTNILALNAAVEAARAGE
Pantoe.297-Pat9b_1605-YP_004115478.1--36H-COG1/1-556     SASETAQKGGTVVDGVVRTMQDIAGSSKKIADIISVIDGIAFQTNILALNAAVEAARAGE
Cr.sak.579-ESA_00210-YP_001436348.1--36H-COG1/1-557      TASETALKGGEVVDGVVRTMDEIAASSNQIAQITNVIDGIAFQTNILALNAAVEAARAGE
Er.tas.1011-ETA_pET460340-YP_001905943.1--36H-COG1/1-543 NASLTAGKGGVLVTDVITTMEGISDSSKKIADITSVINSIAFQTNILALNAAVEAARAGE
Cr.tur.6-CTU_36610-YP_003212024.1--36H-COG1/1-557        TASETALKGGEVVDGVVRTMDEIAASSNQIAQITNVIDGIAFQTNILALNAAVEAARAGE
Pe.car.1139-PC1_1159-YP_003016743.1--36H-COG1/1-556      NASDTANKGGTVVDNVVKTMNEIADSSQKIAHITSVIDGIAFQTNILALNAAVEAARAGE
Er.tas.1011-ETA_14640-YP_001907403.1--36H-COG1/1-554     TASETAQKGGKVVDGVVKTMSDIAGSSKKIADITSVIDGIAFQTNILALNAAVEAARAGE
Pa.vag.184-Pvag_1725-YP_003931362.1--36H-COG1/1-556      SASETAEKGGSVVAGVVKTMSEIAGSSKKIADITSVIDGIAFQTNILALNAAVEAARAGE
Ed.ict.1187-NT01EI_3742-YP_002935104.1--36H-COG1/1-525   SASATANQGGAVVGQVVTAMDEIADSSRKIASITGVIDSIAFQTNILALNAAVESARAGE
En.638.865-Ent638_0380-YP_001175119.1--36H-COG1/1-549    DASVTAKKGGSVVEGVVRTMDDIATSSSKIAQITNVIDGIAFQTNILALNAAVEAARAGE
Er.bil.197-EbC_25360-YP_003741914.1--36H-COG1/1-556      SASETALKGGKVVDGVVKTMSDIAGSSKKIADITSVIDGIAFQTNILALNAAVEAARAGE
Er.tas.1011-ETA_16910-YP_001907630.1--36H-COG1/1-543     NASLTAGKGGDLVTNVITTMEGISDSSKKIADITSVINSIAFQTNILALNAAVEAARAGE
Es.fer.1173-EFER_3055-YP_002384154.1--36H-COG1/1-603     SASETAQRGGKVVDNVVQTMRDISTSSQKIADIISVIDGIAFQTNILALNAAVEAARAGE
Xe.nem.162-XNC1_1619-YP_003711880.1--36H-COG1/1-520      SASQTASKGGELTTSVVATMDEITNSSKKISAIISVIDGIAFQTNILALNAAVEAARAGE
En.clo.1544-EcWSU1_02811-YP_004952664.1--36H-COG1/1-559  SASETAQRGGRVVDGVVKTMHEIADSSKKIADIISVIDGIAFQTNILALNAAVEAARAGE
Di.dad.235-Dda3937_03500-YP_003882750.1--36H-COG1/1-553  EAQQMARQGGDITDSVVTTMQGIADSSRKIADITSVINGIAFQTNILALNAAVEAARAGE
Xe.bov.105-XBJ1_1924-YP_003467828.1--36H-COG1/1-523      SASQTASKGGELTVSVVATMDEIANSSKKISAIISVIDGIAFQTNILALNAAVEAARAGE
Rahnel.1320-Rahaq_1831-YP_004212576.1--36H-COG1/1-556    DASNTAEKGGELTGNVVKTMNDIAGSSKKISAITSVIDGIAFQTNILALNAAVEAARAGE
Ra.aqu.1678-Q7S_08965-YP_005401598.1--36H-COG1/1-556     DASNTAEKGGELTGNVVKTMNDIAGSSKKISAITSVIDGIAFQTNILALNAAVEAARAGE
Cr.sak.579-ESA_01126-YP_001437230.1--36H-COG1/1-514      AASENAGKGGQLAQNVIETMQGISGSSKKIADITSVINSIAFQTNILALNAAVEAARAGE
Pe.atr.485-ECA2712-YP_050803.1--36H-COG1/1-560           DASVAAKKGGEVTADVMATMASITTSSKKIADITSVINGIAFQTNILALNAAVEAARAGE
En.clo.1544-EcWSU1_00560-YP_004950421.1--36H-COG1/1-597  SASETAQKGGKVVDNVVQTMRDIAGSSQKIADIISVIDGIAFQTNILALNAAVEAARAGE
Di.dad.235-Dda3937_03498-YP_003882752.1--36H-COG1/1-561  EATDAAKQGGKITDNVVETMDSITASSRKIADITSVINGIAFQTNILALNAAVEAARAGE
Cr.tur.6-CTU_27880-YP_003211151.1--36H-COG1/1-514        AASENAGKGGQLAQNVIETMQGISGSSKKIADITSVINSIAFQTNILALNAAVEAARAGE
Pe.car.1139-PC1_1669-YP_003017246.1--36H-COG1/1-560      DASVAAKKGGDVTADVMATMASITTSSKKIADITSVINGIAFQTNILALNAAVEAARAGE
Ra.aqu.1678-Q7S_14450-YP_005402672.1--36H-COG1/1-551     QATQEANQGGVIVNDVISTMVKINASSHKIVEIISVINGIAFQTNILALNAAVEAARAGE
Ci.kos.578-CKO_03622-YP_001455137.1--36H-COG1/1-517      NASETAQKGGNVVDGVVRTMDEIATSSSKIAQITSVIDGIAFQTNILALNAAVEAARAGE
Di.zea.1140-Dd1591_2408-YP_003004728.1--36H-COG1/1-557   EAQKMARQGGDITDSVVTTMQSISESSRRIADITSVINGIAFQTNILALNAAVEAARAGE
Di.zea.1140-Dd1591_2406-YP_003004726.1--36H-COG1/1-559   EASDAARQGGKITDNVVETMDSITASSRKIADITSVINGIAFQTNILALNAAVEAARAGE
Ed.ict.1187-NT01EI_2801-YP_002934203.1--36H-COG1/1-554   KASTIAVNGGEITRQMVSTMTEIAESSRKIGDITGVIDGIAFQTNILALNAAVEAARAGE
Pe.car.1139-PC1_1384-YP_003016966.1--36H-COG1/1-559      DAADMAQKGGTISTNVMATMDDISASSRQISDITSVINSIAFQTNILALNAAVEAARAGE
Rahnel.1320-Rahaq_2866-YP_004213595.1--36H-COG1/1-551    QATQEANQGGVIVNDVISTMVKINASSHKIVEIISVINGIAFQTNILALNAAVEAARAGE
Pe.atr.485-ECA1281-YP_049387.1--36H-COG1/1-556           NASETANKGGTVVDNVVKTMNEISDSSQKIAHITSVIDGIAFQTNILALNAAVEAARAGE
Pectob.2320-W5S_1886-YP_006282848.1--36H-COG1/1-549      DASVAAKKGGDVTADVMATMASITTSSKKIADITSVINGIAFQTNILALNAAVEAARAGE
Pectob.2320-W5S_1696-YP_006282659.1--36H-COG1/1-559      DAADMAQKGGNISTNVMATMDGISASSRHISDITSVINSIAFQTNILALNAAVEAARAGE
Di.zea.1140-Dd1591_2407-YP_003004727.1--36H-COG1/1-555   EAQKMARQGGDITDSVVTTMQSISDSSRKIADITSVINGIAFQTNILALNAAVEAARAGE
Pe.atr.485-ECA1509-YP_049611.1--36H-COG1/1-559           DAADMAQKGGNISTNVMATMDGISASSRQISDITSVINSIAFQTNILALNAAVEAARAGE
Pa.vag.184-Pvag_1724-YP_003931361.1--36H-COG1/1-520      TASETAQQGGKVVNGVVTTMKEIAGSSKKIADIISVIDGIAFQTNILALNAAVEAARAGE
Pa.ana.1905-PAJ_1541-YP_005934417.1--36H-COG1/1-520      TASETALQGGKVVDGVVTTMQEIAGSSKKIADIISVIDGIAFQTNILALNAAVEAARAGE
Mo.mor.2189-MU9_1596-YP_007505015.1--36H-COG1/1-519      VTSSTAHQGHEMIAAVIRTMDEISESSRKIAEITNVINSIAFQTNILALNASIEAARAGV
Pr.mir.1265-PMI2808-YP_002152509.1--36H-COG1/1-563       STADIAVQGGEHSNKMVMTMTDIADRSQKIGEITAVIDDIAFQTNILALNAAVEAARAGE
Di.zea.1140-Dd1591_0395-YP_003002764.1--36H-COG1/1-556   DASTAANNGGGVVQAVSHSMQNITQSSQKIADIINVIDGIAFQTNILALNAAVEAARAGE
En.clo.1544-EcWSU1_03786-YP_004953635.1--36H-COG1/1-515  VASTTASRGGSLVEEVVTTMSGISESSKKIAEITNVINSIAFQTNILALNAAVEAARAGE
Pectob.2320-W5S_3170-YP_006284116.1--36H-COG1/1-556      SASETANKGGVVVNNVVKTMDEISDSSRKIAHITSVIDGIAFQTNILALNAAVEAARAGE
Di.dad.235-Dda3937_02184-YP_003884754.1--36H-COG1/1-556  DASKAANNGGDVVKAVSNSMQNITHSSQKIVDIINVIDSIAFQTNILALNAAVEAARAGE
Ci.rod.62-ROD_32641-YP_003366748.1--36H-COG1/1-517       NASGTAQKGGEVVEDVVRTMNDIAASSSKIAQITSVIDGIAFQTNILALNAAVEAARAGE
Di.dad.235-Dda3937_03499-YP_003882751.1--36H-COG1/1-561  EAQRMARQGGDITDSVVSTMQSISDSSRKIADITSVINGIAFQTNILALNAAVEAARAGE
Di.dad.235-Dda3937_00105-YP_003883346.1--36H-COG1/1-561  DASDTAHKGLQVVDNVVSTMGQISGSSQKISDITGLIDSIAFQTNILALNAAVEAARAGE
Pantoe.297-Pat9b_1604-YP_004115477.1--36H-COG1/1-555     SASETAQKGGTVVDGVVRTMQDIAGSSKKIADITSVIDGIAFQTNILALNAAVEAARAGE
Er.tas.1011-ETA_14660-YP_001907405.1--36H-COG1/1-560     SASETALKGGKVVDGVVKTMSEISTSSKQIADIISVIDGIAFQTNILALNAAVEAARAGE
Pr.mir.1265-PMI2809-YP_002152510.1--36H-COG1/1-575       QTSNIASKGGDVTHRMVKTMNDIAQSSQKIGEITTVINSIAFQTNILSLNAAVEAARAGE
Ed.tar.1771-ETAF_3053-YP_005700648.1--36H-COG1/1-516     SASATADQGGTEVGQVVTTMGEIADSSRKIASITGVIDSIAFQTNILALNAAVEAARAGE
Se.pro.864-Spro_1415-YP_001477647.1--36H-COG1/1-546      RASQSAINGGEITRKMVVTMSDIADSSRKIGDITSVIDGIAFQTNILALNAAVEAARAGE
Di.zea.1140-Dd1591_1804-YP_003004134.1--36H-COG1/1-562   DASDTAHKGLQVVDNVVSTMGKISGSSQKISDITGLIDSIAFQTNILALNAAVEAARAGE
Di.dad.235-Dda3937_03501-YP_003882749.1--36H-COG1/1-575  EAQRMARQGGDITDSVVTTMQGISDSSRKIADITSVINGIAFQTNILALNAAVEAARAGE
Pantoe.297-Pat9b_0851-YP_004114731.1--36H-COG2/1-524     NTSDVAKEGGVVMEQVIEKMEAIALSSKKIVDIISVIDSIAFQTNILSLNAAVEAARAGE
Pa.vag.184-Pvag_0291-YP_003929953.1--36H-COG2/1-525      TTSSVAREGGVVMEKVIEKMEAIALSSRKIVDIISVIDSIAFQTNILSLNAAVEAARAGE
En.clo.1544-EcWSU1_03886-YP_004953735.1--36H-COG2/1-519  DAAMVVKNNGEMMKQVTSKMRLINETSNRMSDIIDLIDAIAFQTNILALNAAVEAARAGE
Cr.tur.6-CTU_04790-YP_003208842.1--36H-COG2/1-515        DAAGVVKRNGEMMQQVTDKMRVIRGTSDRMSDIINLIDSIAFQTNILALNAAVEAARAGE
Ci.kos.578-CKO_04484-YP_001455975.1--36H-COG2/1-521      DAASVVKNNGEMMRQVTQKMRVINETSNRMSDIINLIDSIAFQTNILALNAAVEAARAGE
Cr.sak.579-ESA_03488-YP_001439539.1--36H-COG2/1-515      EAADVVKRNGEMMQQVTDKMRVIRGTSDRMSDIINLIDSIAFQTNILALNAAVEAARAGE
Ci.rod.62-ROD_48071-YP_003368199.1--36H-COG2/1-521       EAATVVKNNGGMMNQVTQKMRVINETSQRMTDIINLIDAIAFQTNILALNAAVEAARAGE
Sa.bon.1474-SBG_2851-YP_004731664.1--36H-COG2/1-521      GAASVVKNNGEMMNQVTQKMRVINDTANRMSDIINIIDSIAFQTNILALNAAVEAARAGE
Sa.ent.407-STM3216-NP_462130.1--36H-COG2/1-521           GAASVVKNNGEMMNQVTQKMRVINDTANRMSDIINIIDSIAFQTNILALNAAVEAARAGE
En.asb.1498-Entas_3753-YP_004830248.1--36H-COG2/1-519    DAATVVKNNGEMMKQVTSKMRLINETSNRMSDIIDLIDAIAFQTNILALNAAVEAARAGE
Mo.mor.2189-MU9_3389-YP_007506806.1--36H-COG2/1-521      DTAGVVKHNGEMMEQVTDKIRMINTTATRMSDIINLIDSIAFQTNILALNAAVEAARAGE
Sa.ent.404-STY3394-NP_457606.1--36H-COG2/1-522           GAASVVKNNGEMMNQVTQKMRVINDTANRMSDIINIIDSIAFQTNILALNAAVEAARAGE
En.638.865-Ent638_3526-YP_001178235.1--36H-COG2/1-519    DAATVVKSNGEMMKQVTQKMRLINETSNRMSDIINLIDAIAFQTNILALNAAVEAARAGE
Pe.atr.485-ECA1774-YP_049874.1--36H-COG2/1-554           SASETVKKSGAMMETVTQEMRGIRDSSKRMAEIIGVIDGIAFQTNILALNAAVEAARAGE
En.638.865-Ent638_2119-YP_001176845.1--36H-COG2/1-549    ETGQVVKINSDRMHEVSAAMEEIHKGAEKMGDIIAVIEGIAFQTNILALNAAVEAARAGE
```

```
Pectob.2320-W5S_2813-YP_006283770.1--36H-COG2/1-554     GASATVKKSGAMMETVTQEMRGIRDSSQRMAEIIGVIDGIAFQTNILALNAAVEAARAGE
Di.zea.1140-Dd1591_0695-YP_003003055.1--36H-COG2/1-561  QASSAAHRSGEVMLSVTSKMRGIRESSMRMAEIIGVIDGIAFQTNILALNAAVEAARAGE
Pa.vag.184-Pvag_pPag30079-YP_003729820.1--36H-COG2/1-546 NVREVARQGNEVTADISGTMKSIGESSGRIADITSVIDSIAFQTNILALNAAVEAARAGE
Er.bil.197-EbC_38940-YP_003743272.1--36H-COG2/1-526     LASKVASQSGDAVKQVVSTMEMINGSSRKIVDIISVIDGIAFQTNILALNAAVEAARAGE
Ra.aqu.1678-Q7S_24891-YP_005419232.1--36H-COG2/1-554    QASRVAVQGGEVVGEVVNTMEGINTSSRKIVDIIAVIDGIAFQTNILALNAAVEAARAGE
En.asb.1498-Entas_2287-YP_004828805.1--36H-COG2/1-513   EASQTATHGGRVMRDVVNTMNDINTSSQKIADITAVINSIAFQTNILALNAAVEAARAGE
Rahnel.1320-Rahaq_4869-YP_004215574.1--36H-COG2/1-554   QASRVAVQGGEVVGEVVNTMEGINTSSRKIVDIIAVIDGIAFQTNILALNAAVEAARAGE
Di.dad.235-Dda3937_00027-YP_003883574.1--36H-COG2/1-554 QASDIVRQSGEMMADVTREMREIRSASQRMAEIIGVIDSIAFQTNILALNAAVEAARAGE
Pr.stu.1965-S70_05790-YP_006215722.1--36H-COG2/1-520    DTAVVVANNGRMMNEVTNKIRAIHSSSNQMTEIINLIDSIAFQTNILALNASVEAARAGE
En.638.865-Ent638_2100-YP_001176826.1--36H-COG2/1-512   EASQTATQGGRVMRDVVSTMSDINTSSKKIADITAVINSIAFQTNILALNAAVEAARAGE
Pe.car.1139-PC1_2526-YP_003018093.1--36H-COG2/1-554     SASETVKKSGAMMETVTQEMRGIRDSSQRMAEIIGVIDGIAFQTNILALNAAVEAARAGE
Mo.mor.2189-MU9_702-YP_007504121.1--36H-COG2/1-525      EASETARHGGEKVDLVIHAMQDISNSSRTITDIIGVIDSIAFQTNILALNAAVEAARAGE
Pe.car.1139-PC1_3443-YP_003018995.1--36H-COG2/1-511     DASDTANLGGEQVSKAVNTMHGIAQSSHRIAEITSMINSIAFQTNILALNAAVEAARAGE
Pa.ana.1905-PAJ_0915-YP_005933791.1--36H-COG2/1-559     SVRDVANKGNEATTAITGTMKSISESSGRIAEITAVIDGIAFQTNILALNAAVEAARAGE
Pectob.2320-W5S_3736-YP_006284671.1--36H-COG2/1-510     DASATANQGGEQVSKAVNTMHGIAQSSHRIAEITSMINSIAFQTNILALNAAVEAARAGE
Pe.atr.485-ECA0183-YP_048310.1--36H-COG2/1-553          KASSAAKQSGDVMVSVTQKMRGIRDSSQRMAEIIGVIDGIAFQTNILALNAAVEAARAGE
Rahnel.1320-Rahaq_3107-YP_004213828.1--36H-COG2/1-503   DAANTAARGGKLVADVVGVMKEITVSSKQISEITTVINSIAFQTNILALNAAVEAARAGE
Pe.car.1139-PC1_4071-YP_003019622.1--36H-COG2/1-553     KASGAAKQSGDVMVSVTQKMRGIRDSSQRMAEIIGVIDGIAFQTNILALNAAVEAARAGE
Di.dad.235-Dda3937_03462-YP_003884507.1--36H-COG2/1-563 QASSAAHRSGEVMLSVTTKMRGIREASMRMAEIIGVIDGIAFQTNILALNAAVEAARAGE
Ra.aqu.1678-Q7S_15665-YP_005402913.1--36H-COG2/1-503    DAANTAARGGKLVADVVGVMKEITVSSKQISEITTVINSIAFQTNILALNAAVEAARAGE
Pectob.2320-W5S_4505-YP_006285423.1--36H-COG2/1-555     KASGAAKQSGDVMVSVTQKMRGIRDSSQRMAEIIGVIDGIAFQTNILALNAAVEAARAGE
Pe.atr.485-ECA3642-YP_051730.1--36H-COG2/1-551          STTQQAQSGGQLVTEVVETMGAIDSSSKKIVDIIGVIDSIAFQTNILALNAAVEAARAGE
Pe.car.1139-PC1_1417-YP_003016999.1--36H-COG2/1-554     NASTVAQQGGDVVNEVVDTMRAINESSRSIADIINVIDSIAFQTNILALNAAVEAARAGE
En.asb.1498-Entas_4509-YP_004821569.1--36H-COG2/1-559   DSSDIRKRSSEMMDNTTHQMEGIHNSTKKMSDIISVIESIAFQTNILALNAAVEAARAGE
Pa.ana.1905-PAJ_3064-YP_005935940.1--36H-COG2/1-544     NVRNVANEGNDATAAITSTMRTIKDSSGKIADITAVIDSIAFQTNILALNAAVEAARAGE
Pectob.2320-W5S_1733-YP_006282696.1--36H-COG2/1-535     NASTVALQGGNVVNDVVDTMKAINDSSRSIVDIINVIDSIAFQTNILALNAAVEAARAGE
Pe.atr.485-ECA4334-YP_052421.1--36H-COG2/1-556          SASTTAQKGGDAVSNVVRTMEDITASSRKIGDIIGVIDGIAFQTNILALNAAVEAARAGE
Di.zea.1140-Dd1591_1602-YP_003003935.1--36H-COG2/1-554  EASDIVRQSGEMMANVTREMREIRNASQRMAEIIGVIDSIAFQTNILALNAAVEAARAGE
Pectob.2320-W5S_4506-YP_006285424.1--36H-COG2/1-540     KATGAAKQSGEVMLSVTQKMRGIHDSSQRMAEIIGVIDGIAFQTNILALNAAVEAARAGE
Di.zea.1140-Dd1591_1453-YP_003003794.1--36H-COG2/1-532  TASQVAEQGGSVVTQVVDTMRDINDSAQRIADIIGVIDGIAFQTNILALNAAVEAARAGE
Pectob.2320-W5S_0106-YP_006281117.1--36H-COG2/1-556     SASTTAQKGGDAVNNVVRTMEDITVSSRKIGDIIGVIDSIAFQTNILALNAAVEAARAGE
Di.zea.1140-Dd1591_0769-YP_003003127.1--36H-COG2/1-505  NASSTARQGGEQVSSAVQTMGNIAQGSRRIAEITSVINSIAFQTNILALNAAVEAARAGE
Ra.aqu.1678-Q7S_18035-YP_005403387.1--36H-COG2/1-535    SASQVAQRGGNVVERVVSTMHEIAESSGKVTQIITVIEGIAFQTNILALNAAVEAARAGE
Rahnel.1320-Rahaq_3579-YP_004214298.1--36H-COG2/1-535   SASQVAQRGGNVVERVVSTMHEIAESSGKVTQIITVIEGIAFQTNILALNAAVEAARAGE
Pe.car.1139-PC1_4072-YP_003019623.1--36H-COG2/1-540     KATGAAKQSGDVMLSVTQKMRGIRDSSQRMAEIIGVIDGIAFQTNILALNAAVEAARAGE
Pe.car.1139-PC1_3464-YP_003019016.1--36H-COG2/1-551     STTQQAQSGGQLVTEVVETMGAIDSSSKKIVDIIGVIDSIAFQTNILALNAAVEAARAGE
Pe.car.1139-PC1_0111-YP_003015709.1--36H-COG2/1-556     SASTTAQKGGDAVNNVVRTMEDITASSRKIGDIIGVIDSIAFQTNILALNAAVEAARAGE
Pe.atr.485-ECA0182-YP_048309.1--36H-COG2/1-542          KATGAAKQSGDVMVSVTQKMRGIRDSSQRMAEIIGVIDGIAFQTNILALNAAVEAARAGE
Pectob.2320-W5S_3753-YP_006284688.1--36H-COG2/1-551     STTQQAQSGGQLVTEVVKTMGAIDSSSKKIVDIIGVIDSIAFQTNILALNAAVEAARAGE
Pa.ana.1905-PAJ_2666-YP_005935542.1--36H-COG2/1-514     EAATLARTGESEVQLMSHTMNEIASSATKVRDITGVIESIAFQTNILALNAAVEAARAGE
Ci.rod.62-ROD_16161-YP_003365194.1--36H-COG6/1-557      DASGKASRGGQMVSGVVQTMGNISASSKKISEITAVINSIAFQTNILALNAAVEAARAGE
En.asb.1498-Entas_2095-YP_004828614.1--36H-COG6/1-563   DASGKASRGGQMVSGVVKTMGNISTSSKKISEITAVINSIAFQTNILALNAAVEAARAGE
En.638.865-Ent638_1961-YP_001176688.1--36H-COG6/1-563   DASGKASRGGQMVSGVVKTMSNISTSSKKISEITAVINSIAFQTNILALNAAVEAARAGE
Cr.sak.579-ESA_01710-YP_001437800.1--36H-COG6/1-565     DASGKATTGGQIVSGVVTTMGNISSSSKKISEITAVINSIAFQTNILALNAAVEAARAGE
Cr.tur.6-CTU_22440-YP_003210607.1--36H-COG6/1-565       DASGKATTGGQIVSGVVTTMGNISSSSKKISEITAVINSIAFQTNILALNAAVEAARAGE
Ci.kos.578-CKO_01456-YP_001453025.1--36H-COG6/1-562     DASGKASHGGQMVSGVVKTMGNISNSSKKISEITAVINSIAFQTNILALNAAVEAARAGE
Rahnel.1320-Rahaq_1930-YP_004212674.1--36H-COG6/1-566   DASNKAQKGGNIVAEVVSTMDKISRSSTKIAEITNVINSIAFQTNILALNAAVEAARAGE
Pantoe.297-Pat9b_2403-YP_004116261.1--36H-COG6/1-564    DASGKARNGGEIVAGVVKTMNNISGSSKKIAEITNVINSIAFQTNILALNAAVEAARAGE
Er.bil.197-EbC_26700-YP_003742048.1--36H-COG6/1-567     DASGKASQGGEIVSGVISTMNNISTSSKKIAEITTVINSIAFQTNILALNAAVEAARAGE
Pectob.2320-W5S_1760-YP_006282723.1--36H-COG6/1-570     NASGKAKQGGEIVANVVNTMNNISGSSKKISEITSVINSIAFQTNILALNAAVEAARAGE
Pa.ana.1905-PAJ_1621-YP_005934497.1--36H-COG6/1-568     DASGKARSGGDMVSGVVKTMNDISGSSKKIAEITNVINSIAFQTNILALNAAVEAARAGE
En.clo.1544-EcWSU1_02120-YP_004951978.1--36H-COG6/1-576 DASGKASRGGQMVSGVVKTMSNISTSSKKISEITAVINSIAFQTNILALNAAVEAARAGE
Ra.aqu.1678-Q7S_09815-YP_005401764.1--36H-COG6/1-566    DASNKAQKGGNIVAEVVSTMDKISRSSTKIAEITNVINSIAFQTNILALNAAVEAARAGE
Ye.pse.585-YPTB2412-YP_070925.1--36H-COG6/1-579         NASGKAAQGGDIVSDVVSTMDKISLSSMKIAEITNVINSIAFQTNILALNAAVEAARAGE
Ye.ent.378-YE2588-YP_001006789.1--36H-COG6/1-579        NASGKAAQGGDIVNDVVSTMDKISLSSMKIAEITNVINSIAFQTNILALNAAVEAARAGE
Di.dad.235-Dda3937_02787-YP_003883649.1--36H-COG6/1-572 NASGKAKQGGDIVENVVNTMNSISGSSRKISEITNVINSIAFQTNILALNAAVEAARAGE
Pa.vag.184-Pvag_1800-YP_003931435.1--36H-COG6/1-568     DASGKARSGGDLVNSVVKTMTNISGSSKKIAEITNVINSIAFQTNILALNAAVEAARAGE
En.bac.2261-D782_2307-YP_007340470.1--36H-COG6/1-558    DASGKATRGGQLVTGVVATMGNISTSSKKISEITAVINSIAFQTNILALNAAVEAARAGE
Pe.atr.485-ECA1683-YP_049784.1--36H-COG6/1-573          NASGKAKQGGEIVANVVNTMNSISGSSKKISEITSVINSIAFQTNILALNAAVEAARAGE
Pe.car.1139-PC1_2617-YP_003018183.1--36H-COG6/1-573     NASGKAKQGGEIVANVVNTMNSISGSSKKISEITNVINSIAFQTNILALNAAVEAARAGE
Di.zea.1140-Dd1591_1535-YP_003003868.1--36H-COG6/1-574  NASGKAKQGGEIVENVVHTMNSISGSSRKISEITNVINSIAFQTNILALNAAVEAARAGE
Di.zea.1140-Dd1591_3580-YP_003005868.1--36H-COG6/1-572  NASGKARQGGELVGGVVKTMGNIHQSSKKISEITNIINGIAFQTNILALNAAVEAARAGE
Es.col.1836-Y75_p1397-YP_489687.1--36H-COG6/1-546       EASIKASDGGQTVSGVVKTMGAISTSSKKISEITAVINSIAFQTNILALNAAVEAARAGE
Pe.atr.485-ECA3902-YP_051990.1--36H-COG6/1-575          NASEKAERGGQIVQGVVDTMQDISTSSKRISEITSVINSIAFQTNILALNAAVEAARAGE
Sa.bon.1474-SBG_1454-YP_004730321.1--36H-COG6/1-537     DASGKASRGGQIVSGVVQTMGNISISSKKISEITAVINSIAFQTNILALNAAVEAARAGE
Sa.ent.407-STM1626-NP_460585.1--36H-COG6/1-541          DASGKASRGGQMVSGVVQTMGNISTSSKKISEITAVINSIAFQTNILALNAAVEAARAGE
Pe.car.1139-PC1_3679-YP_003019230.1--36H-COG6/1-567     NASEKAEKGGQIVQGVVDTMQDISTSSKRISEITSVINSIAFQTNILALNAAVEAARAGE
Pectob.2320-W5S_4020-YP_006284955.1--36H-COG6/1-575     NASEKAGKGGQIVQGVVDTMHDISTSSKRISEITSVINSIAFQTNILALNAAVEAARAGE
Di.zea.1140-Dd1591_0523-YP_003002884.1--36H-COG6/1-568  NASSKAEQGGSIVQDVVKTMGDISSSSKKISEITSVINSIAFQTNILALNAAVEAARAGE
Di.dad.235-Dda3937_02665-YP_003881279.1--36H-COG6/1-572 NASGKARQGGELVDSVVKTMGNIHQSSKKISEITNIINGIAFQTNILALNAAVEAARAGE
```

Pr.mir.1265-PMI1666-YP_002151397.1--36H-COG1/1-568	HGRGFAVVAGEVRNLAQRSAEAAKEIKTLIEDSVSRTETGSVLVESAGETMTRIVESVTR
Di.dad.235-Dda3937_02779-YP_003883640.1--36H-COG1/1-566	QGRGFAVVAGEVRNLAQRSAQAAKEIKSLIEDSVNRVEEGSVLVESAGETMGEIVGAVTR
Di.zea.1140-Dd1591_1542-YP_003003875.1--36H-COG1/1-566	QGRGFAVVAGEVRNLAQRSAQAAKEIKSLIEDSVNRVQEGSVLVESAGETMGEIVGAVTR
Pe.atr.485-ECA1691-YP_049792.1--36H-COG1/1-561	QGRGFAVVAGEVRNLAQRSAQAAKEIKSLIEDSVSRVDEGSVLVESAGETMGEIVSAVTR
Se.pro.864-Spro_2983-YP_001479212.1--36H-COG1/1-556	QGRGFAVVAGEVRNLAQRSAQAAKEIKGLIEDSVNRVDMGSVLVESAGETMGDIVNAVTR
Ph.asy.1114-PAU_02685-YP_003041519.1--36H-COG1/1-564	HGRGFAVVAGEVRNLAQRSAEAAKEIKALIEDSVSRADTGSVLVESAGETMNNIVSSVTR
Pr.stu.1965-S70_18135-YP_006218124.1--36H-COG1/1-561	HGRGFAVVADEVRNLAQRSANAAKEIKALIEDSVSRTDTGARQAEDAGETMTKIVESVTR
Mo.mor.2189-MU9_1762-YP_007505181.1--36H-COG1/1-561	HGRGFAVVADEVRNLAQRSADAAKEIKILIEDSVSRTGTGARQAEDAGETMTKIVESVTR
Pe.car.1139-PC1_2609-YP_003018175.1--36H-COG1/1-561	QGRGFAVVAGEVRNLAQRSAQAAKEIKSLIEDSVSRVDEGSVLVESAGETMGEIVSAVTR
Xe.nem.162-XNC1_1620-YP_003711881.1--36H-COG1/1-567	HGRGFAVVAGEVRNLAQRSAEAAKEIKALIEDSVNRTDTGSMQVESAGETMNKIVDSVTR
Pectob.2320-W5S_1768-YP_006282731.1--36H-COG1/1-561	QGRGFAVVAGEVRNLAQRSAQAAKEIKSLIDDSVGRVDEGSVLVESAGETMGEIVSAVTR
Pa.vag.184-Pvag_1723-YP_003931360.1--36H-COG1/1-558	QGRGFAVVAGEVRSLAQRSAQAAKEIKGLIEDSVSRVNTGSVLVESAGDTMSNIVSAVTR
Ye.pes.133-YPZ3_2024-YP_003568196.1--36H-COG1/1-557	QGRGFAVVAGEVRNLAQRSAQAAKEIKGLIDDSVNRVDLGSVLVESAGETMGDIVNAVTR
Ye.pse.585-YPTB2401-YP_070915.1--36H-COG1/1-557	QGRGFAVVAGEVRNLAQRSAQAAKEIKGLIDDSVNRVDLGSVLVESAGETMGDIVNAVTR
Ye.ent.378-YE2575-YP_001006778.1--36H-COG1/1-557	QGRGFAVVAGEVRNLAQRSAQAAKEIKGLIEDSVNRVDMGSVLVESAGETMGDIVNAVTR
Pa.ana.1905-PAJ_1540-YP_005934416.1--36H-COG1/1-563	QGRGFAVVAGEVRNLAQRSAQAAKEIKGLIEDSVGRVNTGSVLVESAGDTMNEIVSAVTR
Ph.lum.1262-plu1853-NP_929126.1--36H-COG1/1-564	HGRGFAVVAGEVRNLAQRSAEAAKEIKALIEDSVSRADTGSVLVESAGETMNNIVSSVTR
Serrat.1901-SerAS13_3075-YP_006025914.1--36H-COG1/1-555	QGRGFAVVAGEVRNLAQRSAQAAKEIKGLIEDSVSRVDMGSVLVESAGETMGDIVNAVTR
Sa.ent.404-STY2128-NP_456485.1--36H-COG1/1-553	QGRGFAVVAGEVRNLASRSAQAAKEIKALIEDSVSRVDTGSVLVESAGETMTDIVNAVTR
Ra.aqu.1678-Q7S_08960-YP_005401597.1--36H-COG1/1-564	QGRGFAVVAGEVRTLAQRSAQAAKEIKGLIDDSVSRINTGSVLAESAGETMHDMVNAVTR
Se.mar.2260-D781_2759-YP_007345195.1--36H-COG1/1-555	QGRGFAVVAGEVRNLAQRSAQAAKEIKGLIDDSVSRVDMGSVLVESAGETMGDIVNAVTR
Rahnel.1320-Rahaq_1830-YP_004212575.1--36H-COG1/1-564	QGRGFAVVAGEVRTLAQRSAQAAKEIKGLIDDSVSRINTGSVLAESAGETMHDMVNAVTR
Se.ply.1407-SerAS9_3072-YP_004506452.1--36H-COG1/1-555	QGRGFAVVAGEVRNLAQRSAQAAKEIKGLIEDSVSRVDMGSVLVESAGETMGDIVNAVTR
Ed.ict.1187-NT01EI_1461-YP_002932882.2--36H-COG1/1-555	QGRGFAVVAGEVRNLAQRSAQAAKEIKGLIEDSVSKVDTGSVLVESAGDTMGDIVNAVTR
En.aer.1436-EAE_15535-YP_004593297.1--36H-COG1/1-556	QGRGFAVVAGEVRNLAQRSAQAAKEIKALIEDSVNRVDMGSVLVESAGDTMGDIVNAVTR
Xe.bov.105-XBJ1_1923-YP_003467827.1--36H-COG1/1-567	HGRGFAVVAGEVRNLAQRSAEAAKEIKSLIEDSVDRTDTGSVLVESAGETMNKIVDSVTR
Serrat.1408-SerAS12_3073-YP_004501499.1--36H-COG1/1-555	QGRGFAVVAGEVRNLAQRSAQAAKEIKGLIEDSVSRVDMGSVLVESAGETMGDIVNAVTR
Er.bil.197-EbC_25350-YP_003741913.1--36H-COG1/1-559	QGRGFAVVAGEVRNLAQRSAQAAKEIKGLIEDSVARVDTGSVLVESAGETMADIVSAVTR
Er.tas.1011-ETA_14650-YP_001907404.1--36H-COG1/1-554	QGRGFAVVAGEVRNLAQRSAQAAREIKGLIENSVARVDTGSALVESAGETMSDIVNAVTR
Sa.ent.407-STM4533-NP_463392.1--36H-COG1/1-553	QGRGFAVVAGEVRNLAQRSAQAAREIKSLIEDSVSRVDVGSTLVESAGETMDEIVNAVTR
Es.col.1836-Y75_p4240-YP_492486.1--36H-COG1/1-551	QGRGFAVVAGEVRNLAQRSAQAAREIKSLIEDSVGKVDVGSTLVESAGETMAEIVSAVTR
En.bac.2261-D782_3913-YP_007342002.1--36H-COG1/1-553	QGRGFAVVAGEVRNLAQRSAQAAREIKSLIEDSVSRVDLGSTLVESAGETMDEIVNAVTR
Ci.kos.578-CKO_03442-YP_001454958.1--36H-COG1/1-554	QGRGFAVVAGEVRNLAQRSAQAAREIKSLIEDSVSRVDVGSTLVESAGETMDEIVNAVTR
Cr.sak.579-ESA_03402-YP_001439456.1--36H-COG1/1-555	QGRGFAVVAGEVRNLAQRSAQAAKEIKGLIEDSVSKVDVGSTLVESAGETMGEIVSAVTR
Pantoe.297-Pat9b_1606-YP_004115479.1--36H-COG1/1-558	QGRGFAVVAGEVRSLAQRSAQAAKEIKGLIEDSVSRVDTGSVLVESAGETMTNIVNAVTR
Cr.tur.6-CTU_05670-YP_003208930.1--36H-COG1/1-555	QGRGFAVVAGEVRNLAQRSAQAAKEIKGLIEDSVSKVDVGSTLVESAGETMGEIVSAVTR
Sa.bon.1474-SBG_3944-YP_004732724.1--36H-COG1/1-553	QGRGFAVVAGEVRNLAQRSAQAAREIKSLIEDSVSRVDVGSTLVESAGETMDEIVNAVTR
En.asb.1498-Entas_0547-YP_004827084.1--36H-COG1/1-554	QGRGFAVVAGEVRNLAQRSAQAAREIKSLIEDSVGRVEVGSTLVESAGETMGEIVNAVTR
Ci.rod.62-ROD_48461-YP_003368233.1--36H-COG1/1-554	QGRGFAVVAGEVRNLAQRSAQAAREIKSLIEDSVNRVDAGSTLVESAGETMDEIVSAVTR
Ed.tar.1771-ETAF_1249-YP_005698855.1--36H-COG1/1-529	QGRGFAVVAGEVRNLAQRSAQAAKEIKGLIEDSVSKVDTGSVLVESAGDTMGDIVNAVTR
En.638.865-Ent638_0513-YP_001175251.1--36H-COG1/1-586	QGRGFAVVAGEVRNLAQRSAQAAREIKSLIEDSVGRVELGSTLVESAGETMGEIVNAVTR
Ed.ict.1187-NT01EI_1462-YP_002932883.1--36H-COG1/1-532	QGRGFAVVAGEVRNLAQRSAQAAKEIKGLIEESVSRVKQGSTQVEHAGNTMDNVVRSVVR
Ye.ent.378-YE2573-YP_001006777.1--36H-COG1/1-545	QGRGFAVVAGEVRNLAQRSAQAAKEIKGLIDESVSRVRQGSTLVENAGTTMEEIVRSVTR
Ed.tar.1771-ETAF_1250-YP_005698856.1--36H-COG1/1-530	QGRGFAVVAGEVRNLAQRSAQAAKEIKGLIDESVSRVKQGSTLVESAGNTMDNVVRSVVR
Mo.mor.2189-MU9_1763-YP_007505182.1--36H-COG1/1-524	QGRGFSVVAGEVRALAQRSADAAKEIKSLIDESVDRVGQGTQLVDSAGKTMEELVTAVNQ
En.aer.1436-EAE_15540-YP_004593298.1--36H-COG1/1-536	QGRGFAVVAGEVRNLASRSAQAAKEIKLLIDESVSRVKHGSVLVENSGATMQDIVRSVTR
En.638.865-Ent638_2456-YP_001177176.1--36H-COG1/1-533	QGRGFAVVAGEVRNLASRSANAAKEIKVLIEESVSRVQQGSGLVDTAARTMTEIVTSVTR
Se.pro.864-Spro_2982-YP_001479211.1--36H-COG1/1-541	QGRGFAVVAGEVRNLAQRSAQAAKEIKGLIDESVSRVKQGSVLVENSGATMQDIVRSVTR
Es.col.1836-Y75_p1861-YP_490147.1--36H-COG1/1-533	QGRGFAVVAGEVRNLASRSAQAAKEIKGLIEESVNRVQQGSKLVNNAAATMIDIVSSVTR
Serrat.1901-SerAS13_3074-YP_006025913.1--36H-COG1/1-541	QGRGFAVVAGEVRNLASRSAQAAKEIKGLIDESVSRVKQGSVLVENSGATMQDIVRSVTR
Ye.pes.133-YPZ3_2023-YP_003568195.1--36H-COG1/1-536	QGRGFAVVAGEVRNLAQRSAQAAKEIKGLIDESVSRVRQGSTLVESAGTTMEEIVRSVAR
Serrat.1408-SerAS12_3072-YP_004501498.1--36H-COG1/1-541	QGRGFAVVAGEVRNLASRSAQAAKEIKGLIDESVSRVKQGSVLVENSGATMQDIVRSVTR
Se.mar.2260-D781_2758-YP_007345194.1--36H-COG1/1-534	QGRGFAVVAGEVRSLAQRSAQAAKEIKGLIDESVGRVRQGSTLVEHSGDTMQDIVRSVTR
Ye.pse.585-YPTB2400-YP_070914.1--36H-COG1/1-536	QGRGFAVVAGEVRNLAQRSAQAAKEIKGLIDESVSRVRQGSTLVESAGTTMEEIVRSVAR
Se.ply.1407-SerAS9_3071-YP_004506451.1--36H-COG1/1-541	QGRGFAVVAGEVRNLASRSAQAAKEIKGLIDESVSRVKQGSVLVENSGATMQDIVRSVTR
Pr.stu.1965-S70_18140-YP_006218125.1--36H-COG1/1-521	QGRGFSVVAGEVRALAQRSAEAAKEIKTLIDESVGRVNQGSQLVNEAGQTMNELVAAVNQ
En.bac.2261-D782_1749-YP_007339930.1--36H-COG1/1-533	QGRGFAVVAGEVRNLASRSAQAAKEIKVLIEESVARVQEGSALVDTAAKTMGEIVHSVTQ
En.asb.1498-Entas_2590-YP_004829104.1--36H-COG1/1-533	QGRGFAVVAGEVRNLASRSANAAKEIKVLIEESVSRVEQGSALVDTAAKTMSEIVTSVTR
En.asb.1498-Entas_3672-YP_004830170.1--36H-COG1/1-545	QGRGFAVVAGEVRNLASRSANAAKEIEGLIADSVNRVEQGAQLVNDTGTTMEAILRAVTE
Ci.kos.578-CKO_01067-YP_001452646.1--36H-COG1/1-537	QGRGFAVVAGEVRNLASRSAQAAKEIKGLIEESVNRVHQGSQLVDTAANTMTEIVSSVTQ
En.clo.1544-EcWSU1_02810-YP_004952663.1--36H-COG1/1-538	QGRGFAVVAGEVRNLASRSANAAKEIKGLIEESVSRVQQGSALVDTAAKTMHEIVTSVTR
Sa.ent.407-STM3152-NP_462067.1--36H-COG1/1-547	QGRGFAVVAGEVRNLASRSAGAAKEIEALIGESVRRVAQGAQLVQETGATMDAILRGVTE
Pe.atr.485-ECA1332-YP_049438.1--36H-COG1/1-555	QGRGFAVVAGEVRSLAQRSAQAAKEIEGLISESVRCVDTGSNLVSDAGDTMQDIVRAVTN
Pe.car.1139-PC1_1208-YP_003016792.1--36H-COG1/1-555	QGRGFAVVAGEVRSLAQRSAQAAKEIEGLISESVRCVDTGSNLVSDAGDTMQDIVRAVTN
En.638.865-Ent638_3407-YP_001178118.1--36H-COG1/1-545	QGRGFAVVAGEVRNLASRSADAAKEIEGLIADSVSRVERGAKIVDDTGTTMEAILRAVTE
Pa.vag.184-Pvag_1413-YP_003931052.1--36H-COG1/1-554	QGRGFAVVASEVRNLAQRSANAAKEIATLIEDSVQRVGKGAELVSTTGGTMNNILKSVQD
Ph.asy.1114-PAU_02684-YP_003041518.1--36H-COG1/1-544	QGRGFSVVAGEVRDLAQRSAEAAKEIKTLISESVQRVSQGSELVSHAGKTMNELVTSVNQ
Pa.ana.1905-PAJ_1316-YP_005934192.1--36H-COG1/1-556	QGRGFAVVASEVRNLAQRSAGAAKEIAGLIADSVERVEKGAQLVSTTGGTMSGILKSVQD
Ci.kos.578-CKO_04394-YP_001455885.1--36H-COG1/1-561	QGRGFAVVAGEVRNLASRSAGAAKEIETLIAESVRRVEQGAQLVQETGATMEAILRGVTE
Sa.bon.1474-SBG_2750-YP_004731563.1--36H-COG1/1-547	QGRGFAVVAGEVRNLASRSAGAAKEIEALIGESVRRVEQGAQLVKETGATMDAILRGVTE
Ra.aqu.1678-Q7S_16025-YP_005402985.1--36H-COG1/1-554	QGRGFAVVASEVRNLAQRSAQAAKEIEQLISESVSRVDSGSKLVENAGQTMNEVVRSITH
Rahnel.1320-Rahaq_3177-YP_004213898.1--36H-COG1/1-554	QGRGFAVVASEVRNLAQRSAQAAKEIEQLISESVSRVDSGSKLVENAGQTMNEVVRSITH
Pectob.2320-W5S_3114-YP_006284066.1--36H-COG1/1-555	QGRGFAVVAGEVRSLAQRSAQAAKEIEGLISESVRCVDTGSNLVSDAGDTMQDIVRAVTN
Ye.ent.378-YE2971-YP_001007160.1--36H-COG1/1-552	QGRGFAVVASEVRNLAQRSAQAAKEIDSLITESVNRVSSGSALVESAGITMDEIVRSITN
Er.bil.197-EbC_20190-YP_003741400.1--36H-COG1/1-556	QGRGFAVVASEVRNLAQRSAGAAKEIEMLIADSVSRVEKGSLLVGEAGSTMDAILSGVVE
Ph.lum.1262-plu1854-NP_929127.1--36H-COG1/1-544	QGRGFSVVAGEVRDLAQRSAEAAKEIKTLISESVQRVSQGSELVSHAGKTMNELVVSVNQ
Er.bil.197-EbC_25340-YP_003741912.1--36H-COG1/1-528	QGRGFAVVAGEVRNLAQRSAQAAKEIKALIEASVDRVDQGAVLVASAGETMDEIVQSVTR
Di.zea.1140-Dd1591_2937-YP_003005238.1--36H-COG1/1-564	QGRGFAVVAGEVRSLAQRSAQAAKEIENLIAESVRCVDNGSALVADAGSTMSEIVKAVTN
Er.tas.1011-ETA_14670-YP_001907406.1--36H-COG1/1-518	QGRGFAVVAGEVRSLAQRSAGAAKEIKTLIEESVSRVDQGAGLVASAGDTMSEIVQSVNR
Sa.ent.404-STY4234-NP_458344.1--36H-COG1/1-547	QGRGFAVVAGEVRTLASRSAQAAKEIKVLIENSVSRIDTGSTQVREAGETMKEIVNAVTR
Sa.ent.407-STM3577-NP_462478.1--36H-COG1/1-547	QGRGFAVVAGEVRTLASRSAQAAKEIKVLIENSVSRIDTGSTQVREAGETMKEIVNAVTR
Sa.bon.1474-SBG_3170-YP_004731982.1--36H-COG1/1-547	QGRGFAVVAGEVRTLASRSAQAAKEIKVLIENSVSRIDTGSTQVREAGETMKEIVTAVTR
En.asb.1498-Entas_2591-YP_004829105.1--36H-COG1/1-555	QGRGFAVVAGEVRNLASRSANAAKEIKALIEDSVSRVDTGSVLVESAGETMNDIVNAVTR
En.638.865-Ent638_1858-YP_001176586.1--36H-COG1/1-552	QGRGFAVVAGEVRTLASRSAQAAKEIKGLIENSVSRVNTGSGQVSEAGKTMKEIVAAVTR
Ci.rod.62-ROD_19331-YP_003365490.1--36H-COG1/1-553	QGRGFAVVAGEVRNLASRSAEAAKEIKALIDDSVSRVDTGSALVESAGDTMHDIVDAVTR
Di.dad.235-Dda3937_01559-YP_003882087.1--36H-COG1/1-566	QGRGFAVVAGEVRNLAQRSAQAAKEIENLIAESVRCVDNGSMLVADAGSTMSEIVTAVTN
Er.bil.197-EbC_37780-YP_003743156.1--36H-COG1/1-553	QGRGFAVVAGEVRNLAQRSAQAAKEIKTLIEDSVNRVNAGSLQVETAGETMHDIVNAVVR
Pantoe.297-Pat9b_2001-YP_004115872.1--36H-COG1/1-558	QGRGFAVVASEVRNLAQRSANAAKEIATLIEDSVQRVEKGSDLVNSAGHTMNDILKSVQD

```
En.clo.1544-EcWSU1_01962-YP_004951821.1--36H-COG1/1-553  QGRGFAVVAGEVRTLASRSAQAAKEIKGLIENSVSRVNTGSEQVSEAGATMKEIVAAVTR
En.asb.1498-Entas_1879-YP_004828403.1--36H-COG1/1-554    QGRGFAVVAGEVRTLASRSAQAAKEIKGLIENSVSRVNTGSEQVSEAGETMREIVAAVTR
En.bac.2261-D782_1748-YP_007339929.1--36H-COG1/1-547     QGRGFAVVAGEVRNLASRSAQAAKEIKALIDNSVERVDAGSRLVESAGETMHEIVSAVTR
Es.col.1836-Y75_p1862-YP_490148.1--36H-COG1/1-553        QGRGFAVVAGEVRNLASRSAQAAKEIKALIEDSVSRVDTGSVLVESAGETMNNIVNAVTR
Cr.tur.6-CTU_33010-YP_003211664.1--36H-COG1/1-555        QGRGFAVVAGEVRTLASRSANAAKEIKALIENSVNRVDTGSGQVQEAGDTMKEIVQAVTR
Cr.tur.6-CTU_25780-YP_003210941.1--36H-COG1/1-536        QGRGFAVVAGEVRNLASRSAQAAKEIKALIEDSVSRVDSGSVLVESAGETMQEIVGAVTR
En.clo.1544-EcWSU1_00403-YP_004950264.1--36H-COG1/1-549  QGRGFAVVAGEVRTLAQRSAQAAKEIKALIDDSGERVNAGSQLVNEAGATMAEIVNAVTR
En.638.865-Ent638_2457-YP_001177177.1--36H-COG1/1-555    QGRGFAVVAGEVRNLASRSANAAKEIKTLIDDSVSRVNTGSVLVESAGETMTDIVNAVTR
Sa.bon.1474-SBG_1755-YP_004730609.1--36H-COG1/1-553      QGRGFAVVAGEVRNLASRSAQAAKEIKALIEDSVSRVDTGSVLVESAGETMTDIVNAVTR
Pr.mir.1265-PMI1665-YP_002151396.1--36H-COG1/1-548       QGRGFSVVAGEVRNLAQRSAEAAKEIKTLIDESVSRVSQGSRLVNDAGQTMEELVTSVNK
Cr.sak.579-ESA_01348-YP_001437444.1--36H-COG1/1-556      QGRGFAVVAGEVRNLASRSAQAAKEIKALIEDSVSRVDSGSVLVESAGETMQEIVGAVTR
Cr.sak.579-ESA_00560-YP_001436686.1--36H-COG1/1-555      QGRGFAVVAGEVRTLASRSANAAKEIKALIENSVNRVDTGSGQVQEAGDTMKEIVQAVTR
Sa.ent.407-STM1919-NP_460876.1--36H-COG1/1-553           QGRGFAVVAGEVRNLASRSAQAAKEIKALIEDSVSRVDTGSVLVESAGETMTDIVNAVTR
Ci.kos.578-CKO_01066-YP_001452645.1--36H-COG1/1-552      QGRGFAVVAGEVRNLASRSAQAAKEIKALIEDSVSRVDTGSVLVESAGETMRDIVNAVTR
En.asb.1498-Entas_0424-YP_004826963.1--36H-COG1/1-549    QGRGFAVVAGEVRTLAQRSAQAAKEIKALIDDSGERVSAGSQLVNEAGATMAEIVNAVTR
Pantoe.297-Pat9b_1273-YP_004115150.1--36H-COG1/1-553     QGRGFAVVAGEVRNLAQRSAQAAKEIKLLIENSVQRVNLGSQQVGSAGDTMQEIVSAVTR
Ed.tar.1771-ETAF_2264-YP_005699859.1--36H-COG1/1-555     QGRGFAVVAGEVRNLAQRSAQAAKEIKGLIDTSVTRVDQGNQLVENVSHAMEEIVTSVNH
Pa.ana.1905-PAJ_3534-YP_005936409.1--36H-COG1/1-553      QGRGFAVVAGEVRSLAGRSASAAQEIRTLIDQSTQRVNSGSTHASEAGDVMGSIVNSVNS
Pa.vag.184-Pvag_1203-YP_003930842.1--36H-COG1/1-552      QGRGFAVVAGEVRSLAGRSASAAQEIRGLIDRSAERIKTGAGHAAQAGTAMESIVKSVSR
Pantoe.297-Pat9b_1605-YP_004115478.1--36H-COG1/1-556     QGRGFAVVAGEVRSLAQRSAQAAKEIKGLIEDSVNRVNTGSELVGTAGETMSDIVNAVTR
Cr.sak.579-ESA_00210-YP_001436348.1--36H-COG1/1-557      QGRGFAVVAGEVRTLASRSAQAAKEIKALIENSGNRVDAGSQLVREAGETMKEVVGAVTR
Er.tas.1011-ETA_pET460340-YP_001905943.1--36H-COG1/1-543 QGRGFAVVASEVRSLAQRSAGAAKEIETLIADSVSRVNNGSKLVAKAGSNMKDIVQSVNE
Cr.tur.6-CTU_36610-YP_003212024.1--36H-COG1/1-557        QGRGFAVVAGEVRTLASRSAQAAKEIKALIENSGNRVDAGSQLVREAGETMKEVVGAVTR
Pe.car.1139-PC1_1159-YP_003016743.1--36H-COG1/1-556      QGRGFAVVAGEVRTLAQRSAQAAKEIKTLIDDSVSRTNTGSVQVKDAGDTMKEIVSAVSR
Er.tas.1011-ETA_14640-YP_001907403.1--36H-COG1/1-554     QGRGFAVVAGEVRNLAQRSAQAAKEIKGLIEDSVARVDSGSALVGTAGETMTDIVNAVTR
Pa.vag.184-Pvag_1725-YP_003931362.1--36H-COG1/1-556      QGRGFAVVAGEVRSLAQRSAQAAKEIKGLIDDSVNRVSVGSQLVGTAGETMSDIVSAVTR
Ed.ict.1187-NT01EI_3742-YP_002935104.1--36H-COG1/1-525   QGRGFAVVAGEVRSLAQRSAQAAREIRTLIEDSVRRIDTGSQQATQAGETMQSVVGAAQR
En.638.865-Ent638_0380-YP_001175119.1--36H-COG1/1-549    QGRGFAVVAGEVRTLAQRSAQAAKEIKALIDDSGERVSAGSQLVNEAGSTMAEIVSAVTR
Er.bil.197-EbC_25360-YP_003741914.1--36H-COG1/1-556      QGRGFAVVAGEVRNLAQRSAQAAKEIKGLIEDSVARVDSGTVLVGTAGETMADIVSAVTR
Er.tas.1011-ETA_16910-YP_001907630.1--36H-COG1/1-543     QGRGFAVVASEVRSLAQRSAGAAKEIETLIADSVSRVNNGSKLVAQAGSNMKDIVQSVNE
Es.fer.1173-EFER_3055-YP_002384154.1--36H-COG1/1-603     QGRGFAVVAGEVRTLASRSAQAAREIKSLIEDSVSRVDTGSTLVESAGETMAEIVNAVTR
Xe.nem.162-XNC1_1619-YP_003711880.1--36H-COG1/1-520      QGRGFSVVAGEVRDLAQRSAEAAKEIKTLIDESVHRVNQGSELVNNAGQTMDELVRSVNR
En.clo.1544-EcWSU1_02811-YP_004952664.1--36H-COG1/1-559  QGRGFAVVAGEVRNLASRSANAAKEIKALIEDSVSRVDTGSVLVESAGETMNDIVNAVTR
Di.dad.235-Dda3937_03500-YP_003882750.1--36H-COG1/1-553  QGRGFAVVAGEVRNLAQRSAQAAKEIETLIGESVSRVSTGSELVREAGNAMEVIISSVSR
Xe.bov.105-XBJ1_1924-YP_003467828.1--36H-COG1/1-523      QGRGFSVVAGEVRDLAQRSAEAAKEIKTLIDESVHRVNQGSELVNHAGNTMDELVKSVNQ
Rahnel.1320-Rahaq_1831-YP_004212576.1--36H-COG1/1-556    QGRGFAVVAGEVRTLAQRSAQAAKEIKALIDESVSRVSQGSQLVESAGNTMDDIVRAVTR
Ra.aqu.1678-Q7S_08965-YP_005401598.1--36H-COG1/1-556     QGRGFAVVAGEVRTLAQRSAQAAKEIKALIDESVSRVSQGSQLVESAGNTMDDIVRAVTR
Cr.sak.579-ESA_01126-YP_001437230.1--36H-COG1/1-514      QGRGFAVVAGEVRNLASRSAEAAREIETLITESVERIEKGSELVNAAGDSMAEIYRGVSN
Pe.atr.485-ECA2712-YP_050803.1--36H-COG1/1-560           QGRGFAVVAGEVRNLAQRSAQAAKEIESLIAESVQRVNMGSNQVTQTGEAMDSIISAITR
En.clo.1544-EcWSU1_00560-YP_004950421.1--36H-COG1/1-597  QGRGFAVVAGEVRNLAQRSAQAAREIKSLIEDSVGRVEVGSTLVESAGETMGEIVNAVTR
Di.dad.235-Dda3937_03498-YP_003882752.1--36H-COG1/1-561  QGRGFAVVAGEVRSLAQRSAQAAKEIEGLIAESVSRVDIGSSQVKQAGEAMGTIITAVSH
Cr.tur.6-CTU_27880-YP_003211151.1--36H-COG1/1-514        QGRGFAVVAGEVRNLASRSAEAAREIETLITESVERIDKGSELVNAAGDSMAEIYRGVSN
Pe.car.1139-PC1_1669-YP_003017246.1--36H-COG1/1-560      QGRGFAVVAGEVRNLAQRSAQAAKEIESLITESVERINTGSNQVTQTGEAMDSIISAITR
Ra.aqu.1678-Q7S_14450-YP_005402672.1--36H-COG1/1-551     QGRGFAVVASEVRNLAQRSGQAAKEIGVLINESVENIQSGSEQVTQAGGAMEKIVSSVSR
Ci.kos.578-CKO_03622-YP_001455137.1--36H-COG1/1-517      QGRGFAVVAGEVRTLAQRSAQAAKEIKTLIDDSGNRVSAGSALVHEAGETMAEIVSAVTR
Di.zea.1140-Dd1591_2408-YP_003004728.1--36H-COG1/1-557   QGRGFAVVAGEVRNLAQRSAQAAKEIETLIGESVSRVDTGSALVREAGSAMEVIISSVSR
Di.zea.1140-Dd1591_2406-YP_003004726.1--36H-COG1/1-559   QGRGFAVVAGEVRSLAQRSAQAAKEIEGLIAESVSRVDVGSTQVKQAGDAMQTIIDVVSH
Ed.ict.1187-NT01EI_2801-YP_002934203.1--36H-COG1/1-554   QGRGFAVVAGEVRNLAQRSAQAAKEIKGLIDTSVTRVDQGNQLVENVSHAMEDIVTSVNH
Pe.car.1139-PC1_1384-YP_003016966.1--36H-COG1/1-559      QGRGFAVVAGEVRTLAQRSAQAAKEIEALIAESVSRVETGAGQVRQSGEAMTAIISSISH
Rahnel.1320-Rahaq_2866-YP_004213595.1--36H-COG1/1-551    QGRGFAVVASEVRNLAQRSGQAAKEIGVLINESVENIQSGSEQVTQAGGAMEKIVSSVSR
Pe.atr.485-ECA1281-YP_049387.1--36H-COG1/1-556           QGRGFAVVAGEVRTLAQRSAQAAKEIKSLIDDSVSRANTGSLQVKDAGDTMKEIVSAVSR
Pectob.2320-W5S_1886-YP_006282848.1--36H-COG1/1-549      QGRGFAVVAGEVRNLAQRSAQAAKEIESLITESVQRINVGSSQVTQTGEAMDSIISAITR
Pectob.2320-W5S_1696-YP_006282659.1--36H-COG1/1-559      QGRGFAVVAGEVRTLAQRSAQAAKEIEALIAESVSRVETGAGQVKQSGEAMSAIIVSIAH
Di.zea.1140-Dd1591_2407-YP_003004727.1--36H-COG1/1-555   QGRGFAVVAGEVRSLAQRSAQAAKEIETLIGESVSRVDTGSALVREAGSAMEVIISSVSR
Pe.atr.485-ECA1509-YP_049611.1--36H-COG1/1-559           QGRGFAVVAGEVRTLAQRSAQAAKEIEALIAESVSRVETGAGQVRQSGEAMTAIIASIAH
Pa.vag.184-Pvag_1724-YP_003931361.1--36H-COG1/1-520      QGRGFAVVAGEVRSLAQRSAQAAKEIKGLIEDSVSRVNSGSLQVESAGSTMNEIVGAVTR
Pa.ana.1905-PAJ_1541-YP_005934417.1--36H-COG1/1-520      QGRGFAVVAGEVRSLAQRSAQAAKEIKGLIEDSVARVNSGSLQVESAGSTMNNIVGAVTR
Mo.mor.2189-MU9_1596-YP_007505015.1--36H-COG1/1-519      HGRGFMVVAGEVRTLASNSADAAKEIERLIANAVSRVDQGIKLVAGGGETMEEIVRASGE
Pr.mir.1265-PMI2808-YP_002152509.1--36H-COG1/1-563       QGRGFSVVASEVRNLAQRSAEAAKEIKELIESTILRVKQGNDLVEQVSLSMGEIVTSVNH
Di.zea.1140-Dd1591_0395-YP_003002764.1--36H-COG1/1-556   AGRGFAVVASEVRSLAQRSAQAAKEIKTLIDESVDNVKNGYEQVSLASKSMEDILKSVTN
En.clo.1544-EcWSU1_03786-YP_004953635.1--36H-COG1/1-515  QGRGFAVVAGEVRNLASRSANAAKEIEGLITDSVSRVEQGAKLVGDTGTTMDAILRDVTE
Pectob.2320-W5S_3170-YP_006284116.1--36H-COG1/1-556      QGRGFAVVAGEVRTLAQRSAQAAKEIKVLIDDSVSRSNTGSLQVKDAGETMKEIVSAVSR
Di.dad.235-Dda3937_02184-YP_003884754.1--36H-COG1/1-556  AGRGFAVVASEVRNLAQRSAQAAKEIKTLIDESVDNVKNGYEQVSLASKSMEDILKSVTN
Ci.rod.62-ROD_32641-YP_003366748.1--36H-COG1/1-517       QGRGFAVVAGEVRTLASRSAQAAKEIKALIDDSGERVSAGSALVHEAGETMSEIVSAVTR
Di.dad.235-Dda3937_03499-YP_003882751.1--36H-COG1/1-561  QGRGFAVVAGEVRNLAQRSAQAAKEIESLISESVGRVSTGSELVREAGSAMEVIISSVSR
Di.dad.235-Dda3937_00105-YP_003883346.1--36H-COG1/1-561  QGRGFAVVAGEVRNLAQRSADAAREIKELIEDSVNRVHEGSVLVGSAGETMGDLVKAVTR
Pantoe.297-Pat9b_1604-YP_004115477.1--36H-COG1/1-555     QGRGFAVVAGEVRSLAQRSAQAAKEIKGLIEDSVNRVNTGSELVGTAGETMSDIVNAVTR
Er.tas.1011-ETA_14660-YP_001907405.1--36H-COG1/1-560     QGRGFAVVAGEVRNLAQRSAQAAREIKGLIENSVARVDTGSVLVESAGETMSDIVNAVTR
Pr.mir.1265-PMI2809-YP_002152510.1--36H-COG1/1-575       QGRGFSVVATEVRELAQRSAEAAKEIKELIDASINRVKQGNQLVEQVSTSMDEILTSVKH
Ed.tar.1771-ETAF_3053-YP_005700648.1--36H-COG1/1-516     QGRGFAVVAGEVRSLAQRSAQAAREIRTLIEDSVRRIDTGSQQATQAGETMQSVVGAAQR
Se.pro.864-Spro_1415-YP_001477647.1--36H-COG1/1-546      QGRGFAVVAGEVRNLAQRSAQAAKEIKTLIDTSVLRVDQGNDLVENVSGAMTEIVTAIGQ
Di.zea.1140-Dd1591_1804-YP_003004134.1--36H-COG1/1-562   QGRGFAVVAGEVRTLAQRSADAAREIKGLIEDSVSRVQEGSVLVASAGETMGDLVKAVTR
Di.dad.235-Dda3937_03501-YP_003882749.1--36H-COG1/1-575  QGRGFAVVAGEVRNLAQRSAQAAKEIETLIGESVSRVSTGSELVREAGNAMEVIISSVSR
Pantoe.297-Pat9b_0851-YP_004114731.1--36H-COG2/1-524     QGRGFAVVASEVRNLAQRSASAAKEIKVLIEDSVAKVDEGSRLVSHAGSTIGEVVNSVKS
Pa.vag.184-Pvag_0291-YP_003929953.1--36H-COG2/1-525      QGRGFAVVASEVRNLAQRSASAAKEIKVLIEDSVAKVDEGSKLVTHAGSTIGEVVNSVQS
En.clo.1544-EcWSU1_03886-YP_004953735.1--36H-COG2/1-519  HGRGFAVVAGEVRQLAQKSASSASEIRQLIESSTSQTQDGMNLVEKASELINGMVGNVEE
Cr.tur.6-CTU_04790-YP_003208842.1--36H-COG2/1-515        HGRGFAVVAGEVRQLAQKSASSASEIRALIEDSTGQTREGMELVEKASGLLHGMVANVSE
Ci.kos.578-CKO_04484-YP_001455975.1--36H-COG2/1-521      HGRGFAVVAGEVRQLAQKSASSASEIRHLIEDSTSQTQEGMQLVEKASALINGMVDNVEE
Cr.sak.579-ESA_03488-YP_001439539.1--36H-COG2/1-515      HGRGFAVVAGEVRQLAQKSASSASEIRALIEDSTGQTREGMELVEKASGLLNGMVANVGE
Ci.rod.62-ROD_48071-YP_003368199.1--36H-COG2/1-521       HGRGFAVVAGEVRQLAQKSASSASEIRKLIEDSASQTQEGMQLVEKANGLISGMVSNVDE
Sa.bon.1474-SBG_2851-YP_004731664.1--36H-COG2/1-521      HGRGFAVVAGEVRQLAQKSASSASEIRNLIEDSTSQTQEGMQLVEKASDLINGMVDNVEE
Sa.ent.407-STM3216-NP_462130.1--36H-COG2/1-521           HGRGFAVVAGEVRQLAQKSASSASEIRNLIEDSTSQTQEGMHLVEKASALINGMVDNVEE
En.asb.1498-Entas_3753-YP_004830248.1--36H-COG2/1-519    HGRGFAVVAGEVRQLAQKSASSASEIRQLIESSTSQTQDGMNLVEKASGLINGMVGNVEE
Mo.mor.2189-MU9_3389-YP_007506806.1--36H-COG2/1-521      HGRGFAVVAGEVRLLAQRSADSAGEIRTLIENSAGQTREGLQLVEEAAESLRGIVSNVSQ
Sa.ent.404-STY3394-NP_457606.1--36H-COG2/1-522           HGRGFAVVAGEVRQLAQKSASSASEIRNLIEDSTSQTQEGMHLVEKASALINGMVDNVEE
En.638.865-Ent638_3526-YP_001178235.1--36H-COG2/1-519    HGRGFAVVAGEVRQLAQKSASSASEIRQLIESSSSQTQDGMSLVEKASALINGMVGNVEG
Pe.atr.485-ECA1774-YP_049874.1--36H-COG2/1-554           QGRGFAVVASEVRALAQRSATAAKEIKELIDDSFKKVQDGMGLVEETGVTMNSLVTNVQG
En.638.865-Ent638_2119-YP_001176845.1--36H-COG2/1-549    QGRGFAVVAGEVRTLAQRSSASAKEIRDIISASVSKIADGRGLVSQADNGMQEIVDTVSS
```

```
Pectob.2320-W5S_2813-YP_006283770.1--36H-COG2/1-554      QGRGFAVVATEVRALAQRSATAAKEIKELIDDSFKKVQDGMGLVEETGVTMNSLVTNVQG
Di.zea.1140-Dd1591_0695-YP_003003055.1--36H-COG2/1-561   QGRGFAVVAGEVRSLAQRSATAAKEIKELIDDSVDKIQEGMDLVDSAESTMNNLTNHVND
Pa.vag.184-Pvag_pPag30079-YP_003729820.1--36H-COG2/1-546 QGRGFAVVAGEVRTLASRSATAAGEIRQLIEANSASVSAGSILANRSVTTMADIVSSVGQ
Er.bil.197-EbC_38940-YP_003743272.1--36H-COG2/1-526      QGRGFAVVASEVRSLAQRSASAAKEIAQLIEDSVSKVDEGGKQVAKAGTTMEEVLSSVKS
Ra.aqu.1678-Q7S_24891-YP_005419232.1--36H-COG2/1-554     QGRGFAVVASEVRNLAQRSASAAKEIKVLIDDSVAKVDNGTQLVAKAGATMAEVVSSVKN
En.asb.1498-Entas_2287-YP_004828805.1--36H-COG2/1-513    QGRGFAVVASEVRSLSQRSSQAAKDIELLISESVNRITTGSDLVVKAGQTMEQVVESVTR
Rahnel.1320-Rahaq_4869-YP_004215574.1--36H-COG2/1-554    QGRGFAVVASEVRNLAQRSASAAKEIKVLIDDSVAKVDNGTQLVAKAGATMAEVVSSVKN
Di.dad.235-Dda3937_00027-YP_003883574.1--36H-COG2/1-554  QGRGFAVVASEVRALAQRSANSAKEIKELIDNSVQKVQEGMSLVENTEQTMVAVIDNAHN
Pr.stu.1965-S70_05790-YP_006215722.1--36H-COG2/1-520     HGRGFAVVAGEVRLLAQKSANSANDIRLLIENSLTRTKEGMELVEHVEQQIDGMIKNVEE
En.638.865-Ent638_2100-YP_001176826.1--36H-COG2/1-512    QGRGFAVVASEVRSLSQRSSQAAKDIELLISESVSRITTGSDLVTKAGQTMEQVVHSVTR
Pe.car.1139-PC1_2526-YP_003018093.1--36H-COG2/1-554      QGRGFAVVASEVRALAQRSATAAKEIKELIDDSFKKVQDGMGLVEETGVTMNSLVTNVQG
Mo.mor.2189-MU9_702-YP_007504121.1--36H-COG2/1-525       HGRGFAVVSGEVRTLAQRSAQAAGEIKNLINTSVEQVDAGSKLVAEAGESMQVIVTQTQG
Pe.car.1139-PC1_3443-YP_003018995.1--36H-COG2/1-511      QGRGFAVVASEVRNLAQRSAQAAKEIDTLIGESVSQINNGAALVNGAGKTMEGIVQSVTQ
Pa.ana.1905-PAJ_0915-YP_005933791.1--36H-COG2/1-559      HGRGFAVVASEVRSLAQRSASAAGEIRQLIAANADTVGQGNALVIHASETMSGILSAVGK
Pectob.2320-W5S_3736-YP_006284671.1--36H-COG2/1-510      QGRGFAVVASEVRNLAQRSAQAAKEIDTLIGESVSQINNGAALVNSAGKTMEGIVESVTQ
Pe.atr.485-ECA0183-YP_048310.1--36H-COG2/1-553           QGRGFAVVAGEVRSLAQRSATAAREIKDLIDDSVSKIQEGMSLVDNAEETMSGLTGYVRD
Rahnel.1320-Rahaq_3107-YP_004213828.1--36H-COG2/1-503    QGRGFAVVASEVRSLAQRSGQAAKEIEGLIGKSVINVNNGSVLVGKAGDTMEEIVGSVRH
Pe.car.1139-PC1_4071-YP_003019622.1--36H-COG2/1-553      QGRGFAVVAGEVRSLAQRSATAAREIKDLIDDSVSKIQEGMSLVDTAEETMGGLTGYVRD
Di.dad.235-Dda3937_03462-YP_003884507.1--36H-COG2/1-563  QGRGFAVVAGEVRSLAQRSATAAKEIKELIDDSVDKIQEGMQLVDTAESTMDGLTGHVKD
Ra.aqu.1678-Q7S_15665-YP_005402913.1--36H-COG2/1-503     QGRGFAVVASEVRSLAQRSGQAAKEIEGLIGKSVINVNNGSVLVGKAGDTMEEIVGSVRH
Pectob.2320-W5S_4505-YP_006285423.1--36H-COG2/1-555      QGRGFAVVAGEVRSLAQRSATAAREIKDLIDDSVNKIQEGMSLVDTAEETMGGLTGYVRD
Pe.atr.485-ECA3642-YP_051730.1--36H-COG2/1-551           QGRGFAVVASEVRSLAQRSASAAKEIKELIDRSVQTVEAGNRLVVQAGVSIQDIVNGVRK
Pe.car.1139-PC1_1417-YP_003016999.1--36H-COG2/1-554      QGRGFAVVAGEVRNLAQRSAEAAKEIKNLITASVERVELGSVLVNKAGDTMQQVVMSIRQ
En.asb.1498-Entas_4509-YP_004821569.1--36H-COG2/1-559    SGRGFAVVAGEVRSLAQKSATAAKDIKVLIEESIVQTEVGRKLIISAASVMNEMTQNALS
Pa.ana.1905-PAJ_3064-YP_005935940.1--36H-COG2/1-544      HGKGFAVVASEVRTLAQRSSTAAGEIRQLIDANARSVGQGDELVAHATATMQSIVTAVGH
Pectob.2320-W5S_1733-YP_006282696.1--36H-COG2/1-535      QGRGFAVVAGEVRNLAQRSAEAAKEIKNLITASVERTERGSVLVNKAGETMQQVVESIRQ
Pe.atr.485-ECA4334-YP_052421.1--36H-COG2/1-556           QGRGFAVVAGEVRSLAQRSASAAREIKDLISVSVANVEMGEKQVNDAGITIKEIVEKSQH
Di.zea.1140-Dd1591_1602-YP_003003935.1--36H-COG2/1-554   QGRGFAVVASEVRALAQRSANSAKEIKELIDNSVQKVQEGMSLVESTEQTMVAVIDNANN
Pectob.2320-W5S_4506-YP_006285424.1--36H-COG2/1-540      QGRGFAVVAGEVRSLAQRSATAAREIKALIDDSVGKIREGMELVDTAEETMGGLTAHVRD
Di.zea.1140-Dd1591_1453-YP_003003794.1--36H-COG2/1-532   QGRGFAVVAGEVRALAQRSAESAREIKTLISNSVERIESGSALADNAGHTMQDVVNSIRR
Pectob.2320-W5S_0106-YP_006281117.1--36H-COG2/1-556      QGRGFAVVASEVRSLAQRSASAAREIKDLISVSVANVEMGEKLVNDAGITIKDIVEKSQH
Di.zea.1140-Dd1591_0769-YP_003003127.1--36H-COG2/1-505   QGRGFAVVAGEVRNLAQRSAQAAHEIEDLIATSVSQINNGASLVENAGKTMEDIVHAVTQ
Ra.aqu.1678-Q7S_18035-YP_005403387.1--36H-COG2/1-535     QGRGFAVVAGEVRTLAQRSANAAKEIKNLISESVERINVGSKLVDEAGTTMMEVVNAVKG
Rahnel.1320-Rahaq_3579-YP_004214298.1--36H-COG2/1-535    QGRGFAVVAGEVRTLAQRSANAAKEIKNLISESVERINVGSKLVDEAGTTMMEVVNAVKG
Pe.car.1139-PC1_4072-YP_003019623.1--36H-COG2/1-540      QGRGFAVVAGEVRSLAQRSATAAREIKDLIDDSVGKIREGMDLVDTAEETMGGLTAHVKD
Pe.car.1139-PC1_3464-YP_003019016.1--36H-COG2/1-551      QGRGFAVVASEVRSLAQRSASAAKEIKELIDRSVQTVEAGNRLVVQAGASIQDIVNGVRK
Pe.car.1139-PC1_0111-YP_003015709.1--36H-COG2/1-556      QGRGFAVVAGEVRSLAQRSASAAREIKDLINASVANVEMGEKMVNDAGITIKDIVEKSQH
Pe.atr.485-ECA0182-YP_048309.1--36H-COG2/1-542           QGRGFAVVAGEVRSLAQRSATAAREIKDLIDDSVSKIREGMELVDTAEETMGGLTAHVKD
Pectob.2320-W5S_3753-YP_006284688.1--36H-COG2/1-551      QGRGFAVVASEVRSLAQRSASAAKEIKELIDRSVQTVEAGNRLVVQAGASIQDIVSGVRK
Pa.ana.1905-PAJ_2666-YP_005935542.1--36H-COG2/1-514      DGRGFAVVAGEVRTLAQRSATAAKEIKELIERAVEQVDSGVHVAAGTGESILKVVGMVNE
Ci.rod.62-ROD_16161-YP_003365194.1--36H-COG6/1-557       QGRGFAVVASEVRTLASRSAQAAKEIEGLISESVRLIDQGSGEVVAAGNTMTDIVDAVKR
En.asb.1498-Entas_2095-YP_004828614.1--36H-COG6/1-563    QGRGFAVVASEVRTLASRSANAAKEIESLINESVSLIDQGSGEVVAAGNTMNEIVEAVKR
En.638.865-Ent638_1961-YP_001176688.1--36H-COG6/1-563    QGRGFAVVASEVRTLASRSANAAKEIEGLINESVTLIDQGSGEVVAAGNTMNDIVEAVKR
Cr.sak.579-ESA_01710-YP_001437800.1--36H-COG6/1-565      QGRGFAVVASEVRTLASRSAQAAKEIEGLISESVALIDRGSSEVVNAGDTMKGIVEAVKR
Cr.tur.6-CTU_22440-YP_003210607.1--36H-COG6/1-565        QGRGFAVVASEVRTLASRSAQAAKEIEGLISESVALIDRGSSEVVNAGDTMKGIVEAVKR
Ci.kos.578-CKO_01456-YP_001453025.1--36H-COG6/1-562      QGRGFAVVASEVRTLASRSAQAAKEIEGLISESVRLIDQGSGEVVAAGNTMNDIVDAVRR
Rahnel.1320-Rahaq_1930-YP_004212674.1--36H-COG6/1-566    QGRGFAVVASEVRSLAQRSAQAAKEIEALIEESVDYVGEGSILVGDAGDTMSEIVTAVSH
Pantoe.297-Pat9b_2403-YP_004116261.1--36H-COG6/1-564     QGRGFAVVASEVRSLAQRSAQAAKEIESLIAESVDLISNGSHQVGAAGNTMSEIVEAVRR
Er.bil.197-EbC_26700-YP_003742048.1--36H-COG6/1-567      QGRGFAVVASEVRNLAQRSAGAAKEIEGLISESVELIGKGSSQVSHAGDTMTDIVGAVRR
Pectob.2320-W5S_1760-YP_006282723.1--36H-COG6/1-570      QGRGFAVVASEVRSLAQRSAQAAKEIETLISESVKLVNSGSVLVDNAGQTMKEIVDAVTN
Pa.ana.1905-PAJ_1621-YP_005934497.1--36H-COG6/1-568      QGRGFAVVASEVRNLAQRSAQAAKEIESLIAESVKLVSSGSHQVGEAGTTMSEIVEAVRR
En.clo.1544-EcWSU1_02120-YP_004951978.1--36H-COG6/1-576  QGRGFAVVASEVRTLASRSANAAKEIESLINESVSLIDQGSGEVVAAGNTMNDIVDAVKR
Ra.aqu.1678-Q7S_09815-YP_005401764.1--36H-COG6/1-566     QGRGFAVVASEVRSLAQRSAQAAKEIEALIEESVDYVGEGSILVGDAGDTMSEIVTAVSH
Ye.pse.585-YPTB2412-YP_070925.1--36H-COG6/1-579         QGRGFAVVASEVRHLAQRSADAAKEIESLIEASVDLIGDGSILVSNAGKTMNEIVTAVTH
Ye.ent.378-YE2588-YP_001006789.1--36H-COG6/1-579        QGRGFAVVASEVRNLAQRSADAAKEIESLIEVSVDLIGDGSILVSDAGKAMNEIVTAVTH
Di.dad.235-Dda3937_02787-YP_003883649.1--36H-COG6/1-572  QGRGFAVVAGEVRSLAQRSAQAAKEIEGLISESVSLVHSGSELVDKAGQTMHEIVQAVSS
Pa.vag.184-Pvag_1800-YP_003931435.1--36H-COG6/1-568      QGRGFAVVASEVRNLAQRSAQAAKEIESLIAESVTLINDGSHQVGAAGSTMGEIVEAVRR
En.bac.2261-D782_2307-YP_007340470.1--36H-COG6/1-558     QGRGFAVVASEVRTLASRSAQAAKEIEGLISESVTLIERGSDEVVNAGATMTEIVDAVKR
Pe.atr.485-ECA1683-YP_049784.1--36H-COG6/1-573          QGRGFAVVASEVRSLAQRSAQAAKEIETLISESVNLVNSGSVLVDNAGQTMREIVDAVTN
Pe.car.1139-PC1_2617-YP_003018183.1--36H-COG6/1-573      QGRGFAVVASEVRSLAQRSAQAAKEIETLISESVNLVNSGSILVDNAGQTMKEIVDAVTN
Di.zea.1140-Dd1591_1535-YP_003003868.1--36H-COG6/1-574   QGRGFAVVAGEVRSLAQRSAQAAKEIEGLISESVSLVHSGSELVDKAGQTMHEIVQAVSN
Di.zea.1140-Dd1591_3580-YP_003005868.1--36H-COG6/1-572   QGRGFAVVAGEVRSLAQRSAQAAKEIEGLITESVALVETGSDQVSRAGETMQDIVDAVTS
Es.col.1836-Y75_p1397-YP_489687.1--36H-COG6/1-546       QGRGFAVVASEVRTLASRSAQAAKEIEGLISESVRLIDLGSDEVATAGKTMSTIVDAVAS
Pe.atr.485-ECA3902-YP_051990.1--36H-COG6/1-575          QGRGFAVVAGEVRNLAQRSAQAAKEIETLIGESGRLVNTGSGLVAQAGTTMGEIVRAVVS
Sa.bon.1474-SBG_1454-YP_004730321.1--36H-COG6/1-537      QGRGFAVVASEVRTLASRSAQAAKEIEGLIGESVNLIEQGADEVVAAGSTMNEIVEAVKR
Sa.ent.407-STM1626-NP_460585.1--36H-COG6/1-541          QGRGFAVVASEVRTLASRSAQAAKEIEGLIGASVSLIEQGSEEVIAAGSTMNEIVDAVKR
Pe.car.1139-PC1_3679-YP_003019230.1--36H-COG6/1-567      QGRGFAVVAGEVRNLAQRSAQAAKEIEGLIGESSRLVETGSGLVSQAGTTMGEIVRAVVS
Pectob.2320-W5S_4020-YP_006284955.1--36H-COG6/1-575      QGRGFAVVAGEVRNLAQRSAQAAKEIETLIGESGRLVDTGSGLVAQAGTTMGEIVRAVVS
Di.zea.1140-Dd1591_0523-YP_003002884.1--36H-COG6/1-568   QGRGFAVVAGEVRNLAQRSAQAAKEIESLIGESGKLVENGSVLVAKAGDTMDEIVKAVVS
Di.dad.235-Dda3937_02665-YP_003881279.1--36H-COG6/1-572  QGRGFAVVAGEVRSLAQRSAQAAKEIEGLITESVSLVETGSGQVSRAGETMQDIVNAVTS
```

```
Pr.mir.1265-PMI1666-YP_002151397.1--36H-COG1/1-568        VTDIMGEIASASDEQSRGISQVGLAVSEMDRVTQQNASLVEQSAAAAAGLEDQAVALTRL
Di.dad.235-Dda3937_02779-YP_003883640.1--36H-COG1/1-566   VTDIMGEIASASEEQSRGIDQVGLAVTEMDRVTQQNASLVEESASAAHALEEQVRVLNQA
Di.zea.1140-Dd1591_1542-YP_003003875.1--36H-COG1/1-566    VTDIMGEIASASEEQSRGIDQVGQAVTEMDRVTQQNSALVEESASAANTLEEQVRVLNQA
Pe.atr.485-ECA1691-YP_049792.1--36H-COG1/1-561            VTDIMGEIASASDEQSKGIDQVGQAVTEMDRVTQQNASLVEESAAAAVALEEQVKVLNQA
Se.pro.864-Spro_2983-YP_001479212.1--36H-COG1/1-556       VTDIMGEIASASDEQSRGIDQVGQAVAEMDRVTQQNASLVEESASAAAALEEQASMLTQS
Ph.asy.1114-PAU_02685-YP_003041519.1--36H-COG1/1-564      VTDIMGEIASASDEQSRGITQVGLAISEMDRVTQQNASLVEQSAAAAAALEDQAAGLKKL
Pr.stu.1965-S70_18135-YP_006218124.1--36H-COG1/1-561      VTDIMSHIALASDEQSKGISQVSIAVEEMDKVTQQNASLVEQSAVAANVLEEQVAKLAQL
Mo.mor.2189-MU9_1762-YP_007505181.1--36H-COG1/1-561       VTDIMAHIATASDEQSKGISQVSIAVAEMDKVTQQNASLVEQSAVAANVLEEQVAKLSQL
Pe.car.1139-PC1_2609-YP_003018175.1--36H-COG1/1-561       VTDIMGEIASASDEQSKGIDQVGQAVTEMDRVTQQNASLVEESAAAAVALEEQVKVLNQA
Xe.nem.162-XNC1_1620-YP_003711881.1--36H-COG1/1-567       VTDIMGEIASASDEQSRGITQVGVAISEMDRVTQQNASLVEQSAAAAAALEEQAKILTKS
Pectob.2320-W5S_1768-YP_006282731.1--36H-COG1/1-561       VTDIMGEIASASDEQSKGIDQVGQAVTEMDRVTQQNASLVEESAAAAVALEEQVKVLNQA
Pa.vag.184-Pvag_1723-YP_003931360.1--36H-COG1/1-558       VTDIMGEIASASDEQSRGIDQVGLAVTEMDRVTQQNASLVEESAAAAAALEDQASHLKQA
Ye.pes.133-YPZ3_2024-YP_003568196.1--36H-COG1/1-557       VTDIMGEIASASDEQSRGIDQIGQAVAEMDRVTQQNASLVEESASAAASLEEQASMLTQA
Ye.pse.585-YPTB2401-YP_070915.1--36H-COG1/1-557           VTDIMGEIASASDEQSRGIDQIGQAVAEMDRVTQQNASLVEESASAAASLEEQASMLTQA
Ye.ent.378-YE2575-YP_001006778.1--36H-COG1/1-557          VTDIMGEIASASDEQSRGIDQVGQAVTEMDRVTQQNASLVEESASAAAALEEQASMLTQS
Pa.ana.1905-PAJ_1540-YP_005934416.1--36H-COG1/1-563       VTDIMGEIASASDEQSRGIDQVGQAVTEMDRVTQQNASLVEESAAAAAALEDQASNLKQA
Ph.lum.1262-plu1853-NP_929126.1--36H-COG1/1-564           VTDIMGEIASASDEQSRGITQVGLAISEMDRVTQQNAALVEQSAAAAAALEDQAAGLKKL
Serrat.1901-SerAS13_3075-YP_006025914.1--36H-COG1/1-555   VTDIMGEIASASDEQSRGIDQVGQAVAEMDRVTQQNASLVEESASAAAALEEQASMLTQS
Sa.ent.404-STY2128-NP_456485.1--36H-COG1/1-553            VTDIMGEIASASDEQSRGIDQVALAVSEMDRVTQQNASLVQESAAAAAALEEQASRLTQA
Ra.aqu.1678-Q7S_08960-YP_005401597.1--36H-COG1/1-564      VTDIMGEITSASDEQSRGIEQVAQAITEMDRVTQQNASLVEESASASAALEEQASLLTQS
Se.mar.2260-D781_2759-YP_007345195.1--36H-COG1/1-555      VTDIMGEIASASDEQSRGIDQVGQAVAEMDRVTQQNASLVEESASAAAALEEQVSLLTRS
Rahnel.1320-Rahaq_1830-YP_004212575.1--36H-COG1/1-564     VTDIMGEITSASDEQSRGIEQVAQAITEMDRVTQQNASLVEESASASAALEEQASLLTQS
Se.ply.1407-SerAS9_3072-YP_004506452.1--36H-COG1/1-555    VTDIMGEIASASDEQSRGIDQVGQAVAEMDRVTQQNASLVEESASAAAALEEQASMLTQS
Ed.ict.1187-NT01EI_1461-YP_002932882.2--36H-COG1/1-555    VTDIMGEIASASDEQSRGIEQVGQAVSEMDRVTQQNAALVEESASAAASLEDQASHLNQA
En.aer.1436-EAE_15535-YP_004593297.1--36H-COG1/1-556      VTDIMGEIASASDEQSRGIDQVGQAVAEMDRVTQQNASLVEESASAAAALEEQASLLTQS
Xe.bov.105-XBJ1_1923-YP_003467827.1--36H-COG1/1-567       VTDIMGEIASASDEQSRGITQVGVAISEMDRVTQQNASLVEQSAAAAAALEEQAMILTKA
Serrat.1408-SerAS12_3073-YP_004501499.1--36H-COG1/1-555   VTDIMGEIASASDEQSRGIDQVGQAVAEMDRVTQQNASLVEESASAAAALEEQASMLTQS
Er.bil.197-EbC_25350-YP_003741913.1--36H-COG1/1-559       VTDIMGEIASASDEQSKGIDQVGTAVTEMDRVTQQNASLVEESAAAAAALEEQASRLTQA
Er.tas.1011-ETA_14650-YP_001907404.1--36H-COG1/1-554      VTDIMGEIASASDEQSRGIDQVGEAVTEMDRVTQQNAALVEQSASAAASLEDQASRLTQA
Sa.ent.407-STM4533-NP_463392.1--36H-COG1/1-553            VTDIMGEIASASDEQSRGIDQVGLAVAEMDRVTQQNASLVEESAAAAAALEEQASRLTQA
Es.col.1836-Y75_p4240-YP_492486.1--36H-COG1/1-551         VTDIMGEIASASDEQSRGIDQVGLAVAEMDRVTQQNAALVEESAAAAAALEEQASRLTEA
En.bac.2261-D782_3913-YP_007342002.1--36H-COG1/1-553      VTDIMGEIASASDEQSRGIDQVGLAVSEMDRVTQQNASLVEESAAAAAALEEQASRLTQA
Ci.kos.578-CKO_03442-YP_001454958.1--36H-COG1/1-554       VTDIMGEIASASDEQSRGIDQVGLAVAEMDRVTQQNASLVEESAAAAAALEEQASRLTQA
Cr.sak.579-ESA_03402-YP_001439456.1--36H-COG1/1-555       VTDIMGEIASASDEQSRGIDQVGLAVAEMDRVTQQNASLVEQSAAAAAALEEQASRLTQS
Pantoe.297-Pat9b_1606-YP_004115479.1--36H-COG1/1-558      VTDIMGEIASASDEQSRGIDQVGLAVNEMDRVTQQNAALVEESATAAAALEDQASRLKQS
Cr.tur.6-CTU_05670-YP_003208930.1--36H-COG1/1-555         VTDIMGEIASASDEQSRGIDQVGLAVAEMDRVTQQNASLVEQSAAAASALEEQASRLTQS
Sa.bon.1474-SBG_3944-YP_004732724.1--36H-COG1/1-553       VTDIMGEIASASDEQSRGIDQVGLAVAEMDRVTQQNASLVEESAAAAAALEEQASRLTQA
En.asb.1498-Entas_0547-YP_004827084.1--36H-COG1/1-554     VTDIMGEIASASDEQSRGIDQVGLAVAEMDRVTQQNASLVEESAAAAAALEEQASRLTQA
Ci.rod.62-ROD_48461-YP_003368233.1--36H-COG1/1-554        VTDIMGEIASASDEQSRGIDQVGLAVAEMDRVTQQNASLVEESAAAAAALEEQASRLTQA
Ed.tar.1771-ETAF_1249-YP_005698855.1--36H-COG1/1-529      VTDIMGEIASASDEQSRGIEQVGQAVSEMDRVTQQNAALVEESASAAASLEDQASHLNQA
En.638.865-Ent638_0513-YP_001175251.1--36H-COG1/1-586     VTDIMGEIASASDEQSRGIDQVGLAVAEMDRVTQQNASLVEESAAAAAALEEQASRLTQA
Ed.ict.1187-NT01EI_1462-YP_002932883.1--36H-COG1/1-532    VTDIMGEISSASEEQSRGIEQVALAVTQMDQVTQQNAALVEEAASATQSLEAQADVLTRA
Ye.ent.378-YE2573-YP_001006777.1--36H-COG1/1-545          VTDIMGEIASASDEQSRGIEQVSLAVTQMDQVTQQNAALVEEAAAAANALEEQAGMLSDA
Ed.tar.1771-ETAF_1250-YP_005698856.1--36H-COG1/1-530      VTDIMGEISSASEEQSRGIEQVAQAVTQMDQVTQQNAALVEEAASATQSLEAQADVLSRA
Mo.mor.2189-MU9_1763-YP_007505182.1--36H-COG1/1-524       VTELMSEIASASNEQSRGIEQVATAVSQMDLVTQQNAALVEQSTGATAALEDQANNLVDT
En.aer.1436-EAE_15540-YP_004593298.1--36H-COG1/1-536      VTDIMGEIASASDEQSRGIEQVTQAVTQMDQVTQQNAALVVESASAAAALEEQAITLADA
En.638.865-Ent638_2456-YP_001177176.1--36H-COG1/1-533     VNDIMGEIASASDEQRRGIEQVALAVAQMDQVTQQNASLVEEAAAATDQLANQADHLTSL
Se.pro.864-Spro_2982-YP_001479211.1--36H-COG1/1-541       VTDIMGEIASASDEQSRGIEQVTQAVTQMDQVTQQNAALVEEAASAAVALEEQAITLADA
Es.col.1836-Y75_p1861-YP_490147.1--36H-COG1/1-533         VNDIMGEIASASEEQQRGIEQVAQAVSQMDQVTQQNASLVEEAAVATEQLANQADHLSSR
Serrat.1901-SerAS13_3074-YP_006025913.1--36H-COG1/1-541   VTDIMGEIASASDEQSRGIEQVTQAVTQMDQVTQQNAALVEEAASAAAALEDQAITLADA
Ye.pes.133-YPZ3_2023-YP_003568195.1--36H-COG1/1-536       VTDIMGEIESASDEQSRGIEQISLAVTQMDQVTQQNASLVEEAAAAANALEEQASMLSDA
Serrat.1408-SerAS12_3072-YP_004501498.1--36H-COG1/1-541   VTDIMGEIASASDEQSRGIEQVTQAVTQMDQVTQQNAALVEEAASAAAALEDQAITLADA
Se.mar.2260-D781_2758-YP_007345194.1--36H-COG1/1-534      VTDIMGEIASASDEQSRGIEQVTQAVTQMDQVTQQNAALVVQAASAASGLEEQAIALADA
Ye.pse.585-YPTB2400-YP_070914.1--36H-COG1/1-536           VTDIMGEIESASDEQSRGIEQISLAVTQMDQVTQQNASLVEEAAAAANALEEQASLLSDA
Se.ply.1407-SerAS9_3071-YP_004506451.1--36H-COG1/1-541    VTDIMGEIASASDEQSRGIEQVTQAVTQMDQVTQQNAALVEEAASAAAALEDQAITLADA
Pr.stu.1965-S70_18140-YP_006218125.1--36H-COG1/1-521      VTELMGNIASASNEQSRGIEQVATAVSQMDLVTQQNAALVEQSTSATAALEDQANNLVDT
En.bac.2261-D782_1749-YP_007339930.1--36H-COG1/1-533      VNDIMGEIASASDEQRRGIEQVALAVSQMDQVTQQNAALVEEGAAATEQLTGQADTLTTL
En.asb.1498-Entas_2590-YP_004829104.1--36H-COG1/1-533     VNDIMGEIASASDEQRRGIEQVAQAVSQMDQVTQQNASLVEEAAAATDQLASQADHLTGL
En.asb.1498-Entas_3672-YP_004830170.1--36H-COG1/1-545     VTTIMKQIASASEEQSKGISQVGVAISQMDGVTQQNASLVEQVSAAASALERQTEDLQRS
Ci.kos.578-CKO_01067-YP_001452646.1--36H-COG1/1-537       VNDIMGEIASASDEQRRGIEQVAQAVSQMDQVTQQNAALVEEAAAATDQLANQADHLSAR
En.clo.1544-EcWSU1_02810-YP_004952663.1--36H-COG1/1-538   VNDIMGEIASASDEQRRGIEQVAQAVSQMDQVTQQNASLVEEAAAATDQLASQADHLTGL
Sa.ent.407-STM3152-NP_462067.1--36H-COG1/1-547            VTTIMKQIASASEEQSKGISQVGVAITQMDSVTQQNAALVEQVSAAAAALERQTEDLQRS
Pe.atr.485-ECA1332-YP_049438.1--36H-COG1/1-555            VTDIMGEIASASEEQSKGIAQVGQAVAEMDSVTQQNAALVEQASSAALSLEEQAALLNQT
Pe.car.1139-PC1_1208-YP_003016792.1--36H-COG1/1-555       VTDIMGEIASASEEQSKGIAQVGQAVAEMDSVTQQNAALVEQASSAALSLEEQAALLNQT
En.638.865-Ent638_3407-YP_001178118.1--36H-COG1/1-545     VTTIMKQIASASEEQSKGISQVGIAITQMDGVTQQNASLVEQVSSAASALEAQTEELQRS
Pa.vag.184-Pvag_1413-YP_003931052.1--36H-COG1/1-554       VDAIMKQIASASEEQSKGISQVGTAVTEMDSVTQQNASLVEEVSAAASALALQTEALQAS
Ph.asy.1114-PAU_02684-YP_003041518.1--36H-COG1/1-544      VTDLMAEIAAASDEQSRGIHQVAQAVTQMDQVTQQNAALVEQSAAAAAALEDQADILVKT
Pa.ana.1905-PAJ_1316-YP_005934192.1--36H-COG1/1-556       VDAIMKQIAAASEEQSKGISQVGTAVTEMDSVTQQNAALVEEVSAAASALALQTEALQAS
Ci.kos.578-CKO_04394-YP_001455885.1--36H-COG1/1-561       VTTIMKQIAAASEEQSKGISQVGVAITQMDSVTQQNAALVEQVSAAAAALERQTEELQRS
Sa.bon.1474-SBG_2750-YP_004731563.1--36H-COG1/1-547       VTTIMKQIASASEEQSKGISQVGVAITQMDSVTQQNAALVEQVSAAAAALERQTEALQRS
Ra.aqu.1678-Q7S_16025-YP_005402985.1--36H-COG1/1-554      VTDIMAEIASASDEQSKGISQVAVAVTEMDSVTQQNAALVEQASQAAVSLEEQASLLNNA
Rahnel.1320-Rahaq_3177-YP_004213898.1--36H-COG1/1-554     VTDIMAEIASASDEQSKGISQVAVAVTEMDSVTQQNAALVEQASQAAVSLEEQASLLNNA
Pectob.2320-W5S_3114-YP_006284066.1--36H-COG1/1-555       VTDIMGEIASASEEQSKGIAQVGQAVAEMDSVTQQNAALVEQASSAALSLEEQAALLNQT
Ye.ent.378-YE2971-YP_001007160.1--36H-COG1/1-552          VTDLMGEIASASDEQSKGITQVGQAVAEMDSVTQQNAALVQQASRAAAALEEQATQLNQA
Er.bil.197-EbC_20190-YP_003741400.1--36H-COG1/1-556       VTEIMKQIASASEEQSKGISQVGTAITEMDSVTQQNASLVEQVSAAASQLERQTEELTLS
Ph.lum.1262-plu1854-NP_929127.1--36H-COG1/1-544           VTDLMAEIAAASDEQSRGIHQVAQAVTQMDQVTQQNAALVEQSAAAAAALEDQADILVQI
Er.bil.197-EbC_25340-YP_003741912.1--36H-COG1/1-528       VNDIMGEIASASDEQRRGIEQVAQAVSQMDQVTQQNAALVEEAAAATEALEAQADHLTGT
Di.zea.1140-Dd1591_2937-YP_003005238.1--36H-COG1/1-564    VTDIMSEIATASDEQSKGIAQAGQAITEMDGVTQQNAALVQEASAATRSLEEQAMVLTEA
Er.tas.1011-ETA_14670-YP_001907406.1--36H-COG1/1-518      VSEIMGEIAAASDEQRRGIEQVAMAVSQMDQVTQQNAALVEEGAAATGALEAQAGQLTET
Sa.ent.404-STY4234-NP_458344.1--36H-COG1/1-547            VTDIMGEIASASDEQSKGIEQVAQAVSEMDSVTQQNASLVEESAAAAAALEDQANELRQA
Sa.ent.407-STM3577-NP_462478.1--36H-COG1/1-547            VTDIMGEIASASDEQSKGIEQVAQAVSEMDSVTQQNASLVEESAAAAAALEDQANELRQA
Sa.bon.1474-SBG_3170-YP_004731982.1--36H-COG1/1-547       VTDIMGEIASASDEQSKGIEQVAQAVSEMDSVTQQNASLVEESAAAAAALEDQANELRQA
En.asb.1498-Entas_2591-YP_004829105.1--36H-COG1/1-555     VTDIMGEIASASDEQSRGIDQVALAVSEMDRVTQQNAALVQESATAAAALEDQASRLKMA
En.638.865-Ent638_1858-YP_001176586.1--36H-COG1/1-552     VTDIMGEIASASDEQSRGIEQVSLAVSQMDSVTQQNAALVQESATAAAALEDQAEQLRQA
Ci.rod.62-ROD_19331-YP_003365490.1--36H-COG1/1-553        VTDIMGEIASASDEQSRGIDQVALAVAEMDRVTQQNASLVQESAAAAAALEDQASRLTRA
Di.dad.235-Dda3937_01559-YP_003882087.1--36H-COG1/1-566   VTDIMAEIATASDEQSKGIAQAGQAITEMDSVTQQNAALVQEASAATRSLEEQSMVLTEA
Er.bil.197-EbC_37780-YP_003743156.1--36H-COG1/1-553       VTDIMGEIASASDEQSRGIDQIGLAVTEMDRVTQQNASLVQESAAASASLEEQASRLSQA
Pantoe.297-Pat9b_2001-YP_004115872.1--36H-COG1/1-558      VNEIMKHIASASEEQSKGISQVGTAVTEMDSVTQQNASLVEEVSAAASALERQTEELQAS
```

```
En.clo.1544-EcWSU1_01962-YP_004951821.1--36H-COG1/1-553  VTDIMGEISSASDEQSRGIEQVSLAVSQMDSVTQQNAALVQQSATAAAALEDQSEQLRQA
En.asb.1498-Entas_1879-YP_004828403.1--36H-COG1/1-554    VTDIMGEISSASDEQSRGIEQVSLAVSQMDSVTQQNAALVQESATAAAALEDQSEQLRQA
En.bac.2261-D782_1748-YP_007339929.1--36H-COG1/1-547     VTDIMGEISSASDEQSRGIDQVALAVTEMDKVTQQNAALVEESAAAAAALEEQAGKLSEA
Es.col.1836-Y75_p1862-YP_490148.1--36H-COG1/1-553        VTDIMGEIASASDEQSRGIDQVALAVSEMDRVTQQNASLVQESAAAAAALEEQASRLTQA
Cr.tur.6-CTU_33010-YP_003211664.1--36H-COG1/1-555        VTDIMGEIASASDEQSRGIEQVSLAVSQMDSVTQQNAALVQESAAAAGALEDQAEQLRQA
Cr.tur.6-CTU_25780-YP_003210941.1--36H-COG1/1-536        VTDIMGEIASASDEQSRGIDQVALAVSEMDRVTQQNASLVQQSAAAASALEDQASRLSQA
En.clo.1544-EcWSU1_00403-YP_004950264.1--36H-COG1/1-549  VTDIMGEIASASDEQSRGIDQVGQAVAEMDRVTQQNASLVEESAAAAAALEDQAARLNEA
En.638.865-Ent638_2457-YP_001177177.1--36H-COG1/1-555    VTDIMGEIASASDEQSRGIDQVALAVQEMDRVTQQNAALVQESAAAAAALEDQASRLKMA
Sa.bon.1474-SBG_1755-YP_004730609.1--36H-COG1/1-553      VTDIMGEIASASDEQSRGIDQVALAVSEMDRVTQQNASLVQESAAAAAALEEQASRLTMA
Pr.mir.1265-PMI1665-YP_002151396.1--36H-COG1/1-548       VTELMAEIASASDEQSRGIHQVADAVSQMDQVTQQNAALVEQSASAATALEDQANNLVNA
Cr.sak.579-ESA_01348-YP_001437444.1--36H-COG1/1-556      VTDIMGEIASASDEQSRGIDQVALAVSEMDRVTQQNASLVQQSAAAASALEDQASRLSQA
Cr.sak.579-ESA_00560-YP_001436686.1--36H-COG1/1-555      VTDIMGEIASASDEQSRGIEQVSLAVSQMDSVTQQNAALVQESAAAAAALEDQAEQLRQA
Sa.ent.407-STM1919-NP_460876.1--36H-COG1/1-553           VTDIMGEIASASDEQSRGIDQVALAVSEMDRVTQQNASLVQESAAAAAALEEQASRLTQA
Ci.kos.578-CKO_01066-YP_001452645.1--36H-COG1/1-552      VTDIMGEIASASDEQSRGIDQVALAVSEMDRVTQQNASLVQESAAAAAALEEQASRLTMA
En.asb.1498-Entas_0424-YP_004826963.1--36H-COG1/1-549    VTDIMGEIASASDEQSRGIDQVGQAVAEMDRVTQQNASLVEESAAAAAALEDQAARLNEA
Pantoe.297-Pat9b_1273-YP_004115150.1--36H-COG1/1-553     VTDIMGEIASASDEQSRGIDQIGQAVNEMDRVTQQNATLVQESANASVSLEQQASRLSAA
Ed.tar.1771-ETAF_2264-YP_005699859.1--36H-COG1/1-555     ISDTMQEISSASEEQSRGIAQIALAINEMDKVTQQNAALVEQSAAAGALEDQADRLNQT
Pa.ana.1905-PAJ_3534-YP_005936409.1--36H-COG1/1-553      VTQLMAEIASASAEQTRGIEQVSKAVSEMDGVTQQNAALVEESASAAASLEDQATQLRQS
Pa.vag.184-Pvag_1203-YP_003930842.1--36H-COG1/1-552      VTQLIEEIAASSTEQTRGIEQVCIAVSEMDGVTQQNAALVEESATAAASLEEQASYLRKT
Pantoe.297-Pat9b_1605-YP_004115478.1--36H-COG1/1-556     VTDIMGEIASASDEQSRGIDQVGQAVTEMDRVTQQNASLVEQSAAAAASLEEQASRLSQS
Cr.sak.579-ESA_00210-YP_001436348.1--36H-COG1/1-557      VTDIMGEIASASDEQSRGIDQVGLAVSEMDKVTQQNAALVEESAAAAAALEDQAGKLNEA
Er.tas.1011-ETA_pET460340-YP_001905943.1--36H-COG1/1-543 VTEIMKQISCASEEQSKGINQVATAVSEMDSVTQQNASLVGEVSSAASALERQTVELTLS
Cr.tur.6-CTU_36610-YP_003212024.1--36H-COG1/1-557        VTDIMGEIASASDEQSRGIDQVGLAVSEMDKVTQQNAALVEESAAAAAALEDQAGKLNEA
Pe.car.1139-PC1_1159-YP_003016743.1--36H-COG1/1-556      VTDIMGEIASASDEQSRGIEQVGQAVNEMDRVTQQNASLVEESASAAAALEEQARYLQNA
Er.tas.1011-ETA_14640-YP_001907403.1--36H-COG1/1-554     VTDIMGEIASASDEQSRGIDQVGEAVNEMDRVTQQNAALVEQSASASASLEEQASRLTQA
Pa.vag.184-Pvag_1725-YP_003931362.1--36H-COG1/1-556      VTDIMGEIASASDEQSRGIDQVGQAVTEMDRVTQQNASLVEESAAAAASLEDQASRLSQS
Ed.ict.1187-NT01EI_3742-YP_002935104.1--36H-COG1/1-525   VTGIIAEIAAASDEQSQGIAQVSQAVSEMEQVTQQNAALVQQSAASATSLEERACSLSQA
En.638.865-Ent638_0380-YP_001175119.1--36H-COG1/1-549    VTDIMGEIASASDEQSRGIDQVGLAVAEMDRVTQQNSSLVEESAAAAAALEDQAARLNEA
Er.bil.197-EbC_25360-YP_003741914.1--36H-COG1/1-556      VTDIMGEIASASDEQSKGIDQVGTAVTEMDRVTQQNASLVEESATAAAALEDQASRLTQA
Er.tas.1011-ETA_16910-YP_001907630.1--36H-COG1/1-543     VTEIMKQISCASEEQSKGIDQVATAVSEMDSVTQQNASLVEEVSSAASALERQTVELTLS
Es.fer.1173-EFER_3055-YP_002384154.1--36H-COG1/1-603     VTDIMGEIASASDEQSRGIDQVGLAVAEMDRVTQQNAALVEESAAAAAALEEQASRLNEA
Xe.nem.162-XNC1_1619-YP_003711880.1--36H-COG1/1-520      VTDLMAEIAAASDEQSRGIHQVAQAVSQMDQVTQQNAALVEQSAAAASALEEKADVLVKT
En.clo.1544-EcWSU1_02811-YP_004952664.1--36H-COG1/1-559  VTDIMGEIASASDEQSRGIDQVALAVSEMDRVTQQNAALVQESAAAAAALEDQASRLKMA
Di.dad.235-Dda3937_03500-YP_003882750.1--36H-COG1/1-553  VHDLMGEISAASDEQSRGIAQIGQAVTEMDGVTQQNAALVEEASTAAASLDDQAQSLAAA
Xe.bov.105-XBJ1_1924-YP_003467828.1--36H-COG1/1-523      VTELMAEIAAASDEQSRGIHQVAQAVSQMDQVTQQNAALVEQSAAAAAALEDKADTLVKT
Rahnel.1320-Rahaq_1831-YP_004212576.1--36H-COG1/1-556    VTDIMGEIASASDEQSRGIEQVSIAVTQMDQVTQQNATLVQQAASSTHALEIESENLTRA
Ra.aqu.1678-Q7S_08965-YP_005401598.1--36H-COG1/1-556     VTDIMGEIASASDEQSRGIEQVSIAVTQMDQVTQQNATLVQQAASSTHALEIESENLTRA
Cr.sak.579-ESA_01126-YP_001437230.1--36H-COG1/1-514      VSTIIKQIASASEEQSKGISQVGIAITQMDTVTQQNASLVEQVSAAAAALERQTEELQST
Pe.atr.485-ECA2712-YP_050803.1--36H-COG1/1-560           VNDLMGEIASASDEQSRGISQIGLAVTEMDGVTQQNSALVQESAAAAASLEDQARQLTEA
En.clo.1544-EcWSU1_00560-YP_004950421.1--36H-COG1/1-597  VTDIMGEIASASDEQSRGIDQVGLAVAEMDRVTQQNASLVEESAAAAAALEEQASRLTQA
Di.dad.235-Dda3937_03498-YP_003882752.1--36H-COG1/1-561  VSDLIGEIASASDEQSRGISQIGQAVNEMDGVTQQNATLVQQAMAAIASLEEQAQQLTKA
Cr.tur.6-CTU_27880-YP_003211151.1--36H-COG1/1-514        VSTIIKQIASASEEQSKGISQVGLAITQMDTVTQQNASLVEQVSAAAAALERQTEELQST
Pe.car.1139-PC1_1669-YP_003017246.1--36H-COG1/1-560      VNDLMGEIASASDEQSRGITQIGQAVTEMDGVTQQNAALVQESAAAAASLEDQARQLTEA
Ra.aqu.1678-Q7S_14450-YP_005402672.1--36H-COG1/1-551     VNDIMSEIAAASTEQSKGINQIGIAVVQMDSVTQQNAALVQESAAAAASLEEQARQLTEI
Ci.kos.578-CKO_03622-YP_001455137.1--36H-COG1/1-517      VTDIMGEIASASDEQSRGIDQVGLAVAEMDRVTQQNAALVEESATAAVALEEQAARLTQT
Di.zea.1140-Dd1591_2408-YP_003004728.1--36H-COG1/1-557   VHDLMGEISAASDEQSRGIAQIGQAVTEMDGVTQQNAALVQEASTAAASLEEQAQNLAEA
Di.zea.1140-Dd1591_2406-YP_003004726.1--36H-COG1/1-559   VSNLIGEIASASDEQSRGISQIGQAVNEMDSVTQQNATLVQQAMAAIASLEEQARQLTQA
Ed.ict.1187-NT01EI_2801-YP_002934203.1--36H-COG1/1-554   ISDTMQEISSASEEQSRGIAQIALAINEMDKVTQQNAALVEQSAAAGALEDQAGHLNQT
Pe.car.1139-PC1_1384-YP_003016966.1--36H-COG1/1-559      FNDLIGEIAAATDEQTRGITQISQAVHEMDSVTQQNASLVMQSAAAAARLDEQTSELSAV
Rahnel.1320-Rahaq_2866-YP_004213595.1--36H-COG1/1-551    VNDIMSEIAAASTEQSKGINQIGIAVVQMDSVTQQNAALVQESAAAAASLEEQARQLTEI
Pe.atr.485-ECA1281-YP_049387.1--36H-COG1/1-556           VTDIMGEIASASDEQSRGIDQVGQAVNEMDRVTQQNASLVEESASAAAALEEQARFLQNA
Pectob.2320-W5S_1886-YP_006282848.1--36H-COG1/1-549      VNDLMGEIASASDEQSRGITQIGQAVTEMDGVTQQNASLVQESAAAAASLEDQARQLTEA
Pectob.2320-W5S_1696-YP_006282659.1--36H-COG1/1-559      VNDLIGEIAAATDEQTRGITQISQAVHEMDSVTQQNALLVTQSAEAAARLDEQTSELSAV
Di.zea.1140-Dd1591_2407-YP_003004727.1--36H-COG1/1-555   VHDLMGEISAASDEQSRGIDQIGQAVTEMDGVTQQNAALVQEASVAAASLDEQAQSLAAA
Pe.atr.485-ECA1509-YP_049611.1--36H-COG1/1-559           VNDLIGEIAAATDEQTRGITQISQAVHEMDSVTQQNASLVMQSAEAAARLDEQTSELSAV
Pa.vag.184-Pvag_1724-YP_003931361.1--36H-COG1/1-520      VTDIMGEIASASDEQSRGIEQVGLAVTEMDRVTQQNASLVEESAAAAAALEQQASVLTQA
Pa.ana.1905-PAJ_1541-YP_005934417.1--36H-COG1/1-520      VTDIMGEIASASDEQSRGIDQVGQAVTEMDRVTQQNASLVEESAAAAAALEQQAGALSQA
Mo.mor.2189-MU9_1596-YP_007505015.1--36H-COG1/1-519      VTEIMKEIAIASEEQTKGILQVGTAITQMDTVTQQNASLVSNVSETAAGLELQTQALQNS
Pr.mir.1265-PMI2808-YP_002152509.1--36H-COG1/1-563       VSGSMKEILAASEEQTRGIAQISLAVNEMDKATQQNAAMVEQSTAASATLSEQASMLDMI
Di.zea.1140-Dd1591_0395-YP_003002764.1--36H-COG1/1-556   VTDIMGEIASASSEQSKGISQVGTAITQMDSVTQQNAALVEQSSATSASLEEQAHHLNEI
En.clo.1544-EcWSU1_03786-YP_004953635.1--36H-COG1/1-515  VTTIMKQIASASEEQSKGISQVGIAITQMDGVTQQNASLVEEVSAAASALERQTEELQRS
Pectob.2320-W5S_3170-YP_006284116.1--36H-COG1/1-556      VTDIMGEIASASDEQSRGIDQVGQAVNEMDRVTQQNASLVEESASAAAALEEQARFLQNA
Di.dad.235-Dda3937_02184-YP_003884754.1--36H-COG1/1-556  VTDIMGEIASASSEQSKGISQVGTAITQMDSVTQQNAALVEQSSATSSSLEEQAHHLNEI
Ci.rod.62-ROD_32641-YP_003366748.1--36H-COG1/1-517       VTDIMGEIASASDEQSRGIDQVGQAVAEMDRVTQQNAALVEESASAAAALEEQAARLNQT
Di.dad.235-Dda3937_03499-YP_003882751.1--36H-COG1/1-561  VHDLMGEISAASDEQSRGIAQIGQAVTEMDGVTQQNAALVQEATTAAASLEEQAQSLAAA
Di.dad.235-Dda3937_00105-YP_003883346.1--36H-COG1/1-561  VTDIMGEISSASEEQSRGIDQVSQAVSEMDRVTQQNAVLVEQSANAATSLEEQSDHLNRA
Pantoe.297-Pat9b_1604-YP_004115477.1--36H-COG1/1-555     VTDIMGEIASASDEQSRGIDQVGQAVTEMDRVTQQNASLVEQSAAAAASLEEQASRLSQS
Er.tas.1011-ETA_14660-YP_001907405.1--36H-COG1/1-560     VTDIMGEIASASDEQSRGIDQVSEAVTEMDRVTQQNAALVEQSASAAASLEEQASRLTQA
Pr.mir.1265-PMI2809-YP_002152510.1--36H-COG1/1-575       VESSMTEILSASEEQTRGISQVSLAVTEMDKATQQNAAMVEQSSAVASLLTEEAGDLEQI
Ed.tar.1771-ETAF_3053-YP_005700648.1--36H-COG1/1-516     VTGIIAEIAAASDEQSLGIAQVSQAVSEMEQVTQQNAALVQQSAASATSLEERACGLSQA
Se.pro.864-Spro_1415-YP_001477647.1--36H-COG1/1-546      VTETMQEISSASEEQSRGIAQIAQAVNEMDKVTQQNAALVEQSASAASALEDQANHLNQT
Di.zea.1140-Dd1591_1804-YP_003004134.1--36H-COG1/1-562   VTDIMGEISSASEEQSRGIDQVSQAVSEMDRVTQQNAVLVEQSANAAVSLEEQSDQLNRA
Di.dad.235-Dda3937_03501-YP_003882749.1--36H-COG1/1-575  VHELMGEISAASDEQSRGIAQIGQAVTEMDGVTQQNAALVEEASAAAASLEDQAQSLAVA
Pantoe.297-Pat9b_0851-YP_004114731.1--36H-COG2/1-524     VADIMSEITIASSEQSSGISEINLAITQMEAVTQQNAALVQEASAASQALQEQAERLAQS
Pa.vag.184-Pvag_0291-YP_003929953.1--36H-COG2/1-525      VANIMSEITIASSEQSSGINEINQAITQMEAVTQQNAALVQEASAASQALQDQAERMAQA
En.clo.1544-EcWSU1_03886-YP_004953735.1--36H-COG2/1-519  MDVILREIRQASHEQTEGISQINSAIGLIDATTQQNSALVEESVAAAASLNEQAMHLKDL
Cr.tur.6-CTU_04790-YP_003208842.1--36H-COG2/1-515        MDTILREIAQASREQTDGISQINSAIGMIDTTTQQNSALVEESVAAASSLNDQAHHLKEM
Ci.kos.578-CKO_04484-YP_001455975.1--36H-COG2/1-521      MDVILREIGQASREQTDGISQINSAIGLIDATTQQNSCLVEESVAAAASLNEQAQHLKEI
Cr.sak.579-ESA_03488-YP_001439539.1--36H-COG2/1-515      MDTILREIAQASREQTDGISQINSAIGMIDTTTQQNSALVEESVAAASSLNDQAHNLKEM
Ci.rod.62-ROD_48071-YP_003368199.1--36H-COG2/1-521       MDVILREIGQASSEQTDGISQINSAVGLIDAATHQNSSLVEESVAAAASLNEQALRLKDM
Sa.bon.1474-SBG_2851-YP_004731664.1--36H-COG2/1-521      MDVILREIGQASREQTDGISQINSAIGLIDAATQQNSCLVEESVAAAASLNEQALHLKEL
Sa.ent.407-STM3216-NP_462130.1--36H-COG2/1-521           MDVILREIGQASREQTDGISQINSAIGLIDAATQQNSCLVEESVAAAASLNEQALHLKEL
En.asb.1498-Entas_3753-YP_004830248.1--36H-COG2/1-519    MDVILREIRQASHEQTEGISQINSAIGLIDATTQQNSALVEESVAAAASLNEQATQLKEL
Mo.mor.2189-MU9_3389-YP_007506806.1--36H-COG2/1-521      MADLLHEIGHASHEQTDGITQINSAIGLIDSATQQNATLVEQSVAAASSLNEQAGTLNEM
Sa.ent.404-STY3394-NP_457606.1--36H-COG2/1-522           MDVILREIGQASREQTDGISQINSAIGLIDAATQQNSCLVEESVAAAASLNEQALHLKEL
En.638.865-Ent638_3526-YP_001178235.1--36H-COG2/1-519    MDLILRDIRQASHEQTEGISQINSAIGLIDSTTQQNSALVEESVAAAASLNDQAMHLKEL
Pe.atr.485-ECA1774-YP_049874.1--36H-COG2/1-554           VTGIISEIAQASREQSDGINQINLAVGQIDTTTQQNASLVEESAAAALSLQDQANSLART
En.638.865-Ent638_2119-YP_001176845.1--36H-COG2/1-549    VHTLVDEIARASHEQSEGITQINITMGQIDTTTQQNASLVEESSAASGSLKEQARILIES
```

```
Pectob.2320-W5S_2813-YP_006283770.1--36H-COG2/1-554      VTGIISEIAQASREQSDGINQINLAVGQIDTTTQQNAALVEESAAAALSLQDQANSLART
Di.zea.1140-Dd1591_0695-YP_003003055.1--36H-COG2/1-561   VHGIINEISQASHEQSDGISQINIAVGQIDTTTQQNAALVEESASAALSLQSQAAQLAEA
Pa.vag.184-Pvag_pPag30079-YP_003729820.1--36H-COG2/1-546 LSDTVEEITAASAEQARGIEQVGIAVTEMDGVTQQNASLVEESTSASQSLREQASLLLQS
Er.bil.197-EbC_38940-YP_003743272.1--36H-COG2/1-526      VTAIMGEISIASSEQSTGINEISMAITQMDQVTQQNAALVNQSASAAQSLQEQAEQLSRA
Ra.aqu.1678-Q7S_24891-YP_005419232.1--36H-COG2/1-554     VTDIVGEIAIASNEQSTGIEEINKAITQMDEVTQQNAALVQEASSAAYSLNEQAERLSQA
En.asb.1498-Entas_2287-YP_004828805.1--36H-COG2/1-513    VNDIMGEISSASEEQSRGIEQISRAVMELDTTTQQNASLVGASSTAAGALEDQARVLEEL
Rahnel.1320-Rahaq_4869-YP_004215574.1--36H-COG2/1-554    VTDIVGEIAIASNEQSTGIEEINKAITQMDEVTQQNAALVQEASSAAYSLNEQAERLSQA
Di.dad.235-Dda3937_00027-YP_003883574.1--36H-COG2/1-554  ADGVITEIAKASLEQSEGINQINLAIGQIDTTTQQNAALVEESAAAALSLQEQAQTLAKT
Pr.stu.1965-S70_05790-YP_006215722.1--36H-COG2/1-520     MDQRLQEIGQASHEQSAGILQINSAVGQLDVTTQQNASLVEESVAAAASLNEQALHLKQL
En.638.865-Ent638_2100-YP_001176826.1--36H-COG2/1-512    VNDIMGEISSASEEQSRGIEQISRAVMELDSTTQQNASLVGASSSAAGALEDQARLLESL
Pe.car.1139-PC1_2526-YP_003018093.1--36H-COG2/1-554      VTGIISEIAQASREQSDGINQINLAVGQIDTTTQQNAALVEESAAAALSLQDQANSLART
Mo.mor.2189-MU9_702-YP_007504121.1--36H-COG2/1-525       VSQMIDELTLAANEQSQGISQVGIAVQQIDEVTQQNAALVVENAAAAESLHQQAGQLMQV
Pe.car.1139-PC1_3443-YP_003018995.1--36H-COG2/1-511      VHTIMKDITTASDEQHRGISQVGQAIIEMDQNTQQNTVLVEQSSSAAHSLEQQAIVLSDA
Pa.ana.1905-PAJ_0915-YP_005933791.1--36H-COG2/1-559      LTDTVEEINSASAEQARGIEQVGIAVTEMDTTTQQNAALVEESASAASSLREQAERLLQA
Pectob.2320-W5S_3736-YP_006284671.1--36H-COG2/1-510      VHAIMKDITTASDEQHRGISQVGQTIIEMDQNTQQNTVLVEQSSSAAHSLAQQAIMLSDA
Pe.atr.485-ECA0183-YP_048310.1--36H-COG2/1-553           VNEIISEISQASREQSDGINQMNLAIGQIDTTTQQNAALVEESASAALSLQAQASVLAEA
Rahnel.1320-Rahaq_3107-YP_004213828.1--36H-COG2/1-503    VTQIIHEIASASDEQSRGISQIAQAVAEMDTVTQQNSALVQQSASAAASLDEQAVNLERT
Pe.car.1139-PC1_4071-YP_003019622.1--36H-COG2/1-553      VNEIISEISQASREQSDGINQMNLAVGQIDTTTQQNAALVEESASAALSLQAQASVLAEA
Di.dad.235-Dda3937_03462-YP_003884507.1--36H-COG2/1-563  VHSIINEISQASHEQSDGISQINIAVGQIDTTTQQNAALVEESASAALSLQSQASLLAEA
Ra.aqu.1678-Q7S_15665-YP_005402913.1--36H-COG2/1-503     VTQIIHEIASASDEQSRGISQIAQAVAEMDTVTQQNSALVQQSASAAASLDEQAVNLERT
Pectob.2320-W5S_4505-YP_006285423.1--36H-COG2/1-555      VNEIISEISQASREQSDGINQMNLAVGQIDTTTQQNAALVEESASAALSLQAQASVLAEA
Pe.atr.485-ECA3642-YP_051730.1--36H-COG2/1-551           VSDLVGEISSASNEQTMGIEQVNVAVNQMEVTTQQNASLVNEASAATQSLQQQAAQLAET
Pe.car.1139-PC1_1417-YP_003016999.1--36H-COG2/1-554      LSDTVSEISAASAEQSTGVEQVGIAVSQMDQTTQQNAALVEESAAAAQSLKEQADQLVRE
En.asb.1498-Entas_4509-YP_004821569.1--36H-COG2/1-559    VDTFINEIARAAAEQSEGISQINLAVNQLDTTTQQNASLVEESAGAAHSMAAQANELTRL
Pa.ana.1905-PAJ_3064-YP_005935940.1--36H-COG2/1-544      LSDTVEEITSASAEQARGVEQVGIAVTEMDSATQQNAALVEESASAANSLREQARQLLQA
Pectob.2320-W5S_1733-YP_006282696.1--36H-COG2/1-535      LSDTVSEISSASAEQSTGVEQVGIAVSQMDQTTQQNAALVEESAAAAQSLKEQADQLVRE
Pe.atr.485-ECA4334-YP_052421.1--36H-COG2/1-556           VANLLNEIGLTTHEQEQGVAQVNDAVNQLDQVTQQNAALVEESASAADSLSQQARTLLEL
Di.zea.1140-Dd1591_1602-YP_003003935.1--36H-COG2/1-554   ADGFIKEIARASLEQSEGINQINLAIGQIDTTTQQNAALVEESAAAALSLQEQAQTLAKT
Pectob.2320-W5S_4506-YP_006285424.1--36H-COG2/1-540      VHDIISEISQASREQSDGINQMNLAIGQIDTTTQQNAALVEESASAAQSLQSQATVLAEA
Di.zea.1140-Dd1591_1453-YP_003003794.1--36H-COG2/1-532   VTDIMGEISSASHEQSSGVSQIGEAVVSMERTTQQNAALVEEMAAAAGSLSSQSEELVQA
Pectob.2320-W5S_0106-YP_006281117.1--36H-COG2/1-556      VANLLSEIGLTTHEQEQGVAQVNDAVNQLDQVTQQNAALVEESASAADSLSEQARTLLEL
Di.zea.1140-Dd1591_0769-YP_003003127.1--36H-COG2/1-505   VHDIMGDIAVASDEQHRGISQVGNAIVEMDQTTQQNAVLVEQSSAAATSLEQQAELLSNA
Ra.aqu.1678-Q7S_18035-YP_005403387.1--36H-COG2/1-535     VNSLMAEITLASSEQHTGIDQVNKAVVNMDEATQQNAALVEQSAAAADSLKQQAHILLEG
Rahnel.1320-Rahaq_3579-YP_004214298.1--36H-COG2/1-535    VNSLMAEITLASSEQHTGIDQVNKAVVNMDEATQQNAALVEQSAAAADSLKQQAHILLEG
Pe.car.1139-PC1_4072-YP_003019623.1--36H-COG2/1-540      VHDIISEISQASREQSDGINQMNLAIGQIDTTTQQNAALVEESASAALSLQAQASVLAEA
Pe.car.1139-PC1_3464-YP_003019016.1--36H-COG2/1-551      VSDLVGEISSASNEQTMGIEQVNVAVNQMEVTTQQNASLVNEASAATQSLQQQAAQLAET
Pe.car.1139-PC1_0111-YP_003015709.1--36H-COG2/1-556      VANLLSEIGLTTHEQEQGVAQVNDAVNQLDQVTQQNAALVEESASAADSLSQQARTLLEL
Pe.atr.485-ECA0182-YP_048309.1--36H-COG2/1-542           VHGIISEISQASREQSDGINQMNLAIGQIDTTTQQNAALVEESASAALSLQSQASVLADA
Pectob.2320-W5S_3753-YP_006284688.1--36H-COG2/1-551      VSDLVGEISSASNEQTMGIEQVNVAVNQMEVTTQQNASLVNEASAATQSLQQQAAQLTET
Pa.ana.1905-PAJ_2666-YP_005935542.1--36H-COG2/1-514      LAGAMDEISMASSEQMQGISQVSIAVSQMDGVTQNNAALVEESSSASQSLSEQAHSLRGM
Ci.rod.62-ROD_16161-YP_003365194.1--36H-COG6/1-557       VTDIMLEIAAASDEQSRGIVQVSQAISEMDKVTQQNASLVQEASAAAASLEEQAARLTEA
En.asb.1498-Entas_2095-YP_004828614.1--36H-COG6/1-563    VTDIMLEIAAASDEQSRGIVQVSQAISEMDKVTQQNASLVEEASAAAASLEEQGARLTEA
En.638.865-Ent638_1961-YP_001176688.1--36H-COG6/1-563    VTDIMLEIAAASDEQSRGIVQVSQAISEMDKVTQQNASLVEEASAAAASLEEQGARLTEA
Cr.sak.579-ESA_01710-YP_001437800.1--36H-COG6/1-565      LTDIMLEIAAASDEQRQGIEQVAQAIAEMDNVTQQNASLVEEASAAAVSLEEQAGRLTQA
Cr.tur.6-CTU_22440-YP_003210607.1--36H-COG6/1-565        LTDIMLEIAAASDEQRQGIEQVAQAIAEMDNVTQQNASLVEEASSAAVSLEEQAGRLTQA
Ci.kos.578-CKO_01456-YP_001453025.1--36H-COG6/1-562      VTDIMLEIAAASDEQSRGIVQVSQAISEMDKVTQQNASLVEEASAAAASLEEQAARLTEA
Rahnel.1320-Rahaq_1930-YP_004212674.1--36H-COG6/1-566    VTDIMGEIASASDEQSRGISQVSQAVSEMDGVTQQNASLVQEASAAAASLEEQAARLTQA
Pantoe.297-Pat9b_2403-YP_004116261.1--36H-COG6/1-564     VTDIMSEIAAASDEQSRGIQQVSLAVTEMDNVTQQNASLVEEASSAAASLEDQAGRLTQA
Er.bil.197-EbC_26700-YP_003742048.1--36H-COG6/1-567      VTDIMAEIAAASDEQSRGIQQVSLAVTEMDNVTQQNASLVEEASAAASSLEEQASRLTQA
Pectob.2320-W5S_1760-YP_006282723.1--36H-COG6/1-570      VTDIMGEIASASDEQSRGITQVGQAISEMDSVTQQNAALVQEASAAAASLEEQAALLTRA
Pa.ana.1905-PAJ_1621-YP_005934497.1--36H-COG6/1-568      VTDIMSEIAAASDEQSRGIQQVSQAVTEMDNVTQQNASLVEEASAAAASLEDQAGKLTKA
En.clo.1544-EcWSU1_02120-YP_004951978.1--36H-COG6/1-576  VTDIMLEIAAASDEQSRGIEQVSQAISEMDKVTQQNASLVEEASAAAASLEEQGARLTEA
Ra.aqu.1678-Q7S_09815-YP_005401764.1--36H-COG6/1-566     VTDIMGEIASASDEQSRGISQVSQAVSEMDGVTQQNASLVQEASAAAASLEEQAARLTQA
Ye.pse.585-YPTB2412-YP_070925.1--36H-COG6/1-579          VTDIMGEIASASDEQSRGISQVAQAVSEMDNVTQQNASLVQEASAAAASLEQQAEILTQA
Ye.ent.378-YE2588-YP_001006789.1--36H-COG6/1-579         VTDIMGEIASASDEQSRGISQVAQAVSEMDNVTQQNASLVQEASAAAASLEQQAEILTQA
Di.dad.235-Dda3937_02787-YP_003883649.1--36H-COG6/1-572  VTDIMNEIASASDEQSRGITQVGQAISEMDSVTQQNAALVQQASAAAASLEEQAMVLTRA
Pa.vag.184-Pvag_1800-YP_003931435.1--36H-COG6/1-568      VTDIMSEIAAASDEQSRGIQQVSQAVTEMDNVTQQNASLVEEASAAAASLEDQAGKLTQA
En.bac.2261-D782_2307-YP_007340470.1--36H-COG6/1-558     VTDIMLEIAAASDEQSRGILQVSQAITEMDNVTQQNASLVEEASAAAASLEAQAARLTEA
Pe.atr.485-ECA1683-YP_049784.1--36H-COG6/1-573           VTDIMGEIASASDEQSRGITQVGQAISEMDSVTQQNASLVQEASAAAASLEEQAALLTRA
Pe.car.1139-PC1_2617-YP_003018183.1--36H-COG6/1-573      VTDIMGEIASASDEQSRGISQVGQAISEMDSVTQQNAALVQEASAAAASLEEQAALLTRA
Di.zea.1140-Dd1591_1535-YP_003003868.1--36H-COG6/1-574   VTDIMNEIASASDEQSRGITQVGQAISEMDSVTQQNAALVQQASAAAASLEEQAMVLTRA
Di.zea.1140-Dd1591_3580-YP_003005868.1--36H-COG6/1-572   VTDIMAEISVASQEQSKGIDQVGQAINSIDNVTQQNAALVEQATAAATSLAEQAAGLNEA
Es.col.1836-Y75_p1397-YP_489687.1--36H-COG6/1-546        VTHIMQEIAAASDEQSRGITQVSQAISEMDKVTQQNASLVEEASAAAVSLEEQAARLTEA
Pe.atr.485-ECA3902-YP_051990.1--36H-COG6/1-575           VTDIMGEIASASDEQSRGIGQVSQAVSEMDSATQQNAALVQEASAAAVSLEEQAARLTQA
Sa.bon.1474-SBG_1454-YP_004730321.1--36H-COG6/1-537      VNDIMLEIAAASDEQSRGIVQVSQAISEMDKVTQQNASLVEEASAAAASLEEQAARLTQA
Sa.ent.407-STM1626-NP_460585.1--36H-COG6/1-541           VTDIMLDIAAASDEQSRGIVQVSQAISEMDRVTQQNASLVEEASAAARSLEEQAARLTQA
Pe.car.1139-PC1_3679-YP_003019230.1--36H-COG6/1-567      VTDIMGEIASASDEQSRGIGQVSQAVSEMDSATQQNAALVQEASAAAVSLEEQAARLTQA
Pectob.2320-W5S_4020-YP_006284955.1--36H-COG6/1-575      VTDIMGEIASASDEQSRGIGQVSQAVSEMDSATQQNAALVQEASAAAVSLEEQAARLTQA
Di.zea.1140-Dd1591_0523-YP_003002884.1--36H-COG6/1-568   VTDIMGEIASASDEQSRGIGQVNQAVLEMEGTTQQNAALVQEASAAAASLESQAERLTQA
Di.dad.235-Dda3937_02665-YP_003881279.1--36H-COG6/1-572  VTDIMAEISVASQEQSKGIDQVGQAINSIDNVTQQNAALVEQATAAAASLAEQAAGLNEA
```

```
Pr.mir.1265-PMI1666-YP_002151397.1--36H-COG1/1-568        VSIFKLPGQ-EEKALEKKE--LDAP--PVVQ----A--------AAPLIKPGT-----SE
Di.dad.235-Dda3937_02779-YP_003883640.1--36H-COG1/1-566   VAVFRLSDN-AMAGRPTAI--ASRA--PVAK--------------PVLLATSAS---AEK
Di.zea.1140-Dd1591_1542-YP_003003875.1--36H-COG1/1-566    VAVFRLSER-SMISRPAAV--AAPV--RAAK--------------PALLATSVA---MNK
Pe.atr.485-ECA1691-YP_049792.1--36H-COG1/1-561            VAVFRLSED-TGSFRRTTQ--ATAG--QK----------------PVLLAPSV-----NG
Se.pro.864-Spro_2983-YP_001479212.1--36H-COG1/1-556       VAVFRLKAA-GQDEFKMPV--SSKA-----T------------------VTPV-----LN
Ph.asy.1114-PAU_02685-YP_003041519.1--36H-COG1/1-564      VSLFQLPNL-DDKPQPETK--TEHL--PVVK------------------TLPV-KSNSPV
Pr.stu.1965-S70_18135-YP_006218124.1--36H-COG1/1-561      ISIFRLPAM-KQKELQTTE--PAAR--TISN-V-V-------------KTPDL-----NK
Mo.mor.2189-MU9_1762-YP_007505181.1--36H-COG1/1-561       IAIFRLPAL-KHRDDKHTE--PAAR--TISN-V-V-------------KTPDL-----SK
Pe.car.1139-PC1_2609-YP_003018175.1--36H-COG1/1-561       VAVFRLSED-AGSFRRTTP--AAAG--QK----------------PVLLAPSA-----GG
Xe.nem.162-XNC1_1620-YP_003711881.1--36H-COG1/1-567       VALFQLPEQ-AERMQPEKR--KHEV--LLTK-K-T-------------STPPANKTNSPI
Pectob.2320-W5S_1768-YP_006282731.1--36H-COG1/1-561       VAVFRLSED-AGSFRRTTP--ATTG--QK----------------PVLLAPSV-----KG
Pa.vag.184-Pvag_1723-YP_003931360.1--36H-COG1/1-558       VSVFNIGKE-FVAQAVNKT--TATK--TLQ-------------------SDAP-----LA
Ye.pes.133-YPZ3_2024-YP_003568196.1--36H-COG1/1-557       MSVFVLSVD-NSNSTSDVR--KVKQ--PTQ--------------------EMN-----ST
Ye.pse.585-YPTB2401-YP_070915.1--36H-COG1/1-557           MSVFVLSVD-NSNSTSDVR--KVKQ--PTQ--------------------EMN-----ST
Ye.ent.378-YE2575-YP_001006778.1--36H-COG1/1-557          MSVFILRMD-NSSSKKDVR--KTKQ--PTQ--------------------DKS-----GT
Pa.ana.1905-PAJ_1540-YP_005934416.1--36H-COG1/1-563       VSAFNIGKE-IVTQAVNKT--TAAK--NLR-------------------LESP-----SE
Ph.lum.1262-plu1853-NP_929126.1--36H-COG1/1-564           VSLFQLPNL-DDKSQSEMK--IEHL--PVAK------------------TPPA-KSNPLV
Serrat.1901-SerAS13_3075-YP_006025914.1--36H-COG1/1-555   VAVFRLKAE-GQDEFKMPA--GKAT------------------------VLPV-----LN
Sa.ent.404-STY2128-NP_456485.1--36H-COG1/1-553            VSAFRLASR-PLAVNKPEM----RL--SVD-------------------AQSG-----NT
Ra.aqu.1678-Q7S_08960-YP_005401597.1--36H-COG1/1-564      VAVFTLSQT-ANNNVRQNP--ASSA--RLS-----------------LLTPNL-----SG
Se.mar.2260-D781_2759-YP_007345195.1--36H-COG1/1-555      VAVFRLRDS-GQQAFSAPV--ISKP------------------------AAPS-----IA
Rahnel.1320-Rahaq_1830-YP_004212575.1--36H-COG1/1-564     VAVFTLSQT-ANNNVRQNP--ASSA--RLS-----------------LLTPNL-----SG
Se.ply.1407-SerAS9_3072-YP_004506452.1--36H-COG1/1-555    VAVFRLKAE-GQDEFKMPA--GKAT------------------------VLPV-----LN
Ed.ict.1187-NT01EI_1461-YP_002932882.2--36H-COG1/1-555    VAVFRIEPQ-GEAEPVVRT--APLS--SA---------------------APA-----PV
En.aer.1436-EAE_15535-YP_004593297.1--36H-COG1/1-556      VAVFRLKSE-GQEEYKAPV--SNKT-----A-------------------PAAI-----AT
Xe.bov.105-XBJ1_1923-YP_003467827.1--36H-COG1/1-567       VALFQLPEQ-AERKQSEKR--KHEV--LSTI-K-S-------------PTPPANKTNPPV
Serrat.1408-SerAS12_3073-YP_004501499.1--36H-COG1/1-555   VAVFRLKAE-GQDEFKMPA--GKAT------------------------VLPV-----LN
Er.bil.197-EbC_25350-YP_003741913.1--36H-COG1/1-559       VAVFRIRKE-NVIQAVNTS--KANK--NPL------------------LMPAA-----AP
Er.tas.1011-ETA_14650-YP_001907404.1--36H-COG1/1-554      VAVFRIALQ-QAAPTPELK--RPQL-------------------------GAA-----RS
Sa.ent.407-STM4533-NP_463392.1--36H-COG1/1-553            VAVFRIHQQ-QQRAREVAA--VKTP--------------------------AA-----VS
Es.col.1836-Y75_p4240-YP_492486.1--36H-COG1/1-551         VAVFRIQQQ-Q---RETSA--VVKT-------------------------VTP-----AA
En.bac.2261-D782_3913-YP_007342002.1--36H-COG1/1-553      VAVFHIQQE-KALQEKAEE--RVVA--------------------------VK-----PV
Ci.kos.578-CKO_03442-YP_001454958.1--36H-COG1/1-554       VAVFRIQQE-QQRARDAAA--VKPA-------------------------SAS-----VL
Cr.sak.579-ESA_03402-YP_001439456.1--36H-COG1/1-555       VAVFRISQSAQDKPRSVSP--VQAS-------------------------VAP-----VV
Pantoe.297-Pat9b_1606-YP_004115479.1--36H-COG1/1-558      VAVFNIGKE-FVAQAVNVS--TAPK--LLR-------------------PAAA-----KA
Cr.tur.6-CTU_05670-YP_003208930.1--36H-COG1/1-555         VAVFRISQRAQDKPRSVSP--VLAS-------------------------AAP-----VV
Sa.bon.1474-SBG_3944-YP_004732724.1--36H-COG1/1-553       VAVFRIQQE-QQRAREVAA--IKTT--------------------------AA-----VQ
En.asb.1498-Entas_0547-YP_004827084.1--36H-COG1/1-554     VAVFRIQQE-QMKSREFGS--AKTV-------------------------ATP-----VM
Ci.rod.62-ROD_48461-YP_003368233.1--36H-COG1/1-554        VAVFHIQQE-QQRARNVAN--VKAA-------------------------PKP-----VQ
Ed.tar.1771-ETAF_1249-YP_005698855.1--36H-COG1/1-529      VAVFRIEPQ-GEAEPVVRA--APRS--SA---------------------APA-----PA
En.638.865-Ent638_0513-YP_001175251.1--36H-COG1/1-586     VAVFRIQQE-QVKSREQTG--LKTP-------------------------ATS-----VK
Ed.ict.1187-NT01EI_1462-YP_002932883.1--36H-COG1/1-532    VATFRLGGD-ADAAQSDLQ--GHAN--GMS------------------------------
Ye.ent.378-YE2573-YP_001006777.1--36H-COG1/1-545          VSVFRLEQD-SDNGEGQSA--DRSSKQPAVK-E-----------------IP--------
Ed.tar.1771-ETAF_1250-YP_005698856.1--36H-COG1/1-530      VATFRLSGD-AAQT--DLQ--GQAN--GTS------------------------------
Mo.mor.2189-MU9_1763-YP_007505182.1--36H-COG1/1-524       VAFFELPDN-RGTQSGFAG-----------------------------------------
En.aer.1436-EAE_15540-YP_004593298.1--36H-COG1/1-536      VAVFRLADD-NFVAPETSS---------TVK-E----------------TL---------
En.638.865-Ent638_2456-YP_001177176.1--36H-COG1/1-533     VAVFNVKEQ-VEKVAEAGR--SQTV--PVVS-----------------------------
Se.pro.864-Spro_2982-YP_001479211.1--36H-COG1/1-541       VSVFRLADD-NFTAPANSQ--NAVS--LVVK-E----------------AP---------
Es.col.1836-Y75_p1861-YP_490147.1--36H-COG1/1-533         VAVFTLEEH-EVARHESVQ--LQIA--PVVS-----------------------------
Serrat.1901-SerAS13_3074-YP_006025913.1--36H-COG1/1-541   VSVFRLADD-NFAAPGNNQ--DAVS--PVVK-E----------------AS---------
Ye.pes.133-YPZ3_2023-YP_003568195.1--36H-COG1/1-536       VSVFRLGQG-HDEQSVAGN--SQQS--LATH-----------------------------
Serrat.1408-SerAS12_3072-YP_004501498.1--36H-COG1/1-541   VSVFRLADD-NFAAPGNNQ--DAVS--PVVK-E----------------AS---------
Se.mar.2260-D781_2758-YP_007345194.1--36H-COG1/1-534      VSVFRLADA-HAGASPSDR--LHRH-----K-E------------------TP-------
Ye.pse.585-YPTB2400-YP_070914.1--36H-COG1/1-536           VSVFRLGQG-HDEQSVAGN--SQQS--LATH-----------------------------
Se.ply.1407-SerAS9_3071-YP_004506451.1--36H-COG1/1-541    VSVFRLADD-NFAAPGNNQ--DAVS--PVVK-E----------------AS---------
Pr.stu.1965-S70_18140-YP_006218125.1--36H-COG1/1-521      VAFFEVSET-KAAQVS--------------------------------------------
En.bac.2261-D782_1749-YP_007339930.1--36H-COG1/1-533      VSVFKIEEL-NTFSPERVV--PNAQ--PAVS-----------------------------
En.asb.1498-Entas_2590-YP_004829104.1--36H-COG1/1-533     VAVFNVKEH-VEAVTEVGR--SQAV--PVGT-----------------------------
En.asb.1498-Entas_3672-YP_004830170.1--36H-COG1/1-545     VQKFRLSAQ-EPQTQAAAT--PLKT--PAR------------------------------
Ci.kos.578-CKO_01067-YP_001452646.1--36H-COG1/1-537       VAVFKLEEQ-VVTQQESAP--PQAV--PVVS-----------------------------
En.clo.1544-EcWSU1_02810-YP_004952663.1--36H-COG1/1-538   VAVFNVKEH-VEAVTEVGR--SQAV--PVVS-----------------------------
Sa.ent.407-STM3152-NP_462067.1--36H-COG1/1-547            VQQFRLSAS-EPQQRVTAK--AAPG--VQRM-----------------------------
Pe.atr.485-ECA1332-YP_049438.1--36H-COG1/1-555            VSLFQLSDT-QSALQVAAK--PVRK--AQAI------------------APRA-------
Pe.car.1139-PC1_1208-YP_003016792.1--36H-COG1/1-555       VSLFQLSDT-QSALQVAAK--PVQK--TQAV------------------APRA-------
En.638.865-Ent638_3407-YP_001178118.1--36H-COG1/1-545     VQKFRLSSQ-ESASAVGVS--SPKA--IVR------------------------------
Pa.vag.184-Pvag_1413-YP_003931052.1--36H-COG1/1-554       VSKFRLSSN-RPASPAVSN---TPA--APK-------------------APVL-------
Ph.asy.1114-PAU_02684-YP_003041518.1--36H-COG1/1-544      VAQFELPNN-SENKLENEL--ARTL--RSA------------------------------
Pa.ana.1905-PAJ_1316-YP_005934192.1--36H-COG1/1-556       VAQFRLSSD-APLAPTSTR--KPPS--QPA-------------------SPTT------S
Ci.kos.578-CKO_04394-YP_001455885.1--36H-COG1/1-561       VQQFRLSHN-DAQISPAKT--VSPA--SPRG-----------------------------
Sa.bon.1474-SBG_2750-YP_004731563.1--36H-COG1/1-547       VQQFRLSAD-EPQQSTTAT--AAPG--AQRA-----------------------------
Ra.aqu.1678-Q7S_16025-YP_005402985.1--36H-COG1/1-554      VATFKLKDS-VTSVGSRPG--KSIS--APLL------------------RPDT-------
Rahnel.1320-Rahaq_3177-YP_004213898.1--36H-COG1/1-554     VATFKLKDS-VTSVGSRPG--KSIS--APLL------------------RPDT-------
Pectob.2320-W5S_3114-YP_006284066.1--36H-COG1/1-555       VSLFQLSDS-QSALQVAAK--PVQK--AAVI------------------APRA-------
Ye.ent.378-YE2971-YP_001007160.1--36H-COG1/1-552          VAVFKLQSD-DVRTKSVAK--PRAT--VLTT------------------APIA-------
Er.bil.197-EbC_20190-YP_003741400.1--36H-COG1/1-556       VSKFRLSPV-SAAAAPAPAAYVKPA--GG-----------------------------MT
Ph.lum.1262-plu1854-NP_929127.1--36H-COG1/1-544           VAQFELPNN-SENKLENEL--SLTL--RSA------------------------------
Er.bil.197-EbC_25340-YP_003741912.1--36H-COG1/1-528       VTRFTLDAE-GQPGYPDGI-----------------------------------------
Di.zea.1140-Dd1591_2937-YP_003005238.1--36H-COG1/1-564    VSVFRLTEE-QMAGLSPRR--ERQA--AVKAVS----------------APAA-----KA
Er.tas.1011-ETA_14670-YP_001907406.1--36H-COG1/1-518      VSRFALDAR-HD------------------------------------------------
Sa.ent.404-STY4234-NP_458344.1--36H-COG1/1-547            VAAFRIQKQ-PRREASPTP--LSKG-----------------------LT----------
Sa.ent.407-STM3577-NP_462478.1--36H-COG1/1-547            VAAFRIQKQ-PRREASPTT--LSKG-----------------------LT----------
Sa.bon.1474-SBG_3170-YP_004731982.1--36H-COG1/1-547       VSAFRIRQP-SRRETPPAP--VSKG-----------------------LT----------
En.asb.1498-Entas_2591-YP_004829105.1--36H-COG1/1-555     VSAFRLASK-TTNTANTRA--MHSE--SAT-------------------APAA-----AR
En.638.865-Ent638_1858-YP_001176586.1--36H-COG1/1-552     VAAFRLNAQ-AQNSAPRPS--NLKT--PQV-----------------------------L
Ci.rod.62-ROD_19331-YP_003365490.1--36H-COG1/1-553        VSAFRLAAG-SRTAKAGEE----RA--VAS-------------------GPRA-----AQ
Di.dad.235-Dda3937_01559-YP_003882087.1--36H-COG1/1-566   VSVFRLTED-QASGLPPRR--ERQA--PSKAVA----------------AP-V-----KA
Er.bil.197-EbC_37780-YP_003743156.1--36H-COG1/1-553       VAVFKIGQQ-TLLPAVSQP--VKAI--ATL-----------------------------P
Pantoe.297-Pat9b_2001-YP_004115872.1--36H-COG1/1-558      VAKFRLAPA-AAVTPAATP----PA--TPLL-R----------------RPTL------N
```

```
En.clo.1544-EcWSU1_01962-YP_004951821.1--36H-COG1/1-553  VAAFRLNGK-EKATAPRPA--TVKT--PQL-----------------------------L
En.asb.1498-Entas_1879-YP_004828403.1--36H-COG1/1-554    VAAFRLNAK-DQAAAPRPK--NVKT--PQL-----------------------------L
En.bac.2261-D782_1748-YP_007339929.1--36H-COG1/1-547     VSAFRLASS-ARAVASLKT--ASAP--VVK------------------------------
Es.col.1836-Y75_p1862-YP_490148.1--36H-COG1/1-553        VSAFRLAAS-PLTNKPQTP----SR--PAS-------------------EQPP-----AQ
Cr.tur.6-CTU_33010-YP_003211664.1--36H-COG1/1-555        VAAFKVSRG-VVSTSTRTG--VAPS--ARIE---------------------------PV
Cr.tur.6-CTU_25780-YP_003210941.1--36H-COG1/1-536        VAAFRLASA-RANAPRSEP--AAAP--VSA-------------------SPAL-----AA
En.clo.1544-EcWSU1_00403-YP_004950264.1--36H-COG1/1-549  VAVFKITRN-PVVNAAPVK------------------------------S-------YTP
En.638.865-Ent638_2457-YP_001177177.1--36H-COG1/1-555    VSAFRLTST-PVNTDTSRA--VFAG--TTS-------------------AAVV-----KN
Sa.bon.1474-SBG_1755-YP_004730609.1--36H-COG1/1-553      VSAFRLASR-PLATNKSEA----RL--SAD-------------------IQPG-----NT
Pr.mir.1265-PMI1665-YP_002151396.1--36H-COG1/1-548       VSAFELPDT-DEDGSSHSL--EGNG--LKKA-----------------------------
Cr.sak.579-ESA_01348-YP_001437444.1--36H-COG1/1-556      VAAFRLAAS-RASAPRSVQ--TAAP--VSA-------------------SLTP-----VA
Cr.sak.579-ESA_00560-YP_001436686.1--36H-COG1/1-555      VAAFKVSRG-VVSASARTG--AAPL--VRVE---------------------------PV
Sa.ent.407-STM1919-NP_460876.1--36H-COG1/1-553           VSAFRLASR-PLAVNKPEM----RL--SVN-------------------AQSG-----NT
Ci.kos.578-CKO_01066-YP_001452645.1--36H-COG1/1-552      VSAFRLASR-PLTVKTDL-----NT--TAA-------------------APRT-----AQ
En.asb.1498-Entas_0424-YP_004826963.1--36H-COG1/1-549    VAVFKIARG-QAVKAAPVK------------------------------T-------YVP
Pantoe.297-Pat9b_1273-YP_004115150.1--36H-COG1/1-553     VARFNTGAR-QATAVLVRP--SAPV-----------------------VAPRA-------
Ed.tar.1771-ETAF_2264-YP_005699859.1--36H-COG1/1-555     VSQFRTNDQ-EEKAPVSAK--PVAA--PATP------------------APSA-----KA
Pa.ana.1905-PAJ_3534-YP_005936409.1--36H-COG1/1-553      VAVFNTGRT-PAAGQNVLR--KPSL--KKPL------------------A----------
Pa.vag.184-Pvag_1203-YP_003930842.1--36H-COG1/1-552      VSVFKTGTL-HAAFSAAPV--AKAI--AVPK-----------------------------
Pantoe.297-Pat9b_1605-YP_004115478.1--36H-COG1/1-556     VSVFNIPRV-QAADGVTRH--PQIA--PKV-------------------LD-------TP
Cr.sak.579-ESA_00210-YP_001436348.1--36H-COG1/1-557      VAVFKLNRA-QARAASVAP--QASS--FS--------------------APAP-----VA
Er.tas.1011-ETA_pET460340-YP_001905943.1--36H-COG1/1-543 VSRFRLPES-SESALRSTPAVVKPA--AA-------------------------------
Cr.tur.6-CTU_36610-YP_003212024.1--36H-COG1/1-557        VAVFKLNRA-HSRTASVAP--QAPS--FS--------------------APAP-----AA
Pe.car.1139-PC1_1159-YP_003016743.1--36H-COG1/1-556      VAVFKINQS-VAQEHRAES--APSL--AA--------------------LPTS-----LL
Er.tas.1011-ETA_14640-YP_001907403.1--36H-COG1/1-554     VAVFRITQQ-TVVAVRELK--RPVL------------------------KPAA-----MP
Pa.vag.184-Pvag_1725-YP_003931362.1--36H-COG1/1-556      VSVFKIKRE-ARPTLTKH---LPIA--STV-------------------TKAL-----AP
Ed.ict.1187-NT01EI_3742-YP_002935104.1--36H-COG1/1-525   VALFTLPES-QASRPA--------------------------------------------
En.638.865-Ent638_0380-YP_001175119.1--36H-COG1/1-549    VAVFKINRG-RAVKAAPVK------------------------------T-------FAA
Er.bil.197-EbC_25360-YP_003741914.1--36H-COG1/1-556      VAVFRVRKE-SGFTPAAKA--STVK--RAA-------------------SP-------VA
Er.tas.1011-ETA_16910-YP_001907630.1--36H-COG1/1-543     VSRFRLPES-SESALRSTPAVAKPA--AA-------------------------------
Es.fer.1173-EFER_3055-YP_002384154.1--36H-COG1/1-603     VAVFRIHHE-QQRARESAV--VKTV-------------------------TAP-----AA
Xe.nem.162-XNC1_1619-YP_003711880.1--36H-COG1/1-520      VAKFELPKG-ENSGYSHQK--PSAS--NMTD-EYI-------------------------
En.clo.1544-EcWSU1_02811-YP_004952664.1--36H-COG1/1-559  VSAFRLASL-AGNTVTPQA--TYLA--PAA-------------------AAAA-----TR
Di.dad.235-Dda3937_03500-YP_003882750.1--36H-COG1/1-553  VAAFDLGDT-PTALRSPRA----SA--PALK------------------RPVL-----KA
Xe.bov.105-XBJ1_1924-YP_003467828.1--36H-COG1/1-523      VALFELSES-LDNEYDHQT--SSAS--KMTD-EYI-------------------------
Rahnel.1320-Rahaq_1831-YP_004212576.1--36H-COG1/1-556    VSVFRLATT-DGAGQRRSG--LAQG--LTSR-Q-V-------------AQPAL-----LP
Ra.aqu.1678-Q7S_08965-YP_005401598.1--36H-COG1/1-556     VSVFRLATT-DGAGQRRSG--LAQG--LTSR-Q-V-------------AQPAL-----LP
Cr.sak.579-ESA_01126-YP_001437230.1--36H-COG1/1-514      VQKFRLSA----------------------------------------------------
Pe.atr.485-ECA2712-YP_050803.1--36H-COG1/1-560           VSVFQLSAA-EAPRRPQQR--LAEK--APAA-Q----------------KPML-----LA
En.clo.1544-EcWSU1_00560-YP_004950421.1--36H-COG1/1-597  VAVFRIQQE-QMKAREQAS--AKTA-------------------------AAP-----MM
Di.dad.235-Dda3937_03498-YP_003882752.1--36H-COG1/1-561  VEVFHLGSE-YQTAASRPR--PAGN--MALK------------------RPAL-----SG
Cr.tur.6-CTU_27880-YP_003211151.1--36H-COG1/1-514        VQKFRLSA----------------------------------------------------
Pe.car.1139-PC1_1669-YP_003017246.1--36H-COG1/1-560      VSVFQLSAS-EAHRRPQQR--LAER--APAV-E----------------KPML-----LA
Ra.aqu.1678-Q7S_14450-YP_005402672.1--36H-COG1/1-551     VSVFKIEGR-APLSSASLL--PKNK--TQKN-----------------------------
Ci.kos.578-CKO_03622-YP_001455137.1--36H-COG1/1-517      VAVFKV------------------------------------------------------
Di.zea.1140-Dd1591_2408-YP_003004728.1--36H-COG1/1-557   VAAFDLGDR-HGIAGSARR----VE--PALK------------------RPAA-----AS
Di.zea.1140-Dd1591_2406-YP_003004726.1--36H-COG1/1-559   VEVFHLGTE-HRSTAVNTG--RSGG--MALK------------------RPAL-----KG
Ed.ict.1187-NT01EI_2801-YP_002934203.1--36H-COG1/1-554   VSQFRISEQ-ADKASVSAK--PVAT---APA------------------APSA-----KA
Pe.car.1139-PC1_1384-YP_003016966.1--36H-COG1/1-559      VDVFDLDSD-CDPATSFSR--PAVA--APVH------------------RAVG-----QS
Rahnel.1320-Rahaq_2866-YP_004213595.1--36H-COG1/1-551    VSVFKIEGR-APLSSASLL--PKNK--TQKN-----------------------------
Pe.atr.485-ECA1281-YP_049387.1--36H-COG1/1-556           VEVFKINQA-VAQEHRAAS--ASSL--AA--------------------LPKS-----LL
Pectob.2320-W5S_1886-YP_006282848.1--36H-COG1/1-549      ISVFQLSGS-EAHRRPQQR--LAEK--TPTA-Q----------------KPML-----LA
Pectob.2320-W5S_1696-YP_006282659.1--36H-COG1/1-559      VDVFSLDSD-SDLQPAFSR--SALS--SPAH------------------RAIG-----QS
Di.zea.1140-Dd1591_2407-YP_003004727.1--36H-COG1/1-555   VAAFDLGGS-SGALHSPRV--TPTA--PALK------------------RPSQ-----KA
Pe.atr.485-ECA1509-YP_049611.1--36H-COG1/1-559           VDVFNLDSD-SDQQTAFSR--PAIA--APVH------------------RAVA-----QS
Pa.vag.184-Pvag_1724-YP_003931361.1--36H-COG1/1-520      VSVFKISRA---------------------------------------------------
Pa.ana.1905-PAJ_1541-YP_005934417.1--36H-COG1/1-520      VSVFKISRV---------------------------------------------------
Mo.mor.2189-MU9_1596-YP_007505015.1--36H-COG1/1-519      VQKFRLSAA-D-------------------------------------------------
Pr.mir.1265-PMI2808-YP_002152509.1--36H-COG1/1-563       VNTFKVEQV-AVTSPRSPT--FSAK--PA--------------------EPEI-----TK
Di.zea.1140-Dd1591_0395-YP_003002764.1--36H-COG1/1-556   VAVFRLPGV-ADASATRRVHKPAGK--VAAL----------------------------T
En.clo.1544-EcWSU1_03786-YP_004953635.1--36H-COG1/1-515  VQKFRLSA----------------------------------------------------
Pectob.2320-W5S_3170-YP_006284116.1--36H-COG1/1-556      VEVFKINQS-AAQEPRVAS--APSP--AA--------------------LPKS-----LL
Di.dad.235-Dda3937_02184-YP_003884754.1--36H-COG1/1-556  VSVFKLPGV-ADATAARRELKPAGK--VAAL----------------------------A
Ci.rod.62-ROD_32641-YP_003366748.1--36H-COG1/1-517       VAIFKV------------------------------------------------------
Di.dad.235-Dda3937_03499-YP_003882751.1--36H-COG1/1-561  VTAFDLGDR-QSVLIAPRA----AV--PALK------------------RPAL-----KA
Di.dad.235-Dda3937_00105-YP_003883346.1--36H-COG1/1-561  VSLFRLDTA-ASPSASRAT--PAAI--TPA-------------------APPL-----LA
Pantoe.297-Pat9b_1604-YP_004115477.1--36H-COG1/1-555     VSVFNIPRT-QATGGVTRH--PQIA--PKV-------------------LA-------TP
Er.tas.1011-ETA_14660-YP_001907405.1--36H-COG1/1-560     VAVFQMRKE-NRIAAVNNL--AAPK--TSS------------------LSPPA-----TA
Pr.mir.1265-PMI2809-YP_002152510.1--36H-COG1/1-575       VEQFKTAES-LFSKKSTLT-KPVEK--SANI-G-LSPIKENQQDSRIK-EPKN-----HN
Ed.tar.1771-ETAF_3053-YP_005700648.1--36H-COG1/1-516     VALFTLPGD-STGSDARAT--TA-------------------------------------
Se.pro.864-Spro_1415-YP_001477647.1--36H-COG1/1-546      VSLFKLATH-ASAGVHVQP--QAIA------------------------APKV-----VA
Di.zea.1140-Dd1591_1804-YP_003004134.1--36H-COG1/1-562   VSLFRLHGA-ASLTTEKHR--PAVG--MTAE------------------TPRL-----SV
Di.dad.235-Dda3937_03501-YP_003882749.1--36H-COG1/1-575  VAAFDLGDT-PTALRSPRA----SA--AALK------------------RPVS-----AS
Pantoe.297-Pat9b_0851-YP_004114731.1--36H-COG2/1-524     MSVFKTGHG-MELSVV--------------------------------------------
Pa.vag.184-Pvag_0291-YP_003929953.1--36H-COG2/1-525      MSVFNTGKL-ALQPVLR-------------------------------------------
En.clo.1544-EcWSU1_03886-YP_004953735.1--36H-COG2/1-519  VRVFRVSDR-VLA-----------------------------------------------
Cr.tur.6-CTU_04790-YP_003208842.1--36H-COG2/1-515        IKVFRLSA----------------------------------------------------
Ci.kos.578-CKO_04484-YP_001455975.1--36H-COG2/1-521      VKVFRIRTE-DVPPA---------------------------------------------
Cr.sak.579-ESA_03488-YP_001439539.1--36H-COG2/1-515      IKVFRLSA----------------------------------------------------
Ci.rod.62-ROD_48071-YP_003368199.1--36H-COG2/1-521       VKMFRVSEK-DALAA---------------------------------------------
Sa.bon.1474-SBG_2851-YP_004731664.1--36H-COG2/1-521      VNVFRVREE-DVQPA---------------------------------------------
Sa.ent.407-STM3216-NP_462130.1--36H-COG2/1-521           VNVFRVREE-DTQPA---------------------------------------------
En.asb.1498-Entas_3753-YP_004830248.1--36H-COG2/1-519    VKVFRVNEY-APA-----------------------------------------------
Mo.mor.2189-MU9_3389-YP_007506806.1--36H-COG2/1-521      VGVFQLAGT-PSSVS---------------------------------------------
Sa.ent.404-STY3394-NP_457606.1--36H-COG2/1-522           VNVFRVREE-DTQPA---------------------------------------------
En.638.865-Ent638_3526-YP_001178235.1--36H-COG2/1-519    VKVFRVRED-ALV-----------------------------------------------
Pe.atr.485-ECA1774-YP_049874.1--36H-COG2/1-554           VSVFNLGAS-YKSAALNRK----TE--TPAL-A----------------APKN-----NR
En.638.865-Ent638_2119-YP_001176845.1--36H-COG2/1-549    IRVFVLRDS-EVNSAPVRT----------HV------------------SPAV-----IP
```

```
Pectob.2320-W5S_2813-YP_006283770.1--36H-COG2/1-554 VSVFNLGAS-YKSAALTRK----VE--TPAL-A----------------APKN-----NR
Di.zea.1140-Dd1591_0695-YP_003003055.1--36H-COG2/1-561 VSAFRLTPQ-GNSSATSPARQSAVL--SPAL-P-V--------------RPAV-----TE
Pa.vag.184-Pvag_pPag30079-YP_003729820.1--36H-COG2/1-546 VSVFTLAAV-SPPPVTRQA--IVSV-------------------------PAT-------
Er.bil.197-EbC_38940-YP_003743272.1--36H-COG2/1-526 ISVFKVNDA-HYHGQALA------------------------------------------
Ra.aqu.1678-Q7S_24891-YP_005419232.1--36H-COG2/1-554 ISIFKVSAG-SATAAVRKA--GQTK------------------------SPAL-----LS
En.asb.1498-Entas_2287-YP_004828805.1--36H-COG2/1-513 VAAFRLEQS-AQAA----------------------------------------------
Rahnel.1320-Rahaq_4869-YP_004215574.1--36H-COG2/1-554 ISIFKVSAG-SATAAVRKA--GQTK------------------------SPAL-----LS
Di.dad.235-Dda3937_00027-YP_003883574.1--36H-COG2/1-554 VSVFKLNNN-WSDKGTTRR-QPGGK--ALAL-A-G--------------LPGA-------
Pr.stu.1965-S70_05790-YP_006215722.1--36H-COG2/1-520 VNYFQVSAT-HENRSV--------------------------------------------
En.638.865-Ent638_2100-YP_001176826.1--36H-COG2/1-512 VAAFRLEQN-ARAA----------------------------------------------
Pe.car.1139-PC1_2526-YP_003018093.1--36H-COG2/1-554 VSVFNLGAS-YKSAALSRK----VE--TPAL-S----------------APKN-----NR
Mo.mor.2189-MU9_702-YP_007504121.1--36H-COG2/1-525 MTQFRLGSS-AA------------------------------------------------
Pe.car.1139-PC1_3443-YP_003018995.1--36H-COG2/1-511 VSVFRLLQP-SQQSIHRLA------------------------------SPAA-------
Pa.ana.1905-PAJ_0915-YP_005933791.1--36H-COG2/1-559 VSVFSLSGP-SALSVAHVP--ATVV------------------------KPGF----KTA
Pectob.2320-W5S_3736-YP_006284671.1--36H-COG2/1-510 VSVFRLLHP-SQQDLRRLT------------------------------SAA--------
Pe.atr.485-ECA0183-YP_048310.1--36H-COG2/1-553 VSTFKLLSY-GNGKTASYASAP-TR--TPTL-S-L--------------APAA-------
Rahnel.1320-Rahaq_3107-YP_004213828.1--36H-COG2/1-503 VSVFRLGNE---------------------------------------------------
Pe.car.1139-PC1_4071-YP_003019622.1--36H-COG2/1-553 VSAFKLLSY-GSGKTASYASAP-VR--TPTL-S-L--------------APAA-------
Di.dad.235-Dda3937_03462-YP_003884507.1--36H-COG2/1-563 VSAFRLTPQ-GGNGAPSPVRQPTAL--SHAL-PSA--------------RPNV-----TE
Ra.aqu.1678-Q7S_15665-YP_005402913.1--36H-COG2/1-503 VSVFRLGNE---------------------------------------------------
Pectob.2320-W5S_4505-YP_006285423.1--36H-COG2/1-555 VSAFKLLSY-GNGKAASYASSP-TR--TPTL-S-L--------------ASAT-----TP
Pe.atr.485-ECA3642-YP_051730.1--36H-COG2/1-551 VSQFRLGNG-HQIARTPAA--AASL--TL--------------------RPAL-----AA
Pe.car.1139-PC1_1417-YP_003016999.1--36H-COG2/1-554 VSVFRIAGH-KPSMLQPAT--QALS--LVAM------------------LPVS-----T-
En.asb.1498-Entas_4509-YP_004821569.1--36H-COG2/1-559 VNQFSLREE-IETDATSIM--RAEV--KQE------------------------------
Pa.ana.1905-PAJ_3064-YP_005935940.1--36H-COG2/1-544 VSVFRFNNT-TQQVHSVPT----------------------------LKNPSR-------
Pectob.2320-W5S_1733-YP_006282696.1--36H-COG2/1-535 VSVFKIANY-GLSTPKPAT--QTLS--L-AR------------------LPAA-----A-
Pe.atr.485-ECA4334-YP_052421.1--36H-COG2/1-556 MGVFKLNGI-QTKAPRLTS--QVKQ--PA--------------------APRL-----AL
Di.zea.1140-Dd1591_1602-YP_003003935.1--36H-COG2/1-554 VSVFRLNNN-WSDNSAARF-QPGEK--GLAL-T-G--------------LPGA-------
Pectob.2320-W5S_4506-YP_006285424.1--36H-COG2/1-540 VSAFKIQSY-SRGNAASYASTP-VS--TPML-A-L--------------ANKA-------
Di.zea.1140-Dd1591_1453-YP_003003794.1--36H-COG2/1-532 VGVFRLSDR-ASGARPDTR--VAEQ--ELLL-----------------------------
Pectob.2320-W5S_0106-YP_006281117.1--36H-COG2/1-556 MGVFKISGV-QAPAPRLTS--PMKP--QT--------------------APRL-----AL
Di.zea.1140-Dd1591_0769-YP_003003127.1--36H-COG2/1-505 VSVFRLSNT---------------------------------------LTPRL-----SA
Ra.aqu.1678-Q7S_18035-YP_005403387.1--36H-COG2/1-535 ISAFDISRA-GNLNVTATA-----------------------------------------
Rahnel.1320-Rahaq_3579-YP_004214298.1--36H-COG2/1-535 ISAFDISRA-GNLNVTATA-----------------------------------------
Pe.car.1139-PC1_4072-YP_003019623.1--36H-COG2/1-540 VSAFKIQSY-SGSNGGSYV--P-MR--TPTL-S-L--------------ALAS-----KE
Pe.car.1139-PC1_3464-YP_003019016.1--36H-COG2/1-551 VSQFRLGNS-HQIARTPAA--APSL--AL--------------------QPAL-----AA
Pe.car.1139-PC1_0111-YP_003015709.1--36H-COG2/1-556 MGVFKISGV-QAQAPRLSA--QVKQ--PT--------------------APRL-----AL
Pe.atr.485-ECA0182-YP_048309.1--36H-COG2/1-542 VSAFKIPSY-SHGNAGSYESVP-MS--TPTL-S-L--------------ALAR-----KE
Pectob.2320-W5S_3753-YP_006284688.1--36H-COG2/1-551 VSQFRLDNS-HQIARTPVA--TPTL--AL--------------------QPAL-----AA
Pa.ana.1905-PAJ_2666-YP_005935542.1--36H-COG2/1-514 VEAFRV------------------------------------------------------
Ci.rod.62-ROD_16161-YP_003365194.1--36H-COG6/1-557 VGAFRLQGG-SAAKRTARQ-------------------------------PEV-------
En.asb.1498-Entas_2095-YP_004828614.1--36H-COG6/1-563 VGAFRLNGA-TASRSVASS-------------------------------PAV-----KS
En.638.865-Ent638_1961-YP_001176688.1--36H-COG6/1-563 VGAFRLNGV-APSRSAKPS-------------------------------ALA-----QS
Cr.sak.579-ESA_01710-YP_001437800.1--36H-COG6/1-565 VGAFRLSGA-SAARKNEPQ--PPA-------------------------AQPL-----AP
Cr.tur.6-CTU_22440-YP_003210607.1--36H-COG6/1-565 VGTFRLSAA-SAARKNEPQ--PPA-------------------------AQPL-----AP
Ci.kos.578-CKO_01456-YP_001453025.1--36H-COG6/1-562 VGAFRLQGG-SAIKRAPSV-------------------------------DVV-----AS
Rahnel.1320-Rahaq_1930-YP_004212674.1--36H-COG6/1-566 VAVFRLDGS-TRSAQPQSP--ASSR------------------------ALPG-----PA
Pantoe.297-Pat9b_2403-YP_004116261.1--36H-COG6/1-564 VAAFRLNDN-PGLR-------VTPP--AM--------------------QPAL-----QT
Er.bil.197-EbC_26700-YP_003742048.1--36H-COG6/1-567 VAAFHLSDS-APRR-------VAQP--VHAV------------------QPKA-----AL
Pectob.2320-W5S_1760-YP_006282723.1--36H-COG6/1-570 VATFKLSSH-LSSAPS--P--ARPN--ALAA------------------KSRS-----SL
Pa.ana.1905-PAJ_1621-YP_005934497.1--36H-COG6/1-568 VAAFRLQDT-PAAKPVHQS--DSAP------------------------KAPA-----PV
En.clo.1544-EcWSU1_02120-YP_004951978.1--36H-COG6/1-576 VGAFRLSGA-KPGRSVSSA-------------------------------SAV-----KN
Ra.aqu.1678-Q7S_09815-YP_005401764.1--36H-COG6/1-566 VAVFRLDGS-TRSAQPQSP--ASSR------------------------ALPG-----PA
Ye.pse.585-YPTB2412-YP_070925.1--36H-COG6/1-579 VAVFQLAGH-SSIKEFKSP--VSPP------------------------TGQN-----GS
Ye.ent.378-YE2588-YP_001006789.1--36H-COG6/1-579 VAVFHLNGR-NPAPALKTP--APTS------------------------IGQN-----KT
Di.dad.235-Dda3937_02787-YP_003883649.1--36H-COG6/1-572 VSAFKLTGH-QDKAYAQAP--LAQK--PLLA------------------TSTA-----GL
Pa.vag.184-Pvag_1800-YP_003931435.1--36H-COG6/1-568 VAAFRLQDS-PATKSVAAR--VTSP--AR--------------------TPAL-------
En.bac.2261-D782_2307-YP_007340470.1--36H-COG6/1-558 VGTFRIPSH-SGVKKENVP----------------------------------------A
Pe.atr.485-ECA1683-YP_049784.1--36H-COG6/1-573 VATFKLSSH-LSSGHS-AP--ARPN--ALAA------------------KGRS-----SL
Pe.car.1139-PC1_2617-YP_003018183.1--36H-COG6/1-573 VATFKLSGH-LSGAPS-AP--GRPN--ALAA------------------KDRS-----SL
Di.zea.1140-Dd1591_1535-YP_003003868.1--36H-COG6/1-574 VSAFKLANHLQDTAPTTAS--IMSK--PLLA-T----------------SSTA-----RL
Di.zea.1140-Dd1591_3580-YP_003005868.1--36H-COG6/1-572 VSAFRIDGM-ERQPAMSPA--SRSA--AAHT------------------ALPK-----TL
Es.col.1836-Y75_p1397-YP_489687.1--36H-COG6/1-546 VDVFRLHKH-SVSAEPRGA------------------------------GEPV-------
Pe.atr.485-ECA3902-YP_051990.1--36H-COG6/1-575 VAVFKLSGI-VQQVRSSLP--KSAP--QPRL------------------APAM-----AI
Sa.bon.1474-SBG_1454-YP_004730321.1--36H-COG6/1-537 VGAFRLQGT-DPSFL---------------------------------------------
Sa.ent.407-STM1626-NP_460585.1--36H-COG6/1-541 VDAFRLHDT-GATMRSSFL-----------------------------------------
Pe.car.1139-PC1_3679-YP_003019230.1--36H-COG6/1-567 VAVFKLAGV-AQKLKASLP--KAAP--QPRL------------------APAM-----AI
Pectob.2320-W5S_4020-YP_006284955.1--36H-COG6/1-575 VAVFKLAGV-AQQRRTSLP--KAAP--QPRL------------------APAM-----AL
Di.zea.1140-Dd1591_0523-YP_003002884.1--36H-COG6/1-568 VAVFKLRHR-RENESVRAS--LPSR--PVVQ-A----------------IPAV-----AA
Di.dad.235-Dda3937_02665-YP_003881279.1--36H-COG6/1-572 VSAFRIDGA-ERRPAINIA--ARPA--SAQA------------------ALPK-----AL
```

```
Pr.mir.1265-PMI1666-YP_002151397.1--36H-COG1/1-568       KKK------SSS-VEDPA--NWETF-----
Di.dad.235-Dda3937_02779-YP_003883640.1--36H-COG1/1-566  EQK------ART-SKTTE--NWETF-----
Di.zea.1140-Dd1591_1542-YP_003003875.1--36H-COG1/1-566   ENK------ART-SKTSD--NWETF-----
Pe.atr.485-ECA1691-YP_049792.1--36H-COG1/1-561           GKK------AKE-GSSTD--NWETF-----
Se.pro.864-Spro_2983-YP_001479212.1--36H-COG1/1-556      HKK------MNA-SDPQD--NWETF-----
Ph.asy.1114-PAU_02685-YP_003041519.1--36H-COG1/1-564     LKK------VSN-VEEQS--NWETF-----
Pr.stu.1965-S70_18135-YP_006218124.1--36H-COG1/1-561     ------I-SSKN-HQDDD--NWETF-----
Mo.mor.2189-MU9_1762-YP_007505181.1--36H-COG1/1-561      ------I-SSKN-HQDDD--NWETF-----
Pe.car.1139-PC1_2609-YP_003018175.1--36H-COG1/1-561      GKK------AKE-GLSND--NWETF-----
Xe.nem.162-XNC1_1620-YP_003711881.1--36H-COG1/1-567      LKK------SSD-VEDPS--NWETF-----
Pectob.2320-W5S_1768-YP_006282731.1--36H-COG1/1-561      GQK------AKE-GSSTD--NWETF-----
Pa.vag.184-Pvag_1723-YP_003931360.1--36H-COG1/1-558      VGR------SSA-VRSDD--NWETF-----
Ye.pes.133-YPZ3_2024-YP_003568196.1--36H-COG1/1-557      AKK------ALG-NNLQD--NWETF-----
Ye.pse.585-YPTB2401-YP_070915.1--36H-COG1/1-557          AKK------ALG-NNLQD--NWETF-----
Ye.ent.378-YE2575-YP_001006778.1--36H-COG1/1-557         AKK------ALG-SDLQE--NWETF-----
Pa.ana.1905-PAJ_1540-YP_005934416.1--36H-COG1/1-563      GIR------QPS-PRGDE--NWETF-----
Ph.lum.1262-plu1853-NP_929126.1--36H-COG1/1-564          LKK------ANN-VEEQA--NWETF-----
Serrat.1901-SerAS13_3075-YP_006025914.1--36H-COG1/1-555  HKK------TNA-SDLQD--NWETF-----
Sa.ent.404-STY2128-NP_456485.1--36H-COG1/1-553           -PR------PLA-AGDDA--NWETF-----
Ra.aqu.1678-Q7S_08960-YP_005401597.1--36H-COG1/1-564     SKAG-KG-GAQG-TDLTE--NWETF-----
Se.mar.2260-D781_2759-YP_007345195.1--36H-COG1/1-555     PRK------ASA-GETQD--NWETF-----
Rahnel.1320-Rahaq_1830-YP_004212575.1--36H-COG1/1-564    SKAG-KG-GAQG-TDLTE--NWETF-----
Se.ply.1407-SerAS9_3072-YP_004506452.1--36H-COG1/1-555   HKK------TNA-SDLQD--NWETF-----
Ed.ict.1187-NT01EI_1461-YP_002932882.2--36H-COG1/1-555   KAK------AS--DSSDD--NWETF-----
En.aer.1436-EAE_15535-YP_004593297.1--36H-COG1/1-556     HKK------TSA-SDYQD--NWETF-----
Xe.bov.105-XBJ1_1923-YP_003467827.1--36H-COG1/1-567      LKK------GSN-VEDPS--NWETF-----
Serrat.1408-SerAS12_3073-YP_004501499.1--36H-COG1/1-555  HKK------TNA-SDLQD--NWETF-----
Er.bil.197-EbC_25350-YP_003741913.1--36H-COG1/1-559      RKA------LAA-PTSND--NWETF-----
Er.tas.1011-ETA_14650-YP_001907404.1--36H-COG1/1-554     AKS------VAV-TRGDD--HWETF-----
Sa.ent.407-STM4533-NP_463392.1--36H-COG1/1-553           SPK------AAV-ADGSD--NWETF-----
Es.col.1836-Y75_p4240-YP_492486.1--36H-COG1/1-551        PRK------MAV-ADSEE--NWETF-----
En.bac.2261-D782_3913-YP_007342002.1--36H-COG1/1-553     QRK------AAA-TDAGE--NWETF-----
Ci.kos.578-CKO_03442-YP_001454958.1--36H-COG1/1-554      PRK------AAV-ADSGD--NWETF-----
Cr.sak.579-ESA_03402-YP_001439456.1--36H-COG1/1-555      TAK------PVV-AQGSD--NWETF-----
Pantoe.297-Pat9b_1606-YP_004115479.1--36H-COG1/1-558     LAQ------PAG-TRADD--NWETF-----
Cr.tur.6-CTU_05670-YP_003208930.1--36H-COG1/1-555        TQK------PVV-AQGSD--NWETF-----
Sa.bon.1474-SBG_3944-YP_004732724.1--36H-COG1/1-553      PRK------AAV-ADGSD--NWETF-----
En.asb.1498-Entas_0547-YP_004827084.1--36H-COG1/1-554    ARK------TAT-TDAGD--NWETF-----
Ci.rod.62-ROD_48461-YP_003368233.1--36H-COG1/1-554       TRK------AVT-ADTGD--NWETF-----
Ed.tar.1771-ETAF_1249-YP_005698855.1--36H-COG1/1-529     KAK------ASD-DSSSD--NWETF-----
En.638.865-Ent638_0513-YP_001175251.1--36H-COG1/1-586    ARK------AAA-TTSGE--NWETF-----
Ed.ict.1187-NT01EI_1462-YP_002932883.1--36H-COG1/1-532   ------------------------------
Ye.ent.378-YE2573-YP_001006777.1--36H-COG1/1-545         --------------------DCQTA-----
Ed.tar.1771-ETAF_1250-YP_005698856.1--36H-COG1/1-530     ------------------------------
Mo.mor.2189-MU9_1763-YP_007505182.1--36H-COG1/1-524      ------------------------------
En.aer.1436-EAE_15540-YP_004593298.1--36H-COG1/1-536     --------------------DCQTA-----
En.638.865-Ent638_2456-YP_001177176.1--36H-COG1/1-533    ------------------------------
Se.pro.864-Spro_2982-YP_001479211.1--36H-COG1/1-541      --------------------DCQTA-----
Es.col.1836-Y75_p1861-YP_490147.1--36H-COG1/1-533        ------------------------------
Serrat.1901-SerAS13_3074-YP_006025913.1--36H-COG1/1-541  --------------------DCQTA-----
Ye.pes.133-YPZ3_2023-YP_003568195.1--36H-COG1/1-536      -----------------------------Y
Serrat.1408-SerAS12_3072-YP_004501498.1--36H-COG1/1-541  --------------------DCQTA-----
Se.mar.2260-D781_2758-YP_007345194.1--36H-COG1/1-534     --------------------DCQTS-----
Ye.pse.585-YPTB2400-YP_070914.1--36H-COG1/1-536          -----------------------------Y
Se.ply.1407-SerAS9_3071-YP_004506451.1--36H-COG1/1-541   --------------------DCQTA-----
Pr.stu.1965-S70_18140-YP_006218125.1--36H-COG1/1-521     ------------------------------
En.bac.2261-D782_1749-YP_007339930.1--36H-COG1/1-533     ------------------------------
En.asb.1498-Entas_2590-YP_004829104.1--36H-COG1/1-533    ------------------------------
En.asb.1498-Entas_3672-YP_004830170.1--36H-COG1/1-545    ---------AKP-AATPD--DWVSF-----
Ci.kos.578-CKO_01067-YP_001452646.1--36H-COG1/1-537      ------------------------------
En.clo.1544-EcWSU1_02810-YP_004952663.1--36H-COG1/1-538  ------------------------------
Sa.ent.407-STM3152-NP_462067.1--36H-COG1/1-547           --------ASAP-AQSTD--EWVSF-----
Pe.atr.485-ECA1332-YP_049438.1--36H-COG1/1-555           --------GKAL-PTSSD--NWEKF-----
Pe.car.1139-PC1_1208-YP_003016792.1--36H-COG1/1-555      --------AKAL-PPSND--NWEKF-----
En.638.865-Ent638_3407-YP_001178118.1--36H-COG1/1-545    ---------AKT-TGKTD--EWVSF-----
Pa.vag.184-Pvag_1413-YP_003931052.1--36H-COG1/1-554      NAP------QAK-PADNG--DWVTF-----
Ph.asy.1114-PAU_02684-YP_003041518.1--36H-COG1/1-544     ---------------DSEGAVSQTR-----
Pa.ana.1905-PAJ_1316-YP_005934192.1--36H-COG1/1-556      FQP------KAA-SADNG--DWVTF-----
Ci.kos.578-CKO_04394-YP_001455885.1--36H-COG1/1-561      ---------ASA-AKSGD--EWVAF-----
Sa.bon.1474-SBG_2750-YP_004731563.1--36H-COG1/1-547      --------ALVP-TQAND--DWVAF-----
Ra.aqu.1678-Q7S_16025-YP_005402985.1--36H-COG1/1-554     --------SMLS-AKSAE--NWETF-----
Rahnel.1320-Rahaq_3177-YP_004213898.1--36H-COG1/1-554    --------SMLS-EKSAE--NWETF-----
Pectob.2320-W5S_3114-YP_006284066.1--36H-COG1/1-555      --------GKAL-PASSD--NWEKF-----
Ye.ent.378-YE2971-YP_001007160.1--36H-COG1/1-552         -----------K-VDNNA--NWETF-----
Er.bil.197-EbC_20190-YP_003741400.1--36H-COG1/1-556      RAL---T-AKPA-AASAD--EWVSF-----
Ph.lum.1262-plu1854-NP_929127.1--36H-COG1/1-544          ---------------DSEGAVNQTH-----
Er.bil.197-EbC_25340-YP_003741912.1--36H-COG1/1-528      ----------------------QGI-----
Di.zea.1140-Dd1591_2937-YP_003005238.1--36H-COG1/1-564   LPA---APASSG-KKSND--NWETF-----
Er.tas.1011-ETA_14670-YP_001907406.1--36H-COG1/1-518     ------------------------------
Sa.ent.404-STY4234-NP_458344.1--36H-COG1/1-547           ---------PQP-AAEQA--NWESF-----
Sa.ent.407-STM3577-NP_462478.1--36H-COG1/1-547           ---------PQP-AAEQA--NWESF-----
Sa.bon.1474-SBG_3170-YP_004731982.1--36H-COG1/1-547      ---------PQP-AREQA--NWESF-----
En.asb.1498-Entas_2591-YP_004829105.1--36H-COG1/1-555    -TR------TVT-TGQDE--NWETF-----
En.638.865-Ent638_1858-YP_001176586.1--36H-COG1/1-552    RPA------AA--SASDG--NWETF-----
Ci.rod.62-ROD_19331-YP_003365490.1--36H-COG1/1-553       -KP------SVN-TEPNT--HWETF-----
Di.dad.235-Dda3937_01559-YP_003882087.1--36H-COG1/1-566  LPA---V-KTGG-KNSSD--NREKF-----
Er.bil.197-EbC_37780-YP_003743156.1--36H-COG1/1-553      RKA------LTV-STDQG--NWETF-----
Pantoe.297-Pat9b_2001-YP_004115872.1--36H-COG1/1-558     AAP---A-TQPK-SASAD--EWVSF-----
```

```
En.clo.1544-EcWSU1_01962-YP_004951821.1--36H-COG1/1-553  RPA------TAN-TATDS--NWETF-----
En.asb.1498-Entas_1879-YP_004828403.1--36H-COG1/1-554    RPA------TATASVADG--NWETF-----
En.bac.2261-D782_1748-YP_007339929.1--36H-COG1/1-547     ---------PAV-ARSEE--NWETF-----
Es.col.1836-Y75_p1862-YP_490148.1--36H-COG1/1-553        -PR------LRI-AEQDP--NWETF-----
Cr.tur.6-CTU_33010-YP_003211664.1--36H-COG1/1-555        KPA------LAA-PAGGD--NWETF-----
Cr.tur.6-CTU_25780-YP_003210941.1--36H-COG1/1-536        -AS------KPA-VAGQD--NWETF-----
En.clo.1544-EcWSU1_00403-YP_004950264.1--36H-COG1/1-549  AAK------PVA-ATSEA--NWETF-----
En.638.865-Ent638_2457-YP_001177177.1--36H-COG1/1-555    -TR------ATA-HGKEE--NWETF-----
Sa.bon.1474-SBG_1755-YP_004730609.1--36H-COG1/1-553      -PR------SPA-TVDDA--NWETF-----
Pr.mir.1265-PMI1665-YP_002151396.1--36H-COG1/1-548       --------------------DKKSFFKKTH
Cr.sak.579-ESA_01348-YP_001437444.1--36H-COG1/1-556      -AS------QPA-VASQD--NWETF-----
Cr.sak.579-ESA_00560-YP_001436686.1--36H-COG1/1-555      KPA------LAA-PAGGD--NWETF-----
Sa.ent.407-STM1919-NP_460876.1--36H-COG1/1-553           -PQ------SLA-ARDDA--NWETF-----
Ci.kos.578-CKO_01066-YP_001452645.1--36H-COG1/1-552      -PR------QLA-TEQDT--HWETF-----
En.asb.1498-Entas_0424-YP_004826963.1--36H-COG1/1-549    KTQ------PVA-TATEA--NWETF-----
Pantoe.297-Pat9b_1273-YP_004115150.1--36H-COG1/1-553     -LK------APA-AAGND--NWETF-----
Ed.tar.1771-ETAF_2264-YP_005699859.1--36H-COG1/1-555     ------A-AASR-SDDDA--NWDQF-----
Pa.ana.1905-PAJ_3534-YP_005936409.1--36H-COG1/1-553      -ES------TSA-GLSAE--NWQSF-----
Pa.vag.184-Pvag_1203-YP_003930842.1--36H-COG1/1-552      -RQ------PVA-VTDNE--NWQTF-----
Pantoe.297-Pat9b_1605-YP_004115478.1--36H-COG1/1-556     RKA------LTA-PVSDS--NWETF-----
Cr.sak.579-ESA_00210-YP_001436348.1--36H-COG1/1-557      SLK------AAP-AGGSD--NWETF-----
Er.tas.1011-ETA_pET460340-YP_001905943.1--36H-COG1/1-543 ---------KGA-AGSGD--DWLAF-----
Cr.tur.6-CTU_36610-YP_003212024.1--36H-COG1/1-557        SLK------AAP-AGGSE--NWETF-----
Pe.car.1139-PC1_1159-YP_003016743.1--36H-COG1/1-556      PKP------ASA-GSANA--NWETF-----
Er.tas.1011-ETA_14640-YP_001907403.1--36H-COG1/1-554     -ER------ASS-SVSDS--NWETF-----
Pa.vag.184-Pvag_1725-YP_003931362.1--36H-COG1/1-556      RKA------LTA-PVNDT--NWETF-----
Ed.ict.1187-NT01EI_3742-YP_002935104.1--36H-COG1/1-525   ------------------------------
En.638.865-Ent638_0380-YP_001175119.1--36H-COG1/1-549    KVK------PTA-TVSDG--NWETF-----
Er.bil.197-EbC_25360-YP_003741914.1--36H-COG1/1-556      RKA------SAP-VASDS--NWETF-----
Er.tas.1011-ETA_16910-YP_001907630.1--36H-COG1/1-543     ---------KGA-AGSGD--DWVAF-----
Es.fer.1173-EFER_3055-YP_002384154.1--36H-COG1/1-603     PRK------AAV-ADNSD--NWETF-----
Xe.nem.162-XNC1_1619-YP_003711880.1--36H-COG1/1-520      ---------VS-------------------
En.clo.1544-EcWSU1_02811-YP_004952664.1--36H-COG1/1-559  -KP------AAT-TG-HE--NWETF-----
Di.dad.235-Dda3937_03500-YP_003882750.1--36H-COG1/1-553  SLP--------A-SSSHS--DWETF-----
Xe.bov.105-XBJ1_1924-YP_003467828.1--36H-COG1/1-523      ---------ASQ-VR---------------
Rahnel.1320-Rahaq_1831-YP_004212576.1--36H-COG1/1-556    --------ATAG-AVSAD--NWETF-----
Ra.aqu.1678-Q7S_08965-YP_005401598.1--36H-COG1/1-556     --------ATAG-AVSAD--NWETF-----
Cr.sak.579-ESA_01126-YP_001437230.1--36H-COG1/1-514      ------------------------------
Pe.atr.485-ECA2712-YP_050803.1--36H-COG1/1-560           AAG------GKK-GNAND--NWETF-----
En.clo.1544-EcWSU1_00560-YP_004950421.1--36H-COG1/1-597  ARK------AAT-TDAGE--NWETF-----
Di.dad.235-Dda3937_03498-YP_003882752.1--36H-COG1/1-561  MAR---A-LPPA-RTASDEGSWEKF-----
Cr.tur.6-CTU_27880-YP_003211151.1--36H-COG1/1-514        ------------------------------
Pe.car.1139-PC1_1669-YP_003017246.1--36H-COG1/1-560      AAG------GKK-GNAND--NWETF-----
Ra.aqu.1678-Q7S_14450-YP_005402672.1--36H-COG1/1-551     --------SRVL-TTEDG--GWTKF-----
Ci.kos.578-CKO_03622-YP_001455137.1--36H-COG1/1-517      ------------------------------
Di.zea.1140-Dd1591_2408-YP_003004728.1--36H-COG1/1-557   SPG---V-RSVS-AATQA--DWETF-----
Di.zea.1140-Dd1591_2406-YP_003004726.1--36H-COG1/1-559   AAP---A-LPGK-VSTQD--NWEKF-----
Ed.ict.1187-NT01EI_2801-YP_002934203.1--36H-COG1/1-554   ------A-AASR-NDDDD--NWDQF-----
Pe.car.1139-PC1_1384-YP_003016966.1--36H-COG1/1-559      STP---L-LSAH-GRNGE--GWEKF-----
Rahnel.1320-Rahaq_2866-YP_004213595.1--36H-COG1/1-551    --------SRVL-TTEDG--GWTKF-----
Pe.atr.485-ECA1281-YP_049387.1--36H-COG1/1-556           PKP------TSA-GSSNA--NWETF-----
Pectob.2320-W5S_1886-YP_006282848.1--36H-COG1/1-549      AAS------GKK-GNAND--NWETF-----
Pectob.2320-W5S_1696-YP_006282659.1--36H-COG1/1-559      TTP---L-LSVH-GRHGE--GWEKF-----
Di.zea.1140-Dd1591_2407-YP_003004727.1--36H-COG1/1-555   ALP--------N-RSSHG--GWETF-----
Pe.atr.485-ECA1509-YP_049611.1--36H-COG1/1-559           TTP---L-LSVH-GRHGE--GWEKF-----
Pa.vag.184-Pvag_1724-YP_003931361.1--36H-COG1/1-520      ------------------------------
Pa.ana.1905-PAJ_1541-YP_005934417.1--36H-COG1/1-520      ------------------------------
Mo.mor.2189-MU9_1596-YP_007505015.1--36H-COG1/1-519      ------------------------------
Pr.mir.1265-PMI2808-YP_002152509.1--36H-COG1/1-563       TATTSTI-KSHH-HEADD--NWESF-----
Di.zea.1140-Dd1591_0395-YP_003002764.1--36H-COG1/1-556   NLS---T-ASAP-QKSGD--DWTSF-----
En.clo.1544-EcWSU1_03786-YP_004953635.1--36H-COG1/1-515  ------------------------------
Pectob.2320-W5S_3170-YP_006284116.1--36H-COG1/1-556      PKP------TSA-GSSNA--NWETF-----
Di.dad.235-Dda3937_02184-YP_003884754.1--36H-COG1/1-556  NLS---Q-TSRQ-EKAGD--DWTSF-----
Ci.rod.62-ROD_32641-YP_003366748.1--36H-COG1/1-517       ------------------------------
Di.dad.235-Dda3937_03499-YP_003882751.1--36H-COG1/1-561  SLP--------A-SSSHG--DWETF-----
Di.dad.235-Dda3937_00105-YP_003883346.1--36H-COG1/1-561  SRS--VA-TASA-YNTAS--HWETF-----
Pantoe.297-Pat9b_1604-YP_004115477.1--36H-COG1/1-555     RKA------VTA-PVGDS--NWETF-----
Er.tas.1011-ETA_14660-YP_001907405.1--36H-COG1/1-560     HKA-----LPKA-TGSDE--NWETF-----
Pr.mir.1265-PMI2809-YP_002152510.1--36H-COG1/1-575       -QS------TFK-EDEDD--DWTSF-----
Ed.tar.1771-ETAF_3053-YP_005700648.1--36H-COG1/1-516     ------------------------------
Se.pro.864-Spro_1415-YP_001477647.1--36H-COG1/1-546      -------------DDNGE--NWSRF-----
Di.zea.1140-Dd1591_1804-YP_003004134.1--36H-COG1/1-562   GHK--VA-TAGA-YQETG--NWETF-----
Di.dad.235-Dda3937_03501-YP_003882749.1--36H-COG1/1-575  SLG---A-RPVT-ASSQG--DWETF-----
Pantoe.297-Pat9b_0851-YP_004114731.1--36H-COG2/1-524     ------------------------------
Pa.vag.184-Pvag_0291-YP_003929953.1--36H-COG2/1-525      ------------------------------
En.clo.1544-EcWSU1_03886-YP_004953735.1--36H-COG2/1-519  ------------------------------
Cr.tur.6-CTU_04790-YP_003208842.1--36H-COG2/1-515        ------------------------------
Ci.kos.578-CKO_04484-YP_001455975.1--36H-COG2/1-521      ------------------------------
Cr.sak.579-ESA_03488-YP_001439539.1--36H-COG2/1-515      ------------------------------
Ci.rod.62-ROD_48071-YP_003368199.1--36H-COG2/1-521       ------------------------------
Sa.bon.1474-SBG_2851-YP_004731664.1--36H-COG2/1-521      ------------------------------
Sa.ent.407-STM3216-NP_462130.1--36H-COG2/1-521           ------------------------------
En.asb.1498-Entas_3753-YP_004830248.1--36H-COG2/1-519    ------------------------------
Mo.mor.2189-MU9_3389-YP_007506806.1--36H-COG2/1-521      ------------------------------
Sa.ent.404-STY3394-NP_457606.1--36H-COG2/1-522           ------------------------------
En.638.865-Ent638_3526-YP_001178235.1--36H-COG2/1-519    ------------------------------
Pe.atr.485-ECA1774-YP_049874.1--36H-COG2/1-554           AEK------TSA-KGELA--DWTTF-----
En.638.865-Ent638_2119-YP_001176845.1--36H-COG2/1-549    GKP------AAP-RGSEK--DWQAF-----
```

```
Pectob.2320-W5S_2813-YP_006283770.1--36H-COG2/1-554      AEK------TPA-KGDLA--DWTTF-----
Di.zea.1140-Dd1591_0695-YP_003003055.1--36H-COG2/1-561   RR-------AAA-IGSNS--DWTSF-----
Pa.vag.184-Pvag_pPag30079-YP_003729820.1--36H-COG2/1-546 -VK-----KETP-ADADG--NWTTF-----
Er.bil.197-EbC_38940-YP_003743272.1--36H-COG2/1-526      ------------------------------
Ra.aqu.1678-Q7S_24891-YP_005419232.1--36H-COG2/1-554     NRQ------PVP-AADQG--NWETF-----
En.asb.1498-Entas_2287-YP_004828805.1--36H-COG2/1-513    ------------------------------
Rahnel.1320-Rahaq_4869-YP_004215574.1--36H-COG2/1-554    NRQ------PVP-AADQG--NWETF-----
Di.dad.235-Dda3937_00027-YP_003883574.1--36H-COG2/1-554  --------KDAN-ASDTP--EWAAF-----
Pr.stu.1965-S70_05790-YP_006215722.1--36H-COG2/1-520     ------------------------------
En.638.865-Ent638_2100-YP_001176826.1--36H-COG2/1-512    ------------------------------
Pe.car.1139-PC1_2526-YP_003018093.1--36H-COG2/1-554      AEK------TAA-KGELA--DWTTF-----
Mo.mor.2189-MU9_702-YP_007504121.1--36H-COG2/1-525       ------------------------------
Pe.car.1139-PC1_3443-YP_003018995.1--36H-COG2/1-511      ------------------------------
Pa.ana.1905-PAJ_0915-YP_005933791.1--36H-COG2/1-559      AVK------TRA-EDEDG--SWTHF-----
Pectob.2320-W5S_3736-YP_006284671.1--36H-COG2/1-510      ------------------------------
Pe.atr.485-ECA0183-YP_048310.1--36H-COG2/1-553           ---------AKN-QSNND--NWTTF-----
Rahnel.1320-Rahaq_3107-YP_004213828.1--36H-COG2/1-503    -----------A------------L-----
Pe.car.1139-PC1_4071-YP_003019622.1--36H-COG2/1-553      ---------AKD-QGNNS--DWTSF-----
Di.dad.235-Dda3937_03462-YP_003884507.1--36H-COG2/1-563  KRA------ATT-AGTND--DWTSF-----
Ra.aqu.1678-Q7S_15665-YP_005402913.1--36H-COG2/1-503     -----------A------------L-----
Pectob.2320-W5S_4505-YP_006285423.1--36H-COG2/1-555      ---------AKD-QGNNG--DWTTF-----
Pe.atr.485-ECA3642-YP_051730.1--36H-COG2/1-551           PGK------SGI-SAGEG--DWTSF-----
Pe.car.1139-PC1_1417-YP_003016999.1--36H-COG2/1-554      ---------RAA-AQRPS--DWTNF-----
En.asb.1498-Entas_4509-YP_004821569.1--36H-COG2/1-559    ---------KKV-IGQDT--NWEKF-----
Pa.ana.1905-PAJ_3064-YP_005935940.1--36H-COG2/1-544      -VK------PAQ-PLTHE--GWTHF-----
Pectob.2320-W5S_1733-YP_006282696.1--36H-COG2/1-535      ---------SAS-ASGAS--DWTSF-----
Pe.atr.485-ECA4334-YP_052421.1--36H-COG2/1-556           ------A-SKSG-HTSSD--NWETF-----
Di.zea.1140-Dd1591_1602-YP_003003935.1--36H-COG2/1-554   --------KDAR-VTDTP--EWAAF-----
Pectob.2320-W5S_4506-YP_006285424.1--36H-COG2/1-540      ------------------------------
Di.zea.1140-Dd1591_1453-YP_003003794.1--36H-COG2/1-532   --------------------GYQ-------
Pectob.2320-W5S_0106-YP_006281117.1--36H-COG2/1-556      ------A-SQSG-NASSN--NWETF-----
Di.zea.1140-Dd1591_0769-YP_003003127.1--36H-COG2/1-505   ------------------------------
Ra.aqu.1678-Q7S_18035-YP_005403387.1--36H-COG2/1-535     ---------TR-------------------
Rahnel.1320-Rahaq_3579-YP_004214298.1--36H-COG2/1-535    ---------TR-------------------
Pe.car.1139-PC1_4072-YP_003019623.1--36H-COG2/1-540      ------------------------------
Pe.car.1139-PC1_3464-YP_003019016.1--36H-COG2/1-551      PGK------NVA-TAGEG--DWTSF-----
Pe.car.1139-PC1_0111-YP_003015709.1--36H-COG2/1-556      ------A-SQSG-STSSD--NWETF-----
Pe.atr.485-ECA0182-YP_048309.1--36H-COG2/1-542           ------------------------------
Pectob.2320-W5S_3753-YP_006284688.1--36H-COG2/1-551      PSK------GNA-ATGEG--DWTSF-----
Pa.ana.1905-PAJ_2666-YP_005935542.1--36H-COG2/1-514      ------------------------------
Ci.rod.62-ROD_16161-YP_003365194.1--36H-COG6/1-557       ---------KPV-AVSGD--SWETF-----
En.asb.1498-Entas_2095-YP_004828614.1--36H-COG6/1-563    APL------RPAAAVSGD--NWETF-----
En.638.865-Ent638_1961-YP_001176688.1--36H-COG6/1-563    TPL------RPAVADGGD--NWETF-----
Cr.sak.579-ESA_01710-YP_001437800.1--36H-COG6/1-565      VVP------RAA-LAQSE--HWETF-----
Cr.tur.6-CTU_22440-YP_003210607.1--36H-COG6/1-565        IVP------RAA-LAQSD--HWETF-----
Ci.kos.578-CKO_01456-YP_001453025.1--36H-COG6/1-562      SSP------QPV-TTSGD--NWETF-----
Rahnel.1320-Rahaq_1930-YP_004212674.1--36H-COG6/1-566    SRS------SKG-VNDEP--NWETF-----
Pantoe.297-Pat9b_2403-YP_004116261.1--36H-COG6/1-564     LTP------RVA-TVSND--NWETF-----
Er.bil.197-EbC_26700-YP_003742048.1--36H-COG6/1-567      ATG------RPA-LANSD--NWETF-----
Pectob.2320-W5S_1760-YP_006282723.1--36H-COG6/1-570      ALP------RQT-NTENG--NWETF-----
Pa.ana.1905-PAJ_1621-YP_005934497.1--36H-COG6/1-568      LRP------AAA-TSGNE--NWETF-----
En.clo.1544-EcWSU1_02120-YP_004951978.1--36H-COG6/1-576  APL------RPAPAVSGD--NWETF-----
Ra.aqu.1678-Q7S_09815-YP_005401764.1--36H-COG6/1-566     SRS------SKG-VNDEP--NWETF-----
Ye.pse.585-YPTB2412-YP_070925.1--36H-COG6/1-579          GIN------KKP-TDGSL--NWETF-----
Ye.ent.378-YE2588-YP_001006789.1--36H-COG6/1-579         GGN------KRP-TDDSL--NWETF-----
Di.dad.235-Dda3937_02787-YP_003883649.1--36H-COG6/1-572  GSS------SSA-KPGSD--NWETF-----
Pa.vag.184-Pvag_1800-YP_003931435.1--36H-COG6/1-568      -TP------RPALATGSD--NWETF-----
En.bac.2261-D782_2307-YP_007340470.1--36H-COG6/1-558     TAP------TLA-VAGGD--SWESF-----
Pe.atr.485-ECA1683-YP_049784.1--36H-COG6/1-573           ALP------RQA-NTENG--NWETF-----
Pe.car.1139-PC1_2617-YP_003018183.1--36H-COG6/1-573      ALP------RKT-NTENG--NWETF-----
Di.zea.1140-Dd1591_1535-YP_003003868.1--36H-COG6/1-574   KAP------SGA-KPGSD--NWETF-----
Di.zea.1140-Dd1591_3580-YP_003005868.1--36H-COG6/1-572   SGA------KKA-KTSNE--DWETF-----
Es.col.1836-Y75_p1397-YP_489687.1--36H-COG6/1-546        --------------------SFATV-----
Pe.atr.485-ECA3902-YP_051990.1--36H-COG6/1-575           AG-------SSK-GNSNQ--NWETF-----
Sa.bon.1474-SBG_1454-YP_004730321.1--36H-COG6/1-537      ------------------------------
Sa.ent.407-STM1626-NP_460585.1--36H-COG6/1-541           ------------------------------
Pe.car.1139-PC1_3679-YP_003019230.1--36H-COG6/1-567      AGS------SSK-GSDNQ--NWETF-----
Pectob.2320-W5S_4020-YP_006284955.1--36H-COG6/1-575      ASS------SGK-GSDNQ--NWETF-----
Di.zea.1140-Dd1591_0523-YP_003002884.1--36H-COG6/1-568   IGV------GKG-GRSDQ--NWETF-----
Di.dad.235-Dda3937_02665-YP_003881279.1--36H-COG6/1-572  PGA------KKA-KSSND--GWETF-----
```
