# Supplementary material for: Evolutionary Genomics Suggests That CheV Is an Additional Adaptor for Accommodating Specific Chemoreceptors within the Chemotaxis Signaling Complex
Source: PLoS Comput Biol. 2016 Feb 4;12(2):e1004723. doi: 10.1371/journal.pcbi.1004723 (PMC4742279; doi:10.1371/journal.pcbi.1004723)

S10 Fig. Information content in the putative adaptor binding region of chemoreceptor sequences from COG1 (blue) and COG2 (red). Positions are numbered as in *E. coli* Tar protein. Ser406 (marked with the star) is the only position in this region, which is more conserved in COG2 than in COG1.

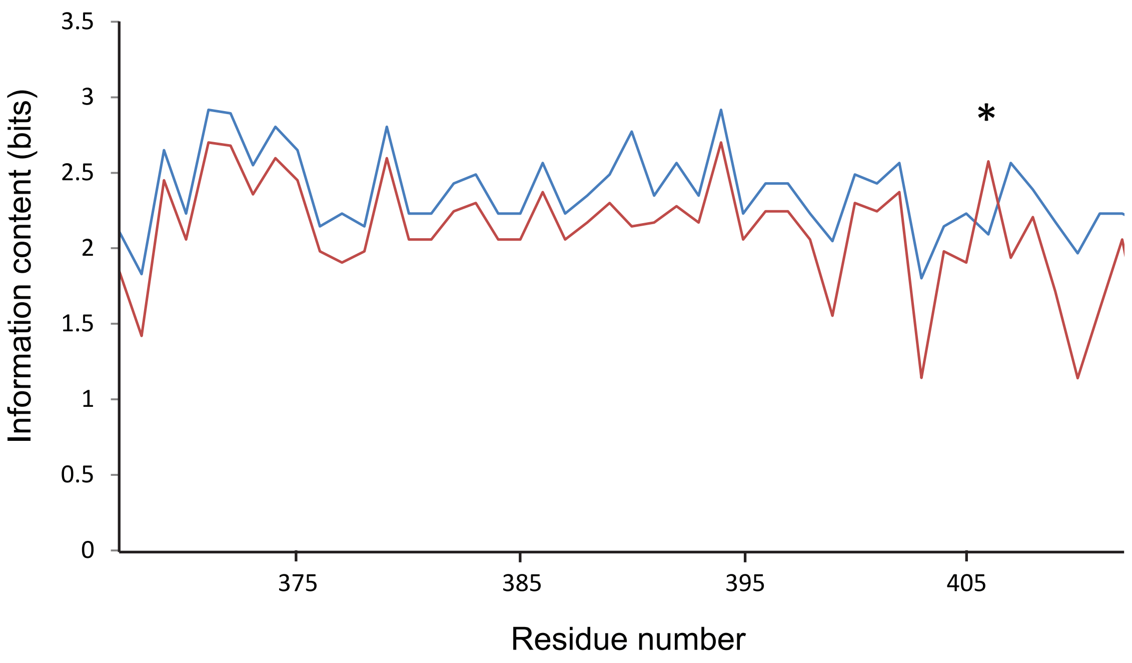

Supplement: S10 Fig — Positions are numbered as in E. coli Tar protein. Ser406 (marked with the star) is the only position in this region, which is more conserved in COG2 than COG1. (PDF) [file pcbi.1004723.s012.pdf]
