# Supplementary material for: Evolutionary Genomics Suggests That CheV Is an Additional Adaptor for Accommodating Specific Chemoreceptors within the Chemotaxis Signaling Complex
Source: PLoS Comput Biol. 2016 Feb 4;12(2):e1004723. doi: 10.1371/journal.pcbi.1004723 (PMC4742279; doi:10.1371/journal.pcbi.1004723)
Supplement: S11 Fig — Each sequence tag contains the first two letters of the genus, the first three letters of the species and the organism id in the MiST database, followed by the locus and accession number. The tag also includes the chemotaxis class for CheV (e.g. F7) (PDF) [file pcbi.1004723.s013.pdf]

S11 Fig. Multiple sequence alignment of CheW proteins and CheV-W domains from the non-redundant *Enterobacteriales* genome set. Each sequence tag contains the first two letters of the genus, the first three letters of the species and the organism id in the MIST database, followed by the locus and accession number. The tag also includes the chemotaxis class for CheV (e.g. F7).

```
Er.tas.1011-ETA_14630-YP_001907402.1       FLVFTLG-------DEEYGIDILKVQEIRGYDQVTRIANTPPFIKGVTNLRGVIVPIIDLRIKFAQPDVEYNDN
Ed.tar.1771-ETAF_1248-YP_005698854.1       FLVFTLG-------DEEYGIDILKVQEIRGYDQVTRIANTPSFIKGVTNLRGVIVPIIDLRVKFSQQDVSYNDN
Ed.ict.1187-NT01EI_1459-YP_002932880.1     FLVFTLG-------DEEYGIDILKVQEIRGYDQVTRIANTPSFIKGVTNLRGVIVPIIDLRVKFSQQDVSYNDN
Di.zea.1140-Dd1591_1541-YP_003003874.1     FLIFTLG-------DEEYGVDILKVQEIRGYDQVTRIANTPGFIKGVTNLRGVIVPIVDLRIKFMQQEVDYDDN
Pe.car.1139-PC1_2610-YP_003018176.1        FLIFTLG-------DEEYGVDILKVQEIRGYDQVTRIANTPSFIKGVTNLRGVIVPIVDLRIKFAKQDVEYDDN
Pectob.2320-W5S_1767-YP_006282730.1        FLIFTLG-------DEEYGVDILKVQEIRGYDQVTRIANTPSFIKGVTNLRGVIVPIVDLRIKFAKQDVEYDDN
Pa.vag.184-Pvag_1726-YP_003931363.1        FLVFTLG-------DEEYGIDILKVQEIRGYDQVTRIANTPEFIRGVTNLRGVIVPIIDLRVKFAQPDVEYNDN
Di.dad.235-Dda3937_02780-YP_003883641.1    FLIFTLG-------DEEYGVDILKVQEIRGYDQVTRIANTPSFIKGVTNLRGVIVPIVDLRIKFMQQEVDYDDN
Pantoe.297-Pat9b_1603-YP_004115476.1       FLVFTLG-------DEEYGIDILKVQEIRGYDQVTRIANTPEFIKGVTNLRGVIVPIIDLRVKFSQPDVDYNEN
Se.pro.864-Spro_2984-YP_001479213.1        FLIFTLG-------NEEYGIDILKVQEIRGYDQVTRIANTPAFIKGVTNLRGVIVPIIDLRVKFAQQDVSYDEN
Ye.pse.585-YPTB2404-YP_070918.1            FLIFTLG-------DEEYGIDILKVQEIRGYDQVTRIANTPAFIKGVTNLRGVIVPIIDLRVKFAQQGVTYNEN
Ye.pes.133-YPZ3_2032-YP_003568204.1        FLIFTLG-------DEEYGIDILKVQEIRGYDQVTRIANTPAFIKGVTNLRGVIVPIIDLRVKFAQQGVTYNEN
En.aer.1436-EAE_15530-YP_004593296.1       FLIFTLG-------NEEYGIDILKVQEIRGYDQVTRIANTPDFIKGVTNLRGVIVPIIDLRVKFAQQGVSYDEN
Pa.ana.1905-PAJ_1542-YP_005934418.1        FLVFTLG-------DEEYGIDILKVQEIRGYDQVTRIANTPDFIRGVTNLRGVIVPIIDLRVKFAQADVEYNEN
Er.bil.197-EbC_25370-YP_003741915.1        FLVFTLG-------DEEYGIDILKVQEIRGYDQVTRIANTPSFIKGVTNLRGVIVPIIDLRIKFAQSDVGYNEN
Ra.aqu.1678-Q7S_08675-YP_005401542.1       FLIFTLG-------DEEYGIDILKVQEIRGYDQVTRIANTPAFIKGVTNLRGVIVPILDLRVKFAQEDVIYNEN
Rahnel.1320-Rahaq_1775-YP_004212520.1      FLIFTLG-------DEEYGIDILKVQEIRGYDQVTRIANTPAFIKGVTNLRGVIVPILDLRVKFAQEDVIYNEN
Sa.bon.1474-SBG_1756-YP_004730610.1        FLVFTLG-------DEEYGIDILKVQEIRGYDQVTRIANTPAFIKGVTNLRGVIVPIVDLRVKFCEGDVEYDDN
Ye.ent.378-YE2576-YP_001006779.1           FLIFTLG-------AEEYGIDILKVQEIRGYDQVTRIANTPAFIKGVTNLRGVIVPIIDLRVKFAQQGVSYNEN
Sa.ent.407-STM1920-NP_460877.1             FLVFTLG-------NEEYGIDILKVQEIRGYDQVTRIANTPAFIKGVTNLRGVIVPIVDLRVKFCEGDVEYDDN
Ci.rod.62-ROD_19401-YP_003365497.1         FLVFTLG-------DEEYGIDILKVQEIRGYDQVTRIANTPAFIKGVTNLRGVIVPIVDLRVKFCEGDVEYNDN
Se.mar.2260-D781_2760-YP_007345196.1       FLIFTLG-------NEEYGIDILKVQEIRGYDQVTRIANTPAFIKGVTNLRGVIVPIIDLRVKFAQQDVSYDEN
En.bac.2261-D782_1746-YP_007339927.1       FLVFTLG-------DEEYGIDILKVQEIRGYDQVTRIANTPAFIKGVTNLRGVIVPIVDLRVKFSQQDVEYNDN
En.638.865-Ent638_2465-YP_001177185.1      FLVFTLG-------DEEYGIDILKVQEIRGYDQVTRIANTPAFIKGVTNLRGVIVPIVDLRVKFSQGDVEYNEN
Pr.mir.1265-PMI1667-YP_002151398.1         -LIFTLG-------NEEYGIDILKVQEIRGYDQVTRIANAPSFIKGVTNLRGVIVPIVDLRIKFSQENVTYNDN
Ci.kos.578-CKO_01062-YP_001452641.1        FLVFTLG-------DEEYGIDILKVQEIRGYDQVTRIANTPSFIKGVTNLRGVIVPIVDLRVKFCQGDVEYNDN
Se.ply.1407-SerAS9_3074-YP_004506454.1     FLIFTLG-------NEEYGIDILKVQEIRGYDQVTRIANTPAFIKGVTNLRGVIVPIIDLRVKFAQQDVSYDEN
Serrat.1408-SerAS12_3075-YP_004501501.1    FLIFTLG-------NEEYGIDILKVQEIRGYDQVTRIANTPAFIKGVTNLRGVIVPIIDLRVKFAQQDVSYDEN
Serrat.1901-SerAS13_3077-YP_006025916.1    FLIFTLG-------NEEYGIDILKVQEIRGYDQVTRIANTPAFIKGVTNLRGVIVPIIDLRVKFAQQDVSYDEN
Pe.atr.485-ECA1690-YP_049791.1             FLIFTLG-------DEEYGVDILKVQEIRGYDQVTRIANTPSFIKGVTNLRGVIVPIVDLRVKFAKLDVEYDDN
Es.fer.1173-EFER_1134-YP_002382296.1       FLVFTLG-------EEEYGIDILKVQEIRGYDQVTRIANTPAFIKGVTNLRGVIVPIVDLRVKFCQGDVDYDDN
En.asb.1498-Entas_2598-YP_004829112.1      FLVFTLG-------DEEYGIDILKVQEIRGYDQVTRIANTPAFIKGVTNLRGVIVPIVDLRVKFSQGDVDYNDN
Cr.sak.579-ESA_01342-YP_001437438.1        FLVFTLG-------DEEYGIDILKVQEIRGYDQVTRIANTPSFIKGVTNLRGVIVPIVDLRVKFEQGDVEYNEN
En.clo.1544-EcWSU1_02819-YP_004952672.1    FLVFTLG-------DEEYGIDILKVQEIRGYDQVTRIANTPAFIKGVTNLRGVIVPIVDLRVKFSQGDVDYNEN
Cr.tur.6-CTU_25840-YP_003210947.1          FLVFTLG-------DEEYGIDILKVQEIRGYDQVTRIANTPSFIKGVTNLRGVIVPIVDLRVKFEQGDVEYNEN
Xe.nem.162-XNC1_1622-YP_003711883.1        YLVFTLG-------DEEYGIEILKVQEIRGYDHVTRIANTPNFIKGITNLRGVIVPIIDLRIKFSQEHVTYNDN
Xe.bov.105-XBJ1_1922-YP_003467826.1        YLVFTLG-------DEEYGIEILKVQEIRGYDQVTRIANTPSFIKGITNLRGVIVPIIDLRIKFSQENVTYNDN
Es.col.1836-Y75_p1863-YP_490149.1          FLVFTLG-------DEEYGIDILKVQEIRGYDQVTRIANTPAFIKGVTNLRGVIVPIVDLRIKFSQVDVDYNDN
Ph.lum.1262-plu1852-NP_929125.1            -LVFTLG-------DEEYGIEILKVQEIRGYDQVTRIANTPAFIKGITNLRGVIVPIIDLRIKFAQETVTYNDN
Ph.asy.1114-PAU_02686-YP_003041520.1       -LVFTLG-------DEEYGIEILKVQEIRGYDQVTRIANTPAFIKGITNLRGVIVPIIDLRIKFAQETVTYNDN
Mo.mor.2189-MU9_1761-YP_007505180.1        -LIFTLG-------EEEYGIEILKVQEIRGYEQVTRIANTPDFIKGVTNLRGVIIPIIDLRVKFSHQNVTYNDN
Sa.ent.404-STY2129-NP_456486.1             FLVFTLG-------NEEYGIDILKVQEIRGYDQVTRIANTPAFIKGVTNLRGVIVPIVDLRVKFCEGDVEYDDN
Pr.stu.1965-S70_18130-YP_006218123.1       -LIFTLG-------EEEYGIEILKVQEIRGYEQVTRIANTPDFIKGVTNLRGVIIPIIDLRVKFSHQNVTYNDN
Di.zea.1140-Dd1591_2613-YP_003004925.1-F7  LLLFRLGSATGEGPSELFGINVFKLREIVPMPTLTKAAGMAPPMLGMINIRGQIIPVIDLPAVVGCAAST--GR
Di.dad.235-Dda3937_02985-YP_003882467.1-F7 LLLFRLGSATGEGPSELFGINVFKLREIVPMPTLTKAAGMIPPMLGMINIRGQIIPVIDLPAVVGCAAST--GR
Ye.ent.378-YE2673-YP_001006864.1-F7        LLLFRLGESQEEQQSELYGINVFKLREIVPMPTLTKAAGMASPMMGMANIRGEIIPVIDLPAIVGCVPKT--GL
Pe.car.1139-PC1_1449-YP_003017031.1-F7     -LLFQLGKSPEGGKSELFGINVFKLREIVPMPTLTKAAGMKSPMLGMVNIRGQIIPVIDLPAVVGSSPDT--GL
En.clo.1544-EcWSU1_03147-YP_004952996.1-F7 LLLFRLGTSLNANKSELFGINVFKLREIVPMPEFTKPAGMKSPLMGMVNIRDQVIPVIDLAAVAGCKPTT--GL
Pectob.2320-W5S_1751-YP_006282714.1-F7     -LLFQLGKSPEGGKSELFGINVFKLREIVPMPTLTKAAGMKSPMLGMVNIRGQIIPVIDLPAVVGSAPET--GL
Er.tas.1011-ETA_19840-YP_001907913.1-F7    -LLFRLGAAYQDEQPELFGINVFKLREIVPMTNITRAAGMKSPLLGMANIRGQLIPVIDLPAVAGCTPET--GL
En.asb.1498-Entas_2991-YP_004829501.1-F7   LLLFRLGTSLNENKSELFGINVFKLREIVPMPEFTKPAGMKSPLMGMVNIRDQVIPVIDLAAVAGCKPTT--GL
Cr.sak.579-ESA_02190-YP_001438275.1-F7     LLLFRLGSANEEEKPELYGINVFKLREIVPMPTITKAAGMQAPLLGMANIRGQIIPVVDLPAVTGCKPTT--GL
Cr.tur.6-CTU_17860-YP_003210149.1-F7       LLLFRLGSASEEEKPELYGINVFKLREIVPMPTITKAAGMQAPLLGMANIRGQIIPVVDLPAVAGCKPTT--GL
En.638.865-Ent638_2819-YP_001177535.1-F7   LLLFRLGTSLNENKSELFGINVFKLREIVPMPEFTKPAGMKSPLMGMVNIRDQVIPVIDLAAVAGCKPAT--GL
Er.bil.197-EbC_11920-YP_003740575.1-F7     LLVFRLGSDLNEEKSELFGINVFKLREIVPMTTITKAAGMKSPLLGMANIRGQFIPVIDLPAVVGCTPTT--GL
Sa.bon.1474-SBG_2102-YP_004730935.1-F7     LLLFRLGTSLHEQKSELFGINVFKLREIVPMPAFTRPAGMKAPLLGMVNIRDQVIPVIDLPAVAGCKPET--GL
Es.fer.1173-EFER_0899-YP_002382071.1-F7    -LLFRLGTSPQAEKSELFGINVFKLREIMPMPQFIRPAGMKSPLLGMVNIRDQVIPVLDLPAIAGCQPQS--GL
Sa.ent.407-STM2314-NP_461256.1-F7          LLLFRLGTSLHEQKSELFGINVFKLREIVPMPAFTRPAGMKAPLLGMVNIRDQVIPVIDLPAVAGCKPET--GL
Pe.atr.485-ECA1568-YP_049670.1-F7          -LLFQLGKSPEGGKSELFGINVFKLREIVPMPTLTKAAGMKSPMLGMVNIRGQIIPVIDLPAVVGSATDT--GL
Ci.kos.578-CKO_00521-YP_001452113.1-F7     LLLFRLGTSLHEQKSELFGINVFKLREIVPMPTFTRPAGMKAPLLGMVNIRDQVIPVIDLPAVAGCKPEN--GL
Sa.ent.404-STY2545-NP_456857.1-F7          LLLFRLGTSLHEQKSELFGINVFKLREIIPMPAFTRPAGMKAPLLGMVNIRDQVIPVIDLPAVAGCKPET--GL
Ci.rod.62-ROD_26761-YP_003366207.1-F7      LLLFRLGTSLHEQKSELFGINVFKLREIVPMPAFTRPAGMKAPLLGMVNIRDQVIPVIDLPAVAGCKAEN--GL
Ra.aqu.1678-Q7S_09145-YP_005401634.1-F7    -LLFRLGTSPDDEQSELFGINVFKLREIVPMPTLTKAAGMVSPMMGMANIRGEIIPVIDLPAVVGCVPKT--GL
Rahnel.1320-Rahaq_1865-YP_004212610.1-F7   -LLFRLGTSPDDEQSELFGINVFKLREIVPMPTLTKAAGMVSPMMGMANIRGEIIPVIDLPAVVGCVPKT--GL
Pantoe.297-Pat9b_0852-YP_004114732.1-F7    LLLFRLGSDQRKGKSELYGINVFKLREIVPMPNITRAAGMQSPLLGMASIRDQFIPVIDLPAVTGCTPET--GL
Pa.ana.1905-PAJ_0248-YP_005933124.1-F7     LLLFRLGSDQLKGKSELFGINVFKLREIVPMPPITKAAGMRSPLLGMASIRGQFIPVIDLPAVAGCVPET--GL
Pa.vag.184-Pvag_0292-YP_003929954.1-F7     LLLFRLGSDQLKGKSELFGINVFKLREIVPMPTITKAAGMRSPLLGMASIRGQFIPVIDLPAVAGCVPET--GL
```

```
Er.tas.1011-ETA_14630-YP_001907402.1       TVVIVLNLEQRVVGIVVDGVSDVLSLTQEQIRPSPEFAVTMSTEYMTGLGALGE------RMLILVDIEKLLSSEE
Ed.tar.1771-ETAF_1248-YP_005698854.1       TVVIVLNFSQRVVGIVVDGVSDVLSLSADQIRPAPEFAVTLSTEYLTGLGALGE------RMLILVDIEKLLSSEE
Ed.ict.1187-NT01EI_1459-YP_002932880.1     TVVIVLNFSQRVVGIVVDGVSDVLSLSADQIRPAPEFAVTLSTEYLTGLGALGE------RMLILVDIEKLLSSEE
Di.zea.1140-Dd1591_1541-YP_003003874.1     TVVIVLNLGQRVVGIVVDGVSDVLSLTADQIRPAPEFAVTLSTEYLTGLGSLGE------RMLILVDIEKLLSSEE
Pe.car.1139-PC1_2610-YP_003018176.1        TVVIVLNLGQRVVGIVVDGVSDVLSLTADQIRPAPEFAVTLSTEYLTGLGSLGE------RMLILVDIEKLLSSEE
Pectob.2320-W5S_1767-YP_006282730.1        TVVIVLNLGQRVVGIVVDGVSDVLSLTADQIRPAPEFAVTLSTEYLTGLGSLGE------RMLILVDIEKLLSSEE
Pa.vag.184-Pvag_1726-YP_003931363.1        TVVIVLNLENRVVGIVVDGVSDVLSLTQDQIRPSPEFAVTMSTEYLTGLGALGD------RMLILVDIEKLLSSD-
Di.dad.235-Dda3937_02780-YP_003883641.1    TVVIVLNLGQRVVGIVVDGVSDVLSLTADQIRPAPEFAVTLSTEYLTGLGSLGE------RMLILVDIEKLLSSEE
Pantoe.297-Pat9b_1603-YP_004115476.1       TVVIVLNLEHRVVGIVVDGVSDVLSLTQDQIRPAPEFAVTMSTEYLTGLGALGE------RMLILVDIEKLLSSEE
Se.pro.864-Spro_2984-YP_001479213.1        TVVIVLNFGQRVVGIVVDGVSDVLSLTTEQIRPAPEFAVTLATEYLTGLGSLGD------RMLILVDIEKLLSSEE
Ye.pse.585-YPTB2404-YP_070918.1            TVVIVLNFGQRVVGIVVDGVSDVLSLTNEQIRPAPEFAVTLATEYLTGLGSLGE------RMLILVDIEKLLSSEE
Ye.pes.133-YPZ3_2032-YP_003568204.1        TVVIVLNFGQRVVGIVVDGVSDVLSLTNEQIRPAPEFAVTLATEYLTGLGSLGE------RMLILVDIEKLLSSEE
En.aer.1436-EAE_15530-YP_004593296.1       TVVIVLNFGQRVVGIVVDGVSDVLSLTAEQIRPAPEFAVTLATEYLTGLGALGE------RMLILVDIEKLLSSEE
Pa.ana.1905-PAJ_1542-YP_005934418.1        TVVIVLNLENRVVGIVVDGVSDVLSLTQEQIRPSPEFAVTMSTEYLTGLGALGD------RMLILVDIEKLLSSEE
Er.bil.197-EbC_25370-YP_003741915.1        TVVIVLNLEQRVVGIVVDGVSDVLSLTQDQIRPSPEFAVTMSTEYLTGLGALGD------RMLILVDIEKLLNSEE
Ra.aqu.1678-Q7S_08675-YP_005401542.1       TVVIVLNFENRVVGIVVDGVSDVLSLSQDQIRPAPEFAVTMSTEYLTGLGSLGD------RMLILVDIEKLLSSEE
Rahnel.1320-Rahaq_1775-YP_004212520.1      TVVIVLNFENRVVGIVVDGVSDVLSLSQDQIRPAPEFAVTMSTEYLTGLGSLGD------RMLILVDIEKLLSSEE
Sa.bon.1474-SBG_1756-YP_004730610.1        TVVIVLNLGQRVVGIVVDGVSDVLSLTAEQIRPAPEFAVTLSTEYLTGLGALGE------RMLILVNIEKLLNSEE
Ye.ent.378-YE2576-YP_001006779.1           TVVIVLNFGQRVVGIVVDGVSDVLSLTAEQIRPAPEFAVTLATEYLTGLGSLGE------RMLILVDIEKLLSSE-
Sa.ent.407-STM1920-NP_460877.1             TVVIVLNLGQRVVGIVVDGVSDVLSLTAEQIRPAPEFAVTLSTEYLTGLGALGE------RMLILVNIEKLLNSEE
Ci.rod.62-ROD_19401-YP_003365497.1         TVVIVLNLGQRVVGIVVDGVSDVLSLTAEQIRPAPEFAVTLSTEYLTGLGALGE------RMLILVNIEKLLNSD-
Se.mar.2260-D781_2760-YP_007345196.1       TVVIVLNLSQRVVGIVVDGVSDVLSLTAEQIRPAPEFAVTLATEYLTGLGALGE------RMLILVDIEKLLSSEE
En.bac.2261-D782_1746-YP_007339927.1       TVVIVLNLGQRVVGIVVDGVSDVLSLAADQIRPAPEFAVTLSTEYLTGLGALGD------RMLILVNIEKLLSSD-
En.638.865-Ent638_2465-YP_001177185.1      TVVIVLNLGQRVVGIVVDGVSDVLSLAADQIRPAPEFAVTLSTEYLTGLGALGD------RMLILVNIEKLLNSD-
Pr.mir.1265-PMI1667-YP_002151398.1         TVVIVVNLLNRIVGIVVDGVSDVLTLKPEQICPAPEFAVTMSTEYLTGLGTLDE------RMLILVDIEKLLNSEE
Ci.kos.578-CKO_01062-YP_001452641.1        TVVIVLNLGQRVVGIVVDGVSDVLSLTAEQIRPAPEFAVTLSTEYLTGLGALGE------RMLILVNIEKLLNSD-
Se.ply.1407-SerAS9_3074-YP_004506454.1     TVVIVLNFGLRVVGIVVDGVSDVLSLTTEQIRPAPEFAVTLATEYLTGLGSLGD------RMLILVDIEKLLSSEE
Serrat.1408-SerAS12_3075-YP_004501501.1    TVVIVLNFGLRVVGIVVDGVSDVLSLTTEQIRPAPEFAVTLATEYLTGLGSLGD------RMLILVDIEKLLSSEE
Serrat.1901-SerAS13_3077-YP_006025916.1    TVVIVLNFGLRVVGIVVDGVSDVLSLTTEQIRPAPEFAVTLATEYLTGLGSLGD------RMLILVDIEKLLSSEE
Pe.atr.485-ECA1690-YP_049791.1             TVVIVLNLGQRVVGIVVDGVSDVLSLTADQIRPAPEFAVTLSTEYLTGLGSLGE------RMLILVDIEKLLSSEE
Es.fer.1173-EFER_1134-YP_002382296.1       TVVIVLNLGQRVVGIVVDGVSDVLSLTAEQIRPAPEFAVTLATEYLTGLGALGD------RMLILVNIEKLLNSEE
En.asb.1498-Entas_2598-YP_004829112.1      TVVIVLNLGQRVVGIVVDGVSDVLSLTSDQIRPAPEFAVTLSTEYLTGLGALGE------RMLILVNIEKLLNSD-
Cr.sak.579-ESA_01342-YP_001437438.1        TVVIVLNFGQRVVGIVVDGVSDVLSLTAEQIRPAPEFAVTLSTEYLTGLGAIGE------RMLILVHIEKLLNSEE
En.clo.1544-EcWSU1_02819-YP_004952672.1    TVVIVLNLGQRVVGIVVDGVSDVLSLTSDQIRPAPEFAVTLSTEYLTGLGALGE------RMLILVNIEKLLNSD-
Cr.tur.6-CTU_25840-YP_003210947.1          TVVIVLNFGQRVVGIVVDGVSDVLSLTAEQIRPAPEFAVTLSTEYLTGLGAIGE------RMLILVHIEKLLNSEE
Xe.nem.162-XNC1_1622-YP_003711883.1        TVVIVLNLLNRIVGIVVDGVSDVLSLKEDQICPAPEFAVTLSTEYLTGLGSLDE------RMLILVDIEKLLSSEE
Xe.bov.105-XBJ1_1922-YP_003467826.1        TVVIVLNLLNRVVGIVVDGVSDVLSLKDDQICPAPEFAVTLSTEYLTGLGSIDE------RMLILVDIEKLLNSEE
Es.col.1836-Y75_p1863-YP_490149.1          TVVIVLNLGQRVVGIVVDGVSDVLSLTAEQIRPAPEFAVTLSTEYLTGLGALGD------RMLILVNIEKLLNSEE
Ph.lum.1262-plu1852-NP_929125.1            TVVIVLNLLNRVVGIVVDGVSDVLSLKAEQICPAPEFAVTLSTEYLTGLGSLDD------RMLILVDIEKLLNSEE
Ph.asy.1114-PAU_02686-YP_003041520.1       TVVIVLNLLNRVVGIVVDGVSDVLSLKAEQICPAPEFAVTLSTEYLTGLGSLDD------RMLILVDIEKLLNSEE
Mo.mor.2189-MU9_1761-YP_007505180.1        TVVIVVNLKDRVVGIVVDGVSDVLVLKDEQIAPAPDFAVTLSTKYLTGLGTIGD------RMLILVDIEKLLSSEE
Sa.ent.404-STY2129-NP_456486.1             TVVIVLNLGQRVVGIVVGGVSDVLSLTAEQIRPAPEFAVTLSTEYLTGLGALGE------RMLILVNIEKLLNSEE
Pr.stu.1965-S70_18130-YP_006218123.1       TVVIVVNLKDRVVGIVVDGVSDVLVLNEEQIAAPPDFAVTLSTKYLTGLGTVGE------RMLILVDIEKLLTSD-
Di.zea.1140-Dd1591_2613-YP_003004925.1-F7  NILLVTEYARSTQAFAVESVDDIVRLDWSQVNTA---EAGVSNTYITSIARLDSDPSSN-RLALVLDVEQILHD--
Di.dad.235-Dda3937_02985-YP_003882467.1-F7 NILLVTEYARSTQAFAVESVDDIVRLDWSQVNTA---EAGVSNTYITSIARLDSDPSSN-RLALVLDVEQILHD--
Ye.ent.378-YE2673-YP_001006864.1-F7        NILLVTEYARSTQAFAVESVDDIVRLEWSQVLAA---DAGVKSRNITSIARLDNDKASN-RLALVLDVEQILYD--
Pe.car.1139-PC1_1449-YP_003017031.1-F7     NILLITEYARSTQAFAVESVDDIVRLEWSQVLAA---EAGVSSSYITSIARLDNDKDSN-RLALVLDVEQIL----
En.clo.1544-EcWSU1_03147-YP_004952996.1-F7 NILLITEYARSVQAFAVESVENIMRLDWKQVHAA---ETAVSGRYITSIACLDEKTDTN-ELAMVLDVEQILYD--
Pectob.2320-W5S_1751-YP_006282714.1-F7     NILLITEYARSTQAFAVESVDDIVRLEWSQVLAA---EAGVSSSYITSIARLDSDKDSN-RLALVLDVEQIL----
Er.tas.1011-ETA_19840-YP_001907913.1-F7    NLLLVTEYARSTQAFAVESVEDIVRLDWNQVRAA---ESGVSTRNITSIACLDN---QN-KMAMVLDVEQILHD--
En.asb.1498-Entas_2991-YP_004829501.1-F7   NILLITEYARSVQAFAVESVENIMRLDWKQVHAA---ETAVSGRYITSIACLDEKTDTN-DLAMVLDVEQILYD--
Cr.sak.579-ESA_02190-YP_001438275.1-F7     NLLLVTEYARSTQAFAVESVENIIRLDWSQVHTA---ESGVSSRNITSIARLDSDNQSN-ELALVLDVEQILYD--
Cr.tur.6-CTU_17860-YP_003210149.1-F7       NLLLVTEYARSTQAFAVESVENIIRLDWSQVHTA---ESGVSSRNITSIARLDSDNQSN-ELALVLDVEQILYD--
En.638.865-Ent638_2819-YP_001177535.1-F7   NILLITEYARSVQAFAVESVENIMRLDWKQVHAA----TAVSGRYITSIACLDENTDTN-DLAMVLDVEQILYD--
Er.bil.197-EbC_11920-YP_003740575.1-F7     NLILVTEYARSTQAFAVESVEDIVRLDWSQVHAA---EAGVSSRNITSIACLNTDKDNNTTMALVLDVEQILHDV-
Sa.bon.1474-SBG_2102-YP_004730935.1-F7     NILLITEYARSVQAFAVESVENIMRLDWKQVHTA---EKAVNGRYITSIACLDEDKETN-NLALVLDVEQILYD--
Es.fer.1173-EFER_0899-YP_002382071.1-F7    NILLITEYARSVQAFAVESVENIVRLDWQHVHTA---EKSINGRYITSVASVDD----T-SLAMVLDVEQILYD--
Sa.ent.407-STM2314-NP_461256.1-F7          NILLITEYARSVQAFAVESVENIMRLDWQQVHTA---EKAVNGRYITSIACLDDNKETN-NLALVLDVEQILYD--
Pe.atr.485-ECA1568-YP_049670.1-F7          NILLITEYARSTQAFAVESVDDIVRLEWSQVLTA---EAGVSSSYITSIARLDTDKDSN-RLALVLDVEQIL----
Ci.kos.578-CKO_00521-YP_001452113.1-F7     NILLITEYARSVQAFAVESVENIMRLDWQQVHTA---EKAVNGRYITSIACLDEDKETN-NLALVLDVEQILYD--
Sa.ent.404-STY2545-NP_456857.1-F7          NILLITEYVRSVQAFAVESVENIMRLDWQQVHTA---EKAVNGRYITSIACLDDNKETN-NLALVLDVEQILYD--
Ci.rod.62-ROD_26761-YP_003366207.1-F7      NILLITEYARSVQAFAVESVENIMRLDWQQVHTA---EKAVNGRYITSIACLDEDKETN-NLALVLDVEQILYD--
Ra.aqu.1678-Q7S_09145-YP_005401634.1-F7    NILLVTEYARSTQAFAVESVDDIVRLEWSQVLAA---EAGVKSRNITSIARLDSDKSSN-RLALVLDVEQILHD--
Rahnel.1320-Rahaq_1865-YP_004212610.1-F7   NILLVTEYARSTQAFAVESVDDIVRLEWSQVLAA---EAGVKSRNITSIARLDSDKSSN-RLALVLDVEQILHD--
Pantoe.297-Pat9b_0852-YP_004114732.1-F7    NLLLVTEYARSTQAFAVESVENIVRLDWNQVHTA---EAGIGSRNITSIACLDSDGESN-NLALVLDVEQILYD--
Pa.ana.1905-PAJ_0248-YP_005933124.1-F7     NLLLVTEYARNTQAFAVESVENIVRLNWSQVHTA---EAGIGGRNITSIACLDDDEQKS-NLAMVLDVEQILYD--
Pa.vag.184-Pvag_0292-YP_003929954.1-F7     NLLLVTEYARNTQAFAVESVENIVRLDWSQVHTA---EAGIGGRNITSIACLDSDKQSN-ELAMVLDVEQILYD--
```
