# Supplementary material for: Evolutionary Genomics Suggests That CheV Is an Additional Adaptor for Accommodating Specific Chemoreceptors within the Chemotaxis Signaling Complex
Source: PLoS Comput Biol. 2016 Feb 4;12(2):e1004723. doi: 10.1371/journal.pcbi.1004723 (PMC4742279; doi:10.1371/journal.pcbi.1004723)
Supplement: S1 Dataset — (PDF) [file pcbi.1004723.s014.pdf]

S1 Dataset. Chemoreceptor COG assignment for 43 *Enterobacteriales* genomes.

Citrobacter koseri ATCC BAA-895
Ci.kos.578-CKO_03442-YP_001454958.1--36H-COG1
Ci.kos.578-CKO_01067-YP_001452646.1--36H-COG1
Ci.kos.578-CKO_04394-YP_001455885.1--36H-COG1
Ci.kos.578-CKO_01066-YP_001452645.1--36H-COG1
Ci.kos.578-CKO_03622-YP_001455137.1--36H-COG1
Ci.kos.578-CKO_04484-YP_001455975.1--36H-COG2
Ci.kos.578-CKO_04485-YP_001455976.1--36H-COG3
Ci.kos.578-CKO_03983-YP_001455491.1--36H-COG3
Ci.kos.578-CKO_01456-YP_001453025.1--36H-COG6
Ci.kos.578-CKO_01287-YP_001452863.1--36H-COG8
Ci.kos.578-CKO_01544-YP_001453112.1--Uncat-COG12
Ci.kos.578-CKO_03716-YP_001455228.1--36H-COG13
Ci.kos.578-CKO_01528-YP_001453097.1--36H-COG13
Ci.kos.578-CKO_03599-YP_001455114.1--36H-COG41

Citrobacter rodentium ICC168
Ci.rod.62-ROD_48461-YP_003368233.1--36H-COG1
Ci.rod.62-ROD_19331-YP_003365490.1--36H-COG1
Ci.rod.62-ROD_32641-YP_003366748.1--36H-COG1
Ci.rod.62-ROD_48071-YP_003368199.1--36H-COG2
Ci.rod.62-ROD_48061-YP_003368198.1--36H-COG3
Ci.rod.62-ROD_16161-YP_003365194.1--36H-COG6
Ci.rod.62-ROD_32921-YP_003366773.1--36H-COG41

Cronobacter sakazakii ATCC BAA-894
Cr.sak.579-ESA_03402-YP_001439456.1--36H-COG1
Cr.sak.579-ESA_01348-YP_001437444.1--36H-COG1
Cr.sak.579-ESA_00560-YP_001436686.1--36H-COG1
Cr.sak.579-ESA_00210-YP_001436348.1--36H-COG1
Cr.sak.579-ESA_01126-YP_001437230.1--36H-COG1
Cr.sak.579-ESA_03488-YP_001439539.1--36H-COG2
Cr.sak.579-ESA_03489-YP_001439540.1--36H-COG3
Cr.sak.579-ESA_pESA3p05464-YP_001440497.1--36H-COG4
Cr.sak.579-ESA_01890-YP_001437980.1--36H-COG4
Cr.sak.579-ESA_00340-YP_001436475.1--36H-COG4
Cr.sak.579-ESA_00239-YP_001436376.1--36H-COG5
Cr.sak.579-ESA_01710-YP_001437800.1--36H-COG6
Cr.sak.579-ESA_pESA3p05527-YP_001440560.1--36H-COG7
Cr.sak.579-ESA_01507-YP_001437601.1--36H-COG8
Cr.sak.579-ESA_00195-YP_001436332.1--36H-COG9
Cr.sak.579-ESA_02212-YP_001438297.1--36H-COG10
Cr.sak.579-ESA_pESA3p05532-YP_001440565.1--36H-COG10
Cr.sak.579-ESA_03817-YP_001439839.1--24H-COG11
Cr.sak.579-ESA_01817-YP_001437907.1--36H-COG12
Cr.sak.579-ESA_02815-YP_001438881.1--36H-COG13
Cr.sak.579-ESA_01914-YP_001438004.1--36H-COG14
Cr.sak.579-ESA_02734-YP_001438802.1--36H-COG17
Cr.sak.579-ESA_00230-YP_001436367.1--36H-COG30
Cr.sak.579-ESA_01321-YP_001437417.1--36H-COG34
Cr.sak.579-ESA_02198-YP_001438283.1--36H-COG77
Cr.sak.579-ESA_00603-YP_001436723.1--Uncat-COG81

Cronobacter turicensis z3032
Cr.tur.6-CTU_05670-YP_003208930.1--36H-COG1
Cr.tur.6-CTU_33010-YP_003211664.1--36H-COG1
Cr.tur.6-CTU_25780-YP_003210941.1--36H-COG1
Cr.tur.6-CTU_36610-YP_003212024.1--36H-COG1
Cr.tur.6-CTU_27880-YP_003211151.1--36H-COG1
Cr.tur.6-CTU_04790-YP_003208842.1--36H-COG2
Cr.tur.6-CTU_04770-YP_003208840.1--36H-COG3
Cr.tur.6-Ctu_1p00330-YP_003212609.1--36H-COG4
Cr.tur.6-CTU_36270-YP_003211990.1--36H-COG5
Cr.tur.6-CTU_22440-YP_003210607.1--36H-COG6
Cr.tur.6-Ctu_1p00800-YP_003212656.1--36H-COG7
Cr.tur.6-CTU_24140-YP_003210777.1--36H-COG8
Cr.tur.6-CTU_36740-YP_003212037.1--36H-COG9
Cr.tur.6-CTU_17110-YP_003210074.1--36H-COG10
Cr.tur.6-Ctu_1p00850-YP_003212661.1--36H-COG10
Cr.tur.6-CTU_21650-YP_003210528.1--36H-COG12
Cr.tur.6-CTU_10630-YP_003209426.1--36H-COG13
Cr.tur.6-CTU_20740-YP_003210437.1--36H-COG14
Cr.tur.6-CTU_29530-YP_003211316.1--36H-COG15
Cr.tur.6-CTU_12280-YP_003209591.1--36H-COG17
Cr.tur.6-CTU_32670-YP_003211630.1--Uncat-COG19
Cr.tur.6-CTU_26050-YP_003210968.1--36H-COG34
Cr.tur.6-CTU_24550-YP_003210818.1--36H-COG43
Cr.tur.6-CTU_36340-YP_003211997.1--36H-COG55
Cr.tur.6-CTU_35450-YP_003211908.1--36H-COG73
Cr.tur.6-CTU_17780-YP_003210141.1--36H-COG78
Cr.tur.6-CTU_20980-YP_003210461.1--36H-COG79
Cr.tur.6-CTU_01830-YP_003208546.1--24H-COG97

Dickeya dadantii 3937
Di.dad.235-Dda3937_02779-YP_003883640.1--36H-COG1
Di.dad.235-Dda3937_01559-YP_003882087.1--36H-COG1
Di.dad.235-Dda3937_03500-YP_003882750.1--36H-COG1
Di.dad.235-Dda3937_03498-YP_003882752.1--36H-COG1
Di.dad.235-Dda3937_02184-YP_003884754.1--36H-COG1
Di.dad.235-Dda3937_03499-YP_003882751.1--36H-COG1
Di.dad.235-Dda3937_00105-YP_003883346.1--36H-COG1
Di.dad.235-Dda3937_03501-YP_003882749.1--36H-COG1
Di.dad.235-Dda3937_00027-YP_003883574.1--36H-COG2
Di.dad.235-Dda3937_03462-YP_003884507.1--36H-COG2
Di.dad.235-Dda3937_00107-YP_003883348.1--36H-COG3
Di.dad.235-Dda3937_00184-YP_003884396.1--36H-COG3
Di.dad.235-Dda3937_03145-YP_003883374.1--36H-COG3
Di.dad.235-Dda3937_00992-YP_003885193.1--36H-COG4
Di.dad.235-Dda3937_02970-YP_003882834.1--36H-COG4
Di.dad.235-Dda3937_01632-YP_003883078.1--36H-COG4
Di.dad.235-Dda3937_00625-YP_003883256.1--36H-COG4
Di.dad.235-Dda3937_00626-YP_003883257.1--36H-COG4
Di.dad.235-Dda3937_03778-YP_003883312.1--36H-COG4
Di.dad.235-Dda3937_02606-YP_003884441.1--36H-COG5
Di.dad.235-Dda3937_00170-YP_003884411.1--36H-COG5
Di.dad.235-Dda3937_02787-YP_003883649.1--36H-COG6
Di.dad.235-Dda3937_02665-YP_003881279.1--36H-COG6
Di.dad.235-Dda3937_03415-YP_003883589.1--36H-COG7
Di.dad.235-Dda3937_01934-YP_003881895.1--36H-COG8
Di.dad.235-Dda3937_02614-YP_003884433.1--36H-COG9
Di.dad.235-Dda3937_01162-YP_003880849.1--36H-COG10
Di.dad.235-Dda3937_01200-YP_003881531.1--24H-COG11
Di.dad.235-Dda3937_00563-YP_003882977.1--36H-COG12
Di.dad.235-Dda3937_02872-YP_003881243.1--36H-COG15
Di.dad.235-Dda3937_02028-YP_003880923.1--36H-COG16
Di.dad.235-Dda3937_01627-YP_003883083.1--Uncat-COG19
Di.dad.235-Dda3937_01293-YP_003881173.1--36H-COG20
Di.dad.235-Dda3937_03899-YP_003881985.1--36H-COG21
Di.dad.235-Dda3937_02025-YP_003880920.1--36H-COG22
Di.dad.235-Dda3937_02635-YP_003884565.1--36H-COG23
Di.dad.235-Dda3937_02026-YP_003880921.1--36H-COG24
Di.dad.235-Dda3937_03142-YP_003883377.1--36H-COG35
Di.dad.235-Dda3937_02163-YP_003884776.1--36H-COG36
Di.dad.235-Dda3937_02027-YP_003880922.1--36H-COG37
Di.dad.235-Dda3937_02023-YP_003880918.1--36H-COG38
Di.dad.235-Dda3937_00404-YP_003884690.1--36H-COG52
Di.dad.235-Dda3937_03722-YP_003883159.1--36H-COG56
Di.dad.235-Dda3937_01861-YP_003883762.1--36H-COG74
Di.dad.235-Dda3937_00072-YP_003882745.1--36H-COG91
Di.dad.235-Dda3937_04369-YP_003883993.1--36H-COG93
Di.dad.235-Dda3937_02134-YP_003884805.1--Uncat-COG99

Dickeya zeae Ech1591
Di.zea.1140-Dd1591_1542-YP_003003875.1--36H-COG1
Di.zea.1140-Dd1591_2937-YP_003005238.1--36H-COG1
Di.zea.1140-Dd1591_2408-YP_003004728.1--36H-COG1
Di.zea.1140-Dd1591_2406-YP_003004726.1--36H-COG1
Di.zea.1140-Dd1591_2407-YP_003004727.1--36H-COG1
Di.zea.1140-Dd1591_0395-YP_003002764.1--36H-COG1
Di.zea.1140-Dd1591_1804-YP_003004134.1--36H-COG1
Di.zea.1140-Dd1591_0695-YP_003003055.1--36H-COG2
Di.zea.1140-Dd1591_1602-YP_003003935.1--36H-COG2
Di.zea.1140-Dd1591_1453-YP_003003794.1--36H-COG2
Di.zea.1140-Dd1591_0769-YP_003003127.1--36H-COG2
Di.zea.1140-Dd1591_1802-YP_003004132.1--36H-COG3
Di.zea.1140-Dd1591_0805-YP_003003163.1--36H-COG3
Di.zea.1140-Dd1591_1779-YP_003004110.1--36H-COG3
Di.zea.1140-Dd1591_0055-YP_003002427.1--36H-COG4
Di.zea.1140-Dd1591_1879-YP_003004208.1--36H-COG4
Di.zea.1140-Dd1591_2086-YP_003004410.1--36H-COG4
Di.zea.1140-Dd1591_2327-YP_003004648.1--36H-COG4
Di.zea.1140-Dd1591_1838-YP_003004168.1--36H-COG4
Di.zea.1140-Dd1591_0765-YP_003003123.1--36H-COG5
Di.zea.1140-Dd1591_0792-YP_003003150.1--36H-COG5
Di.zea.1140-Dd1591_1535-YP_003003868.1--36H-COG6
Di.zea.1140-Dd1591_3580-YP_003005868.1--36H-COG6
Di.zea.1140-Dd1591_0523-YP_003002884.1--36H-COG6
Di.zea.1140-Dd1591_1594-YP_003003927.1--36H-COG7
Di.zea.1140-Dd1591_3111-YP_003005412.1--36H-COG8
Di.zea.1140-Dd1591_0772-YP_003003130.1--36H-COG9
Di.zea.1140-Dd1591_2187-YP_003004509.1--36H-COG12
Di.zea.1140-Dd1591_3613-YP_003005900.1--36H-COG15
Di.zea.1140-Dd1591_4046-YP_003006323.1--36H-COG16
Di.zea.1140-Dd1591_1251-YP_003003594.1--36H-COG17
Di.zea.1140-Dd1591_2083-YP_003004408.1--Uncat-COG19
Di.zea.1140-Dd1591_3723-YP_003006005.1--36H-COG20
Di.zea.1140-Dd1591_3031-YP_003005332.1--36H-COG21
Di.zea.1140-Dd1591_4048-YP_003006325.1--36H-COG22
Di.zea.1140-Dd1591_0641-YP_003003002.1--36H-COG23
Di.zea.1140-Dd1591_1776-YP_003004107.1--36H-COG35
Di.zea.1140-Dd1591_0394-YP_003002763.1--36H-COG36
Di.zea.1140-Dd1591_4047-YP_003006324.1--36H-COG37
Di.zea.1140-Dd1591_4053-YP_003006330.1--36H-COG38
Di.zea.1140-Dd1591_2409-YP_003004729.1--36H-COG46
Di.zea.1140-Dd1591_2271-YP_003004592.1--36H-COG64

Edwardsiella ictaluri 93-146
Ed.ict.1187-NT01EI_1461-YP_002932882.2--36H-COG1
Ed.ict.1187-NT01EI_1462-YP_002932883.1--36H-COG1
Ed.ict.1187-NT01EI_3742-YP_002935104.1--36H-COG1
Ed.ict.1187-NT01EI_2801-YP_002934203.1--36H-COG1
Ed.ict.1187-NT01EI_3772-YP_002935132.1--36H-COG5
Ed.ict.1187-NT01EI_1971-YP_002933382.1--36H-COG8
Ed.ict.1187-NT01EI_3245-YP_002934621.1--36H-COG23
Ed.ict.1187-NT01EI_2581-YP_002933985.1--36H-COG48
Ed.ict.1187-NT01EI_0250-YP_002931728.1--Uncat-COG83

Edwardsiella tarda FL6-60
Ed.tar.1771-ETAF_1249-YP_005698855.1--36H-COG1
Ed.tar.1771-ETAF_1250-YP_005698856.1--36H-COG1
Ed.tar.1771-ETAF_2264-YP_005699859.1--36H-COG1
Ed.tar.1771-ETAF_3053-YP_005700648.1--36H-COG1
Ed.tar.1771-ETAF_3081-YP_005700676.1--36H-COG5
Ed.tar.1771-ETAF_1569-YP_005699169.1--36H-COG8
Ed.tar.1771-ETAF_2605-YP_005700199.1--36H-COG23
Ed.tar.1771-ETAF_2068-YP_005699666.1--36H-COG44

Enterobacter aerogenes KCTC 2190
En.aer.1436-EAE_15535-YP_004593297.1--36H-COG1
En.aer.1436-EAE_15540-YP_004593298.1--36H-COG1

Enterobacter asburiae LF7a
En.asb.1498-Entas_0547-YP_004827084.1--36H-COG1
En.asb.1498-Entas_2590-YP_004829104.1--36H-COG1
En.asb.1498-Entas_3672-YP_004830170.1--36H-COG1
En.asb.1498-Entas_2591-YP_004829105.1--36H-COG1
En.asb.1498-Entas_1879-YP_004828403.1--36H-COG1
En.asb.1498-Entas_0424-YP_004826963.1--36H-COG1
En.asb.1498-Entas_3753-YP_004830248.1--36H-COG2
En.asb.1498-Entas_2287-YP_004828805.1--36H-COG2
En.asb.1498-Entas_4509-YP_004821569.1--36H-COG2
En.asb.1498-Entas_3754-YP_004830249.1--36H-COG3
En.asb.1498-Entas_2280-YP_004828798.1--36H-COG3
En.asb.1498-Entas_0444-YP_004826983.1--36H-COG5
En.asb.1498-Entas_2095-YP_004828614.1--36H-COG6
En.asb.1498-Entas_2434-YP_004828949.1--36H-COG8
En.asb.1498-Entas_0402-YP_004826941.1--36H-COG9
En.asb.1498-Entas_1655-YP_004828181.1--36H-COG10
En.asb.1498-Entas_2185-YP_004828704.1--36H-COG14
En.asb.1498-Entas_2326-YP_004828842.1--36H-COG18
En.asb.1498-Entas_0438-YP_004826977.1--36H-COG30
En.asb.1498-Entas_2153-YP_004828672.1--Uncat-COG98

Enterobacter cloacae EcWSU1
En.clo.1544-EcWSU1_02810-YP_004952663.1--36H-COG1
En.clo.1544-EcWSU1_01962-YP_004951821.1--36H-COG1
En.clo.1544-EcWSU1_00403-YP_004950264.1--36H-COG1
En.clo.1544-EcWSU1_02811-YP_004952664.1--36H-COG1
En.clo.1544-EcWSU1_00560-YP_004950421.1--36H-COG1
En.clo.1544-EcWSU1_03786-YP_004953635.1--36H-COG1
En.clo.1544-EcWSU1_03886-YP_004953735.1--36H-COG2
En.clo.1544-EcWSU1_03888-YP_004953737.1--36H-COG3
En.clo.1544-EcWSU1_02381-YP_004952237.1--36H-COG3
En.clo.1544-EcWSU1_00432-YP_004950293.1--36H-COG5
En.clo.1544-EcWSU1_02120-YP_004951978.1--36H-COG6
En.clo.1544-EcWSU1_02626-YP_004952481.1--36H-COG8
En.clo.1544-EcWSU1_00383-YP_004950244.1--36H-COG9
En.clo.1544-EcWSU1_01715-YP_004951575.1--36H-COG10
En.clo.1544-EcWSU1_02281-YP_004952137.1--36H-COG14
En.clo.1544-EcWSU1_02447-YP_004952303.1--36H-COG18
En.clo.1544-EcWSU1_02183-YP_004952040.1--Uncat-COG65

Enterobacter sp. 638
En.638.865-Ent638_0513-YP_001175251.1--36H-COG1
En.638.865-Ent638_2456-YP_001177176.1--36H-COG1
En.638.865-Ent638_3407-YP_001178118.1--36H-COG1
En.638.865-Ent638_1858-YP_001176586.1--36H-COG1
En.638.865-Ent638_2457-YP_001177177.1--36H-COG1
En.638.865-Ent638_0380-YP_001175119.1--36H-COG1
En.638.865-Ent638_3526-YP_001178235.1--36H-COG2
En.638.865-Ent638_2119-YP_001176845.1--36H-COG2
En.638.865-Ent638_2100-YP_001176826.1--36H-COG2
En.638.865-Ent638_3527-YP_001178236.1--36H-COG3
En.638.865-Ent638_2094-YP_001176820.1--36H-COG3
En.638.865-Ent638_0416-YP_001175155.1--36H-COG5
En.638.865-Ent638_1961-YP_001176688.1--36H-COG6
En.638.865-Ent638_2328-YP_001177048.1--36H-COG8
En.638.865-Ent638_0366-YP_001175105.1--36H-COG9
En.638.865-Ent638_2006-YP_001176733.1--Uncat-COG12
En.638.865-Ent638_2139-YP_001176865.1--36H-COG14
En.638.865-Ent638_2145-YP_001176871.1--36H-COG18

Enterobacteriaceae bacterium strain FGI 57
En.bac.2261-D782_3913-YP_007342002.1--36H-COG1

En.bac.2261-D782_1749-YP_007339930.1--36H-COG1
En.bac.2261-D782_1748-YP_007339929.1--36H-COG1
En.bac.2261-D782_0608-YP_007338849.1--36H-COG3
En.bac.2261-D782_2307-YP_007340470.1--36H-COG6
En.bac.2261-D782_0686-YP_007338925.1--36H-COG40

Erwinia billingiae Eb661
Er.bil.197-EbC_25350-YP_003741913.1--36H-COG1
Er.bil.197-EbC_20190-YP_003741400.1--36H-COG1
Er.bil.197-EbC_25340-YP_003741912.1--36H-COG1
Er.bil.197-EbC_37780-YP_003743156.1--36H-COG1
Er.bil.197-EbC_25360-YP_003741914.1--36H-COG1
Er.bil.197-EbC_38940-YP_003743272.1--36H-COG2
Er.bil.197-EbC_03660-YP_003739757.1--36H-COG3
Er.bil.197-EbC_45290-YP_003743907.1--36H-COG4
Er.bil.197-EbC_44400-YP_003743818.1--36H-COG4
Er.bil.197-EbC_pEb17200840-YP_003739255.1--36H-COG4
Er.bil.197-EbC_04790-YP_003739870.1--36H-COG5
Er.bil.197-EbC_26700-YP_003742048.1--36H-COG6
Er.bil.197-EbC_pEb17200500-YP_003739221.1--36H-COG7
Er.bil.197-EbC_12020-YP_003740585.1--36H-COG7
Er.bil.197-EbC_04620-YP_003739853.1--36H-COG9
Er.bil.197-EbC_13510-YP_003740733.1--36H-COG10
Er.bil.197-EbC_21000-YP_003741481.1--24H-COG11
Er.bil.197-EbC_21960-YP_003741574.1--24H-COG11
Er.bil.197-EbC_13800-YP_003740762.1--36H-COG13
Er.bil.197-EbC_32340-YP_003742612.1--36H-COG13
Er.bil.197-EbC_17630-YP_003741145.1--36H-COG14
Er.bil.197-EbC_43160-YP_003743694.1--Uncat-COG19
Er.bil.197-EbC_23260-YP_003741704.1--36H-COG29
Er.bil.197-EbC_pEb17200750-YP_003739246.1--24H-COG31
Er.bil.197-EbC_pEb17200920-YP_003739263.1--36H-COG42
Er.bil.197-EbC_pEb17200530-YP_003739224.1--36H-COG45
Er.bil.197-EbC_pEb17201160-YP_003739287.1--Uncat-COG49
Er.bil.197-EbC_22510-YP_003741629.1--36H-COG59
Er.bil.197-EbC_pEb10200250-YP_003739082.1--36H-COG90

Erwinia tasmaniensis Et1/99
Er.tas.1011-ETA_14650-YP_001907404.1--36H-COG1
Er.tas.1011-ETA_14670-YP_001907406.1--36H-COG1
Er.tas.1011-ETA_pET460340-YP_001905943.1--36H-COG1
Er.tas.1011-ETA_14640-YP_001907403.1--36H-COG1
Er.tas.1011-ETA_16910-YP_001907630.1--36H-COG1
Er.tas.1011-ETA_14660-YP_001907405.1--36H-COG1
Er.tas.1011-ETA_30660-YP_001908981.1--36H-COG3
Er.tas.1011-ETA_29380-YP_001908853.1--36H-COG5
Er.tas.1011-ETA_29570-YP_001908872.1--36H-COG9
Er.tas.1011-ETA_14120-YP_001907351.1--Uncat-COG12
Er.tas.1011-ETA_33570-YP_001909263.1--36H-COG28
Er.tas.1011-ETA_21980-YP_001908122.1--36H-COG84
Er.tas.1011-ETA_07660-YP_001906710.1--Uncat-COG96

Escherichia coli str. K-12 substr. W3110
Es.col.1836-Y75_p4240-YP_492486.1--36H-COG1
Es.col.1836-Y75_p1861-YP_490147.1--36H-COG1
Es.col.1836-Y75_p1862-YP_490148.1--36H-COG1
Es.col.1836-Y75_p2997-YP_491263.1--36H-COG3
Es.col.1836-Y75_p1397-YP_489687.1--36H-COG6

Escherichia fergusonii ATCC 35469
Es.fer.1173-EFER_3055-YP_002384154.1--36H-COG1
Es.fer.1173-EFER_3024-YP_002384123.1--36H-COG3
Es.fer.1173-EFER_1141-YP_002382303.1--36H-COG47

Morganella morganii subsp. morganii KT
Mo.mor.2189-MU9_1762-YP_007505181.1--36H-COG1
Mo.mor.2189-MU9_1763-YP_007505182.1--36H-COG1
Mo.mor.2189-MU9_1596-YP_007505015.1--36H-COG1
Mo.mor.2189-MU9_3389-YP_007506806.1--36H-COG2
Mo.mor.2189-MU9_702-YP_007504121.1--36H-COG2
Mo.mor.2189-MU9_479-YP_007503898.1--36H-COG3
Mo.mor.2189-MU9_449-YP_007503868.1--36H-COG13
Mo.mor.2189-MU9_478-YP_007503897.1--36H-COG18
Mo.mor.2189-MU9_4-YP_007503423.1--Uncat-COG25

Pantoea ananatis AJ13355
Pa.ana.1905-PAJ_1540-YP_005934416.1--36H-COG1
Pa.ana.1905-PAJ_1316-YP_005934192.1--36H-COG1
Pa.ana.1905-PAJ_3534-YP_005936409.1--36H-COG1
Pa.ana.1905-PAJ_1541-YP_005934417.1--36H-COG1
Pa.ana.1905-PAJ_0915-YP_005933791.1--36H-COG2
Pa.ana.1905-PAJ_3064-YP_005935940.1--36H-COG2
Pa.ana.1905-PAJ_2666-YP_005935542.1--36H-COG2
Pa.ana.1905-PAJ_3456-YP_005936331.1--36H-COG3
Pa.ana.1905-PAJ_1980-YP_005934856.1--36H-COG4
Pa.ana.1905-PAJ_0511-YP_005933387.1--36H-COG4
Pa.ana.1905-PAJ_2734-YP_005935610.1--36H-COG5
Pa.ana.1905-PAJ_1621-YP_005934497.1--36H-COG6
Pa.ana.1905-PAJ_1032-YP_005933908.1--36H-COG7
Pa.ana.1905-PAJ_3633-YP_005936508.1--36H-COG7
Pa.ana.1905-PAJ_p0016-YP_005940982.1--36H-COG7
Pa.ana.1905-PAJ_2753-YP_005935629.1--36H-COG9
Pa.ana.1905-PAJ_2439-YP_005935315.1--36H-COG10
Pa.ana.1905-PAJ_3217-YP_005936093.1--24H-COG11
Pa.ana.1905-PAJ_2484-YP_005935360.1--24H-COG11
Pa.ana.1905-PAJ_2483-YP_005935359.1--24H-COG11
Pa.ana.1905-PAJ_1629-YP_005934505.1--Uncat-COG12
Pa.ana.1905-PAJ_0570-YP_005933446.1--36H-COG13
Pa.ana.1905-PAJ_3465-YP_005936340.1--36H-COG14
Pa.ana.1905-PAJ_0977-YP_005933853.1--Uncat-COG19
Pa.ana.1905-PAJ_1724-YP_005934600.1--36H-COG28
Pa.ana.1905-PAJ_2668-YP_005935544.1--36H-COG29
Pa.ana.1905-PAJ_1490-YP_005934366.1--36H-COG39
Pa.ana.1905-PAJ_3487-YP_005936362.1--36H-COG40
Pa.ana.1905-PAJ_2672-YP_005935548.1--36H-COG42
Pa.ana.1905-PAJ_2482-YP_005935358.1--36H-COG43
Pa.ana.1905-PAJ_3510-YP_005936385.1--36H-COG50
Pa.ana.1905-PAJ_0100-YP_005932976.1--36H-COG51
Pa.ana.1905-PAJ_0806-YP_005933682.1--36H-COG53
Pa.ana.1905-PAJ_p0224-YP_005941189.1--36H-COG60
Pa.ana.1905-PAJ_p0226-YP_005941191.1--36H-COG61
Pa.ana.1905-PAJ_3686-YP_005936561.1--36H-COG66
Pa.ana.1905-PAJ_0838-YP_005933714.1--36H-COG67
Pa.ana.1905-PAJ_1495-YP_005934371.1--36H-COG68
Pa.ana.1905-PAJ_1280-YP_005934156.1--36H-COG85
Pa.ana.1905-PAJ_1210-YP_005934086.1--36H-COG86
Pa.ana.1905-PAJ_p0193-YP_005941158.1--36H-COG88
Pa.ana.1905-PAJ_p0045-YP_005941009.1--36H-COG89

Pantoea sp. At-9b
Pantoe.297-Pat9b_1606-YP_004115479.1--36H-COG1
Pantoe.297-Pat9b_2001-YP_004115872.1--36H-COG1
Pantoe.297-Pat9b_1273-YP_004115150.1--36H-COG1
Pantoe.297-Pat9b_1605-YP_004115478.1--36H-COG1
Pantoe.297-Pat9b_1604-YP_004115477.1--36H-COG1
Pantoe.297-Pat9b_0851-YP_004114731.1--36H-COG2
Pantoe.297-Pat9b_0341-YP_004114223.1--36H-COG3
Pantoe.297-Pat9b_5879-YP_004118591.1--36H-COG4
Pantoe.297-Pat9b_3525-YP_004117371.1--36H-COG5
Pantoe.297-Pat9b_2403-YP_004116261.1--36H-COG6
Pantoe.297-Pat9b_4649-YP_004119174.1--36H-COG7
Pantoe.297-Pat9b_5016-YP_004118863.1--36H-COG7
Pantoe.297-Pat9b_3153-YP_004117006.1--36H-COG7
Pantoe.297-Pat9b_3547-YP_004117393.1--36H-COG9
Pantoe.297-Pat9b_5671-YP_004118383.1--36H-COG10
Pantoe.297-Pat9b_3317-YP_004117167.1--24H-COG11
Pantoe.297-Pat9b_2415-YP_004116273.1--Uncat-COG12
Pantoe.297-Pat9b_1197-YP_004115075.1--36H-COG13
Pantoe.297-Pat9b_4325-YP_004119444.1--36H-COG20
Pantoe.297-Pat9b_0591-YP_004114472.1--36H-COG30

Pantoea vagans C9-1
Pa.vag.184-Pvag_1723-YP_003931360.1--36H-COG1
Pa.vag.184-Pvag_1413-YP_003931052.1--36H-COG1
Pa.vag.184-Pvag_1203-YP_003930842.1--36H-COG1
Pa.vag.184-Pvag_1725-YP_003931362.1--36H-COG1
Pa.vag.184-Pvag_1724-YP_003931361.1--36H-COG1
Pa.vag.184-Pvag_0291-YP_003929953.1--36H-COG2
Pa.vag.184-Pvag_pPag30079-YP_003729820.1--36H-COG2
Pa.vag.184-Pvag_3553-YP_003933121.1--36H-COG3
Pa.vag.184-Pvag_1911-YP_003931544.1--36H-COG4
Pa.vag.184-Pvag_pPag20206-YP_003933542.1--36H-COG4
Pa.vag.184-Pvag_2761-YP_003932369.1--36H-COG5
Pa.vag.184-Pvag_1800-YP_003931435.1--36H-COG6
Pa.vag.184-Pvag_pPag20204-YP_003933540.1--36H-COG7
Pa.vag.184-Pvag_pPag10013-YP_003929523.1--36H-COG7
Pa.vag.184-Pvag_2781-YP_003932389.1--36H-COG9
Pa.vag.184-Pvag_pPag30316-YP_003730057.1--36H-COG10
Pa.vag.184-Pvag_2599-YP_003932208.1--24H-COG11
Pa.vag.184-Pvag_0630-YP_003930281.1--36H-COG13
Pa.vag.184-Pvag_1475-YP_003931114.1--36H-COG14
Pa.vag.184-Pvag_pPag10026-YP_003929536.1--Uncat-COG19
Pa.vag.184-Pvag_1111-YP_003930753.1--36H-COG28
Pa.vag.184-Pvag_pPag10014-YP_003929524.1--36H-COG29
Pa.vag.184-Pvag_1107-YP_003930749.1--24H-COG31
Pa.vag.184-Pvag_pPag20061-YP_003933397.1--36H-COG39
Pa.vag.184-Pvag_pPag30137-YP_003729878.1--36H-COG62
Pa.vag.184-Pvag_pPag10136-YP_003929646.1--36H-COG72
Pa.vag.184-Pvag_pPag30481-YP_003730222.1--36H-COG80
Pa.vag.184-Pvag_pPag20120-YP_003933456.1--Uncat-COG92
Pa.vag.184-Pvag_pPag30081-YP_003729822.1--Uncat-COG95

Pectobacterium atrosepticum SCRI1043
Pe.atr.485-ECA1691-YP_049792.1--36H-COG1
Pe.atr.485-ECA1332-YP_049438.1--36H-COG1
Pe.atr.485-ECA2712-YP_050803.1--36H-COG1
Pe.atr.485-ECA1281-YP_049387.1--36H-COG1
Pe.atr.485-ECA1509-YP_049611.1--36H-COG1
Pe.atr.485-ECA1774-YP_049874.1--36H-COG2
Pe.atr.485-ECA0183-YP_048310.1--36H-COG2
Pe.atr.485-ECA3642-YP_051730.1--36H-COG2
Pe.atr.485-ECA4334-YP_052421.1--36H-COG2
Pe.atr.485-ECA0182-YP_048309.1--36H-COG2
Pe.atr.485-ECA3579-YP_051667.1--36H-COG3
Pe.atr.485-ECA2002-YP_050096.1--36H-COG3
Pe.atr.485-ECA2001-YP_050095.1--36H-COG3
Pe.atr.485-ECA0091-YP_048219.1--36H-COG4
Pe.atr.485-ECA2579-YP_050670.1--36H-COG4
Pe.atr.485-ECA0080-YP_048208.1--36H-COG4
Pe.atr.485-ECA3592-YP_051680.1--36H-COG5
Pe.atr.485-ECA3624-YP_051712.1--36H-COG5
Pe.atr.485-ECA1683-YP_049784.1--36H-COG6
Pe.atr.485-ECA3902-YP_051990.1--36H-COG6
Pe.atr.485-ECA3245-YP_051334.1--36H-COG7
Pe.atr.485-ECA1105-YP_049211.1--36H-COG8
Pe.atr.485-ECA3615-YP_051703.1--36H-COG9
Pe.atr.485-ECA0436-YP_048554.1--36H-COG15
Pe.atr.485-ECA4332-YP_052419.1--36H-COG16
Pe.atr.485-ECA4120-YP_052207.1--36H-COG16
Pe.atr.485-ECA0434-YP_048552.1--36H-COG17
Pe.atr.485-ECA0390-YP_048516.1--36H-COG20
Pe.atr.485-ECA2530-YP_050621.1--36H-COG21
Pe.atr.485-ECA4333-YP_052420.1--36H-COG22
Pe.atr.485-ECA4335-YP_052422.1--36H-COG24
Pe.atr.485-ECA3838-YP_051926.1--36H-COG26
Pe.atr.485-ECA2061-YP_050156.1--36H-COG27
Pe.atr.485-ECA2580-YP_050671.1--36H-COG33
Pe.atr.485-ECA2226-YP_050320.1--36H-COG63
Pe.atr.485-ECA2317-YP_050412.1--36H-COG94

Pectobacterium carotovorum subsp. carotovorum PC1
Pe.car.1139-PC1_2609-YP_003018175.1--36H-COG1
Pe.car.1139-PC1_1208-YP_003016792.1--36H-COG1
Pe.car.1139-PC1_1159-YP_003016743.1--36H-COG1
Pe.car.1139-PC1_1669-YP_003017246.1--36H-COG1
Pe.car.1139-PC1_1384-YP_003016966.1--36H-COG1
Pe.car.1139-PC1_2526-YP_003018093.1--36H-COG2
Pe.car.1139-PC1_3443-YP_003018995.1--36H-COG2
Pe.car.1139-PC1_4071-YP_003019622.1--36H-COG2
Pe.car.1139-PC1_1417-YP_003016999.1--36H-COG2
Pe.car.1139-PC1_4072-YP_003019623.1--36H-COG2
Pe.car.1139-PC1_3464-YP_003019016.1--36H-COG2
Pe.car.1139-PC1_0111-YP_003015709.1--36H-COG2
Pe.car.1139-PC1_3399-YP_003018951.1--36H-COG3
Pe.car.1139-PC1_2292-YP_003017866.1--36H-COG3
Pe.car.1139-PC1_2293-YP_003017867.1--36H-COG3
Pe.car.1139-PC1_4161-YP_003019711.1--36H-COG4
Pe.car.1139-PC1_4172-YP_003019722.1--36H-COG4
Pe.car.1139-PC1_3412-YP_003018964.1--36H-COG5
Pe.car.1139-PC1_3447-YP_003018999.1--36H-COG5
Pe.car.1139-PC1_2617-YP_003018183.1--36H-COG6
Pe.car.1139-PC1_3679-YP_003019230.1--36H-COG6
Pe.car.1139-PC1_3039-YP_003018598.1--36H-COG7
Pe.car.1139-PC1_1003-YP_003016588.1--36H-COG8
Pe.car.1139-PC1_3437-YP_003018989.1--36H-COG9
Pe.car.1139-PC1_2086-YP_003017662.1--36H-COG10
Pe.car.1139-PC1_0007-YP_003015606.1--36H-COG10
Pe.car.1139-PC1_0416-YP_003016008.1--36H-COG15
Pe.car.1139-PC1_0113-YP_003015711.1--36H-COG16
Pe.car.1139-PC1_3910-YP_003019461.1--36H-COG16
Pe.car.1139-PC1_0413-YP_003016005.1--36H-COG17
Pe.car.1139-PC1_0380-YP_003015974.1--36H-COG20
Pe.car.1139-PC1_1797-YP_003017374.1--36H-COG21
Pe.car.1139-PC1_0112-YP_003015710.1--36H-COG22
Pe.car.1139-PC1_0110-YP_003015708.1--36H-COG24
Pe.car.1139-PC1_3614-YP_003019165.1--36H-COG26
Pe.car.1139-PC1_2223-YP_003017798.1--36H-COG27
Pe.car.1139-PC1_1993-YP_003017570.1--36H-COG32

Pectobacterium sp. SCC3193
Pectob.2320-W5S_1768-YP_006282731.1--36H-COG1
Pectob.2320-W5S_3114-YP_006284066.1--36H-COG1
Pectob.2320-W5S_1886-YP_006282848.1--36H-COG1
Pectob.2320-W5S_1696-YP_006282659.1--36H-COG1
Pectob.2320-W5S_3170-YP_006284116.1--36H-COG1
Pectob.2320-W5S_2813-YP_006283770.1--36H-COG2
Pectob.2320-W5S_3736-YP_006284671.1--36H-COG2
Pectob.2320-W5S_4505-YP_006285423.1--36H-COG2
Pectob.2320-W5S_1733-YP_006282696.1--36H-COG2
Pectob.2320-W5S_4506-YP_006285424.1--36H-COG2
Pectob.2320-W5S_0106-YP_006281117.1--36H-COG2
Pectob.2320-W5S_3753-YP_006284688.1--36H-COG2
Pectob.2320-W5S_3696-YP_006284631.1--36H-COG3
Pectob.2320-W5S_2564-YP_006283524.1--36H-COG3
Pectob.2320-W5S_4621-YP_006285539.1--36H-COG4
Pectob.2320-W5S_1965-YP_006282927.1--36H-COG4
Pectob.2320-W5S_4632-YP_006285550.1--36H-COG4
Pectob.2320-W5S_3740-YP_006284675.1--36H-COG5
Pectob.2320-W5S_3708-YP_006284643.1--36H-COG5
Pectob.2320-W5S_1760-YP_006282723.1--36H-COG6
Pectob.2320-W5S_4020-YP_006284955.1--36H-COG6
Pectob.2320-W5S_1146-YP_006282119.1--36H-COG7
Pectob.2320-W5S_3303-YP_006284249.1--36H-COG8
Pectob.2320-W5S_3731-YP_006284666.1--36H-COG9
Pectob.2320-W5S_2351-YP_006283311.1--36H-COG10
Pectob.2320-W5S_0006-YP_006281017.1--36H-COG10

Pectob.2320-W5S_0520-YP_006281518.1--36H-COG15
Pectob.2320-W5S_4243-YP_006285171.1--36H-COG16
Pectob.2320-W5S_0518-YP_006281516.1--36H-COG17
Pectob.2320-W5S_0443-YP_006281442.1--36H-COG20
Pectob.2320-W5S_2016-YP_006282978.1--36H-COG21
Pectob.2320-W5S_0107-YP_006281118.1--36H-COG22
Pectob.2320-W5S_0105-YP_006281116.1--36H-COG24
Pectob.2320-W5S_3942-YP_006284877.1--36H-COG26
Pectob.2320-W5S_2511-YP_006283471.1--36H-COG27
Pectob.2320-W5S_2238-YP_006283198.1--36H-COG32
Pectob.2320-W5S_1964-YP_006282926.1--36H-COG33
Pectob.2320-W5S_0108-YP_006281119.1--36H-COG57

Photorhabdus asymbiotica
Ph.asy.1114-PAU_02685-YP_003041519.1--36H-COG1
Ph.asy.1114-PAU_02684-YP_003041518.1--36H-COG1

Photorhabdus luminescens subsp. laumondii TTO1
Ph.lum.1262-plu1853-NP_929126.1--36H-COG1
Ph.lum.1262-plu1854-NP_929127.1--36H-COG1

Proteus mirabilis HI4320
Pr.mir.1265-PMI1666-YP_002151397.1--36H-COG1
Pr.mir.1265-PMI1665-YP_002151396.1--36H-COG1
Pr.mir.1265-PMI2808-YP_002152509.1--36H-COG1
Pr.mir.1265-PMI2809-YP_002152510.1--36H-COG1
Pr.mir.1265-PMI2813-YP_002152514.1--36H-COG3
Pr.mir.1265-PMI2381-YP_002152099.1--36H-COG58
Pr.mir.1265-PMI1180-YP_002150913.1--36H-COG70
Pr.mir.1265-PMI2380-YP_002152098.1--36H-COG76
Pr.mir.1265-PMI2671-YP_002152372.1--Uncat-COG82

Providencia stuartii MRSN 2154
Pr.stu.1965-S70_18135-YP_006218124.1--36H-COG1
Pr.stu.1965-S70_18140-YP_006218125.1--36H-COG1
Pr.stu.1965-S70_05790-YP_006215722.1--36H-COG2
Pr.stu.1965-S70_14535-YP_006217426.1--36H-COG3
Pr.stu.1965-S70_01415-YP_006214870.1--36H-COG87

Rahnella aquatilis HX2
Ra.aqu.1678-Q7S_08960-YP_005401597.1--36H-COG1
Ra.aqu.1678-Q7S_16025-YP_005402985.1--36H-COG1
Ra.aqu.1678-Q7S_08965-YP_005401598.1--36H-COG1
Ra.aqu.1678-Q7S_14450-YP_005402672.1--36H-COG1
Ra.aqu.1678-Q7S_24891-YP_005419232.1--36H-COG2
Ra.aqu.1678-Q7S_15665-YP_005402913.1--36H-COG2
Ra.aqu.1678-Q7S_18035-YP_005403387.1--36H-COG2
Ra.aqu.1678-Q7S_23326-YP_005418927.1--36H-COG3
Ra.aqu.1678-Q7S_00195-YP_005399878.1--36H-COG4
Ra.aqu.1678-Q7S_13620-YP_005402506.1--36H-COG4
Ra.aqu.1678-Q7S_02285-YP_005400289.1--36H-COG5
Ra.aqu.1678-Q7S_09815-YP_005401764.1--36H-COG6
Ra.aqu.1678-Q7S_00910-YP_005400016.1--36H-COG7
Ra.aqu.1678-Q7S_04670-YP_005400757.1--36H-COG8
Ra.aqu.1678-Q7S_02185-YP_005400269.1--36H-COG9
Ra.aqu.1678-Q7S_06520-YP_005401115.1--24H-COG11
Ra.aqu.1678-Q7S_07590-YP_005401329.1--36H-COG12
Ra.aqu.1678-Q7S_22515-YP_005404275.1--36H-COG13
Ra.aqu.1678-Q7S_23331-YP_005418928.1--36H-COG14
Ra.aqu.1678-Q7S_20535-YP_005403883.1--36H-COG15

Rahnella sp. Y9602
Rahnel.1320-Rahaq_1830-YP_004212575.1--36H-COG1
Rahnel.1320-Rahaq_3177-YP_004213898.1--36H-COG1
Rahnel.1320-Rahaq_1831-YP_004212576.1--36H-COG1
Rahnel.1320-Rahaq_2866-YP_004213595.1--36H-COG1
Rahnel.1320-Rahaq_4869-YP_004215574.1--36H-COG2
Rahnel.1320-Rahaq_3107-YP_004213828.1--36H-COG2
Rahnel.1320-Rahaq_3579-YP_004214298.1--36H-COG2
Rahnel.1320-Rahaq_4561-YP_004215269.1--36H-COG3
Rahnel.1320-Rahaq_0041-YP_004210801.1--36H-COG4
Rahnel.1320-Rahaq_2735-YP_004213465.1--36H-COG4
Rahnel.1320-Rahaq_0458-YP_004211214.1--36H-COG5
Rahnel.1320-Rahaq_1930-YP_004212674.1--36H-COG6
Rahnel.1320-Rahaq_0181-YP_004210938.1--36H-COG7
Rahnel.1320-Rahaq_0984-YP_004211735.1--36H-COG8
Rahnel.1320-Rahaq_0438-YP_004211194.1--36H-COG9
Rahnel.1320-Rahaq_1352-YP_004212102.1--24H-COG11
Rahnel.1320-Rahaq_1570-YP_004212319.1--36H-COG12
Rahnel.1320-Rahaq_4432-YP_004215145.1--36H-COG13
Rahnel.1320-Rahaq_4562-YP_004215270.1--36H-COG14
Rahnel.1320-Rahaq_4041-YP_004214756.1--36H-COG15

Salmonella bongori NCTC 12419
Sa.bon.1474-SBG_3944-YP_004732724.1--36H-COG1
Sa.bon.1474-SBG_2750-YP_004731563.1--36H-COG1
Sa.bon.1474-SBG_3170-YP_004731982.1--36H-COG1
Sa.bon.1474-SBG_1755-YP_004730609.1--36H-COG1
Sa.bon.1474-SBG_2851-YP_004731664.1--36H-COG2
Sa.bon.1474-SBG_2852-YP_004731665.1--36H-COG3
Sa.bon.1474-SBG_1454-YP_004730321.1--36H-COG6
Sa.bon.1474-SBG_1497-YP_004730358.1--36H-COG18
Sa.bon.1474-SBG_2737-YP_004731551.1--Uncat-COG25

Salmonella enterica subsp. enterica serovar Typhi str. CT18
Sa.ent.404-STY2128-NP_456485.1--36H-COG1
Sa.ent.404-STY4234-NP_458344.1--36H-COG1
Sa.ent.404-STY3394-NP_457606.1--36H-COG2
Sa.ent.404-STY3395-NP_457607.1--36H-COG3
Sa.ent.404-STY1408-NP_455847.1--36H-COG18
Sa.ent.404-STY3309-NP_457533.1--Uncat-COG25

Salmonella enterica subsp. enterica serovar Typhimurium str. LT2
Sa.ent.407-STM4533-NP_463392.1--36H-COG1
Sa.ent.407-STM3152-NP_462067.1--36H-COG1
Sa.ent.407-STM3577-NP_462478.1--36H-COG1
Sa.ent.407-STM1919-NP_460876.1--36H-COG1
Sa.ent.407-STM3216-NP_462130.1--36H-COG2
Sa.ent.407-STM3217-NP_462131.1--36H-COG3
Sa.ent.407-STM1626-NP_460585.1--36H-COG6
Sa.ent.407-STM1657-NP_460616.1--36H-COG18
Sa.ent.407-STM3138-NP_462053.1--Uncat-COG25

Serratia marcescens FGI94
Se.mar.2260-D781_2759-YP_007345195.1--36H-COG1
Se.mar.2260-D781_2758-YP_007345194.1--36H-COG1
Se.mar.2260-D781_0032-YP_007342620.1--36H-COG4
Se.mar.2260-D781_0365-YP_007342916.1--36H-COG5
Se.mar.2260-D781_1404-YP_007343896.1--24H-COG11
Se.mar.2260-D781_1283-YP_007343780.1--36H-COG17

Serratia plymuthica AS9
Se.ply.1407-SerAS9_3072-YP_004506452.1--36H-COG1
Se.ply.1407-SerAS9_3071-YP_004506451.1--36H-COG1
Se.ply.1407-SerAS9_0039-YP_004503465.1--36H-COG4
Se.ply.1407-SerAS9_0403-YP_004503820.1--36H-COG5

Serratia proteamaculans 568
Se.pro.864-Spro_2983-YP_001479212.1--36H-COG1
Se.pro.864-Spro_2982-YP_001479211.1--36H-COG1
Se.pro.864-Spro_1415-YP_001477647.1--36H-COG1
Se.pro.864-Spro_0080-YP_001476318.1--36H-COG4
Se.pro.864-Spro_0463-YP_001476699.1--36H-COG5
Se.pro.864-Spro_1472-YP_001477704.1--24H-COG11

Serratia sp. AS12
Serrat.1408-SerAS12_3073-YP_004501499.1--36H-COG1
Serrat.1408-SerAS12_3072-YP_004501498.1--36H-COG1
Serrat.1408-SerAS12_0039-YP_004498513.1--36H-COG4
Serrat.1408-SerAS12_0403-YP_004498868.1--36H-COG5

Serratia sp. AS13
Serrat.1901-SerAS13_3075-YP_006025914.1--36H-COG1
Serrat.1901-SerAS13_3074-YP_006025913.1--36H-COG1
Serrat.1901-SerAS13_0039-YP_006022928.1--36H-COG4
Serrat.1901-SerAS13_0403-YP_006023283.1--36H-COG5

Xenorhabdus bovienii SS-2004
Xe.bov.105-XBJ1_1923-YP_003467827.1--36H-COG1
Xe.bov.105-XBJ1_1924-YP_003467828.1--36H-COG1

Xenorhabdus nematophila ATCC 19061
Xe.nem.162-XNC1_1620-YP_003711881.1--36H-COG1
Xe.nem.162-XNC1_1619-YP_003711880.1--36H-COG1

Yersinia enterocolitica subsp. enterocolitica 8081
Ye.ent.378-YE2575-YP_001006778.1--36H-COG1
Ye.ent.378-YE2573-YP_001006777.1--36H-COG1
Ye.ent.378-YE2971-YP_001007160.1--36H-COG1
Ye.ent.378-YE1731-YP_001006015.1--36H-COG3
Ye.ent.378-YE4139-YP_001008275.1--36H-COG4
Ye.ent.378-YE0409-YP_001004781.1--36H-COG5
Ye.ent.378-YE2588-YP_001006789.1--36H-COG6
Ye.ent.378-YE1405-YP_001005717.1--36H-COG8
Ye.ent.378-YE3338-YP_001007509.1--36H-COG12
Ye.ent.378-YE4183-YP_001008317.1--36H-COG13
Ye.ent.378-YE3615-YP_001007773.1--36H-COG69
Ye.ent.378-YE2848-YP_001007037.1--24H-COG71

Yersinia pestis Z176003
Ye.pes.133-YPZ3_2024-YP_003568196.1--36H-COG1
Ye.pes.133-YPZ3_2023-YP_003568195.1--36H-COG1
Ye.pes.133-YPZ3_2049-YP_003568221.1--36H-COG3
Ye.pes.133-YPZ3_3484-YP_003569655.1--36H-COG4
Ye.pes.133-YPZ3_1131-YP_003567303.1--36H-COG8
Ye.pes.133-YPZ3_2157-YP_003568329.1--24H-COG11
Ye.pes.133-YPZ3_0903-YP_003567075.1--36H-COG12
Ye.pes.133-YPZ3_2040-YP_003568212.1--Uncat-COG54
Ye.pes.133-YPZ3_2039-YP_003568211.1--36H-COG75

Yersinia pseudotuberculosis IP 32953
Ye.pse.585-YPTB2401-YP_070915.1--36H-COG1
Ye.pse.585-YPTB2400-YP_070914.1--36H-COG1
Ye.pse.585-YPTB2424-YP_070937.1--36H-COG3
Ye.pse.585-YPTB3924-YP_072400.1--36H-COG4
Ye.pse.585-YPTB2412-YP_070925.1--36H-COG6
Ye.pse.585-YPTB1265-YP_069800.1--36H-COG8
Ye.pse.585-YPTB2551-YP_071061.1--24H-COG11
Ye.pse.585-YPTB3051-YP_071556.1--36H-COG12
